# Sustained complete response to TMEp-CI-M platform in refractory small-cell lung cancer with brainstem metastasis: a case report with over 20 months of disease-free survival

**DOI:** 10.3389/fimmu.2026.1807865

**Published:** 2026-06-01

**Authors:** Yating Wu, Hang Li, Yonghai Peng, Weiqiang Fan

**Affiliations:** Department of Oncology, The Cangshan District of the 900th Hospital, Fuzhou, Fujian, China

**Keywords:** case report, immunotherapy, small-cell lung cancer, TME (tumor microenvironment), TMEp-CI-M platform

## Abstract

**Background:**

Brainstem metastasis from small-cell lung cancer (SCLC) is exceedingly rare and is associated with a dismal prognosis. This study presents a case of brainstem metastasis from SCLC treated with the TMEp-CI-M platform, achieving no evidence of disease (NED) for more than 20 months. The TMEp-CI-M platform is designed to overcome resistance in immunologically “cold” tumors through sequential tumor microenvironment priming (TMEp), checkpoint inhibition (CI), and microbiome modulation. We have previously reported its efficacy in pancreatic neuroendocrine carcinoma, hepatocellular carcinoma, pancreatic ductal adenocarcinoma, non-small cell lung cancer (NSCLC), and colorectal cancer.

**Case introduction:**

A 60-year-old male with programmed death ligand 1 (PD-L1)-negative extensive-stage small-cell lung cancer (ES-SCLC) and brainstem metastasis received the TMEp-CI-M regimen. The TMEp phase integrated stereotactic body radiotherapy (SBRT), low-dose etoposide, and anlotinib, followed by CI with the programmed death 1 (PD-1)/cytotoxic T lymphocyte antigen 4 (CTLA-4) bispecific antibody cadonilimab and concurrent probiotic supplementation. The patient’s pro-gastrin-releasing peptide (ProGRP) level normalized after the first cycle (from 1803 pg/mL to 23.71 pg/mL) during a total of 6 treatment cycles. At the time of this report (20 months after treatment initiation), the patient remains NED, with only Grade 1 hypothyroidism as an adverse event.

**Conclusion:**

The TMEp-CI-M platform may enhance the efficacy of immunotherapy in ES-SCLC, enabling durable responses even in patients with brainstem metastases. Although this platform has demonstrated promise across multiple tumor types, further prospective and mechanistic studies are warranted to confirm its clinical utility.

## 1 Introduction

Small-cell lung cancer (SCLC) is a highly aggressive neuroendocrine malignancy. Approximately 15% of patients present with brain metastases (BM) at the time of initial diagnosis (1), and the median overall survival for this population is approximately 10 months (2). Brainstem metastases constitute a rare subset of BM in SCLC, accounting for only 1-4% of cases with intracranial involvement (3). Given the brainstem’s essential role in regulating vital physiological functions, including respiration, cardiac activity, and consciousness, metastatic involvement of this “vital zone” presents substantial clinical challenges.

Systemic treatment for SCLC with BM generally follows therapeutic strategies established for extensive-stage disease. In the landmark IMpower133 trial, the addition of atezolizumab to platinum-etoposide chemotherapy improved median progression-free survival from 4.3 months with chemotherapy alone to 5.2 months, and extended median overall survival from 10.3 to 12.3 months. These results established atezolizumab combined with carboplatin and etoposide as a new standard first-line regimen for ES-SCLC, marking the advent of immunotherapy in the management of this aggressive malignancy (4). SCLC, a typical representative of immune-”cold” tumors, is characterized by a profoundly immunosuppressive tumor microenvironment (TME), low PD-L1 expression, and rapid metastasis (5). It has benefited little from immune checkpoint inhibitors (ICIs), underscoring the urgent need for strategies that fundamentally reshape tumor-immune crosstalk.

We have previously conceptualized and clinically implemented the tumor microenvironment priming (TMEp), checkpoint inhibition (CI), and microbiome modulation (TMEp-CI-M) platform, a structured approach designed to initiate, amplify, and sustain a systemic anti-tumor immune response. The TMEp−CI−M platform and the previously designated “BRICS regimen” represent the same therapeutic framework, sharing identical mechanistic principles and conceptual foundations. BRICS serves as the clinical acronym for this combination regimen, whereas TMEp−CI−M denotes its standardized mechanistic nomenclature (6, 7). The platform is based on three aspects. First, in the TMEp phase, SBRT-based multi-mechanistic interventions are used to disrupt the immunosuppressive niche, induce immunogenic cell death (ICD), and release a heterogeneous antigen repertoire. Second, CI is employed to unleash the primed T-cell response. Third, concurrent microbiome modulation aims to optimize the host’s immune milieu to enhance efficacy and tolerability.

Our prior work has observed encouraging clinical activity of this platform (**Table 1**). Complete response (CR) over 7 years in a patient with advanced pancreatic neuroendocrine carcinoma first confirmed its potential (8). A subsequent prospective study in unresectable hepatocellular carcinoma proved the platform’s efficacy and safety (9). Recent retrospective studies further corroborated its feasibility and effectiveness. It achieved a median overall survival of 32.7 months in programmed death ligand 1 (PD-L1)-negative metastatic non-small cell lung cancer (NSCLC) (7). An objective response rate of 33.3% with a median survival of 12.3 months in refractory advanced metastatic colorectal cancer (6), and a case of sustained CR was also documented in locally advanced pancreatic ductal adenocarcinoma (10), both with favorable tolerability. Here, we report the application of the TMEp-CI-M platform in a patient with extensive-stage small-cell lung cancer (ES-SCLC) and brainstem metastasis. The patient maintained a sustained no evidence of disease (NED) status for more than 20 months, providing meaningful clinical support for further investigation of this platform in ES-SCLC.

**Table 1:** Summary of clinical studies evaluating TMEp-CI-M across multiple cancer types.

Cancer type	Study design	Sample size (n)	Line of therapy	TMEp-CI-M			Cycles (immunotherapy)	Primary efficacy	Safety profile
				TMEp	CI	M			
aPNEC (8)	Case report	1	Second-line	(1) SBRT: 8 Gy/fraction, 16 Gy/2 fractions (pancreas); 24 Gy/3 fractions (lymph nodes); (2) I-125 brachytherapy: 36 seeds, 0.6 mCi/seed (intrahepatic lesion)	Pembrolizumab (200 mg IV q3w)	Bifidobacterium triple viable capsule (6 g BID, indefinitely)	14 cycles	NED>7 years	No severe AEs reported
uHCC (9)	Prospective study (ChiCTR2000032533)	20	Predominantly First-line	(1) SBRT: 8 Gy/fraction, 24 Gy/3 fractions (single intrahepatic lesion); (2) Anlotinib: 12 mg/day, 2 weeks on/1 week off	Toripalimab (240 mg IV q3w)	Bifidobacterium triple viable capsule (6 g BID, indefinitely)	Median: 3 cycles (Range: 1-12)	ORR: 15.0%; DCR: 50.0%; mPFS: 7.4 months; 24-month OS: 50.9%	Gr 3-4: 10% (diarrhea, hyperbilirubinemia); Gr 5: 5% (upper GI hemorrhage)
mNSCLC (7)	Retrospective study	23	First-line: 47.8% Second-line or later: 52.2%	(1) SBRT: 8 Gy/fraction, 24 Gy/3 fractions (single metastatic lesion; lung most common, 69.6%); (2) Nab-paclitaxel (200 mg IV q21d) or pemetrexed/lobaplatin	Toripalimab (47.8%), camrelizumab (39.3%), others (sintilimab, tislelizumab, pembrolizumab)	Bifidobacterium/Lactobacillus preparation (6 g/day, indefinitely)	Completed 6 cycles: 30.4% Received 4–6 cycles: 69.6%	ORR: 95.7%; DCR: 95.7%; mPFS: 16.0 months; mOS: 32.7 months	Gr 1–2 AEs only: fatigue (13.2%), rash (8.7%), thrombocytopenia (8.7%); No Gr ≥3 AEs
mCRC (6)	Retrospective study	27	Third-line or later: 70.4% Second-line: 29.6%	(1) SBRT: 5–8 Gy/fraction, 25–40 Gy/3–5 fractions (single metastatic lesion; liver most common, 51.9%); (2) Low-dose mFOLFOX; (3) TKIs: regorafenib (40.7%), anlotinib (22.3%), others	Sintilimab (25.9%), toripalimab (25.9%), camrelizumab (18.5%), others	Bifidobacterium longum (S19980004), 2 × 10^8^ CFU/day, indefinitely	Completed 6 cycles: 37.0% Received <6 cycles: 63.0%	ORR: 33.3%; DCR: 88.9%; mPFS: 7.2 months; mOS: 12.3 months	Gr 3–4 AEs: 22.2% (neutropenia 14.8%, thrombocytopenia 3.7%, anemia 3.7%); No Gr 5 AEs
LA PDAC (10)	Case report	1	First-line	(1) SBRT: 8 Gy/fraction, 24 Gy/3 fractions (primary pancreatic lesion); (2) Nab-paclitaxel (200 mg IV q21d); (3) Anlotinib: 12 mg/day, 2 weeks on/1 week off	Toripalimab (240 mg IV q3w)	Bifidobacterium triple viable capsule (6 g BID, indefinitely)	6 cycles	Sustained complete response for>5 months after treatment	Gr 2 neutropenia

AE, adverse event; aPNEC, advanced pancreatic neuroendocrine carcinoma; CI, checkpoint inhibition; CR, complete response; DCR, disease control rate; IV, intravenous; LA PDAC, locally advanced pancreatic ductal adenocarcinoma; mCRC, metastatic colorectal cancer; mNSCLC, metastatic non-small cell lung cancer; mOS, median overall survival; mPFS, median progression-free survival; NED, no evidence of disease; ORR, objective response rate; OS, overall survival; PDAC, pancreatic ductal adenocarcinoma; PFS, progression-free survival; SBRT, stereotactic body radiotherapy; TKI, tyrosine kinase inhibitor; TMEp-CI-M, tumor microenvironment priming-checkpoint inhibition-microbiome modulation; uHCC, unresectable hepatocellular carcinoma.

## 2 Case presentation

A 60-year-old male presented with dizziness and cough for one month. Physical examination revealed no significant abnormalities, and the baseline Eastern Cooperative Oncology Group (ECOG) performance status was 2. Neurological examination was unremarkable. The patient was alert, fully oriented, and demonstrated intact higher cortical functions. Pupils were equal, round, and bilaterally reactive to light. Extraocular movements were full, without diplopia or nystagmus. Facial sensation was symmetric, and masticatory muscle strength was preserved. Bilateral forehead wrinkling and nasolabial folds were symmetric, with no mouth deviation on showing the teeth. Hearing was grossly intact. Palatal elevation was symmetric, the gag reflex was preserved, speech was clear, and no dysphagia or aspiration on water swallowing was observed. The tongue was midline without atrophy or fasciculations. Neck rotation and shoulder shrug strength were symmetric and intact. Muscle strength was graded 5/5 in all four extremities, with normal muscle tone. Deep tendon reflexes, including the biceps, triceps, patellar, and Achilles reflexes, were symmetric bilaterally (2+). Plantar responses were flexor bilaterally (negative Babinski sign). Finger-to-nose and heel-knee-shin testing showed intact coordination, and rapid alternating movements were performed smoothly. The Romberg sign was negative, and the gait was normal. Sensory examination was grossly intact.

Baseline laboratory investigations, including complete blood count, liver and renal function tests, and serum electrolyte levels, were all within normal limits. Serial laboratory evaluations during follow-up likewise revealed no significant abnormalities. At diagnosis, the serum pro-gastrin-releasing peptide (ProGRP) level was markedly elevated at 1803 pg/mL.

**Figure 1:**
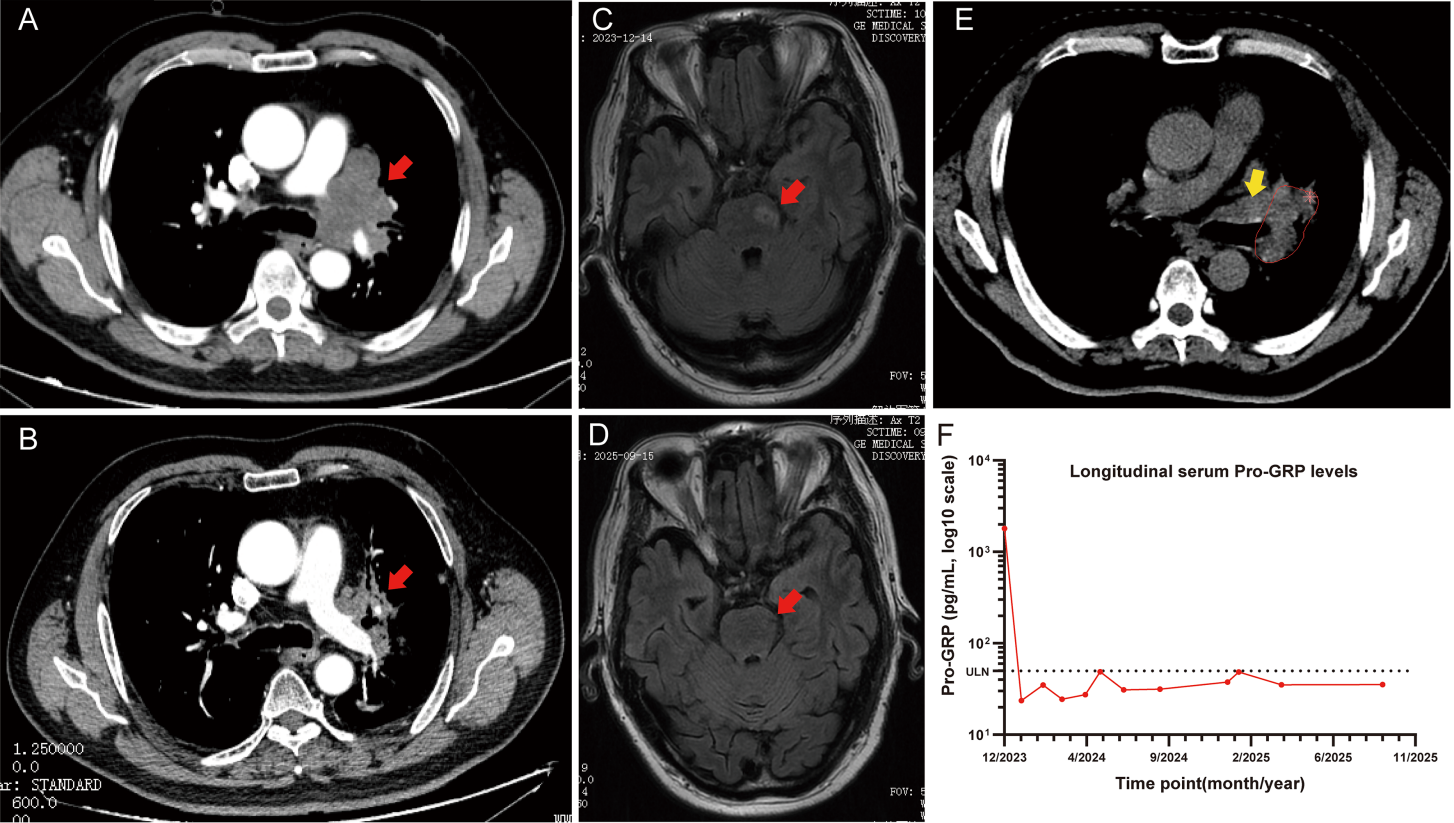
**(A–D)** Comparative imaging before and after treatment. **(A)** Baseline contrast-enhanced chest CT scan of the primary lung lesion. **(C)** Baseline contrast-enhanced brain MRI scan of the brainstem lesion. **(B, D)** show the most recent post-treatment follow-up images corresponding to **(A, C)**, respectively. **(E)** Delineation of the gross tumor volume (GTV). The red contour outlines the primary GTV, While yellow arrows indicate the primary lung lesions that were not irradiated as a result of radiation field reduction (target volume shrinkage) within the lung. **(F)** Longitudinal changes in pro-gastrin-releasing peptide (pro-GRP) levels during the treatment course. X-axis: time point (month/year). Y-axis: Pro-GRP (pg/mL) displayed on a log10 scale. The dashed line indicates the upper limit of normal (ULN, 50 pg/mL). The graph was generated using GraphPad Prism software (version 10).

**Figure 2:**
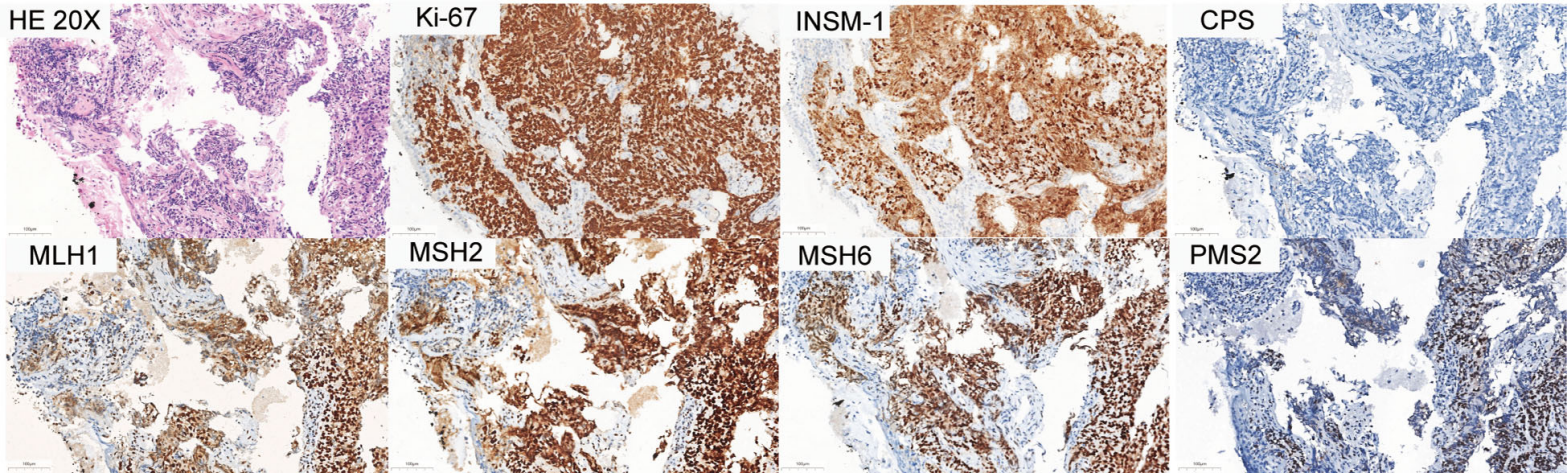
Immunohistochemical (IHC) Pathology Image. **(A)** Hematoxylin and eosin (HE) staining at 20× magnification. **(B–H)** Immunostaining shows: high Ki-67 proliferation index (90%); strong INSM-1 positivity; PD-L1 combined positive score (CPS) of 0; and retained nuclear expression of mismatch repair proteins MLH1, MSH2, MSH6, and PMS2 (proficient mismatch repair, pMMR). Results are consistent with the pathology profile summarized in **Supplementary Table 1**.

Initial imaging demonstrated a 6.8×4.8 cm left hilar mass encasing the pulmonary artery, together with a solitary 1.3×0.6 cm brainstem metastasis (**Figures 1A, C**; **Supplementary Figures 1**, **2**). Owing to the eloquent and high-risk location of the brainstem lesion, biopsy was not feasible. Consequently, the diagnosis was established on the basis of histopathological and immunohistochemical (IHC) analyses of the primary pulmonary lesion. Biopsy of the left hilar mass confirmed small cell lung carcinoma (SCLC). Tumor cells showed diffuse positivity for synaptophysin (Syn), CD56, INSM1, and broad-spectrum cytokeratin (CKP), together with nuclear expression of thyroid transcription factor-1 (TTF-1). The Ki-67 proliferation index was 90%. Additionally, IHC findings included loss of RB1 expression and a mutant-pattern p53 expression profile, with approximately 90% positivity. Programmed death-ligand 1 (PD-L1) combined positive score (CPS) was 0, while mismatch repair proteins were intact (pMMR; MLH1, MSH2, MSH6, and PMS2 all positive) (**Figure 2**; **Supplementary Figure 3**; **Supplementary Table 1**). Collectively, these findings supported a diagnosis of ES-SCLC.

The differential diagnosis for a left hilar mass accompanied by a solitary brainstem lesion includes primary SCLC with isolated BM, large-cell neuroendocrine carcinoma (LCNEC), atypical carcinoid tumor, primary central nervous system lymphoma (PCNSL), and inflammatory pseudotumor. In the absence of brainstem tissue for pathological confirmation, the diagnosis relied heavily on the characteristic IHC profile of the pulmonary lesion. Diffuse expression of Syn, CD56, INSM1, and TTF-1, together with RB1 loss and a TP53-mutant expression pattern, strongly favored SCLC over other neuroendocrine neoplasms. Moreover, the markedly elevated Ki-67 index (90%) effectively excluded low- and intermediate-grade neuroendocrine tumors. PCNSL was considered unlikely because brain imaging lacked the characteristic contrast-enhancing periventricular or deep white matter lesions typically associated with lymphoma, and there were no systemic manifestations suggestive of lymphoproliferative disease. Comprehensive systemic imaging identified no extrapulmonary primary malignancy, and the patient’s smoking history further supported the diagnosis of primary SCLC with solitary brainstem metastasis.

A major diagnostic challenge was the inability to obtain tissue from the brainstem lesion, a common dilemma in neuro-oncology practice. Solitary brainstem metastases are uncommon, and biopsy is frequently precluded by excessive surgical risk. Therefore, the diagnosis of BM was based entirely on radiological features, including contrast enhancement, perilesional edema, and anatomical localization, in the setting of pathologically confirmed primary SCLC. The possibility of synchronous primary malignancies, namely primary pulmonary SCLC coexisting with an independent primary brain tumor such as glioma or PCNSL, was also considered. However, this scenario was regarded as unlikely for several reasons. First, the patient presented with a one-month history of both dizziness and cough, suggesting near-simultaneous clinical onset of the brainstem and pulmonary lesions. Second, the lung mass was substantially larger (6.8×4.8 cm) than the brainstem lesion (1.3×0.6 cm), favoring a primary pulmonary neoplasm with secondary intracranial dissemination rather than two unrelated primary tumors. Third, the highly aggressive biological features of SCLC, including a Ki-67 index of 90%, RB1 loss, and TP53 mutation, are well recognized to predispose to early hematogenous spread, including metastasis to the central nervous system. Finally, the IHC profile of the pulmonary lesion was entirely characteristic of SCLC. Taken together, the clinical, radiological, and pathological evidence strongly supported a unifying diagnosis of primary SCLC with solitary brainstem metastasis rather than concurrent dual primary malignancies.

The patient’s SCLC-specific graded prognostic assessment (SCLC-GPA) score was 3, corresponding to a median overall survival of approximately 14 months. Although the brainstem metastasis was solitary, its critical anatomical location may confer a poorer prognosis because of limited therapeutic options and an increased risk of neurological deterioration.

The patient had a smoking history. There was no notable prior medical history. Family history was unremarkable for malignancy or hereditary cancer syndromes. Psychosocial assessment revealed that he lived with his spouse and had strong family support. He denied alcohol consumption and illicit drug use. The patient was retired and reported no substantial financial or occupational stressors.

### 2.1 Therapeutic intervention and timeline

Given the patient’s refusal of standard platinum-based chemotherapy combined with immunotherapy, and inspired by our prior successful experience, an individualized treatment regimen was requested by the patient for consideration. After informed consent was obtained, treatment was administered according to the established TMEp-CI-M sequence (**Table 2**).

**Table 2:** Timeline of diagnosis and treatment.

Timeline	Regimen/ occurrence	Details	Duration	Cycles	ProGRP (pg/mL)	Notes
2023-12	Definitive Diagnosis	Extensive-stage SCLC with brainstem metastasis (Ki-67: 90%, CPS 0, pMMR)	–	–	1803	–
2023-12-18	Probiotics	Bifidobacterium triple viable capsule, 6 g BID	Continuous	Continuous	–	–
2023-12-18	SBRT	Pulmonary lesion; 24 Gy in 3 fractions (8 Gy/fraction)	3 days	1	–	–
2023-12-22	Combination Therapies	Etoposide 0.1 g on days 1-2 + Cadonilimab 375 mg on day 3 + Anlotinib 12 mg on days 1–14 every 21 days	2 weeks	1/6	–	–
2024-01-10	SBRT	Brainstem metastases; 25 Gy in 5 fractions (5 Gy/fraction)	5 days	1	23.71	ProGRP normalized
2024-01-18	Combination Therapies	Etoposide 0.1 g on days 1-2 + Cadonilimab 375 mg on day 3 + Anlotinib 12 mg on days 1–14 every 21 days	2 weeks	2/6	–	Sustained tumor regression
2024-02-22	Combination Therapies	Etoposide 0.1 g on days 1-2 + Cadonilimab 375 mg on day 3 + Anlotinib 12 mg on days 1–14 every 21 days	2 weeks	3/6	34.9	NED
2024-04-01	Combination Therapies	Etoposide 0.1 g on days 1-2 + Cadonilimab 375 mg on day 3 + Anlotinib 12 mg on days 1–14 every 21 days	2 weeks	4/6	24.48	NED
2024-04-28	Combination Therapies	Etoposide 0.1 g on days 1-2 + Cadonilimab 375 mg on day 3 + Anlotinib 12 mg on days 1–14 every 21 days	2 weeks	5/6	27.4	NED
2024-05-24	Combination Therapies	Etoposide 0.1 g on days 1-2 + Cadonilimab 375 mg on day 3 + Anlotinib 12 mg on days 1–14 every 21 days	2 weeks	6/6	48.77	NED
2024-07-02	Follow-up	–	–	–	30.99	NED
2024-09-02	Follow-up	–	–	–	31.5	NED
2024-12-26	Follow-up	–	–	–	37.72	NED
2025-01-14	Follow-up	–	–	–	48.53	NED
2025-03-28	Follow-up	–	–	–	35.24	NED
2025-09-16	Follow-up	–	–	–	35.45	NED

CPS, Combined Positive Score; NED, no evidence of disease; pMMR, proficient mismatch repair; ProGRP, progastrin-releasing peptide; SBRT, stereotactic body radiotherapy; SCLC, small cell lung cancer.

Stage 1: TMEp. This phase was designed as a synergistic attack on the SCLC TME to overcome spatial heterogeneity.

-Stereotactic Body Radiotherapy (SBRT): SBRT was delivered to the primary lung tumor (**Supplementary Figure 4**) to locally trigger ICD and act as an *in situ* vaccine. The dose was prescribed as 8 Gy×3 fractions (total dose, 24 Gy), selected on the basis of published evidence supporting its immunomodulatory and potential abscopal effects rather than conventional palliative fractionation schedules. To reduce the risk of severe radiotherapy-related adverse events, the clinical target volume of the primary lung lesion was modestly reduced (**Figure 1E**). Before the second treatment cycle, SBRT at 5 Gy×5 fractions was administered to the brainstem metastasis (**Supplementary Figure 5**). This fractionation regimen was chosen to balance effective tumor control with the critical requirement for safety in this highly eloquent central nervous system region. The moderately hypofractionated schedule provides a biologically effective dose sufficient for radiosensitive SCLC while remaining within accepted brainstem tolerance limits, thereby minimizing the risk of radiation-induced necrosis and neurological toxicity.

-Low-Dose Chemotherapy: The patient received low-dose etoposide (0.1 g on days 1–2 per cycle), aiming to target proliferating immunosuppressive cells and potentially enhance antigen presentation. This dose was chosen to minimize myelosuppression and gut dysbiosis while preserving ICD and dendritic cell (DC) activation, because standard cytotoxic doses may exacerbate immunosuppression.

-Anti-angiogenic Therapy: The tyrosine kinase inhibitor anlotinib (12 mg daily, two weeks on/one week off) was administered to promote vascular normalization, improve immune cell infiltration, and target hypoxia-driven resistance pathways.

The synergy of SBRT, low-dose chemotherapy, and anti-angiogenic therapy was intended to release more comprehensive antigens from heterogeneous tumor clones and to shape a more favorable immune microenvironment, resulting in stronger immunostimulation.

Stage 2: CI. The PD-1/CTLA-4 bispecific antibody cadonilimab (375 mg on day 3 per cycle) was used to concurrently block two critical immune checkpoints, aiming to amplify the immune response initiated during the TMEp phase.

Stage 3: Microbiome Modulation. Oral probiotics (Bifidobacterium triple viable tablet, 6 g twice daily) were administered throughout the treatment course, since a favorable gut microbiome may enhance efficacy and mitigate toxicity of ICIs.

Although this therapeutic strategy deviated from guideline-recommended first-line treatment for ES-SCLC, it was considered justified in light of the patient’s refusal of standard-of-care therapy and the exceptionally poor prognosis associated with brainstem metastasis. All treatment decisions were made following comprehensive informed consent and under close safety surveillance.

## 3 Results and follow-up

The patient showed rapid clinical responses. After the first cycle, his cough resolved, and the serum ProGRP level normalized to 23.71 pg/mL, and ECOG performance status improved from 2 to 0. The patient completed all 6 planned treatment cycles (21 days per cycle; total treatment duration: approximately 18 weeks). The regimen was well-tolerated. The only adverse event observed was Grade 1 hypothyroidism, which was effectively managed with levothyroxine supplementation. Serial radiological assessments of extracranial and intracranial tumor responses were conducted according to RECIST version 1.1 and the Response Assessment in Neuro-Oncology Brain Metastases (RANO-BM) criteria, respectively. The patient did not undergo positron emission tomography-computed tomography (PET-CT). Contrast-enhanced chest CT, evaluated according to RECIST 1.1, was performed after every two treatment cycles (weeks 6, 12, and 18). At the first radiological assessment (week 6), the primary pulmonary lesion decreased in size from 6.8×4.8 cm to 2.2×1.7 cm, fulfilling the criteria for partial response (PR). However, the residual lesion remained radiographically stable during subsequent follow-up and, together with normalization of serum ProGRP levels, was retrospectively interpreted as post-treatment fibrotic change, leading to reclassification as a complete response (CR). This CR status was maintained through the final assessment on September 16, 2025. Contrast-enhanced brain MRI, evaluated according to the RANO-BM criteria, was performed at 3, 9, 12, and 20 months after treatment initiation. At the initial intracranial assessment (3 months), the brainstem lesion (1.3×0.6 cm) had completely resolved, consistent with CR, which was sustained through the last follow-up evaluation on September 15, 2025 (**Supplementary Figure 6**). During 20 months of follow-up, ProGRP levels remained within the normal range, and NED status was consistently maintained, with imaging demonstrating only stable fibrotic residues and post-treatment changes (**Figures 1B, D, F**; **Supplementary Figures 7**, **8**).

Patient-reported outcomes were assessed using the European Organization for Research and Treatment of Cancer Quality of Life Questionnaire-Core 30 (EORTC QLQ-C30) at baseline, before each treatment cycle, and throughout follow-up. At baseline, the patient’s global health status score was 58/100. Following the first treatment cycle, the patient reported marked improvement in dizziness and cough, accompanied by an increase in the global health status score to 67. By the third cycle, the score had further improved to 78. These quality-of-life benefits were sustained throughout the 20-month follow-up.

Adverse events were graded according to the Common Terminology Criteria for Adverse Events (CTCAE) version 5.0. The only treatment-related adverse event observed was Grade 1 hypothyroidism, which developed after the third treatment cycle and was successfully managed with oral levothyroxine (25 μg daily) without interruption or dose reduction of immunotherapy. No Grade 2 or higher adverse events, including pneumonitis, colitis, hepatitis, dermatitis, or infusion-related reactions, were documented. Routine laboratory monitoring demonstrated no clinically significant cytopenia, hepatotoxicity, or renal dysfunction. The patient did not report any probiotic-related gastrointestinal adverse effects, such as bloating or diarrhea. Importantly, throughout treatment and follow-up, the patient developed no neurological symptoms, including headache, nausea or vomiting, focal neurological deficits, ataxia, or cranial nerve dysfunction. Serial neuroimaging demonstrated no evidence of radiation necrosis or clinically significant cerebral edema. No neurological event occurred that required corticosteroid therapy. At the most recent follow-up, neurological examination findings remained normal. Although no severe neurological toxicity or high-grade immune-related adverse events were observed, continued long-term surveillance remains necessary to evaluate the potential risk of delayed toxicities.

The patient reported that, before treatment initiation, persistent dizziness and refractory cough had severely impaired his quality of life. After being fully informed of the extremely poor prognosis associated with brainstem metastasis from extensive-stage small cell lung cancer and the limited expected benefit of standard chemotherapy, he elected to decline conventional treatment. Following an extensive shared decision-making process, the patient proactively requested treatment with the TMEp-CI-M platform. After therapy initiation, he described a substantial recovery, characterized by progressive resolution of dizziness and cough. He particularly emphasized that the absence of significant treatment-related toxicities, such as nausea, severe fatigue, or alopecia, enabled him to maintain normal social functioning and psychological well-being. The patient provided explicit consent for the sharing of his treatment experience.

## 4 Discussion

This case report demonstrates the marked efficacy of the sequential TMEp-CI-M platform in a patient with ES-SCLC possessing multiple poor prognostic features. The depth and durability of the response indicate the potent, systemic immune reaction, a particularly significant outcome given the poor prognosis of ES-SCLC and the limited efficacy of standard immunotherapy. These findings also provide clinically relevant references for brainstem radiotherapy in SCLC and offer insights into the potential synergistic effects of SBRT combined with immunotherapy for intracranial oligometastatic disease. The patient expressed gratitude for this innovative therapeutic strategy, which achieved outcomes far exceeding expectations, and provided consent to share this case to help inform and benefit others.

In this case, modulation of the TME was initiated using SBRT (8 Gy×3 fractions), low-dose etoposide, and anlotinib. Previous studies have demonstrated that SBRT delivered at 8 Gy×3 fractions represents an optimal fractionation scheme for inducing abscopal effects and synergizing with immunotherapy (11, 12). The selection of low-dose etoposide was driven by the patient’s refusal of standard chemotherapy, together with evidence indicating that low-dose chemotherapy can favorably remodel the immune microenvironment through multiple immunomodulatory mechanisms, including the enhancement of ICD, DC activation, and improvement of the tumor immune microenvironment (TIME). In contrast, conventional chemotherapy has been shown to increase regulatory T cells (Tregs) and M2-type tumor-associated macrophages (TAMs), suppress antitumor immune responses, and markedly disrupt the diversity and composition of the gut microbiota (13).

Anti-angiogenic therapy is well established to enhance the efficacy of ICIs across multiple malignancies (14). Anlotinib inhibits key signaling pathways such as VEGF, promotes tumor vascular “normalization, ” improves intratumoral oxygenation, and significantly enhances infiltration of cytotoxic immune cells, including CD8^+^ T cells, into the tumor core. Moreover, anlotinib downregulates PD-L1 expression on tumor-associated vascular endothelial cells and certain tumor cells, while reducing the abundance of immunosuppressive M2-type TAMs and myeloid-derived suppressor cells (MDSCs) within the TME, thereby synergizing with immunotherapy (15, 16). The ETER701 study further validated the clinical value of combining anlotinib with immunotherapy in the first-line treatment of ES-SCLC. Although the addition of anlotinib to chemotherapy alone did not yield a significant survival benefit, the combination of anlotinib and benmelstobart (a PD-L1 inhibitor) with chemotherapy extended median overall survival from 11.9 months to 19.3 months, substantially surpassing outcomes historically achieved with PD-L1 inhibitors alone (17).

The role of CTLA-4 inhibitors in ES-SCLC remains under active investigation. The phase III trial NCT01450761, which assessed the addition of ipilimumab to first-line etoposide-platinum chemotherapy, failed to demonstrate a significant improvement in overall survival (median OS: 11.0 months vs. 10.9 months; hazard ratio 0.94; P = 0.3775), indicating that CTLA-4 blockade combined with chemotherapy alone did not confer a survival advantage (18). Similarly, in the CASPIAN phase III trial, the addition of durvalumab and tremelimumab to chemotherapy did not improve survival outcomes (19). However, subsequent exploratory analyses of the CASPIAN study suggested that CTLA-4 inhibitors may provide a survival benefit in specific immunogenic contexts, including tumors with high antigen presentation machinery (APM) expression (median OS 25.9 vs. 14.6 months), elevated MHC class I expression, and increased CD4, FOXP3, and CTLA-4 gene expression (20). These findings imply that a favorable TIME may unleash the therapeutic potential of CTLA-4 blockade. Cadonilimab, the world’s first PD-1/CTLA-4 bispecific antibody, incorporates an innovative tetravalent bispecific architecture and an Fc-null modification, contributing to its favorable safety profile. It has demonstrated notable antitumor activity across multiple advanced solid tumors (21). Compared with landmark clinical trials in ES-SCLC, this case demonstrates several distinctive features. In the IMpower133 and CASPIAN trials, the addition of PD-L1 inhibitors resulted in median overall survival improvements of approximately 2–3 months, whereas patients with BM were either excluded or constituted only a small subgroup. In the ETER701 study, the addition of anlotinib to chemotherapy plus benmelstobart prolonged median overall survival to 19.3 months; however, this outcome still did not approach the durable NED status achieved in our patient. This case is particularly notable for three reasons: (i) the presence of a brainstem metastasis, (ii) a PD-L1 combined positive score (CPS) of 0, and (iii) the omission of platinum-based chemotherapy. We hypothesize that the TMEP phase, consisting of SBRT, low-dose etoposide, and anlotinib, may have transformed an immunologically “cold” TME into a more inflamed, “hot” phenotype, thereby sensitizing the tumor to subsequent PD-1/CTLA-4 bispecific antibody therapy and enabling a deep and durable response. This hypothesis is supported by biomarker analyses from the CASPIAN trial, which suggested that patients with high expression of APM-related genes derive greater benefit from CTLA-4 blockade. Notably, the individual components of the TMEP regimen have each been reported to enhance major histocompatibility complex class I (MHC-I) expression and upregulate APM-associated pathways. Accordingly, this case provides preliminary clinical evidence that a sequential, multi-modal TME reprogramming strategy may have the potential to overcome immune checkpoint inhibitor resistance in PD-L1-negative SCLC. Nevertheless, this mechanistic hypothesis remains exploratory and warrants validation in prospective biomarker-driven clinical studies.

*Bifidobacterium Triple Viable Tablet* (containing *B. longum*, *L. bulgaricus*, and *S. thermophilus*), as a microecological modulator, exhibits substantial theoretical potential and promising application prospects in synergistic tumor immunotherapy. Their central mechanism is rooted in the profound influence of the gut microbiota on host immune regulation. Notably, *Bifidobacterium longum* has been demonstrated in multiple high-impact studies to reshape the host microenvironment toward an antitumor immune state through diverse mechanisms, including enhancement of DC function and antigen presentation, augmentation of CD8^+^ T-cell cytotoxicity, modulation of T-cell subset balance, and generation of beneficial immunomodulatory metabolites. Accordingly, this formulation may exert potent synergistic effects when combined with ICIs and other immunotherapies (22–25). However, it should be emphasized that current evidence is largely derived from strong theoretical frameworks and extensive preclinical studies, and high-quality clinical investigations specifically evaluating this compound formulation remain lacking.

In the management of BM, SBRT or stereotactic radiosurgery (SRS) offers several advantages over whole-brain radiotherapy (WBRT) for patients with oligometastatic disease, particularly when combined with ICIs. SBRT/SRS delivers highly precise, ablative radiation doses that effectively induce ICD, promote tumor antigen release, and activate immune signaling pathways such as the cGAS-STING axis and type I interferon responses, thereby enhancing both local and systemic antitumor immunity (26–28). In addition, SBRT/SRS increases infiltration of effector T cells and upregulates chemokines such as CXCL9 and CXCL10 within the TME, fostering an immunologically “hot” milieu conducive to ICI efficacy (29). In contrast, WBRT, while effective for widespread intracranial disease, relies on low-dose fractionated regimens that frequently induce systemic lymphopenia, potentially impair host immune competence, and contribute to neurocognitive decline (30). Therefore, in patients with limited BM, SBRT/SRS may represent not only a more precise local treatment modality but also a more favorable platform for synergistic immuno-radiotherapy.

The present case of SCLC adds to a growing body of hypothesis-generating evidence and represents the sixth tumor type in which the TMEp-CI-M platform has demonstrated encouraging clinical activity. This observation may provide preliminary insight into the potential adaptability of the platform across diverse tumor biologies. The platform is not a rigid fixed solution but a modular framework. The TMEp components can be customized based on tumor biology; the checkpoint inhibitor can be selected according to major resistance mechanisms; and the microbiome component can be optimized as more specific, synergistic combinations of microbiota are identified. The consistent theme is the strategic sequence: a powerful, SBRT-based multi-mechanistic initial strike to “unlock” the TME and unleash a cascade of heterogeneous tumor antigens, followed by immune activation. Accumulating evidence suggests that probiotics may synergize with cancer immunotherapy and help alleviate treatment-related adverse effects. This sequence is crucial for converting the immune environment from “non-reactive” to “activated, ” thereby bypassing traditional resistance mechanisms, such as PD-L1 negativity.

This case further supports the potential of the TMEp-CI-M platform to achieve durable remission in refractory SCLC with brainstem metastasis. Nevertheless, several important limitations should be acknowledged. First, as a single-case report lacking a control arm, causality cannot be definitively established, and the observed outcome may have been influenced by selection bias or the intrinsic biological behavior of the disease. Second, the multicomponent TMEp regimen, comprising SBRT, low-dose etoposide, and anlotinib, was specifically designed to induce synergistic remodeling of the TME. However, because these therapeutic modalities were administered sequentially and in combination, the relative contribution of each component cannot be delineated from this single observation. Third, all published evidence supporting the TMEp-CI-M platform is derived from the same research group, and independent validation is lacking. Therefore, conclusions regarding its broad applicability across tumor types should be drawn with caution. Fourth, although probiotic supplementation with Bifidobacterium triple viable capsules is supported by a plausible mechanistic rationale, current evidence remains largely derived from preclinical studies or indirect clinical inference, with a lack of robust prospective clinical validation. From a safety perspective, only Grade 1 hypothyroidism was observed in the present case. Nonetheless, the combined regimen—including immunotherapy, radiotherapy, antiangiogenic therapy, and chemotherapy—carries the potential for overlapping toxicities, such as immune-related pneumonitis, hemorrhagic complications, and gastrointestinal perforation. In particular, among patients with brainstem metastases, post-SBRT edema or radiation necrosis warrants close surveillance because of the critical anatomical location and the potential for severe neurological sequelae. Therefore, although the TMEp-CI-M platform appeared well tolerated in this patient, systematic evaluation of its safety profile in larger patient cohorts is necessary.

## 5 Conclusion

The TMEp-CI-M platform achieved durable clinical complete remission in a patient with PD-L1-negative ES-SCLC and brainstem metastasis. This case reinforces its role as a versatile, sequential strategy capable of reprogramming the TME to induce effective anti-tumor immunity in resistant “cold” tumors. Although accumulating observations across several tumor types are encouraging, the current evidence remains predominantly hypothesis-generating. Formal prospective assessments of this platform are warranted to further investigate its clinical utility.

## Data Availability

The original contributions presented in the study are included in the article/**Supplementary Material**. Further inquiries can be directed to the corresponding authors.

## Supplementary material

Supplementary Table 1 IHC pathology report.

Supplementary Figure 1 Baseline contrast-enhanced CT of the pulmonary lesion (December 15, 2023).

Supplementary Figure 2 Baseline contrast-enhanced MRI of the brainstem lesion (December 14, 2023).

Supplementary Figure 3 IHC Pathology images.

Supplementary Figure 4 GTV delineation of pulmonary lesion (The GTV is delineated by the red line).

Supplementary Figure 5 GTV delineation of brainstem lesion (The GTV is delineated by the purple line).

Supplementary Figure 6 Serial contrast-enhanced chest CT and brain MRI, assessed according to RECIST version 1.1 and RANO-BM criteria, respectively, demonstrated sustained complete remission. At baseline, the extracranial target lesion consisted of a left hilar mass measuring 6.8×4.8 cm, while the intracranial target lesion was a brainstem metastasis measuring 1.3×0.6 cm. Following two treatment cycles (week 6), the extracranial lesion achieved a partial response (PR), subsequently regressing to a stable scar-like residual lesion accompanied by normalization of serum ProGRP levels; this finding was retrospectively reclassified as a complete response (CR). Sustained extracranial CR was maintained from cycles 4–6 through the end of treatment. Intracranial CR was achieved at the 3-month evaluation and persisted through the final follow-up assessment at 20 months.

Supplementary Figure 7 Contrast-enhanced CT of the pulmonary lesion (September 16, 2025).

Supplementary Figure 8 Contrast-enhanced MRI of the brainstem lesion (September 15, 2025).
