# Supplementary material for: Sustained complete response to TMEp-CI-M platform in refractory small-cell lung cancer with brainstem metastasis: a case report with over 20 months of disease-free survival
Source: Front Immunol. 2026 Jun 1;17:1807865. doi: 10.3389/fimmu.2026.1807865 (PMC13265516; doi:10.3389/fimmu.2026.1807865)
Supplement: Supplementary Table 1 — IHC pathology report. [file Table1.docx]

### ****Table S1. Immunohistochemical Pathology Report****

| **Category** | **Findings** |
| --- | --- |
| **Pathological Diagnosis** | Small cell carcinoma, left upper lung |
| **PD-L1 Status (CPS)** | 0 (Assay: Dako 22C3) |
| **Mismatch Repair Status** | pMMR  (expression of MLH1, MSH2, MSH6, PMS2) |
| **Proliferation Index** | Ki-67: 90% |
| **IHC-Positive Markers** | TTF-1 (++), CKP (+++), Synaptophysin (+++),  CD56 (+++), INSM-1 (+++),P53 (90%),  INI-1 (+++), BRG-1 (+++) |
| **IHC-Negative Markers** | NapsinA, P40, CK5/6, Vimentin,  ALK (D5F3), CerbB-2 (0), SSTR-2a |
| **Rb-1 Status** | Loss of expression |

**Abbreviations:** CPS, Combined Positive Score; pMMR, proficient mismatch repair; IHC, Immunohistochemistry
