# Supplementary figures and images for: Sustained complete response to TMEp-CI-M platform in refractory small-cell lung cancer with brainstem metastasis: a case report with over 20 months of disease-free survival
Source: Front Immunol. 2026 Jun 1;17:1807865. doi: 10.3389/fimmu.2026.1807865 (PMC13265516; doi:10.3389/fimmu.2026.1807865)

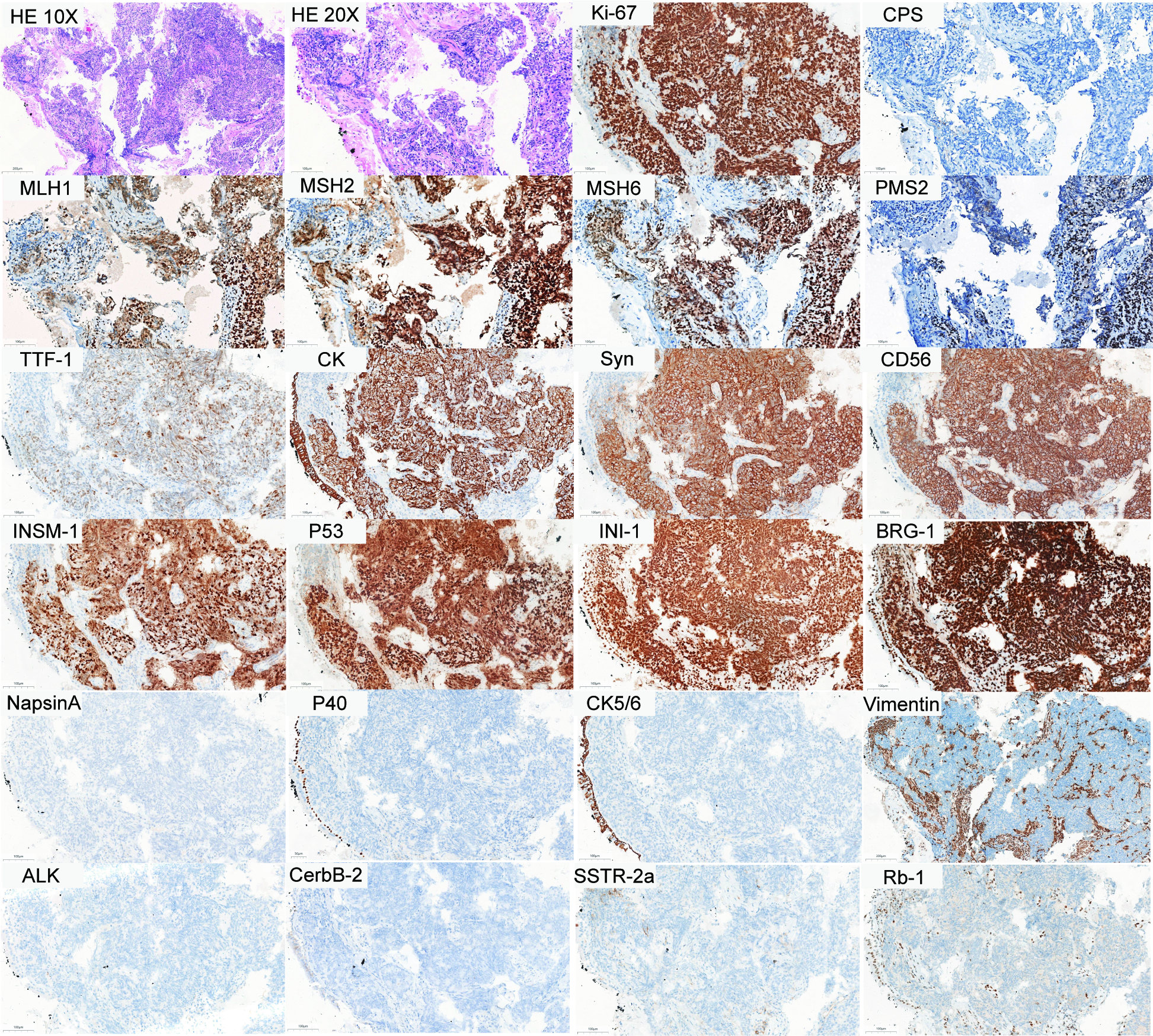

Supplement: Supplementary Figure 1 — Baseline contrast-enhanced CT of the pulmonary lesion (December 15, 2023). [file Image1.tif]

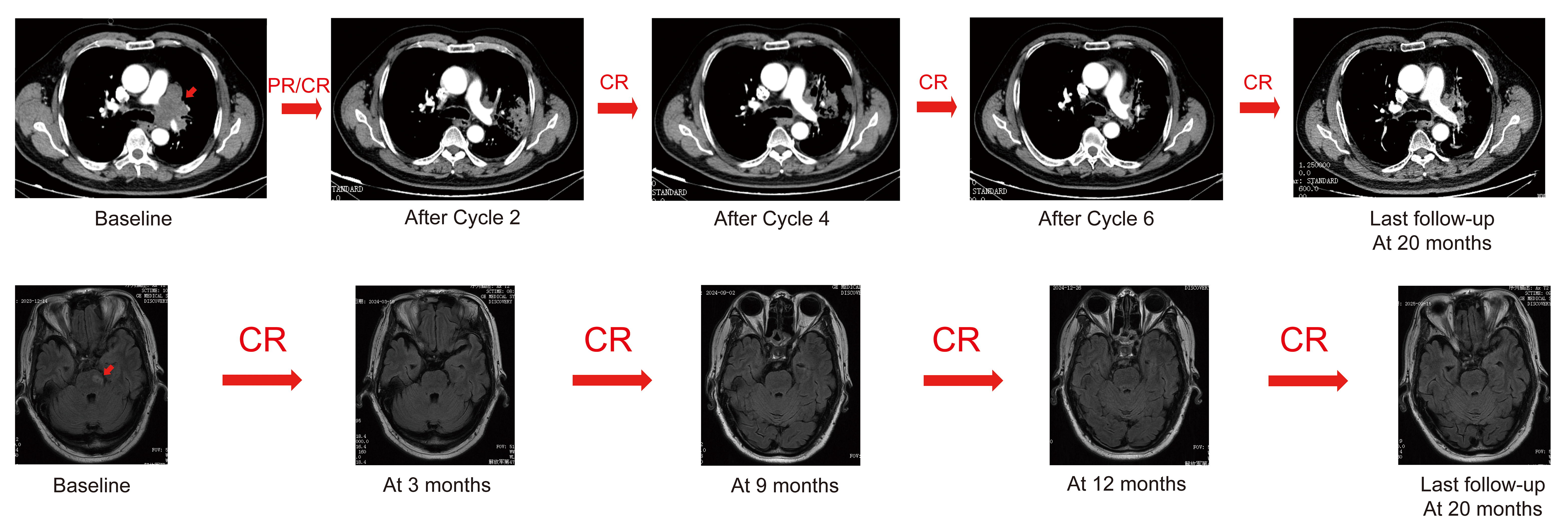

Supplement: Supplementary Figure 2 — Baseline contrast-enhanced MRI of the brainstem lesion (December 14, 2023). [file Image2.tif]
