# Supplementary material for: Sustained complete response to TMEp-CI-M platform in refractory small-cell lung cancer with brainstem metastasis: a case report with over 20 months of disease-free survival
Source: Front Immunol. 2026 Jun 1;17:1807865. doi: 10.3389/fimmu.2026.1807865 (PMC13265516; doi:10.3389/fimmu.2026.1807865)
Supplement: Supplementary Figure 3 — IHC Pathology images. [file DataSheet1.pdf]

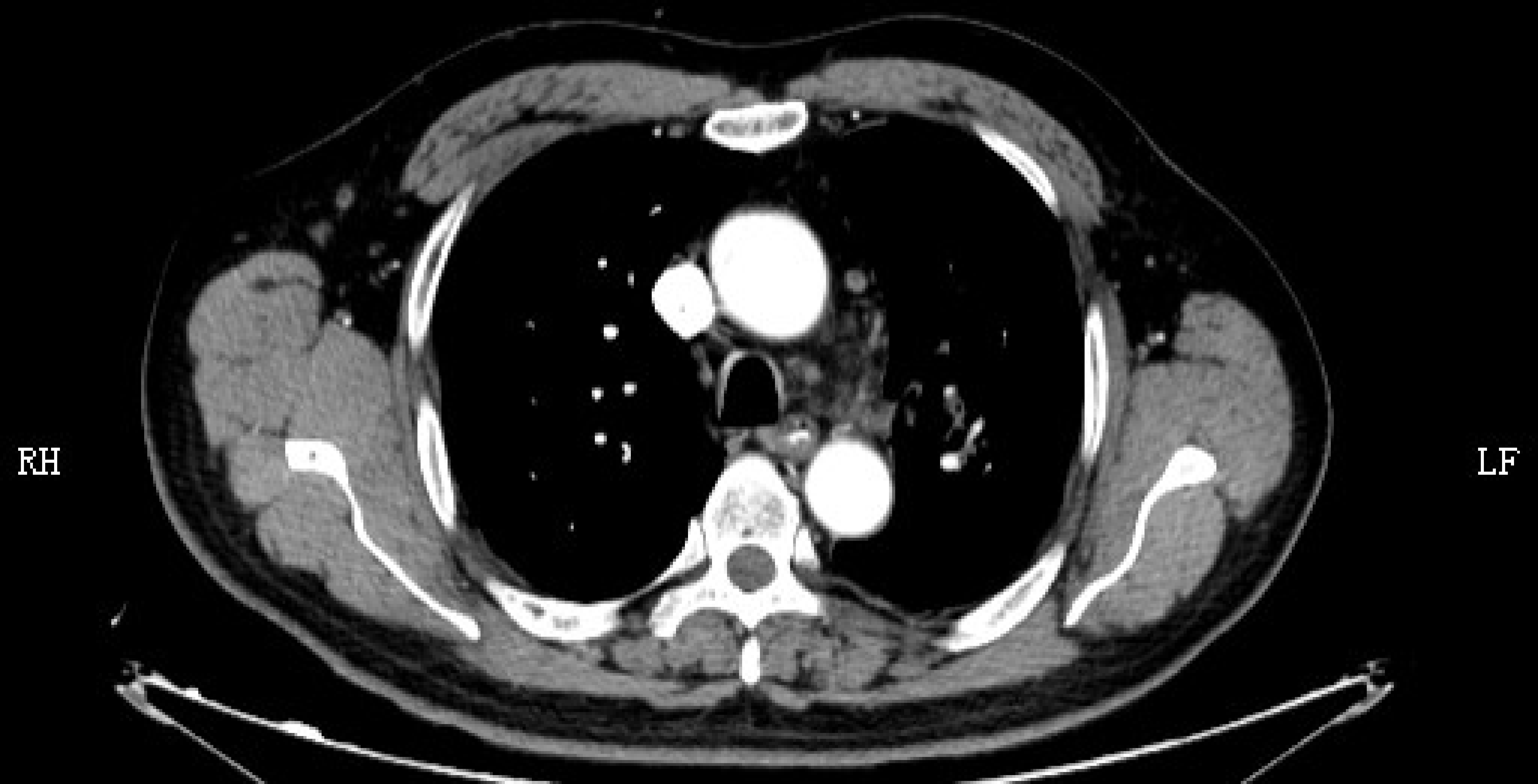
RH
LF

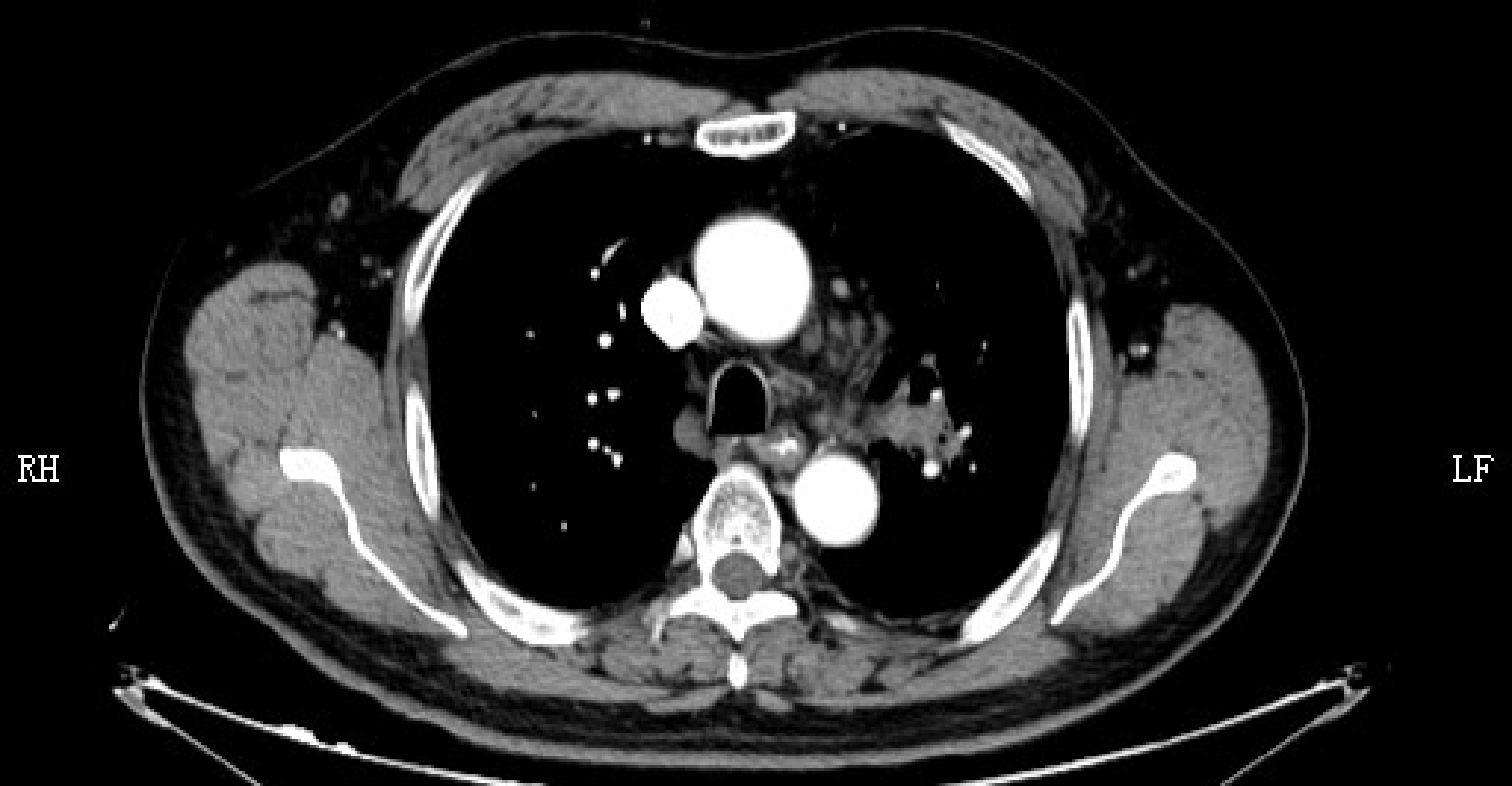
RH
LF

RH 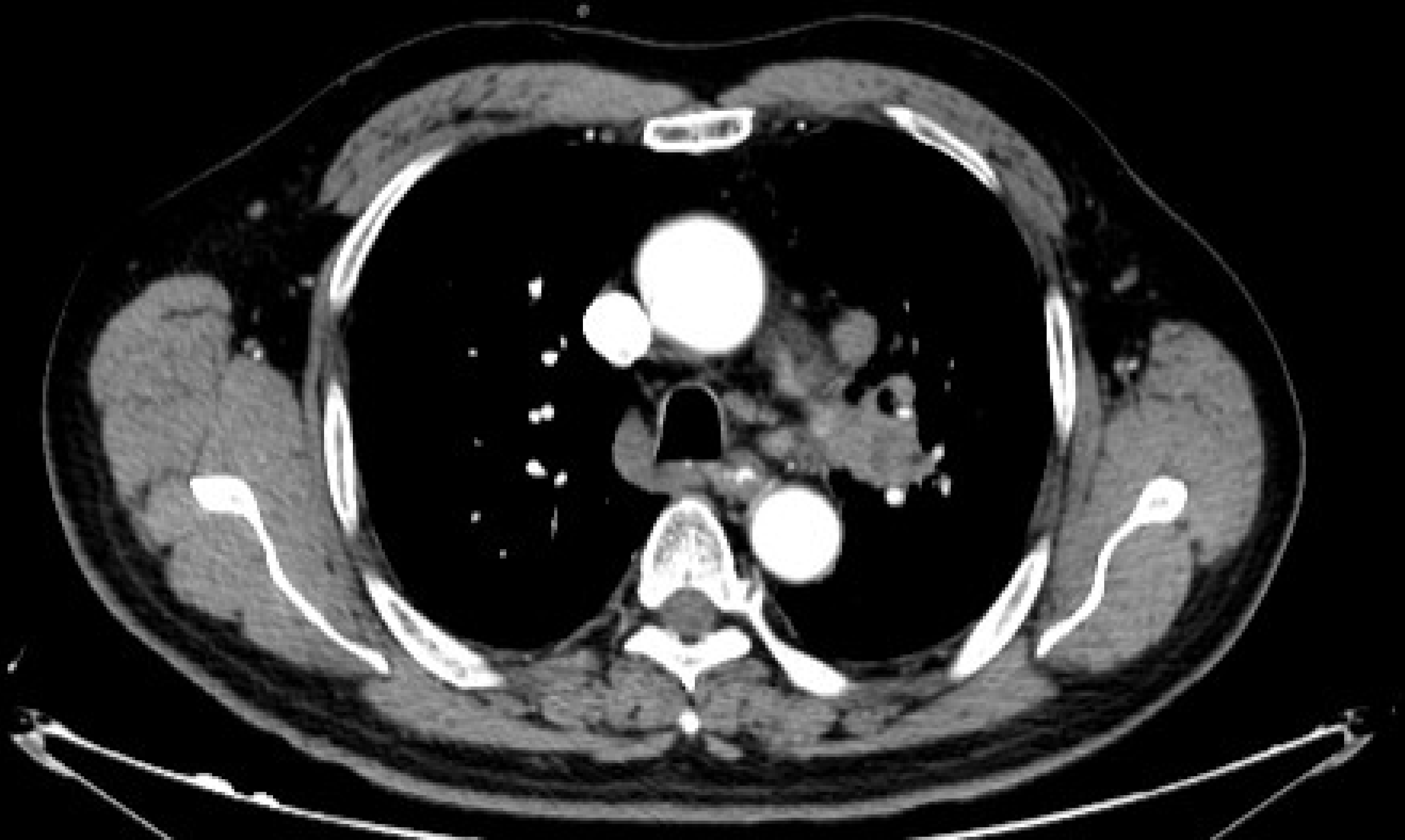 LF

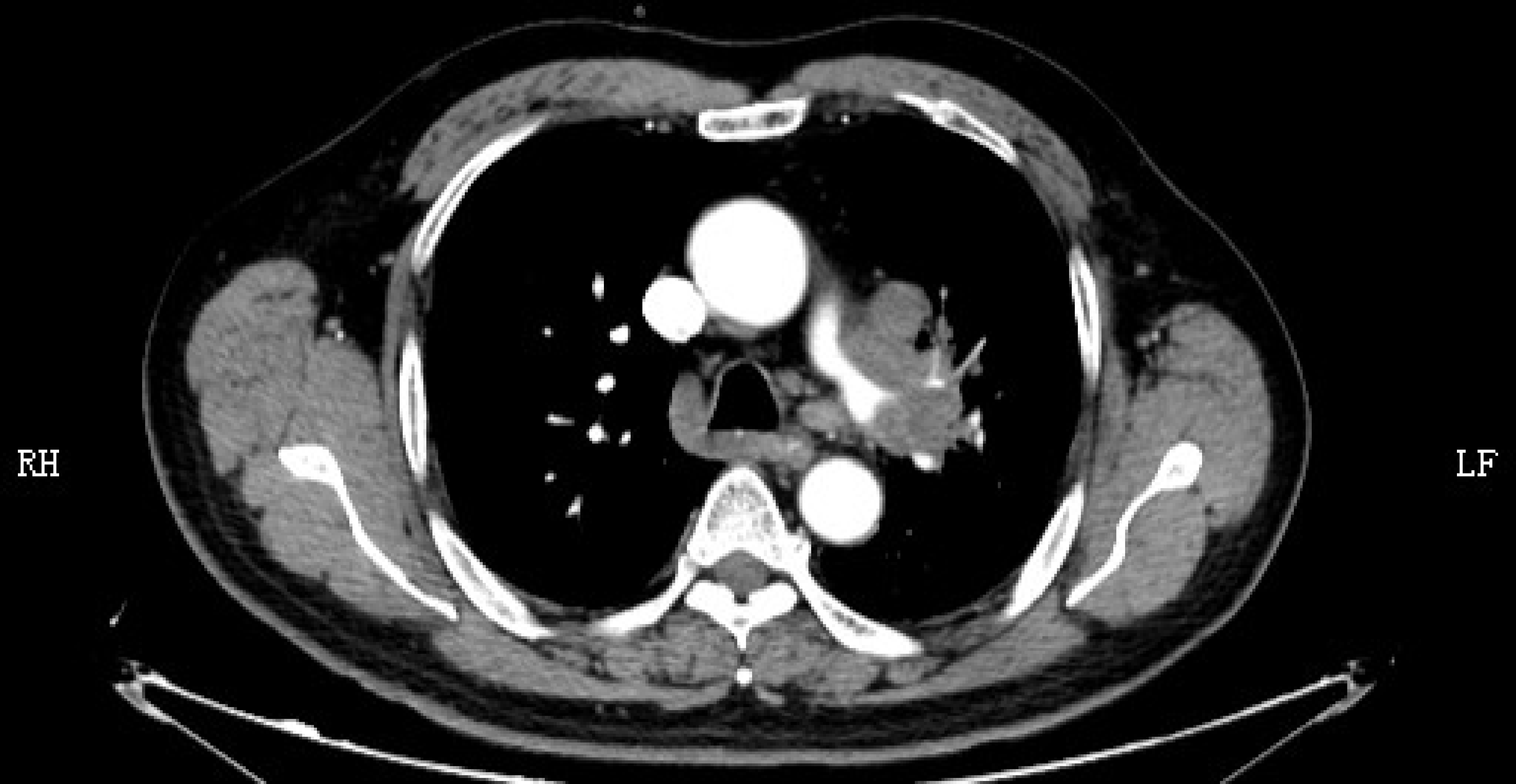
RH
LF

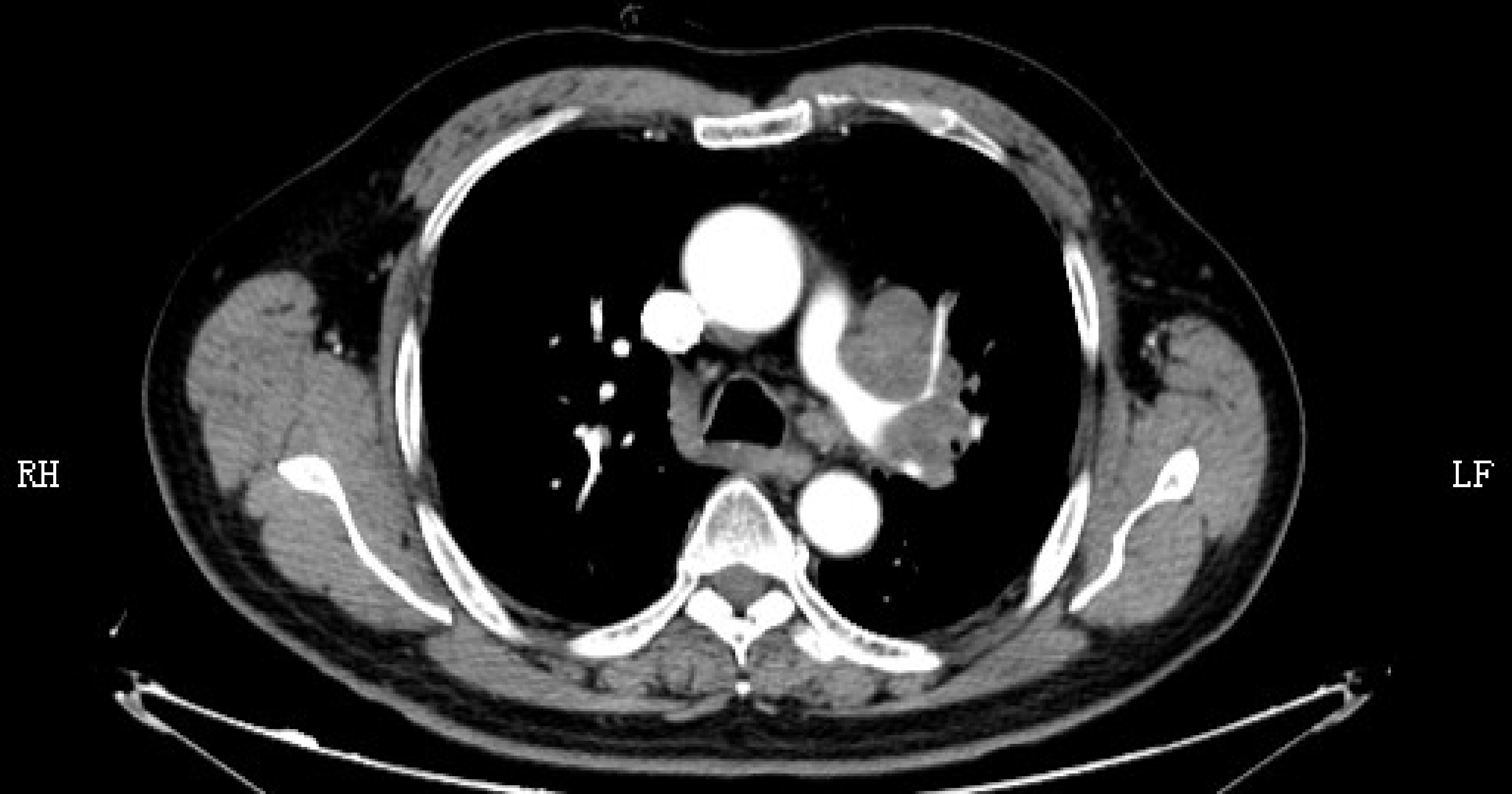
RH
LF

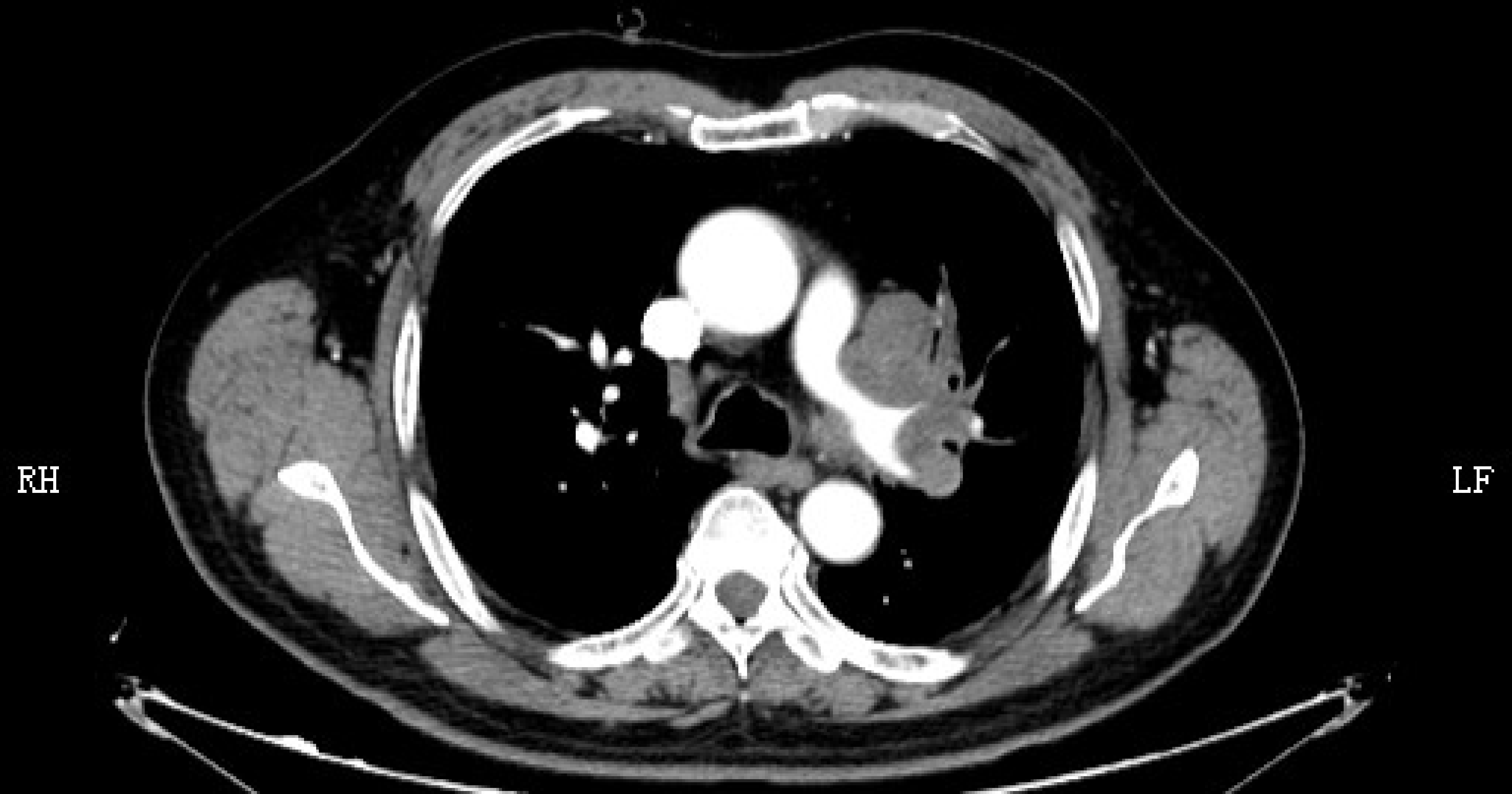

RH
LF

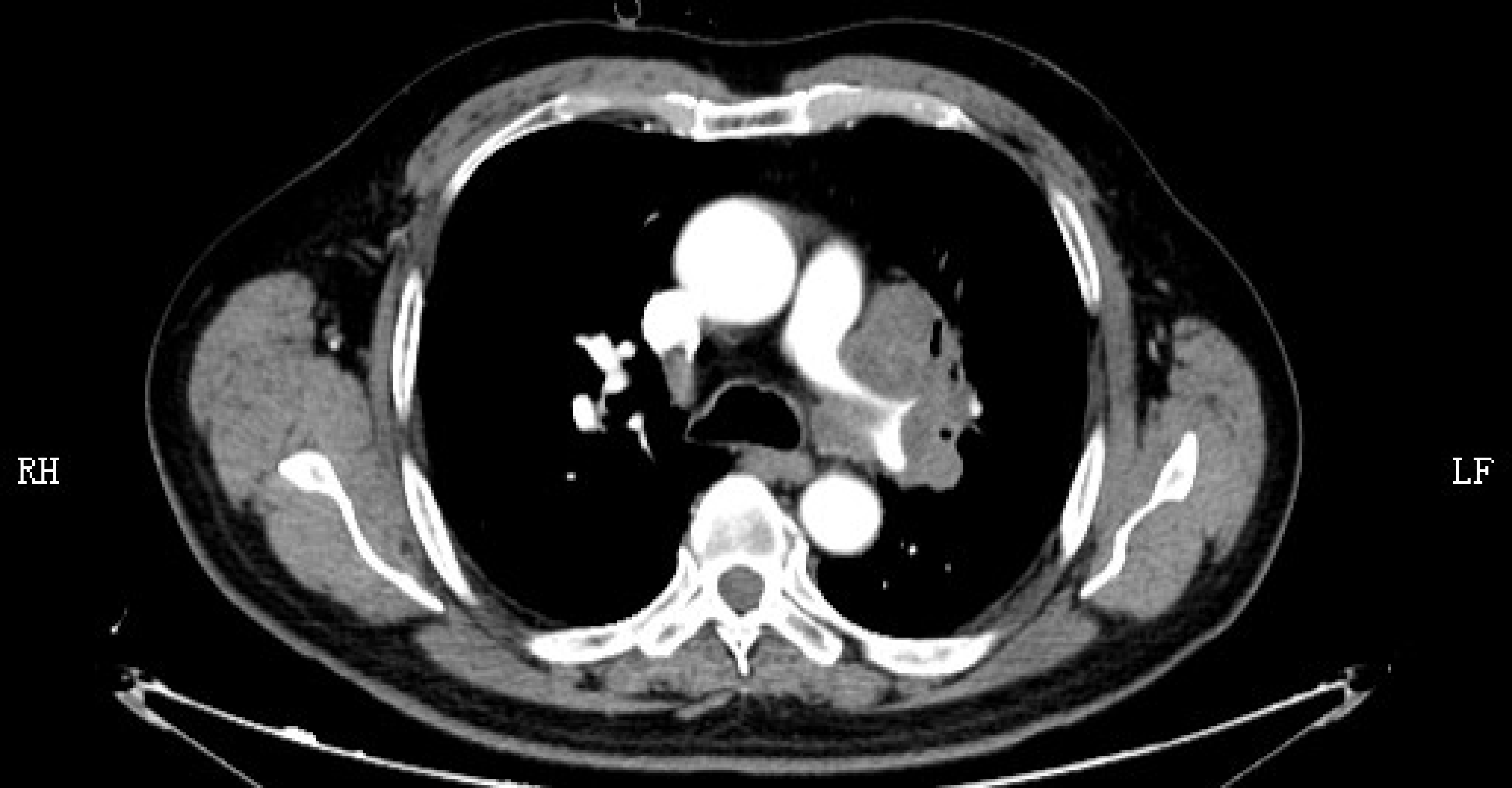
RH
LF

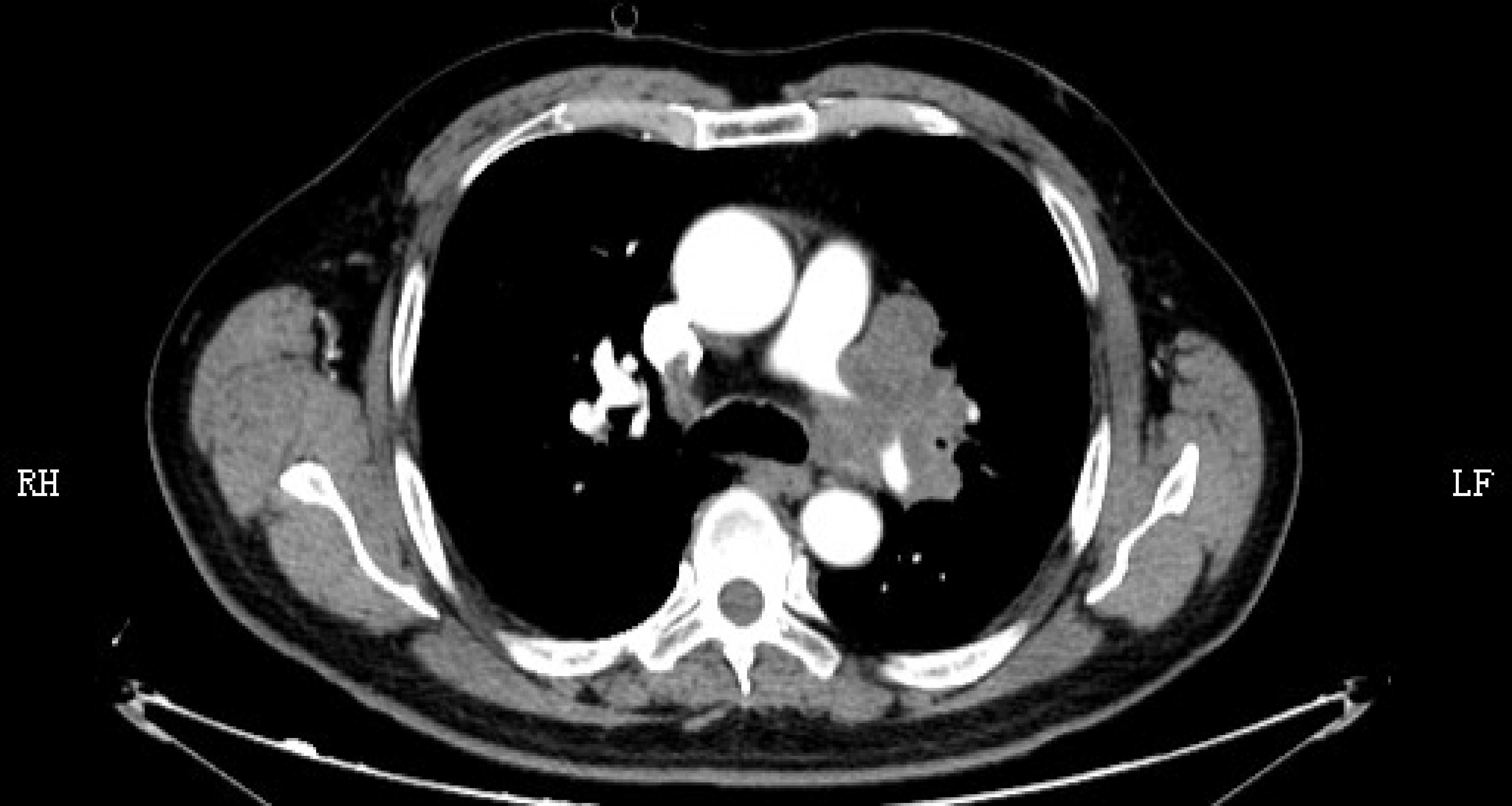
RH
LF

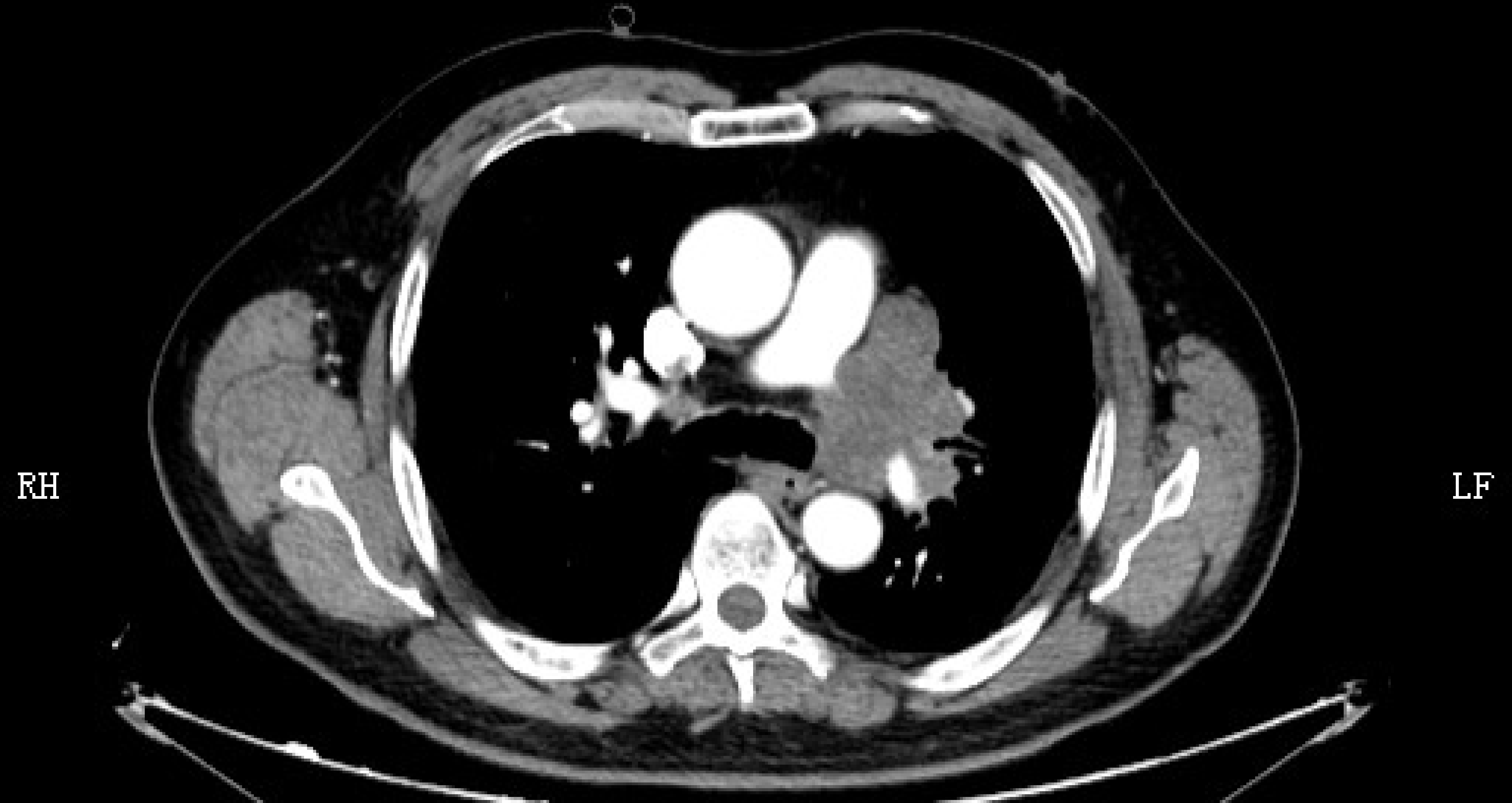
RH
LF

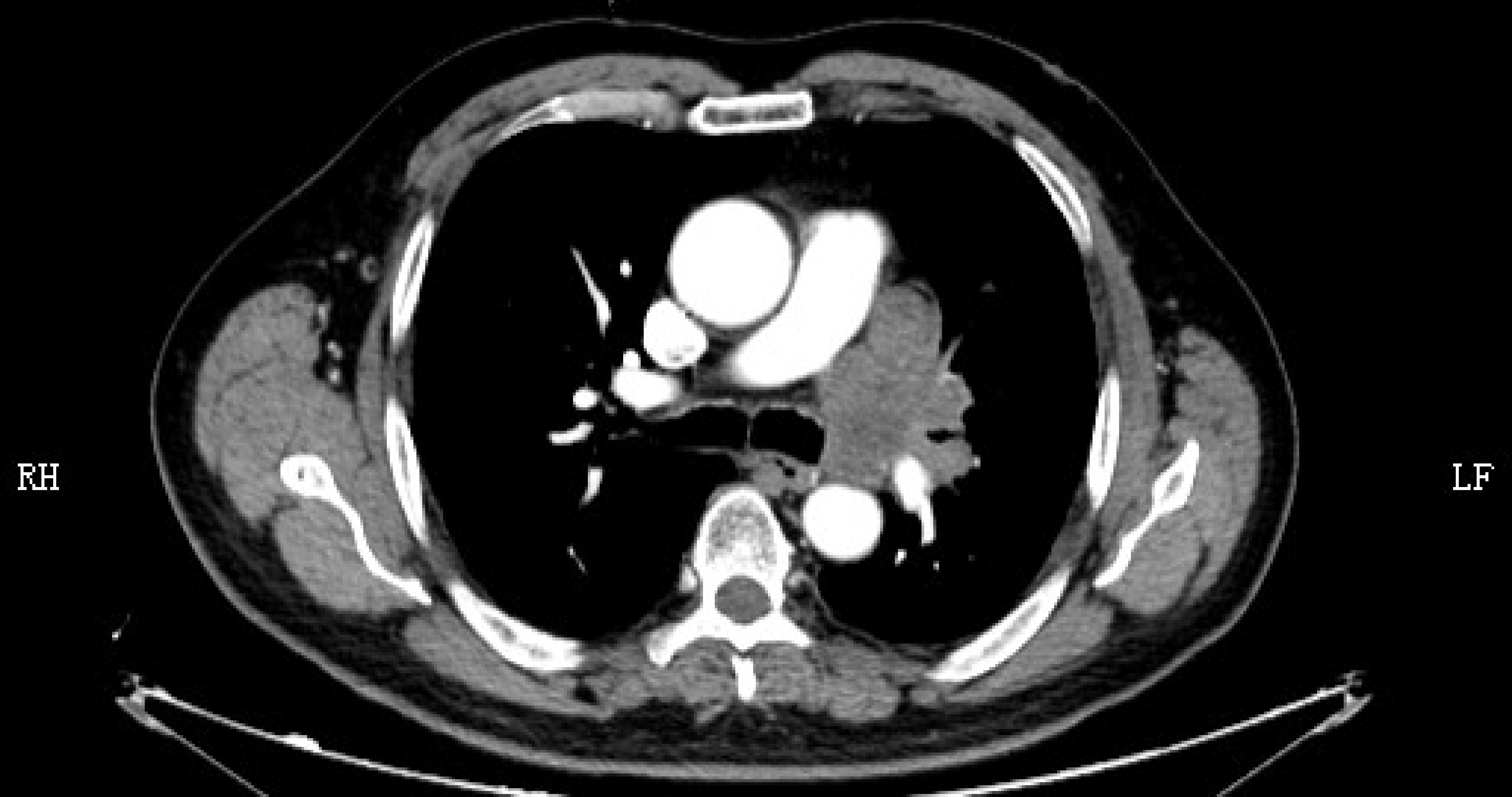
RH
LF

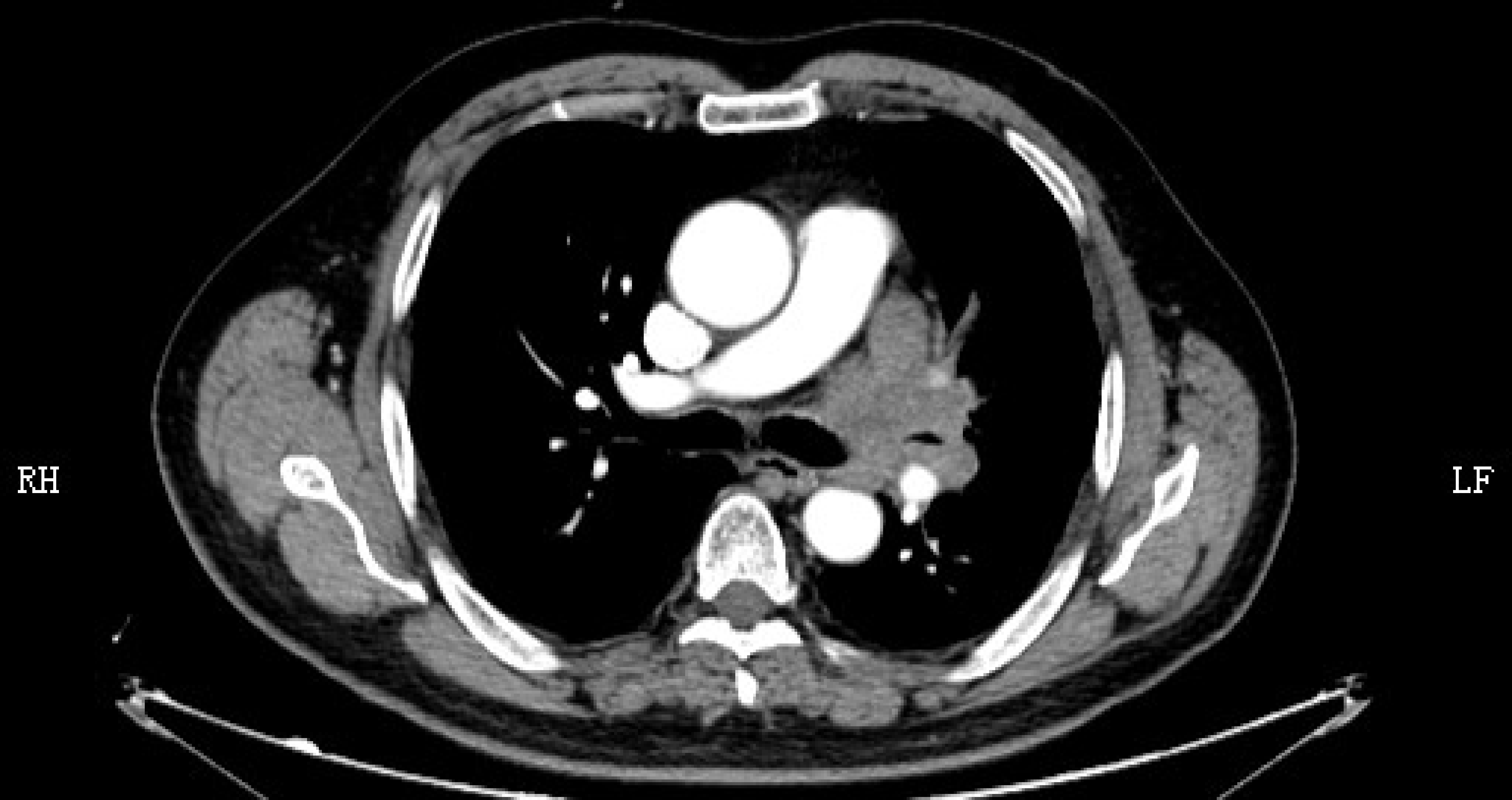
RH
LF

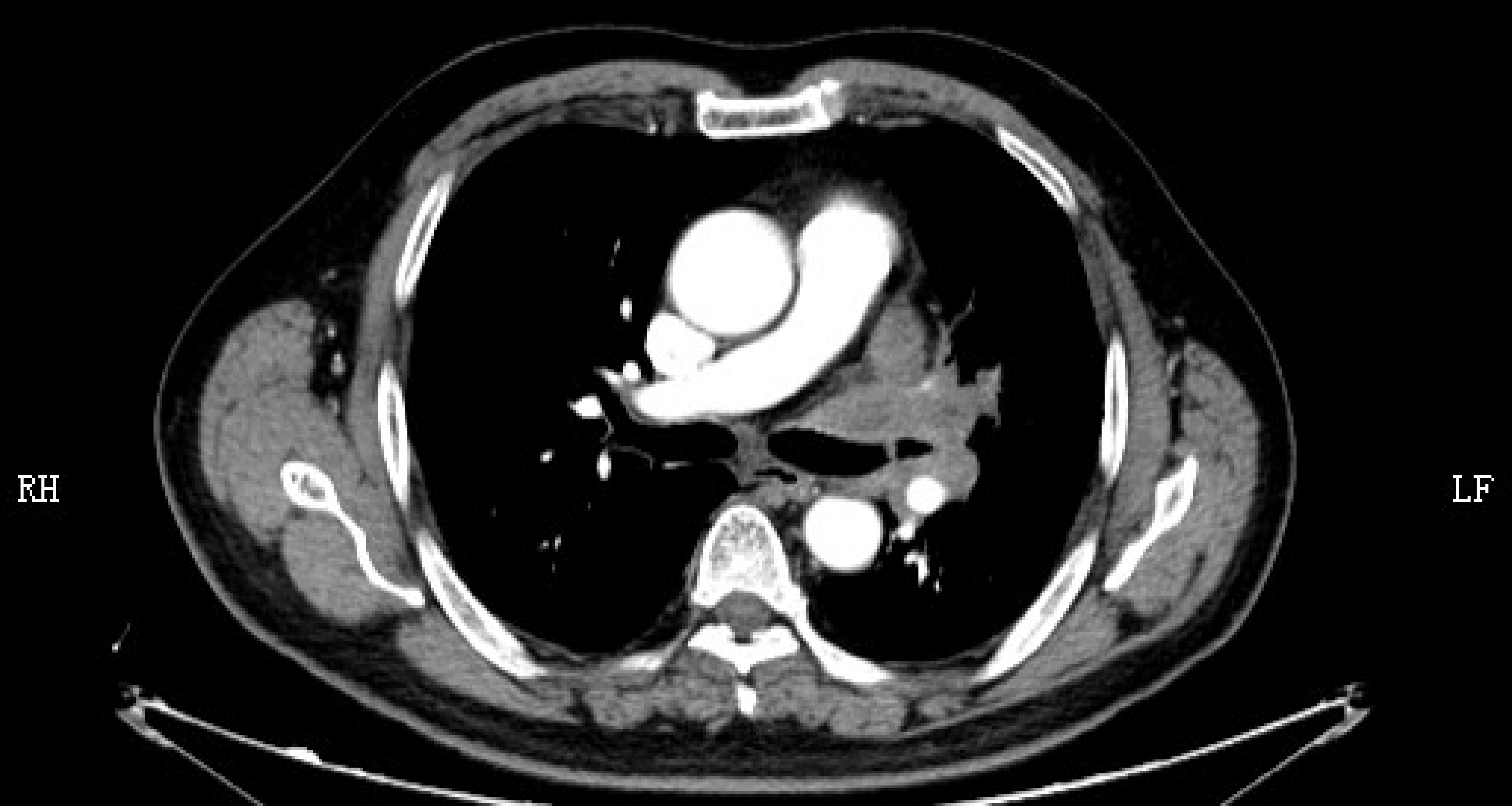
RH
LF

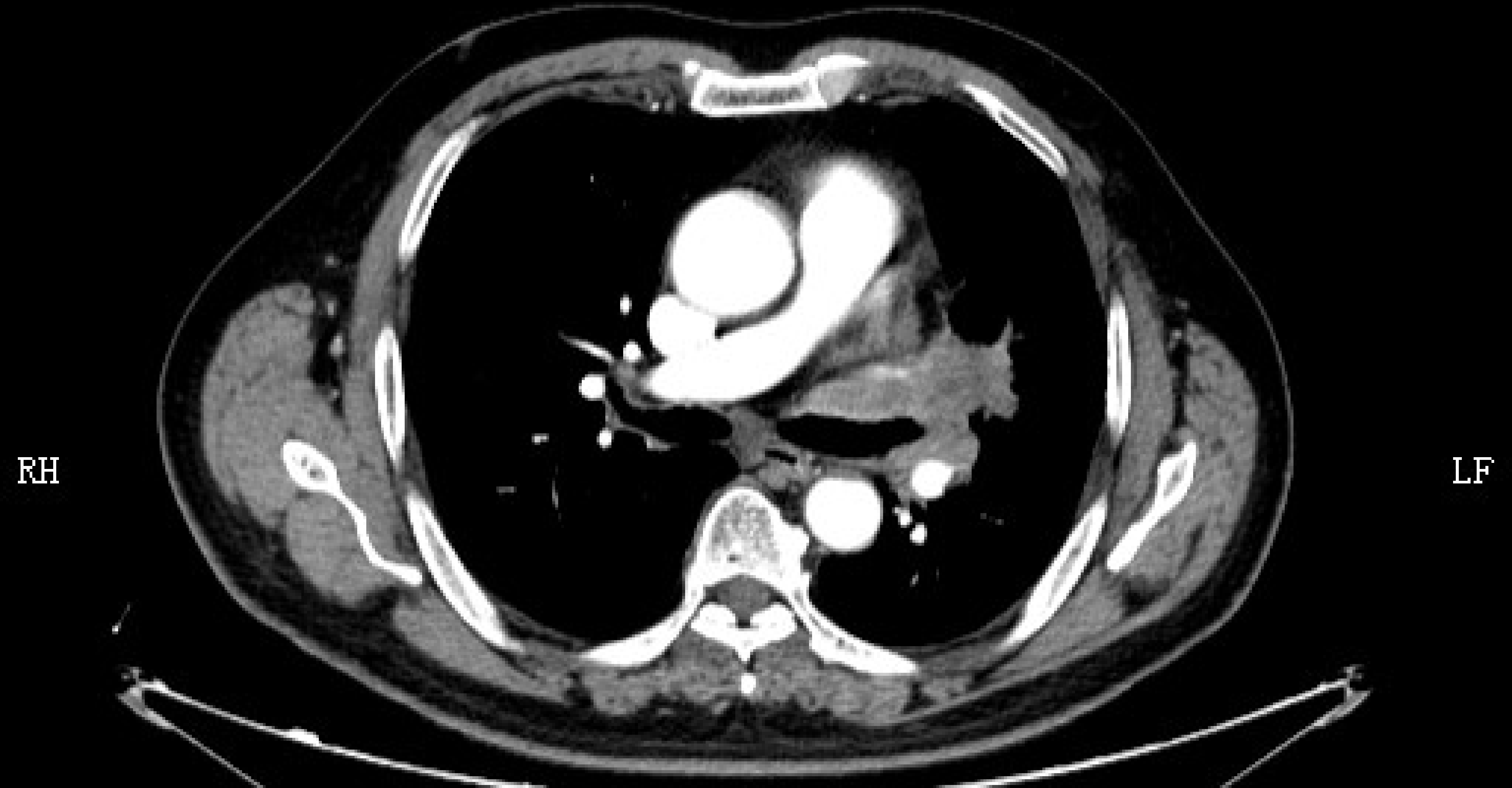
RH
LF

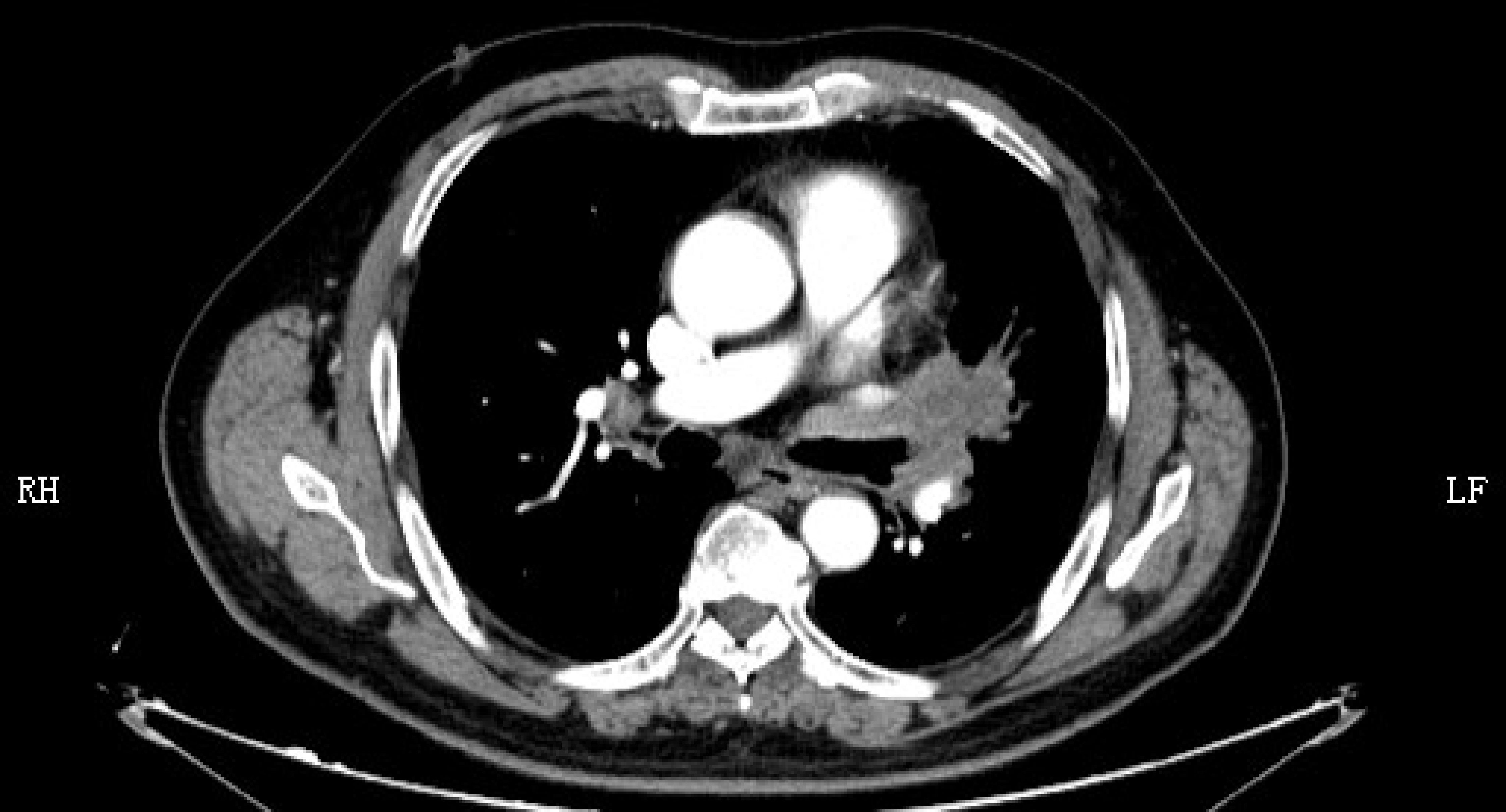
RH
LF

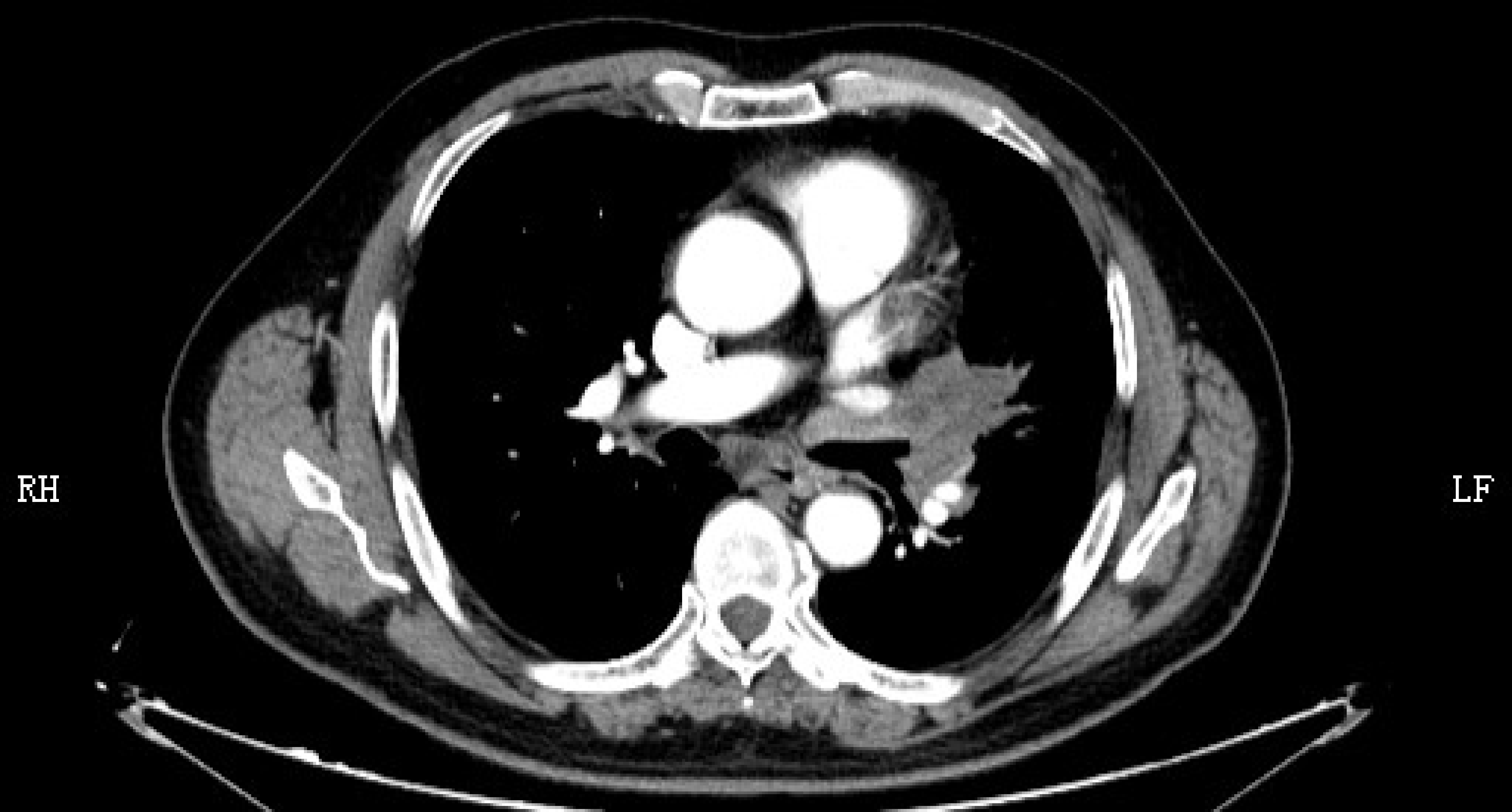
RH
LF

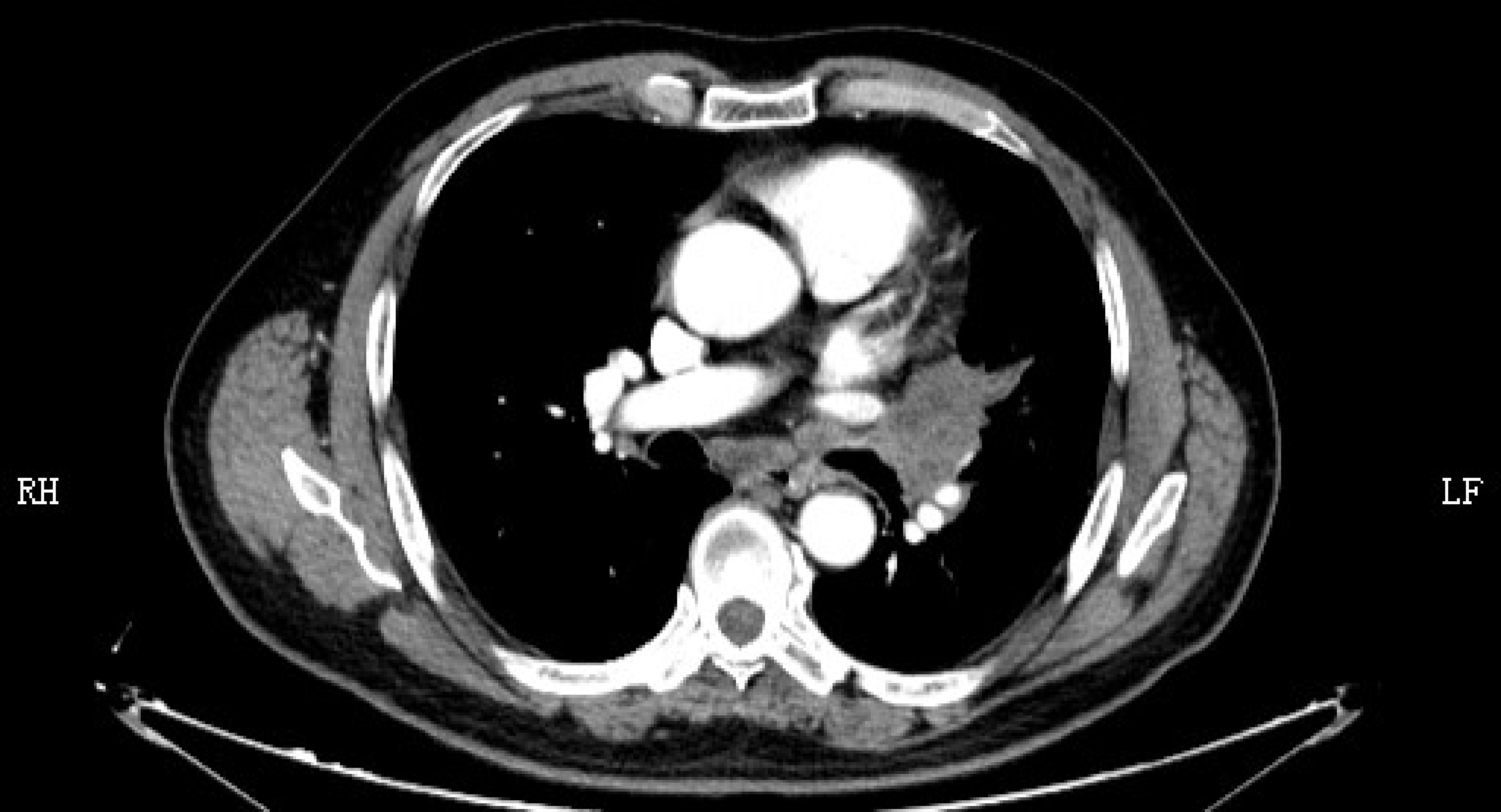
RH
LF

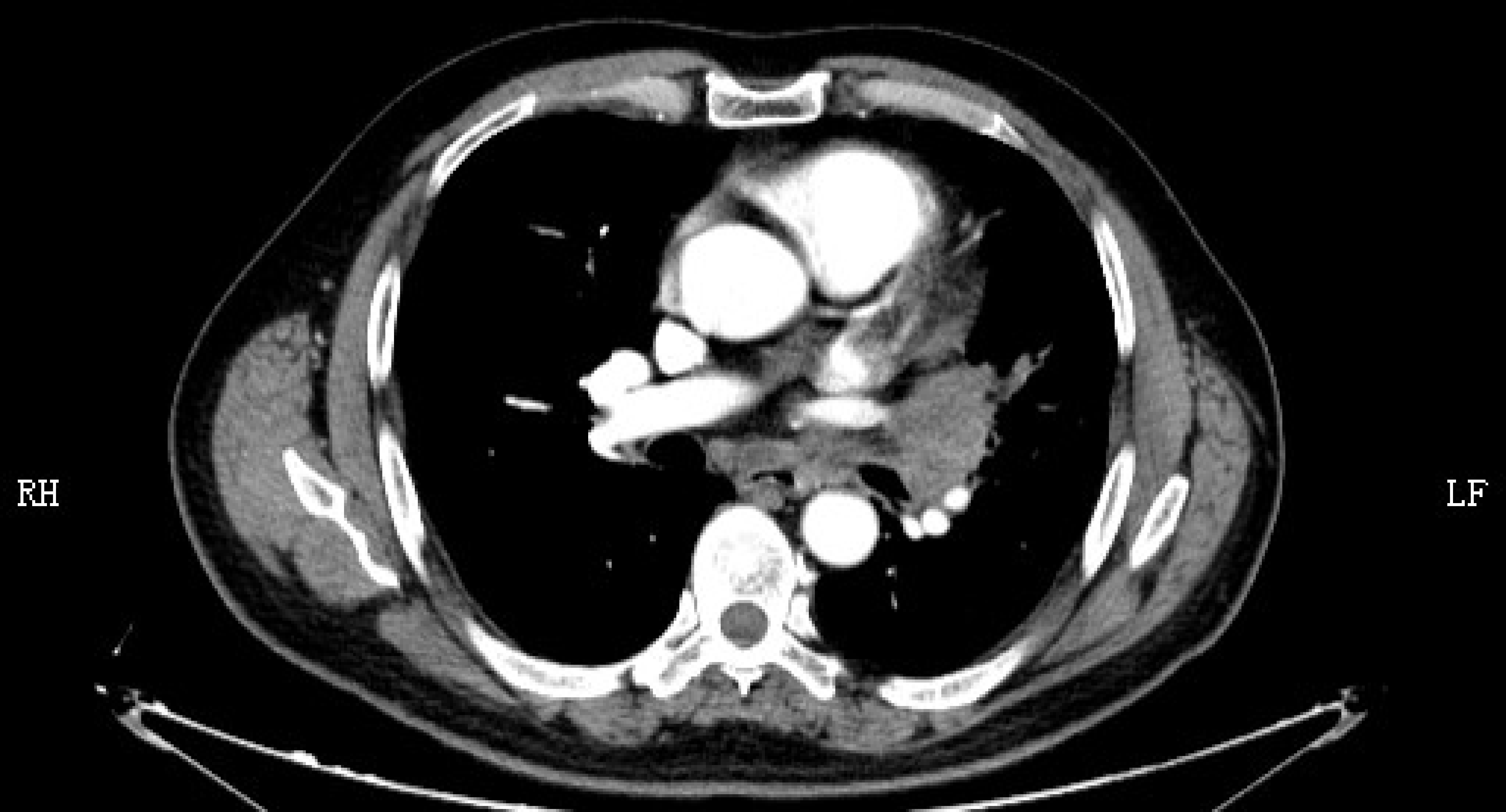
RH
LF

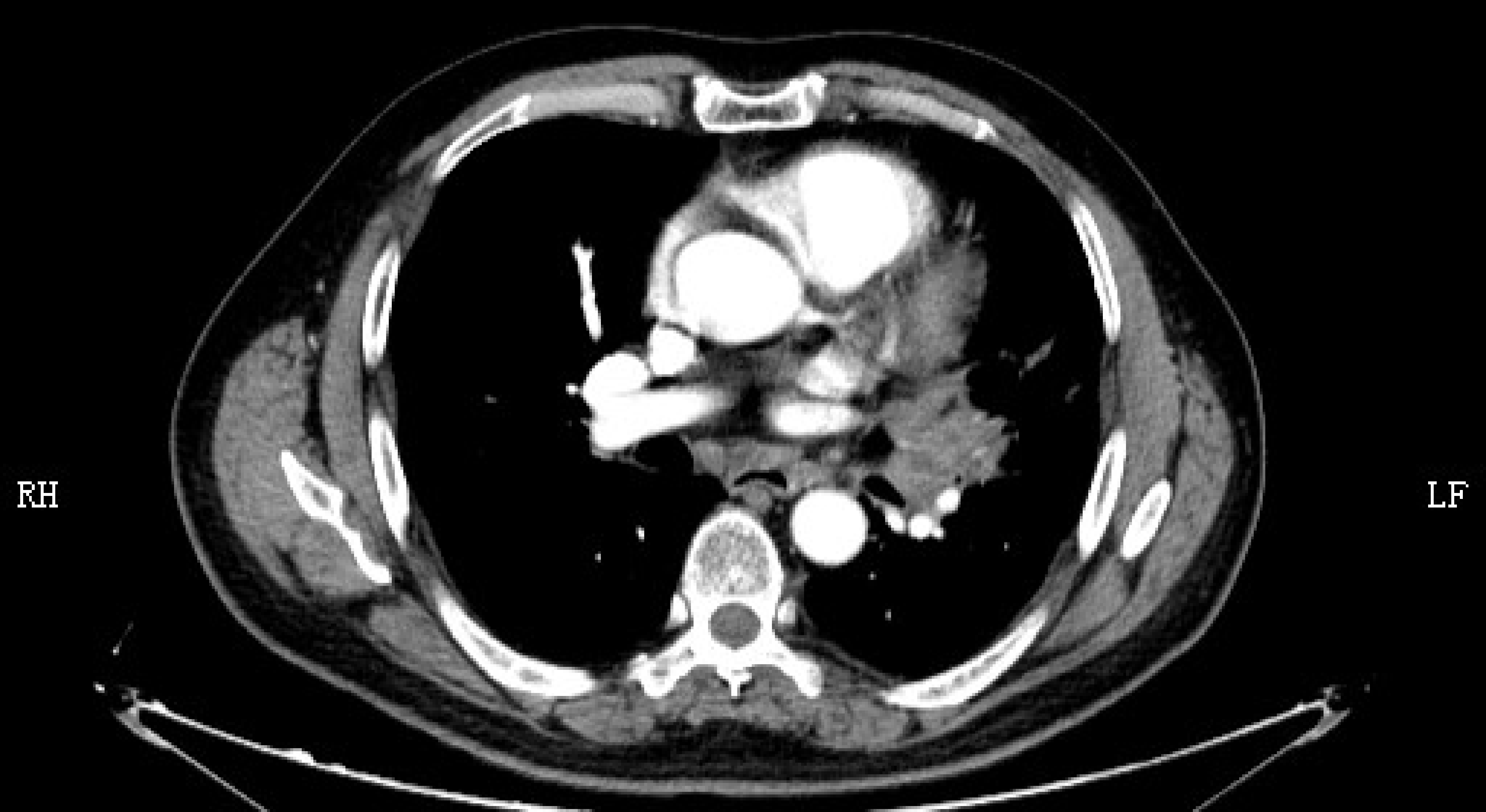
RH
LF

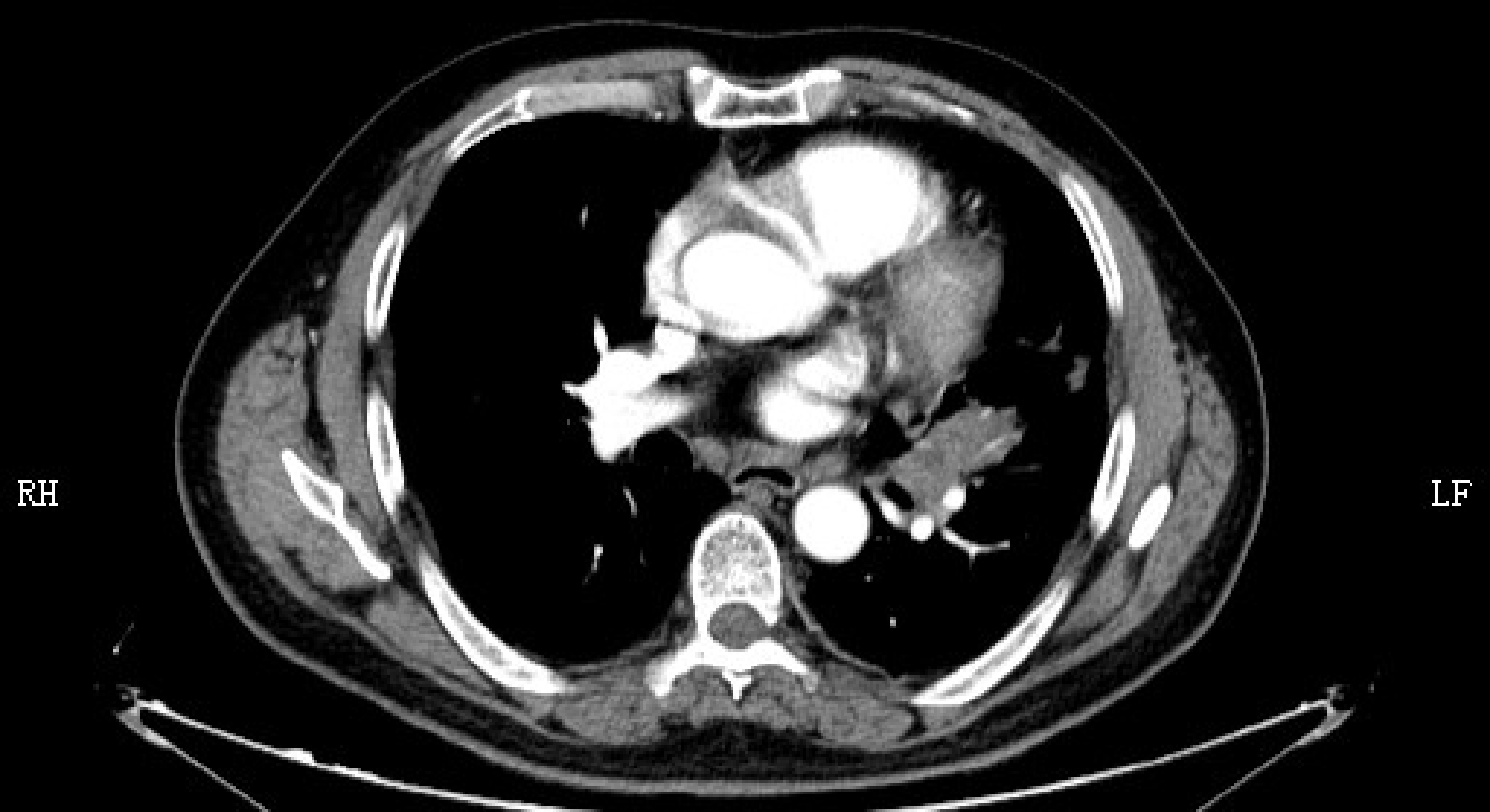
RH
LF

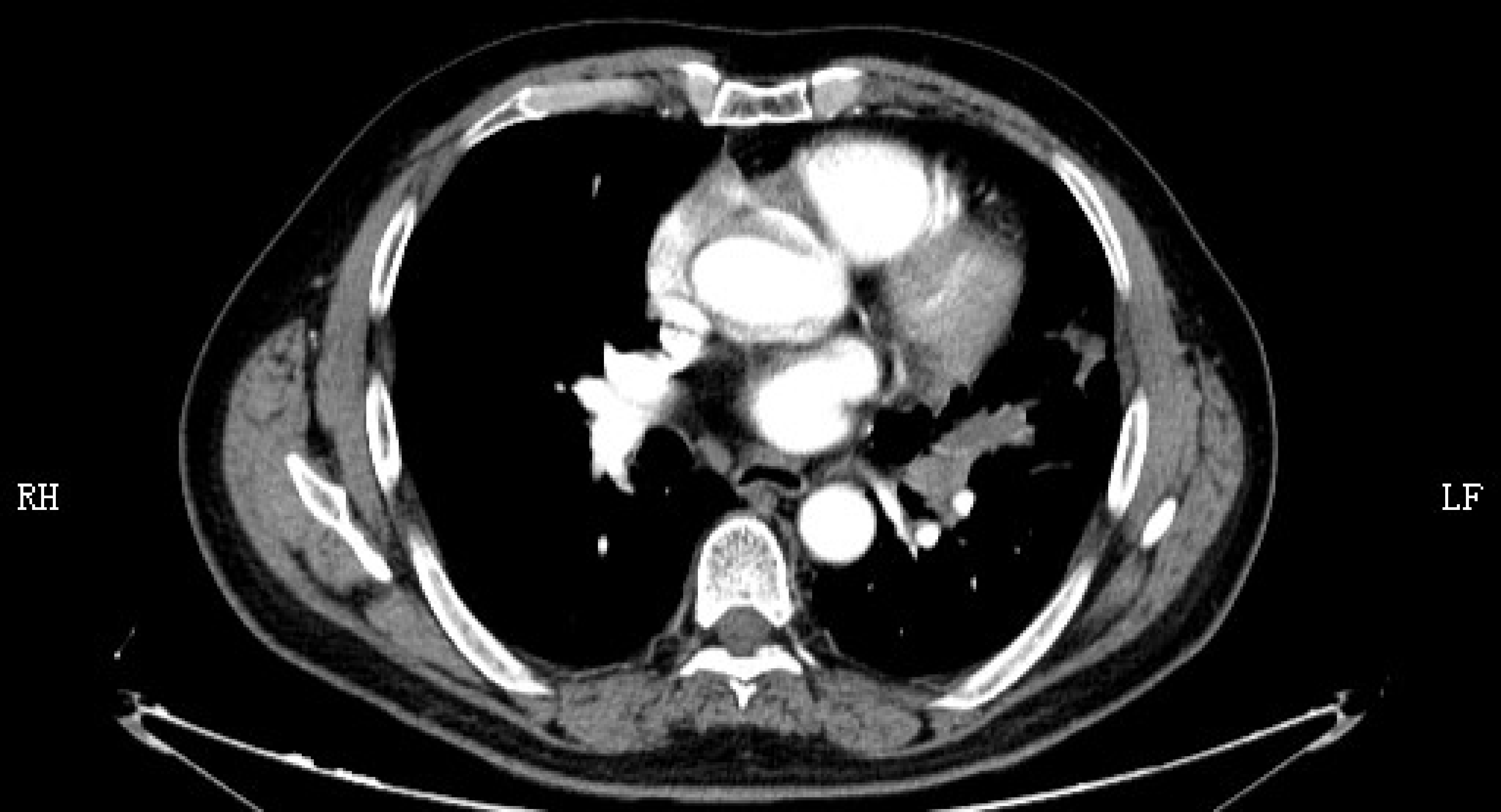
RH
LF

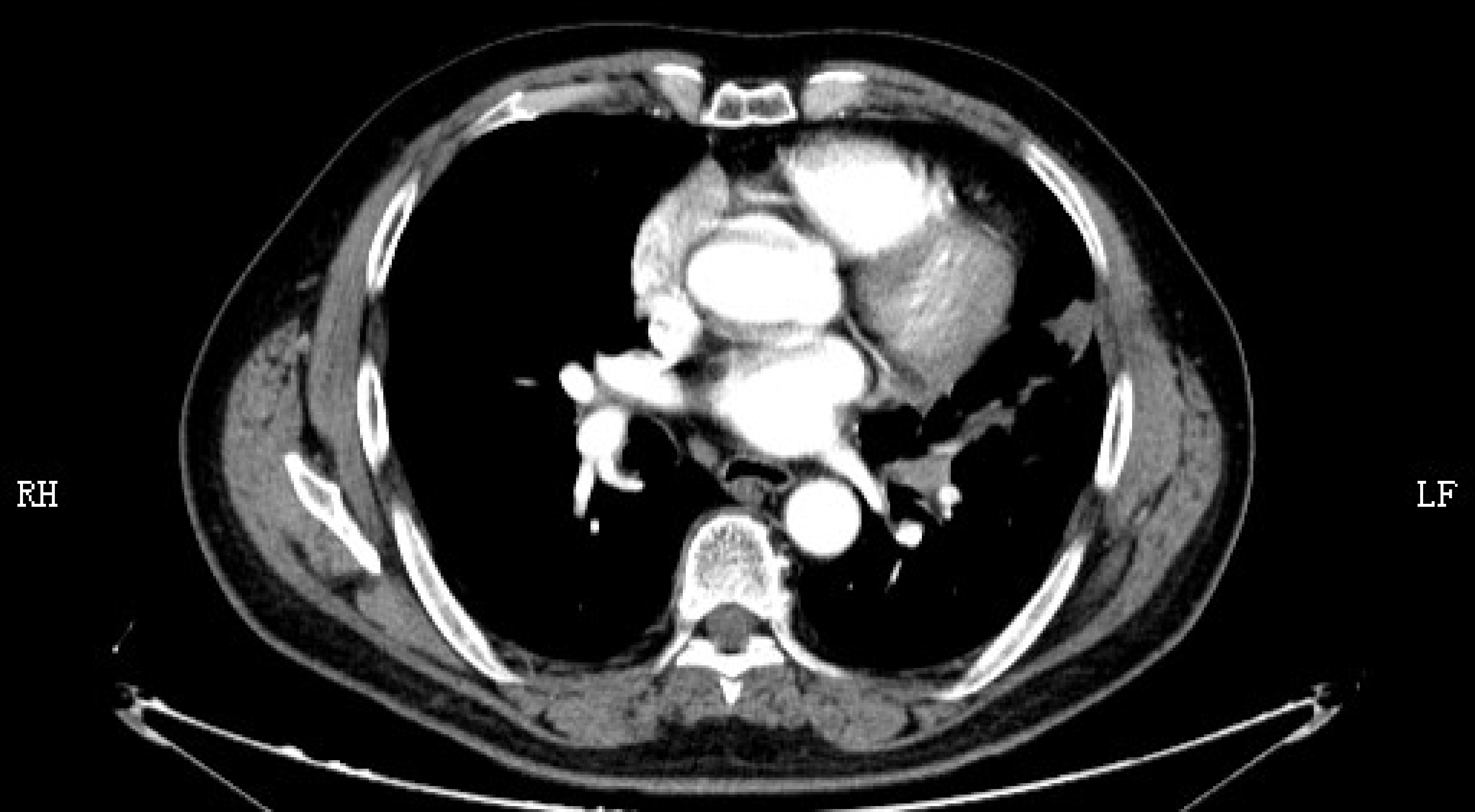
RH
LF
