# Supplementary material for: Sustained complete response to TMEp-CI-M platform in refractory small-cell lung cancer with brainstem metastasis: a case report with over 20 months of disease-free survival
Source: Front Immunol. 2026 Jun 1;17:1807865. doi: 10.3389/fimmu.2026.1807865 (PMC13265516; doi:10.3389/fimmu.2026.1807865)
Supplement: Supplementary Figure 4 — GTV delineation of pulmonary lesion (The GTV is delineated by the red line). [file DataSheet2.pdf]

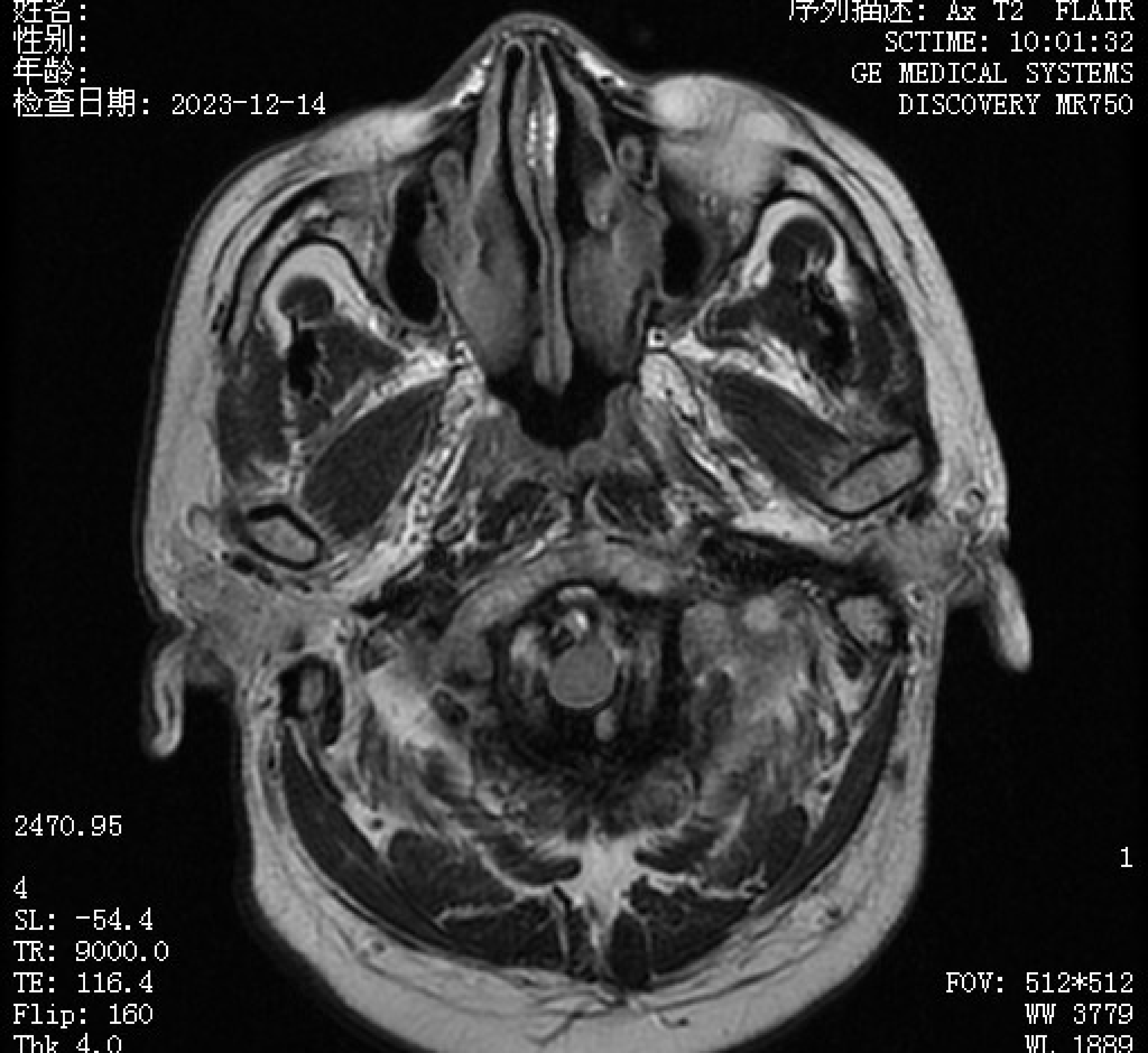

姓名:
性别:
年龄:
检查日期: 2023-12-14
序列描述: Ax T2 FLAIR
SCTIME: 10:01:32
GE MEDICAL SYSTEMS
DISCOVERY MR750
2470.95
4
SL: -54.4
TR: 9000.0
TE: 116.4
Flip: 160
Thk 4.0
1
FOV: 512*512
WW 3779
WL 1889

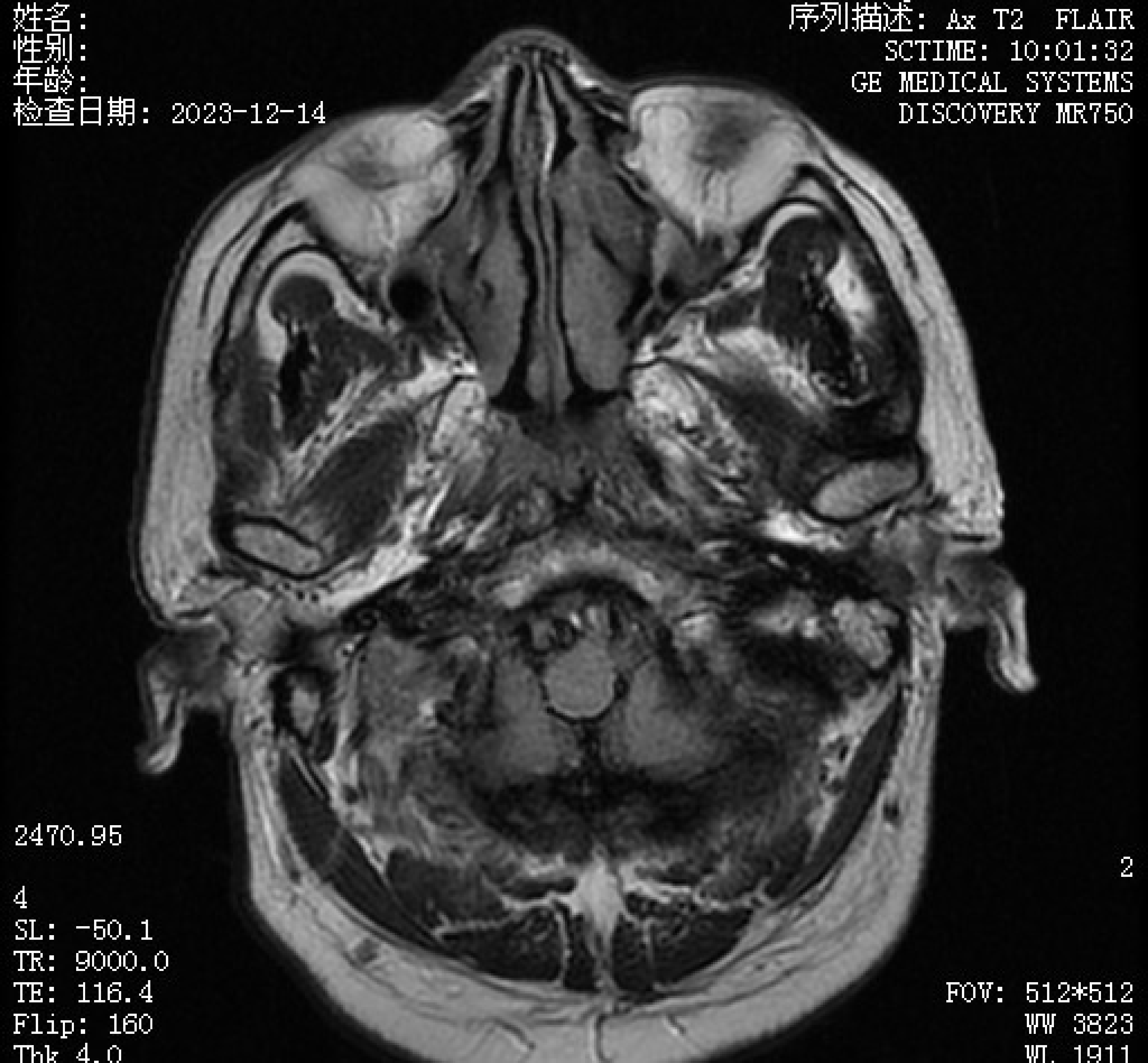

姓名:
性别:
年龄:
检查日期: 2023-12-14
序列描述: Ax T2 FLAIR
SCTIME: 10:01:32
GE MEDICAL SYSTEMS
DISCOVERY MR750
2470.95
2
4
SL: -50.1
TR: 9000.0
TE: 116.4
Flip: 160
Thk 4.0
FOV: 512*512
WW 3823
WL 1911

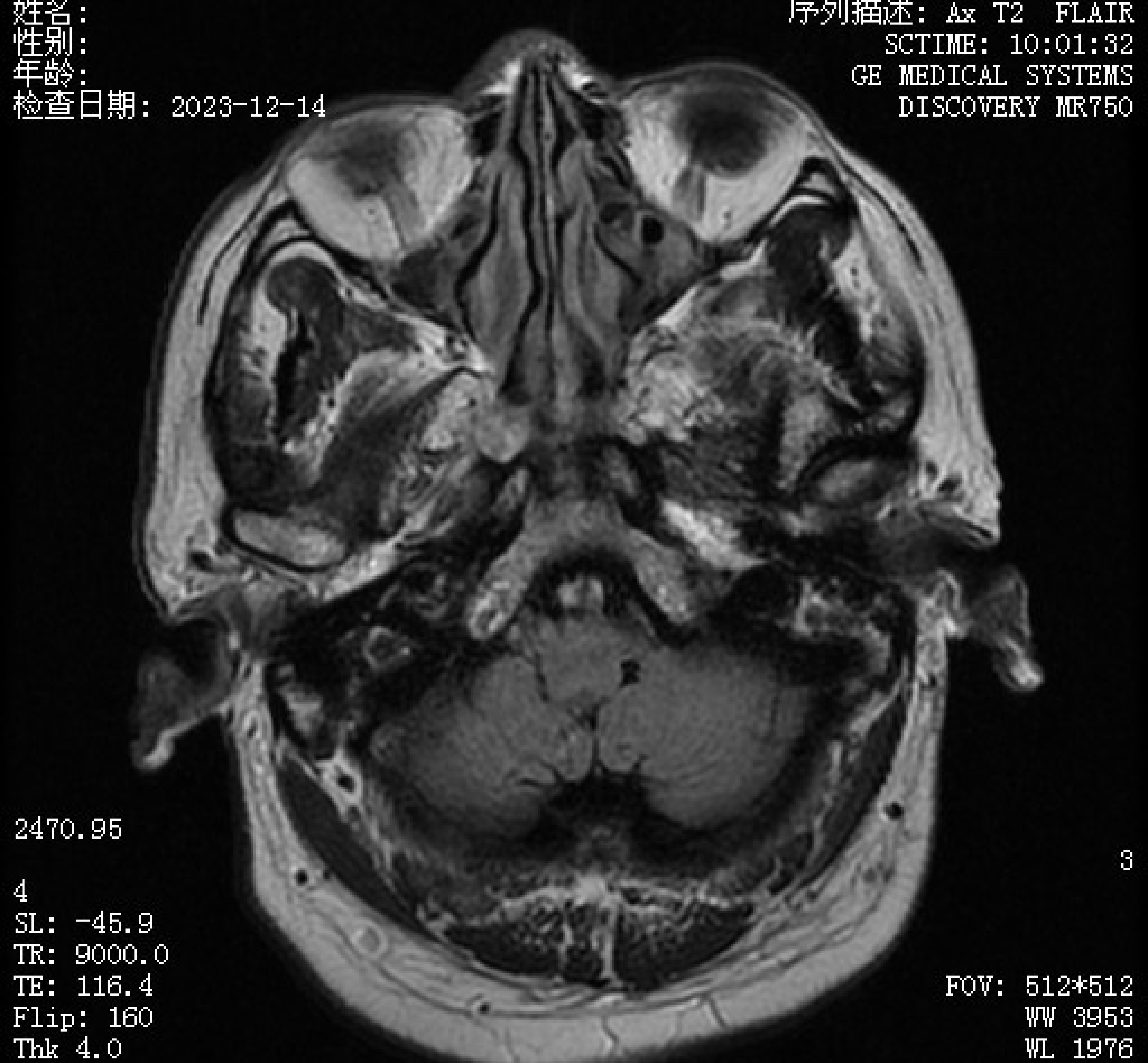
姓名:
性别:
年龄:
检查日期: 2023-12-14
序列描述: Ax T2 FLAIR
SCTIME: 10:01:32
GE MEDICAL SYSTEMS
DISCOVERY MR750
2470.95
3
4
SL: -45.9
TR: 9000.0
TE: 116.4
Flip: 160
Thk 4.0
FOV: 512*512
WW 3953
WL 1976

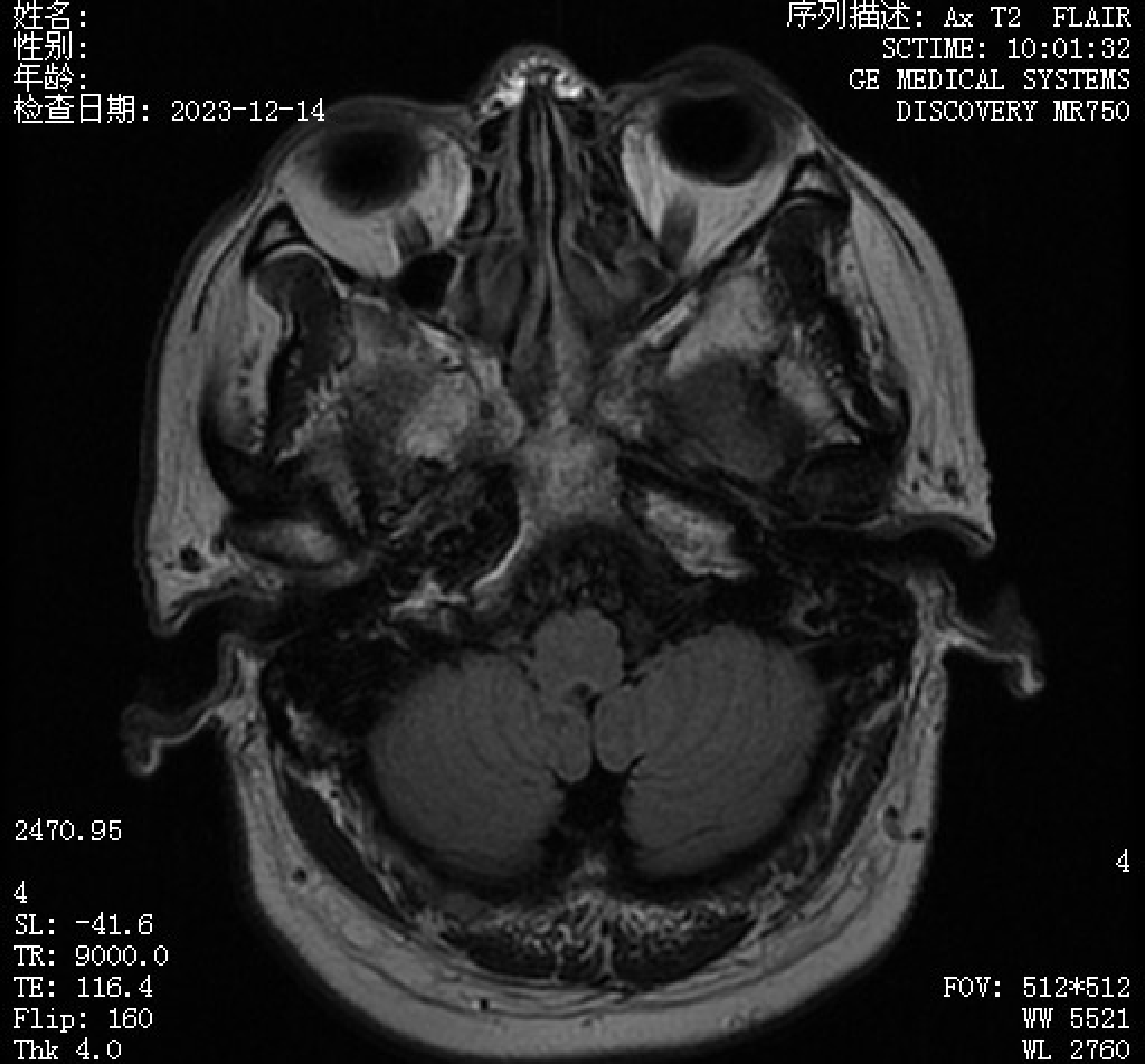
姓名:
性别:
年龄:
检查日期: 2023-12-14
序列描述: Ax T2 FLAIR
SCTIME: 10:01:32
GE MEDICAL SYSTEMS
DISCOVERY MR750
2470.95
4
4
SL: -41.6
TR: 9000.0
TE: 116.4
Flip: 160
Thk 4.0
FOV: 512*512
WW 5521
WL 2760

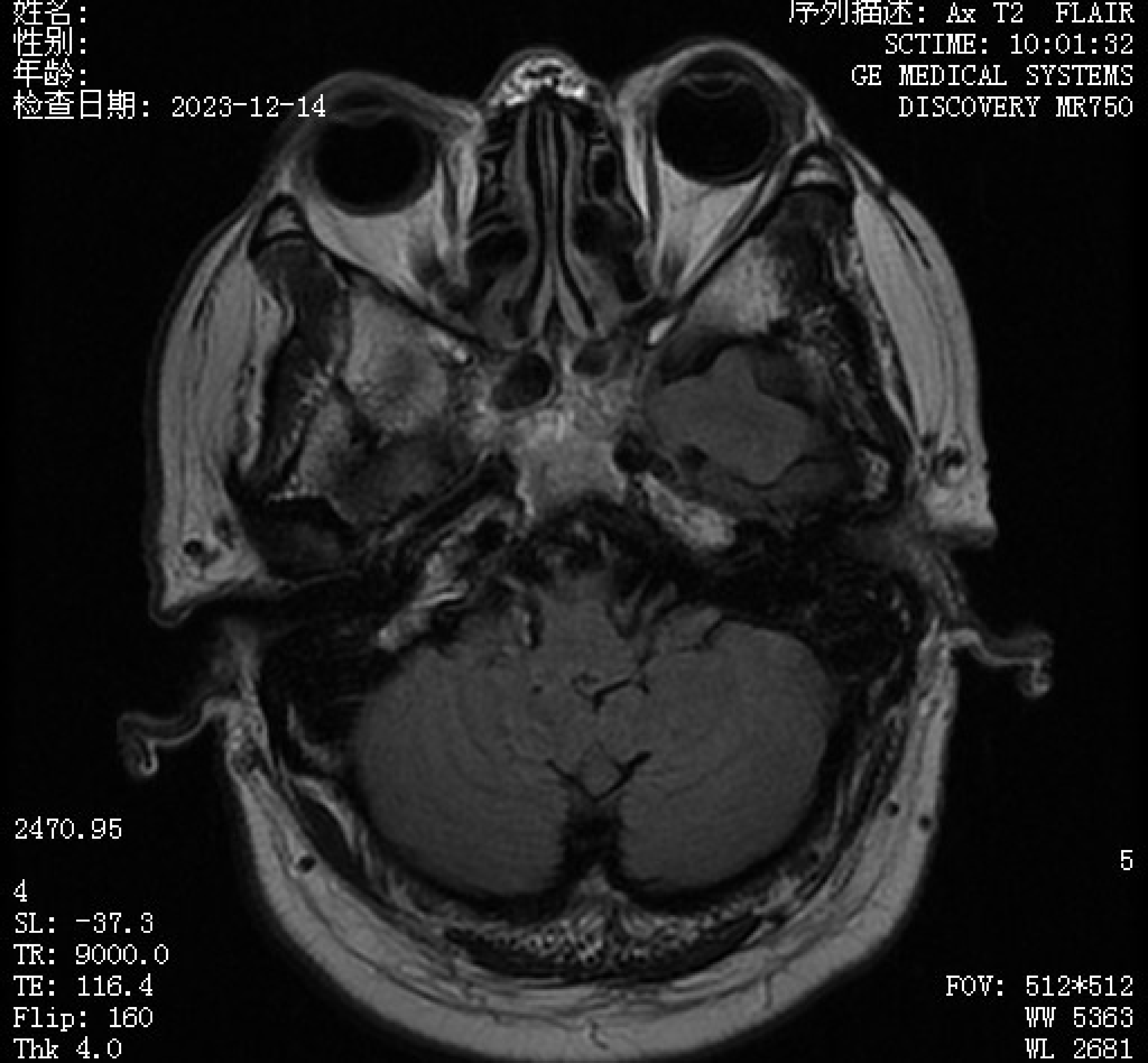

姓名:
性别:
年龄:
检查日期: 2023-12-14
序列描述: Ax T2 FLAIR
SCTIME: 10:01:32
GE MEDICAL SYSTEMS
DISCOVERY MR750
2470.95
5
4
SL: -37.3
TR: 9000.0
TE: 116.4
Flip: 160
Thk 4.0
FOV: 512*512
WW 5363
WL 2681

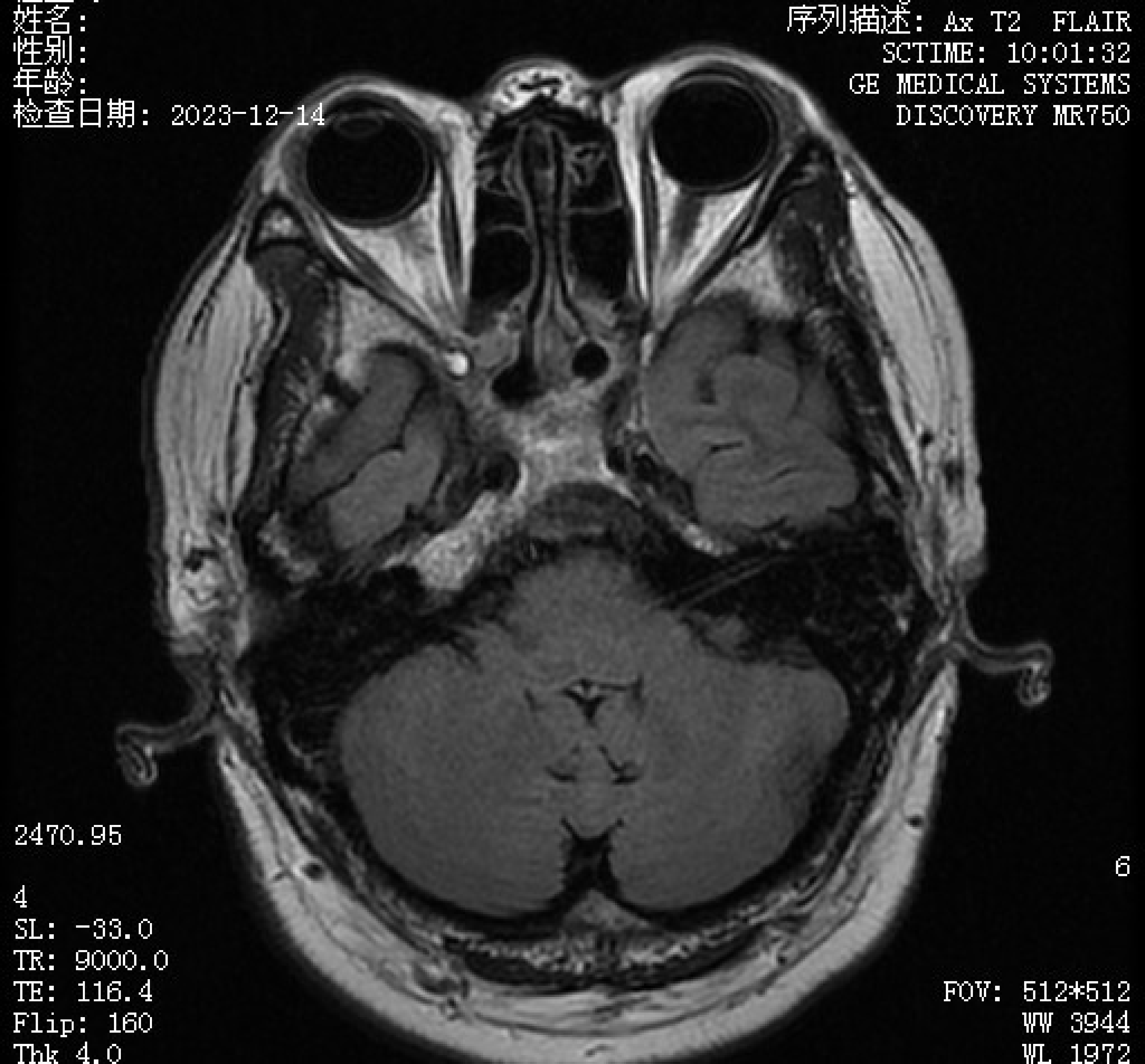
姓名:
性别:
年龄:
检查日期: 2023-12-14
序列描述: Ax T2 FLAIR
SCTIME: 10:01:32
GE MEDICAL SYSTEMS
DISCOVERY MR750
2470.95
6
4
SL: -33.0
TR: 9000.0
TE: 116.4
Flip: 160
Thk 4.0
FOV: 512*512
WW 3944
WL 1972

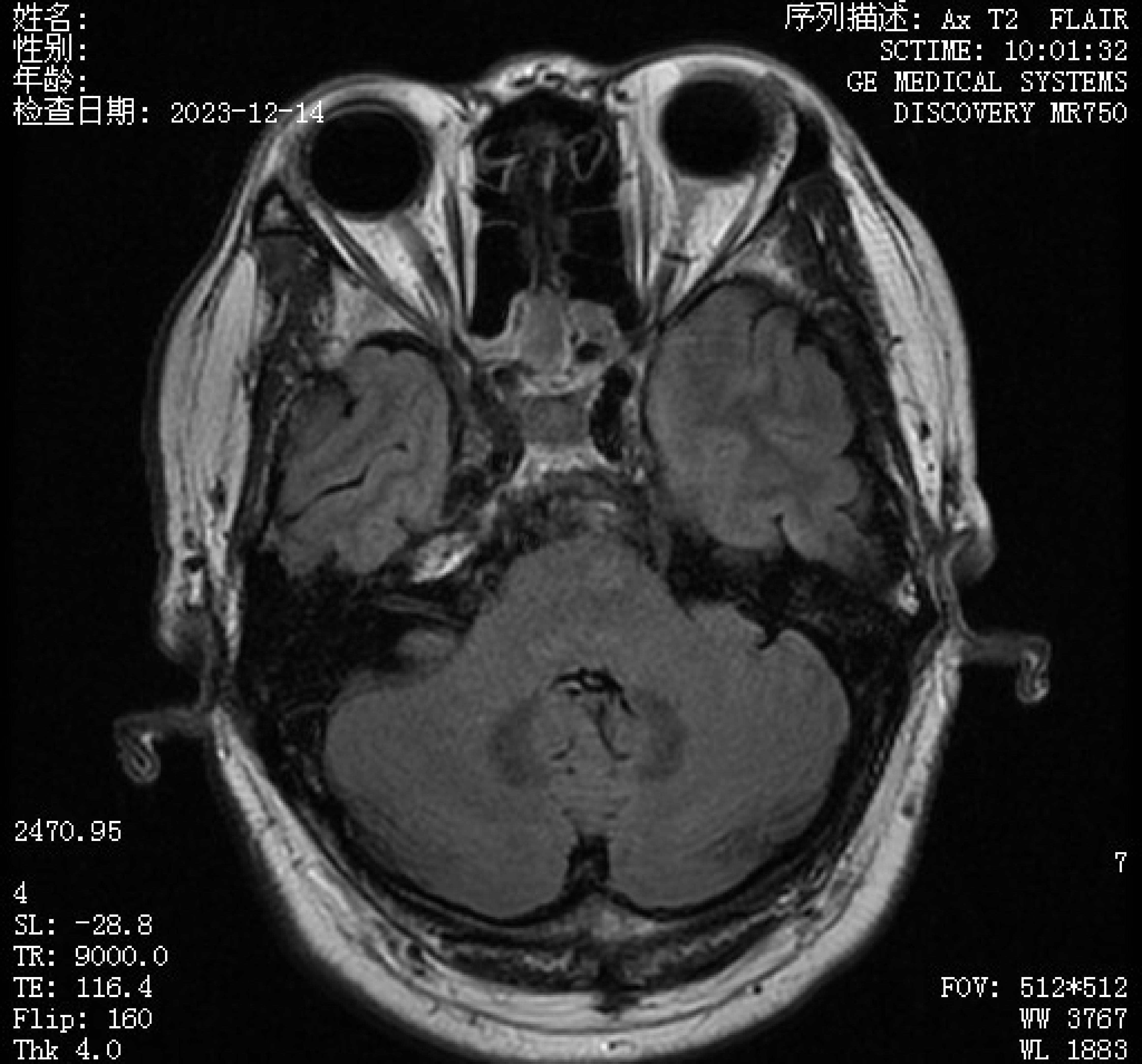
姓名:
性别:
年龄:
检查日期: 2023-12-14
序列描述: Ax T2 FLAIR
SCTIME: 10:01:32
GE MEDICAL SYSTEMS
DISCOVERY MR750
2470.95
7
4
SL: -28.8
TR: 9000.0
TE: 116.4
Flip: 160
Thk 4.0
FOV: 512*512
WW 3767
WL 1883

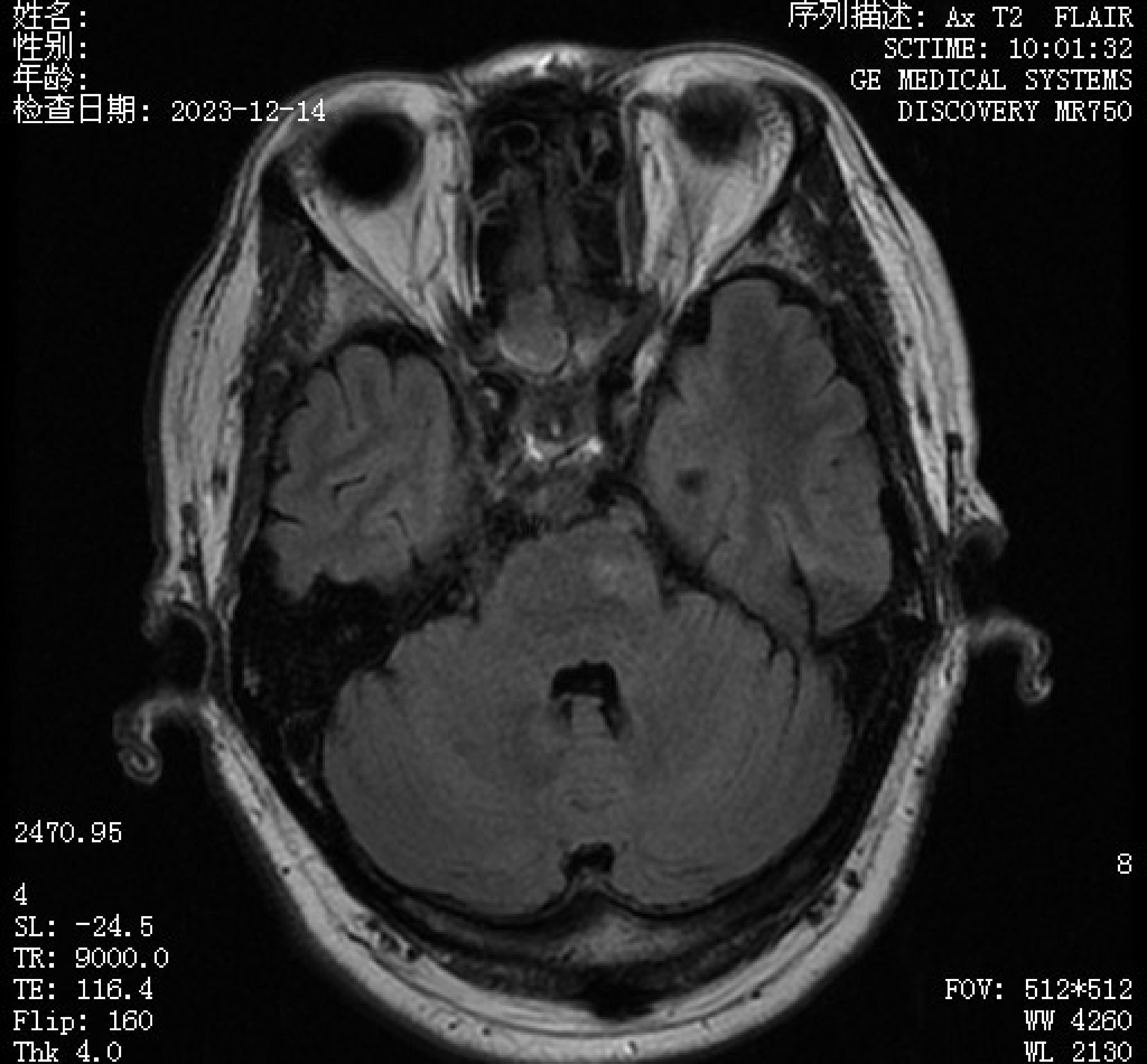
姓名:
性别:
年龄:
检查日期: 2023-12-14
序列描述: Ax T2 FLAIR
SCTIME: 10:01:32
GE MEDICAL SYSTEMS
DISCOVERY MR750
2470.95
8
4
SL: -24.5
TR: 9000.0
TE: 116.4
Flip: 160
Thk 4.0
FOV: 512*512
WW 4260
WL 2130

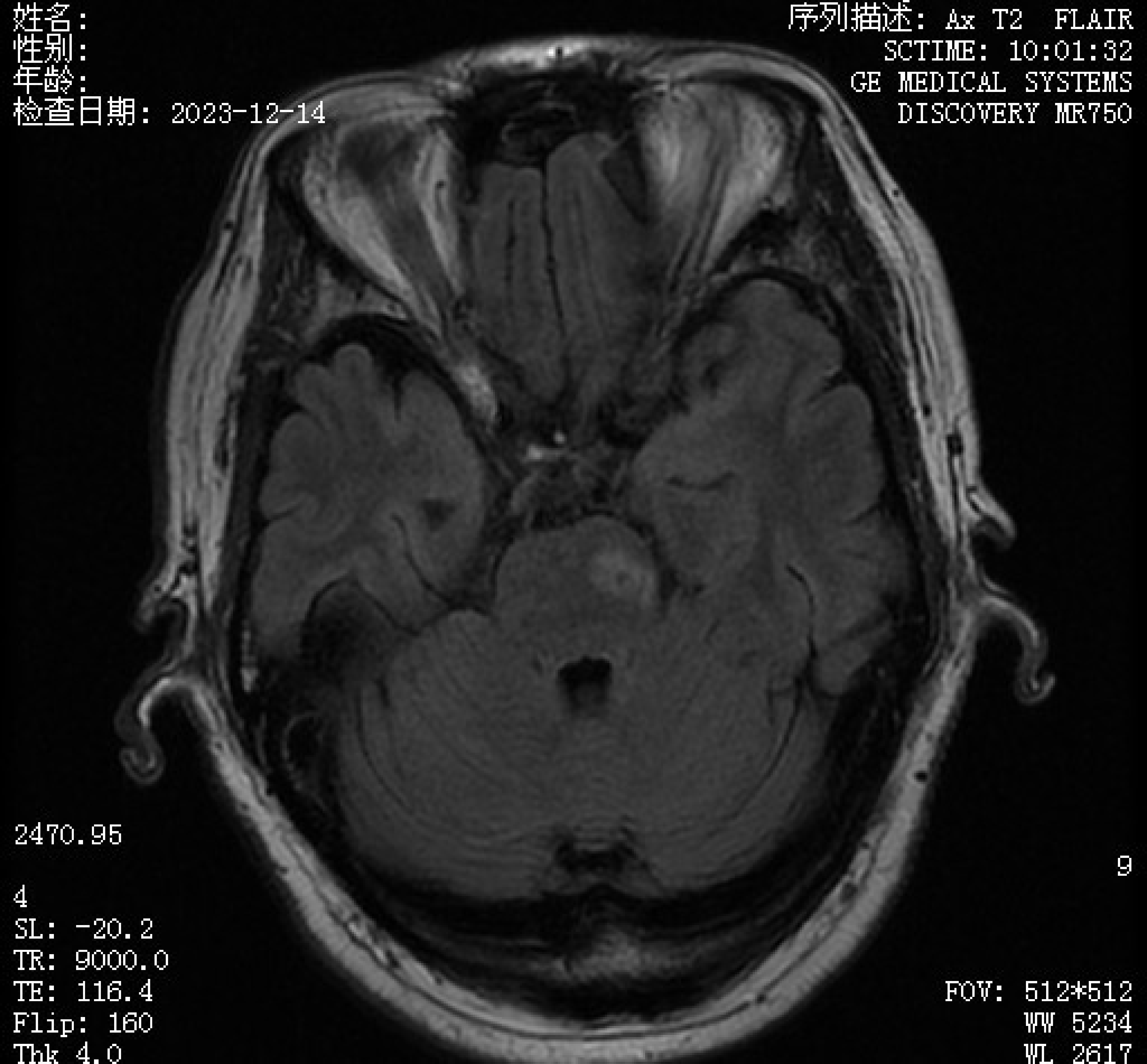
姓名:
性别:
年龄:
检查日期: 2023-12-14
序列描述: Ax T2 FLAIR
SCTIME: 10:01:32
GE MEDICAL SYSTEMS
DISCOVERY MR750
2470.95
9
4
SL: -20.2
TR: 9000.0
TE: 116.4
Flip: 160
Thk 4.0
FOV: 512*512
WW 5234
WL 2617

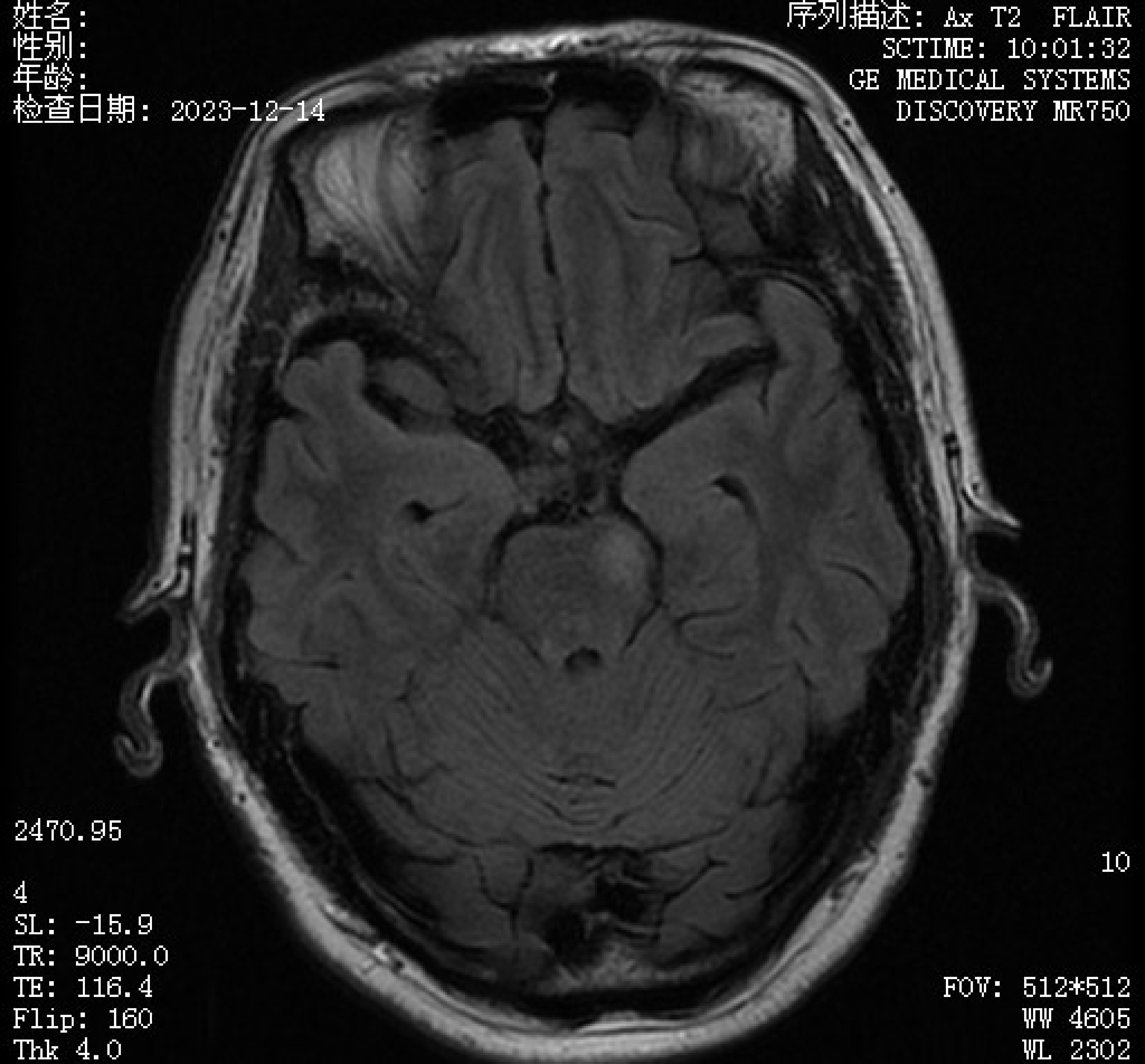
姓名:
性别:
年龄:
检查日期: 2023-12-14
序列描述: Ax T2 FLAIR
SCTIME: 10:01:32
GE MEDICAL SYSTEMS
DISCOVERY MR750
2470.95
10
4
SL: -15.9
TR: 9000.0
TE: 116.4
Flip: 160
Thk 4.0
FOV: 512*512
WW 4605
WL 2302

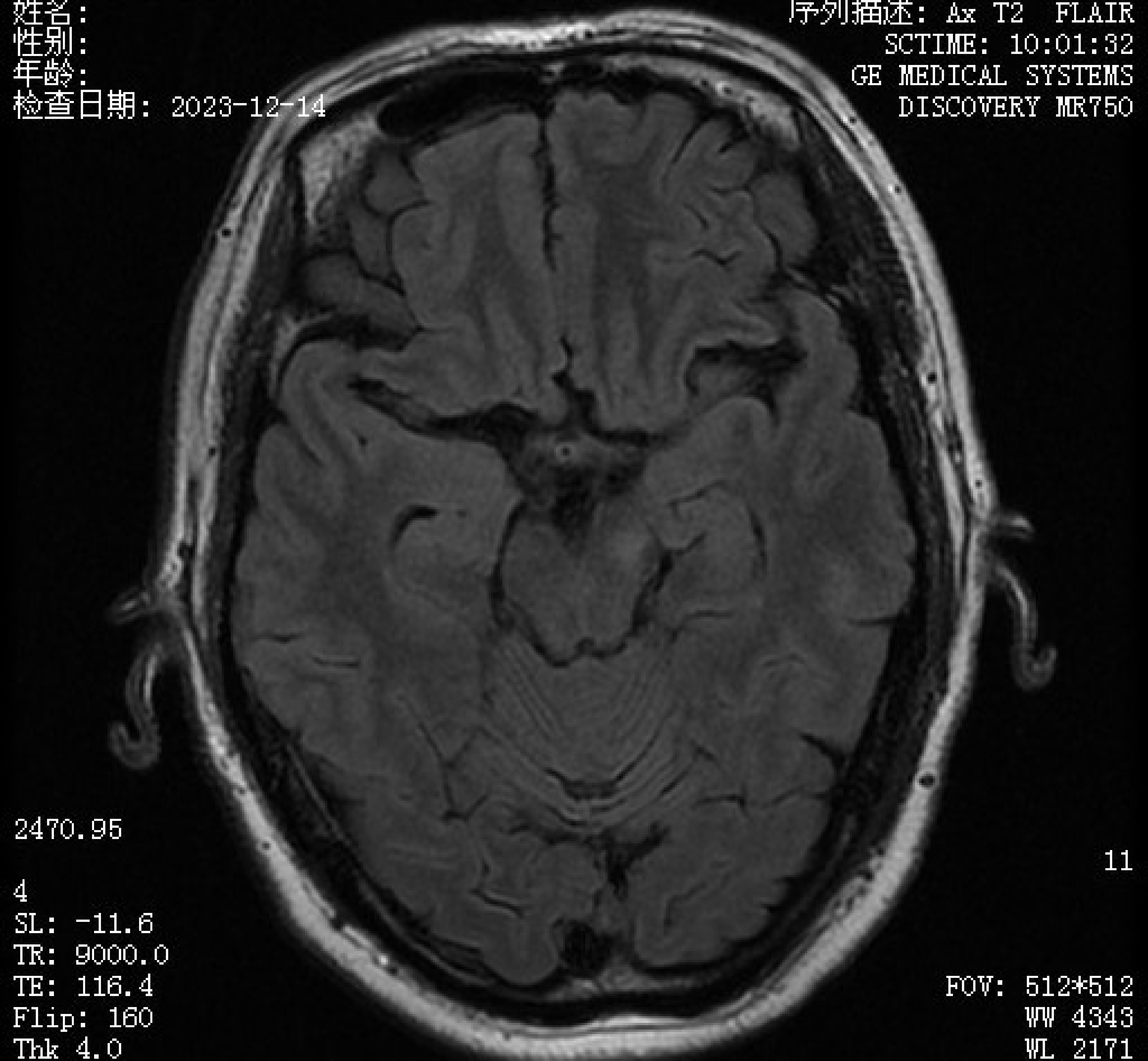
姓名:
性别:
年龄:
检查日期: 2023-12-14
序列描述: Ax T2 FLAIR
SCTIME: 10:01:32
GE MEDICAL SYSTEMS
DISCOVERY MR750
2470.95
11
4
SL: -11.6
TR: 9000.0
TE: 116.4
Flip: 160
Thk 4.0
FOV: 512*512
WW 4343
WL 2171

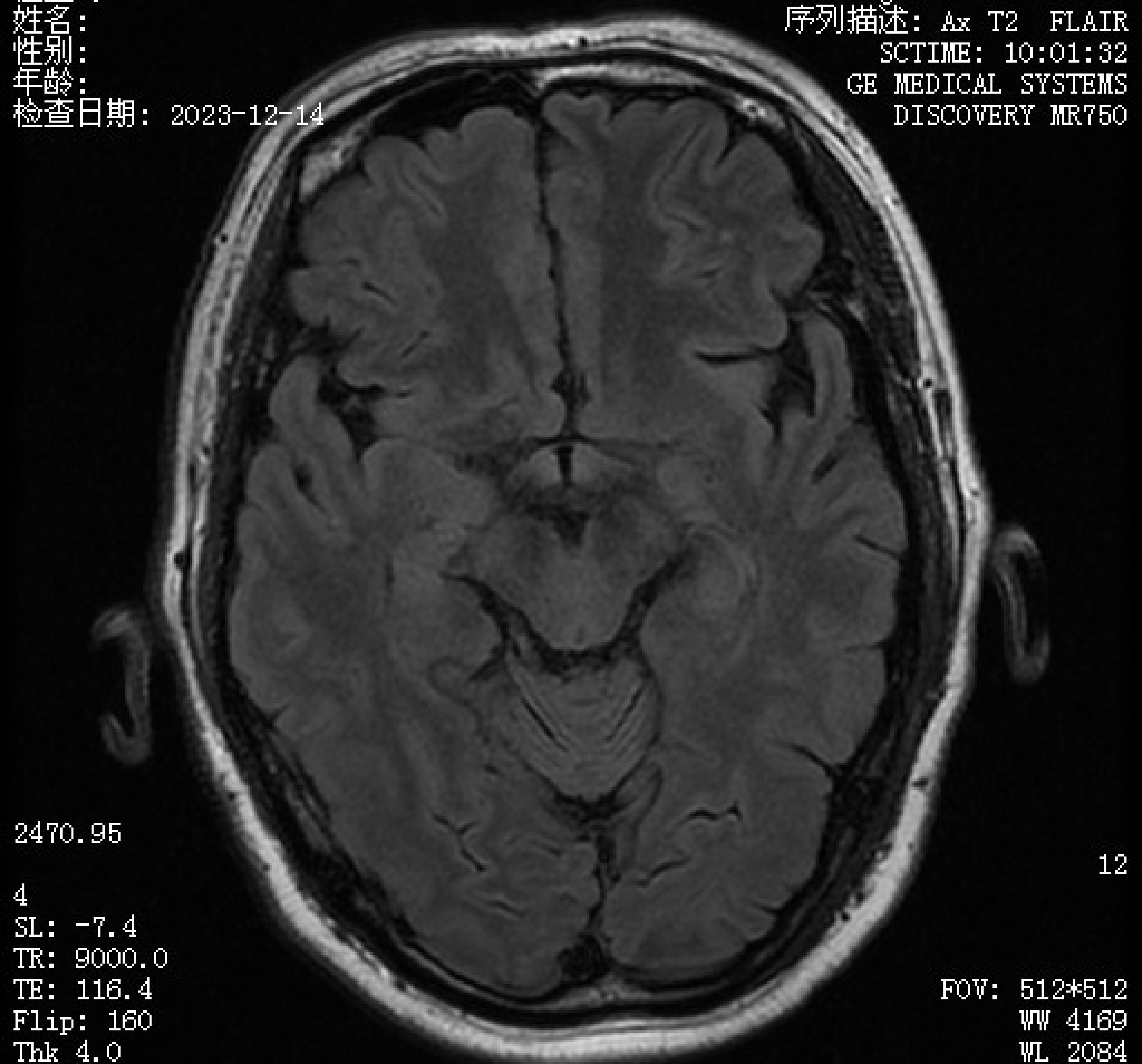
姓名:
性别:
年龄:
检查日期: 2023-12-14
序列描述: Ax T2 FLAIR
SCTIME: 10:01:32
GE MEDICAL SYSTEMS
DISCOVERY MR750
2470.95
4
SL: -7.4
TR: 9000.0
TE: 116.4
Flip: 160
Thk 4.0
12
FOV: 512*512
WW 4169
WL 2084

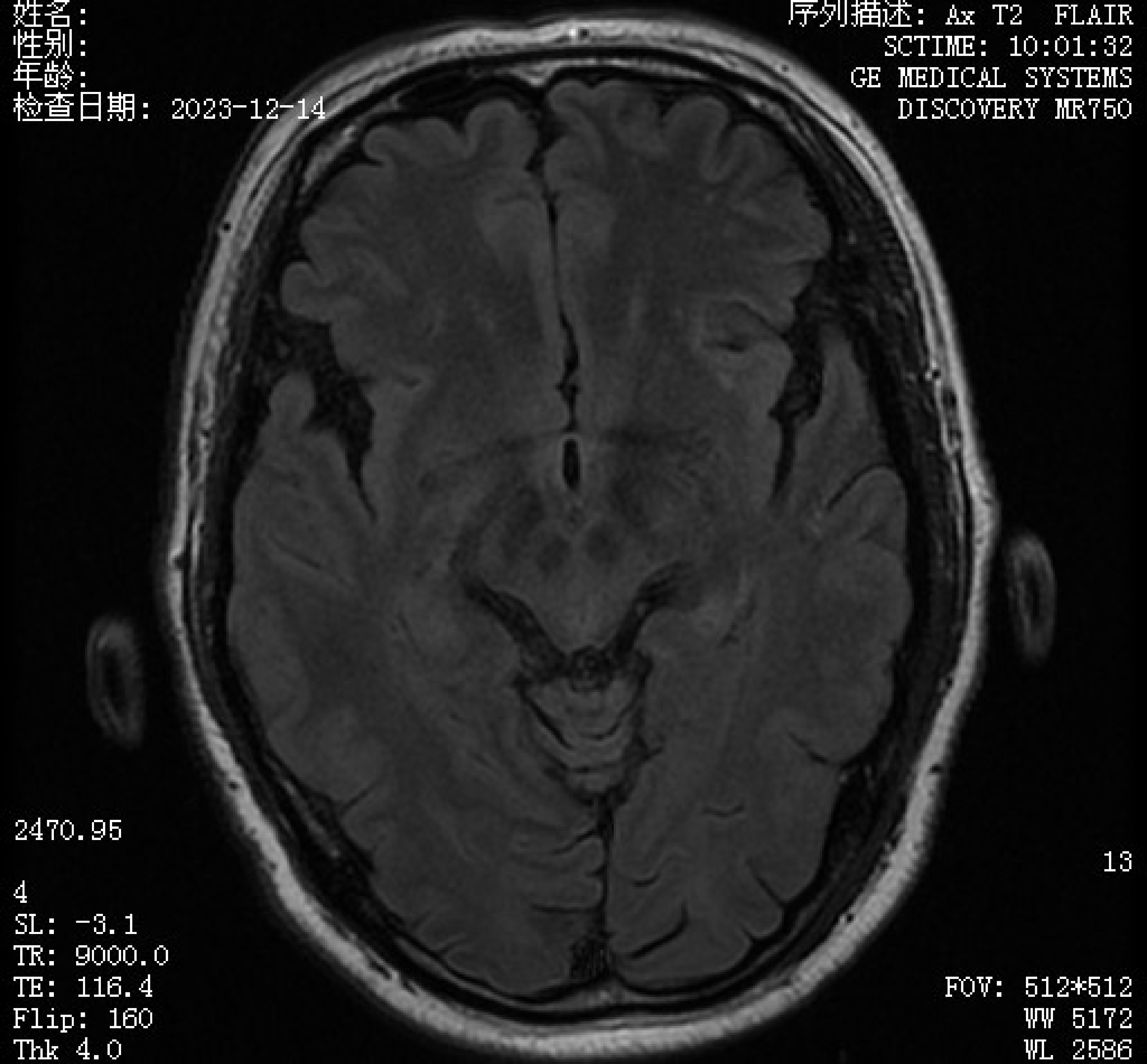
姓名:
性别:
年龄:
检查日期: 2023-12-14
序列描述: Ax T2 FLAIR
SCTIME: 10:01:32
GE MEDICAL SYSTEMS
DISCOVERY MR750
2470.95
13
4
SL: -3.1
TR: 9000.0
TE: 116.4
Flip: 160
Thk 4.0
FOV: 512*512
WW 5172
WL 2586

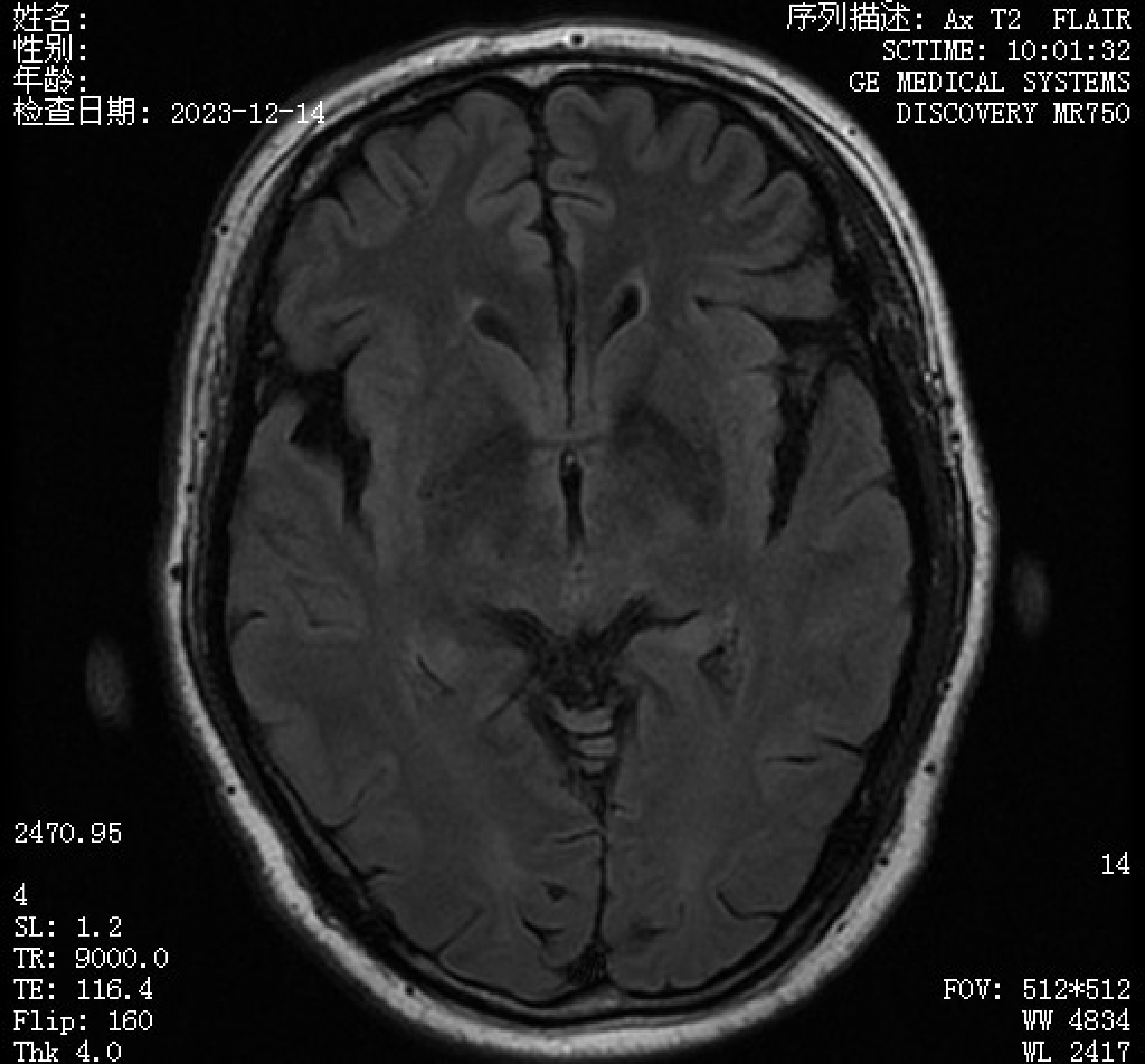

姓名:
性别:
年龄:
检查日期: 2023-12-14
序列描述: Ax T2 FLAIR
SCTIME: 10:01:32
GE MEDICAL SYSTEMS
DISCOVERY MR750
2470.95
4
SL: 1.2
TR: 9000.0
TE: 116.4
Flip: 160
Thk 4.0
14
FOV: 512*512
WW 4834
WL 2417

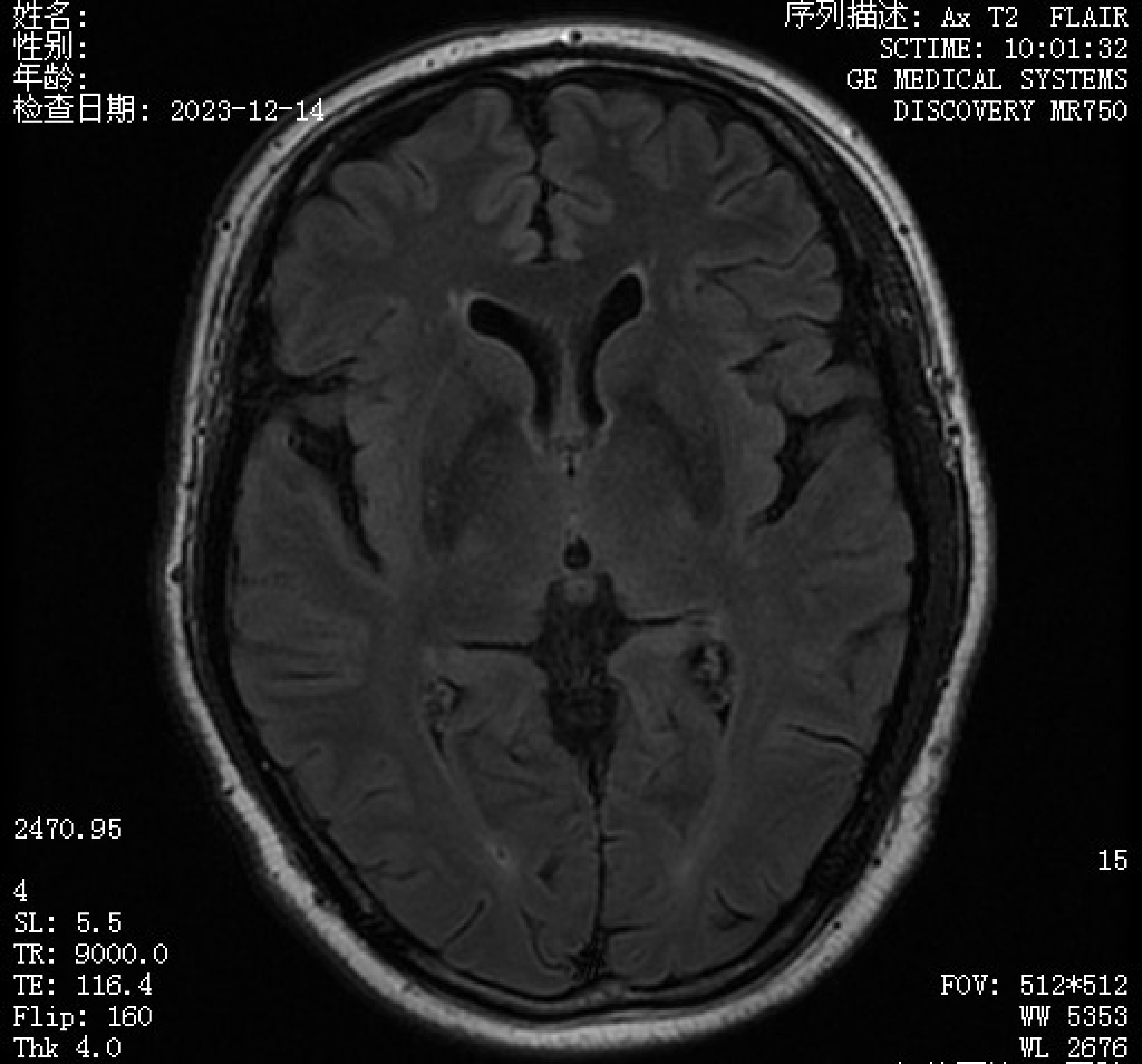
姓名:
性别:
年龄:
检查日期: 2023-12-14
序列描述: Ax T2 FLAIR
SCTIME: 10:01:32
GE MEDICAL SYSTEMS
DISCOVERY MR750
2470.95
4
SL: 5.5
TR: 9000.0
TE: 116.4
Flip: 160
Thk 4.0
15
FOV: 512*512
WW 5353
WL 2676

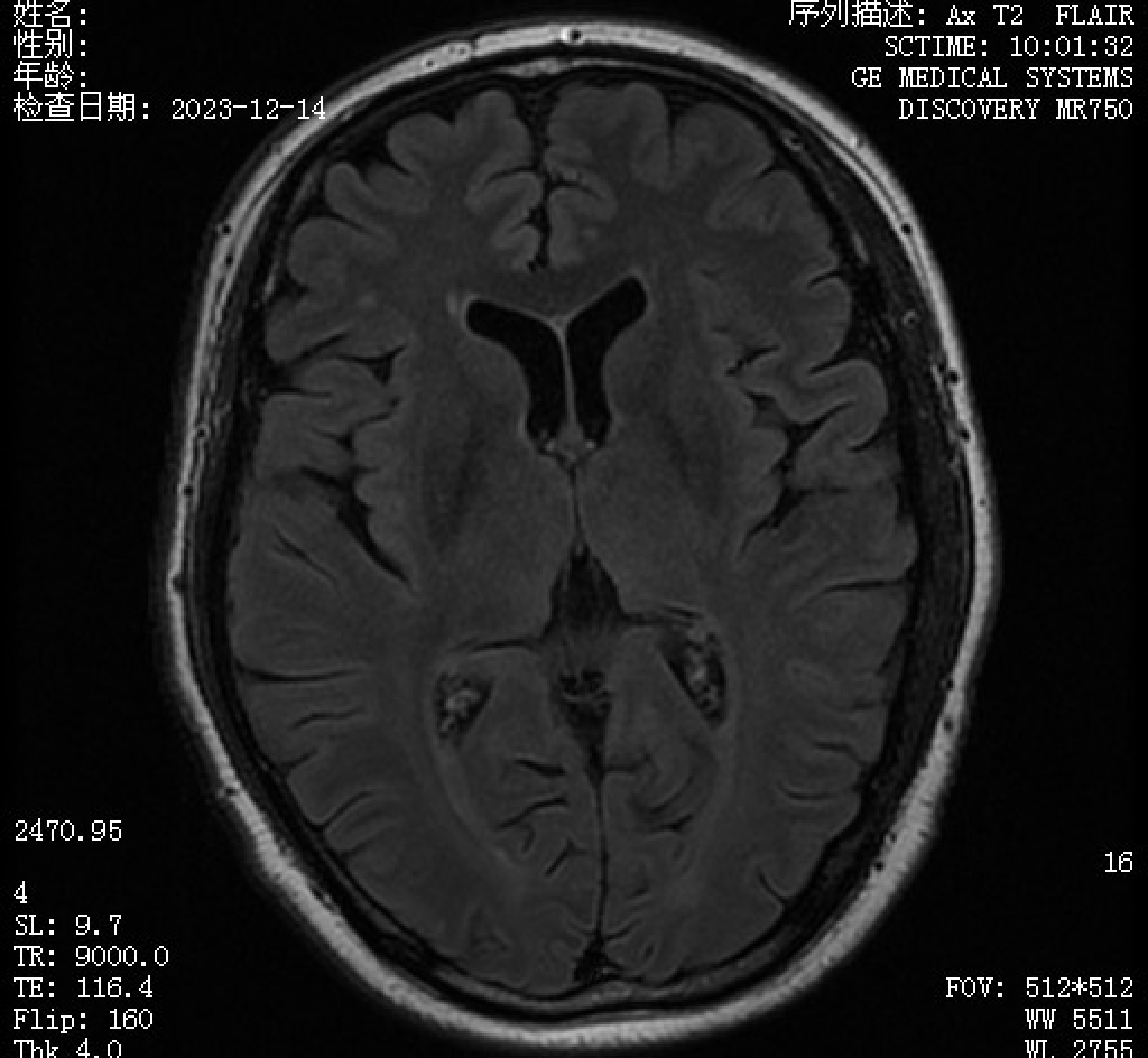
姓名:
性别:
年龄:
检查日期: 2023-12-14
序列描述: Ax T2 FLAIR
SCTIME: 10:01:32
GE MEDICAL SYSTEMS
DISCOVERY MR750
2470.95
16
4
SL: 9.7
TR: 9000.0
TE: 116.4
Flip: 160
Thk 4.0
FOV: 512*512
WW 5511
WL 2755

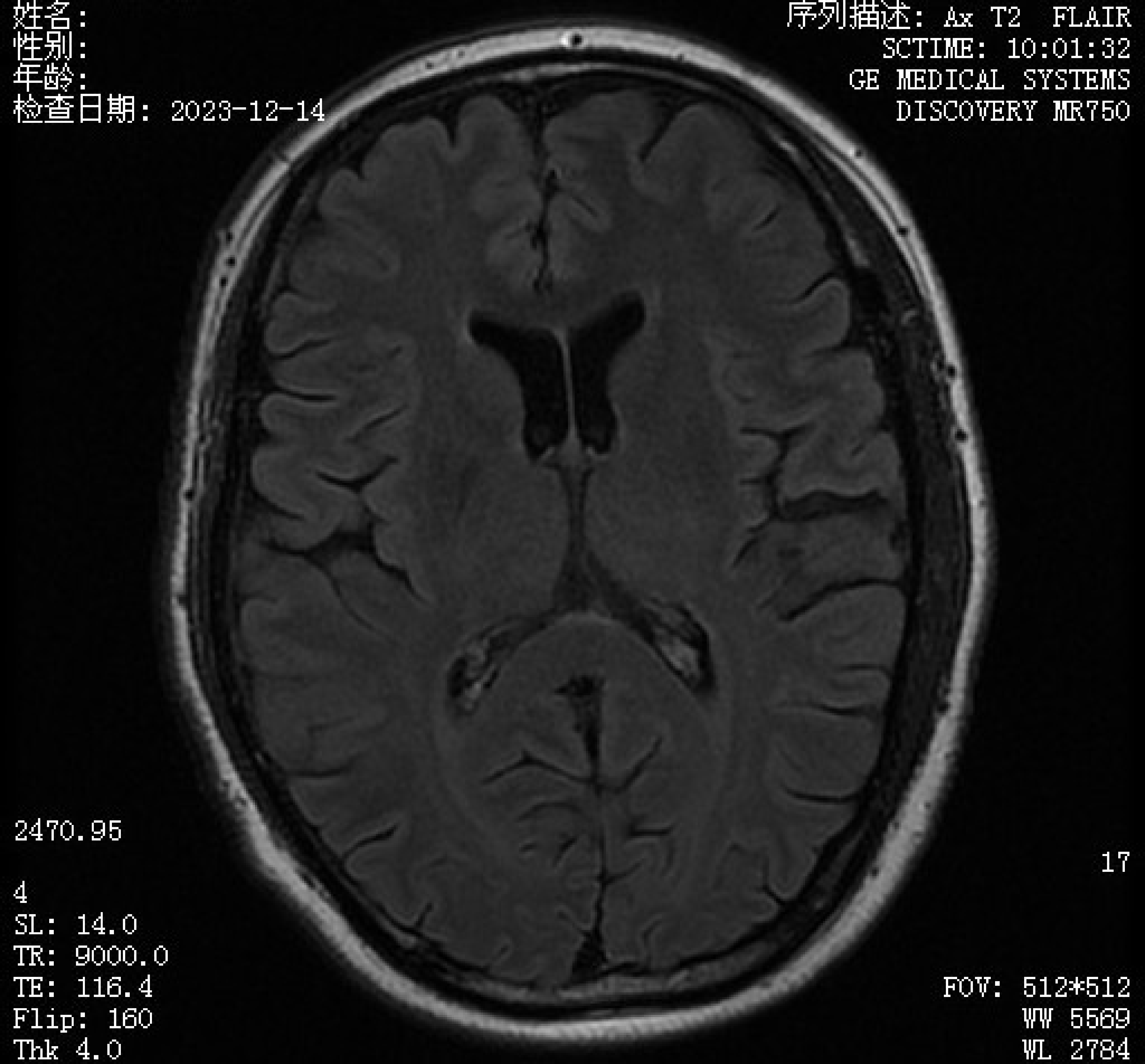
姓名:
性别:
年龄:
检查日期: 2023-12-14
序列描述: Ax T2 FLAIR
SCTIME: 10:01:32
GE MEDICAL SYSTEMS
DISCOVERY MR750
2470.95
17
4
SL: 14.0
TR: 9000.0
TE: 116.4
Flip: 160
Thk 4.0
FOV: 512*512
WW 5569
WL 2784

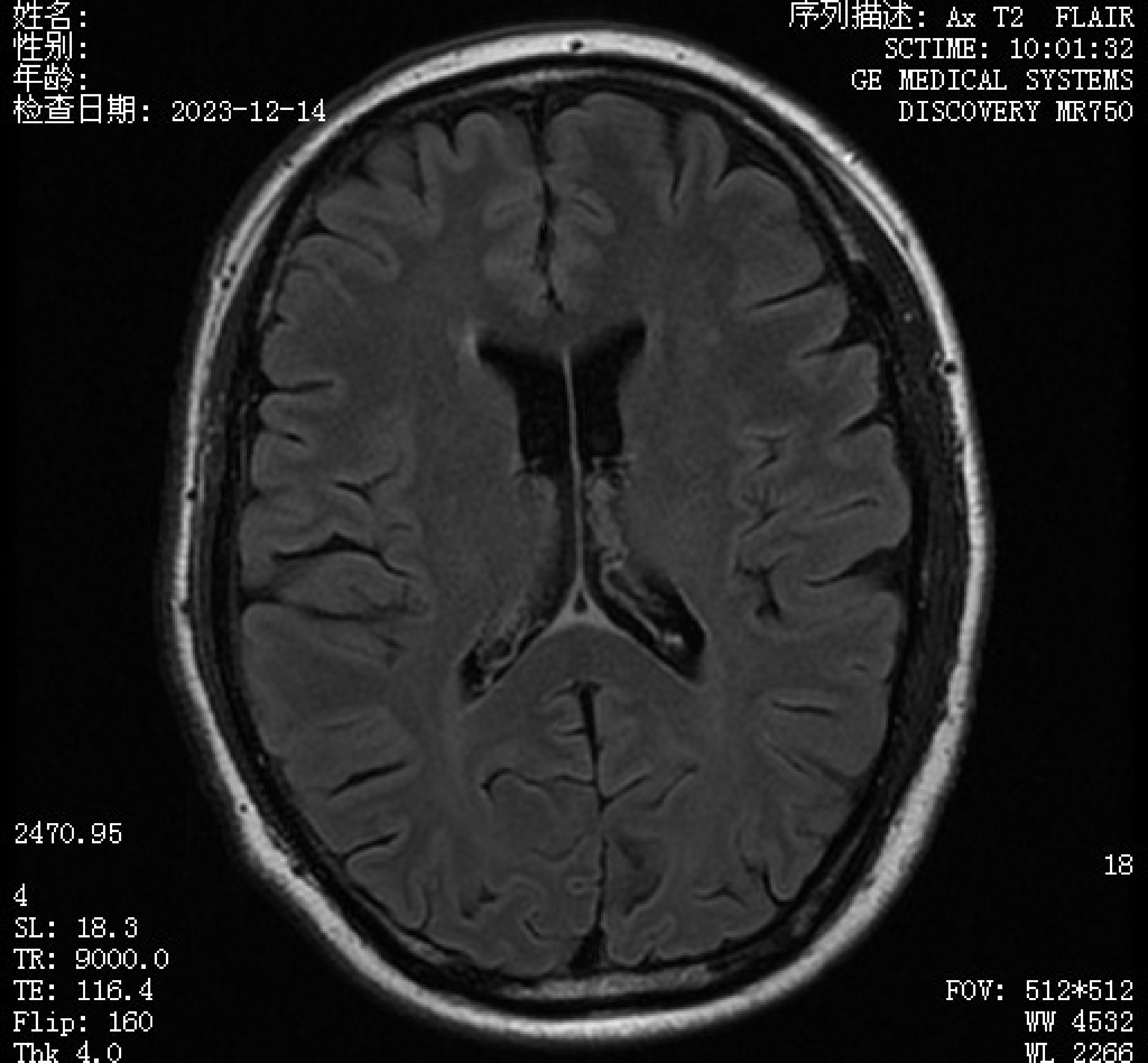
姓名:
性别:
年龄:
检查日期: 2023-12-14
序列描述: Ax T2 FLAIR
SCTIME: 10:01:32
GE MEDICAL SYSTEMS
DISCOVERY MR750
2470.95
18
4
SL: 18.3
TR: 9000.0
TE: 116.4
Flip: 160
Thk 4.0
FOV: 512*512
WW 4532
WL 2266

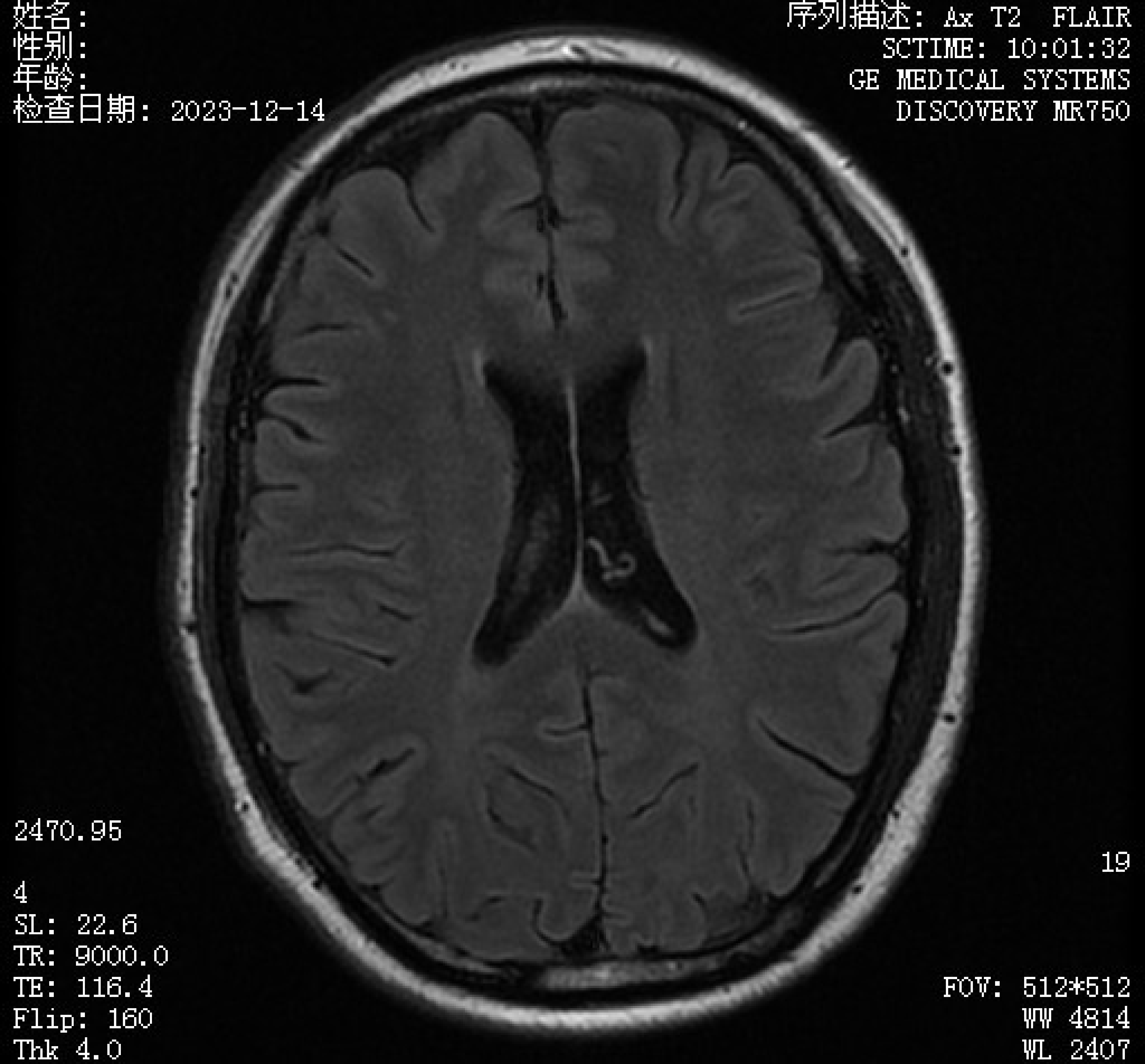
姓名:
性别:
年龄:
检查日期: 2023-12-14
序列描述: Ax T2 FLAIR
SCTIME: 10:01:32
GE MEDICAL SYSTEMS
DISCOVERY MR750
2470.95
4
SL: 22.6
TR: 9000.0
TE: 116.4
Flip: 160
Thk 4.0
19
FOV: 512*512
WW 4814
WL 2407

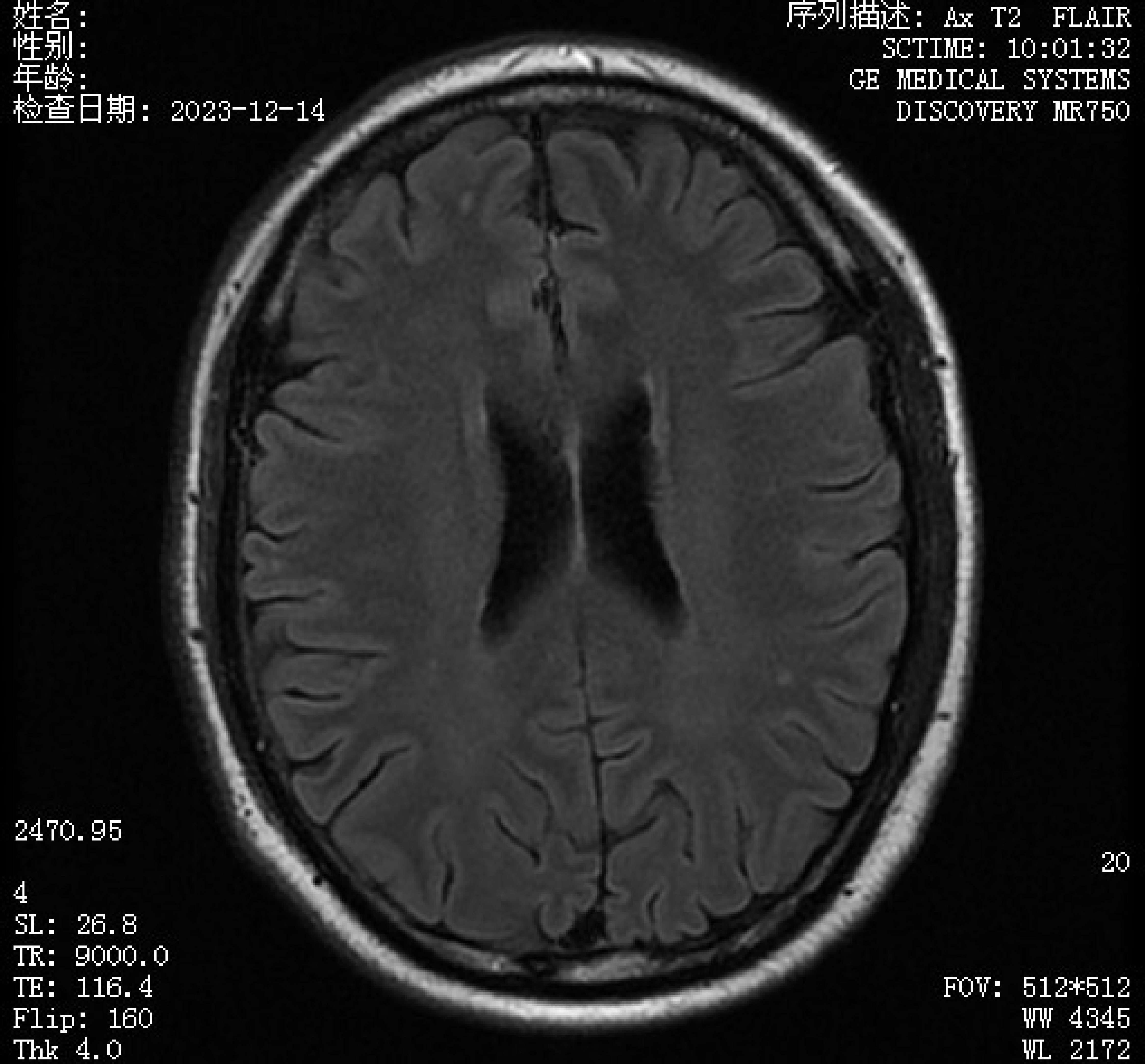
姓名:
性别:
年龄:
检查日期: 2023-12-14
序列描述: Ax T2 FLAIR
SCTIME: 10:01:32
GE MEDICAL SYSTEMS
DISCOVERY MR750
2470.95
20
4
SL: 26.8
TR: 9000.0
TE: 116.4
Flip: 160
Thk 4.0
FOV: 512*512
WW 4345
WL 2172

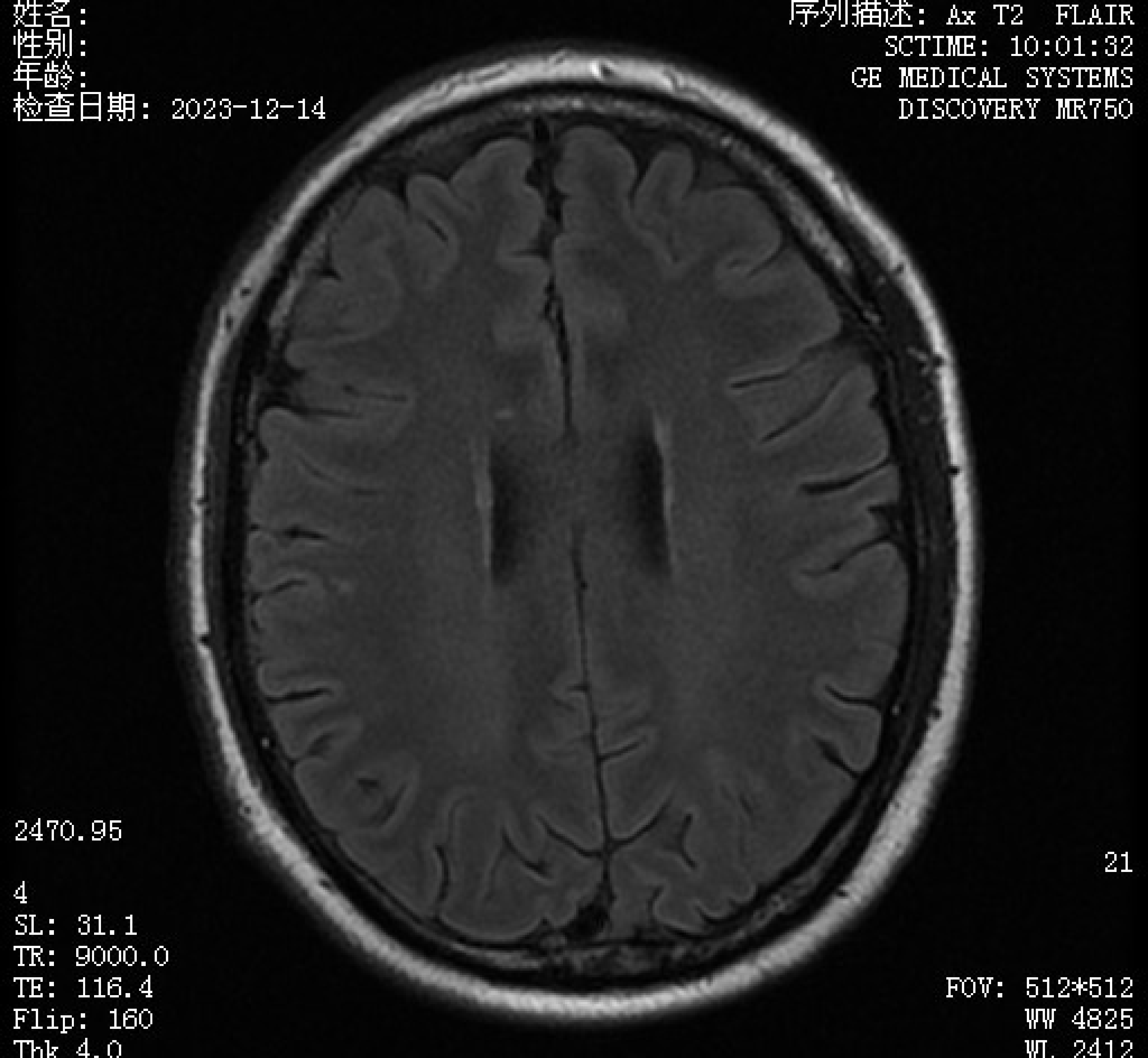

姓名:
性别:
年龄:
检查日期: 2023-12-14
序列描述: Ax T2 FLAIR
SCTIME: 10:01:32
GE MEDICAL SYSTEMS
DISCOVERY MR750
2470.95
21
4
SL: 31.1
TR: 9000.0
TE: 116.4
Flip: 160
Thk 4.0
FOV: 512*512
WW 4825
WL 2412

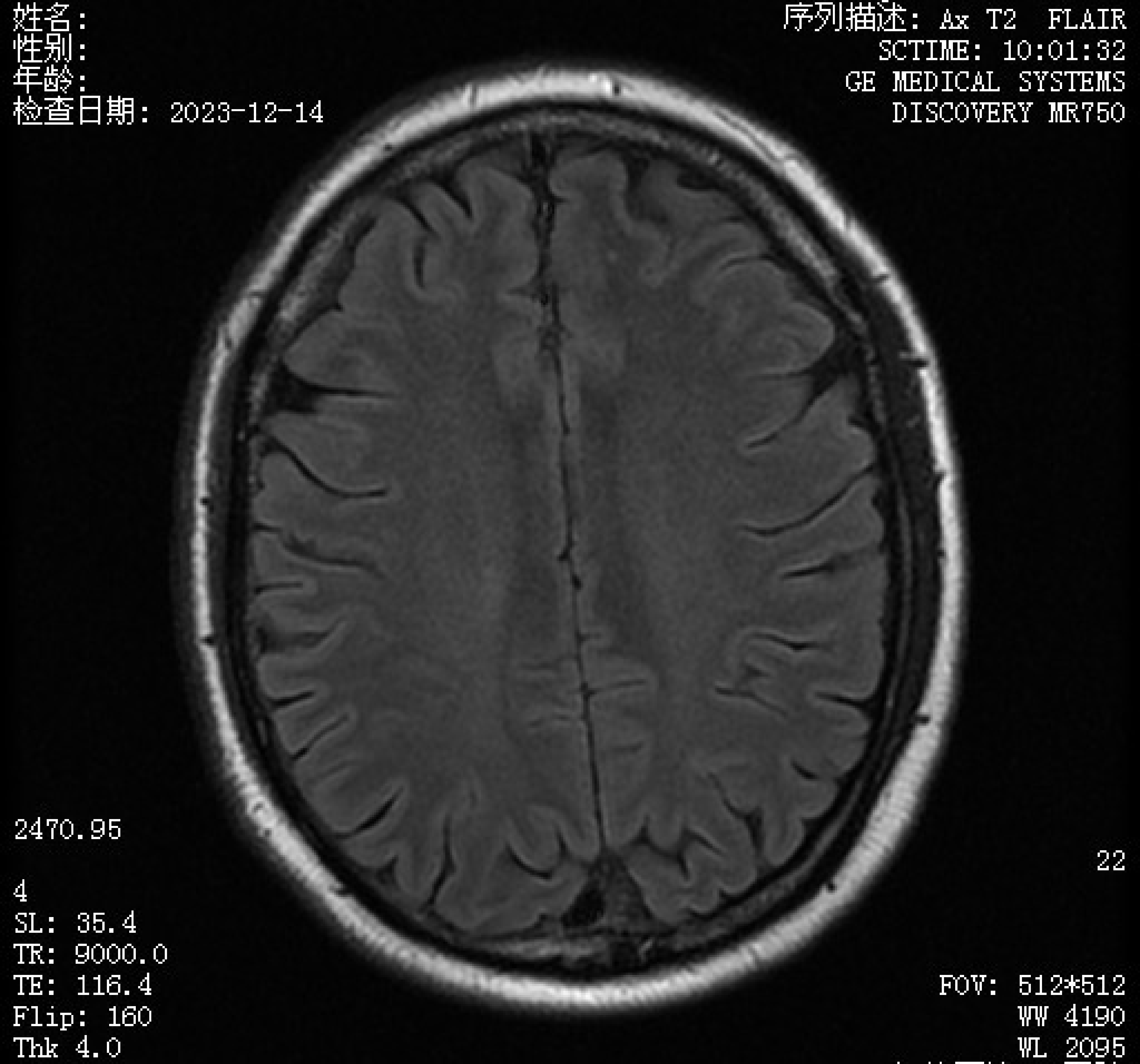
姓名:
性别:
年龄:
检查日期: 2023-12-14
序列描述: Ax T2 FLAIR
SCTIME: 10:01:32
GE MEDICAL SYSTEMS
DISCOVERY MR750
2470.95
4
SL: 35.4
TR: 9000.0
TE: 116.4
Flip: 160
Thk 4.0
22
FOV: 512*512
WW 4190
WL 2095

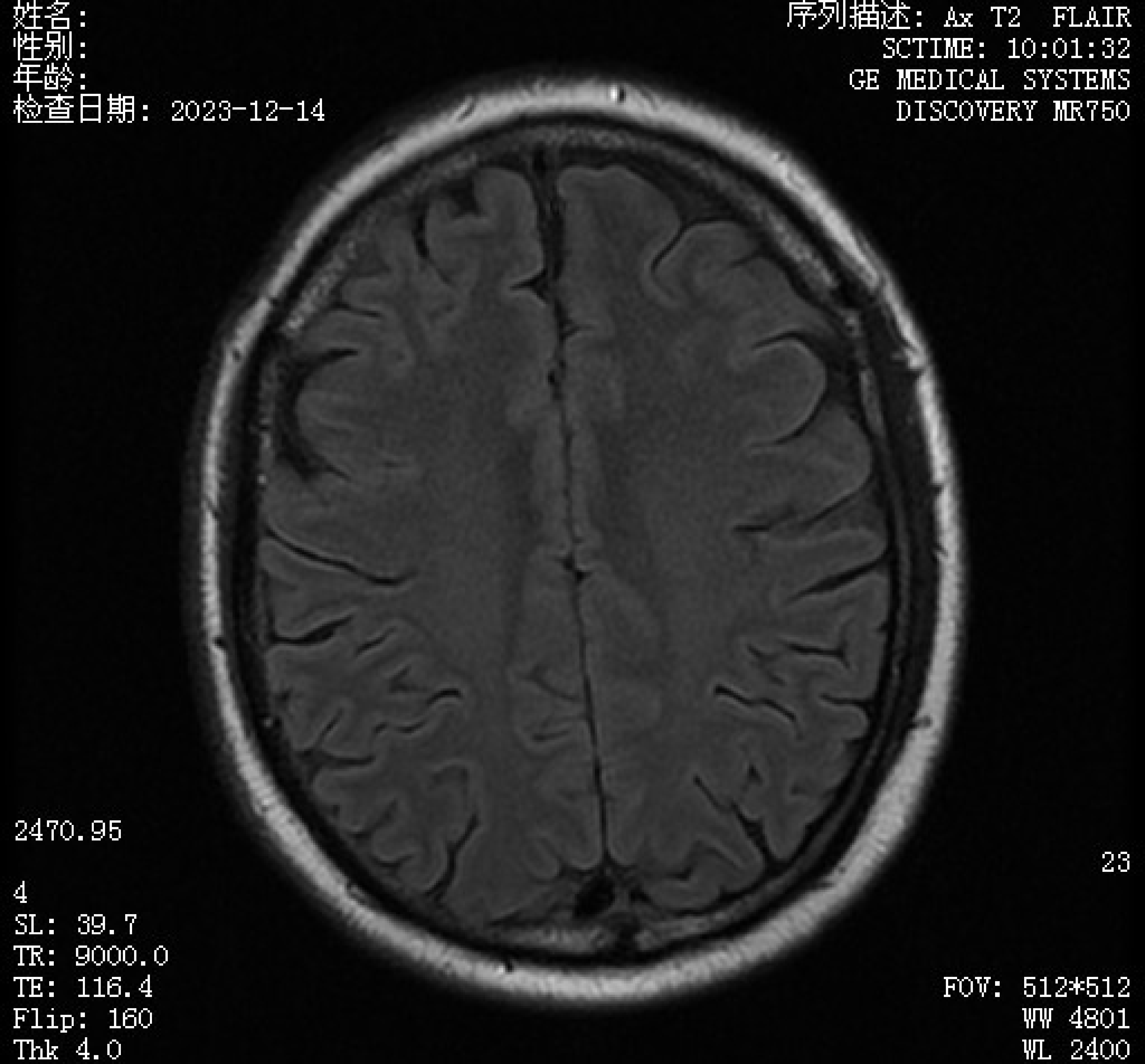
姓名:
性别:
年龄:
检查日期: 2023-12-14
序列描述: Ax T2 FLAIR
SCTIME: 10:01:32
GE MEDICAL SYSTEMS
DISCOVERY MR750
2470.95
4
SL: 39.7
TR: 9000.0
TE: 116.4
Flip: 160
Thk 4.0
23
FOV: 512*512
WW 4801
WL 2400

姓名:
性别:
年龄:
检查日期: 2023-12-14

序列描述: Ax T2 FLAIR
SCTIME: 10:01:32
GE MEDICAL SYSTEMS
DISCOVERY MR750

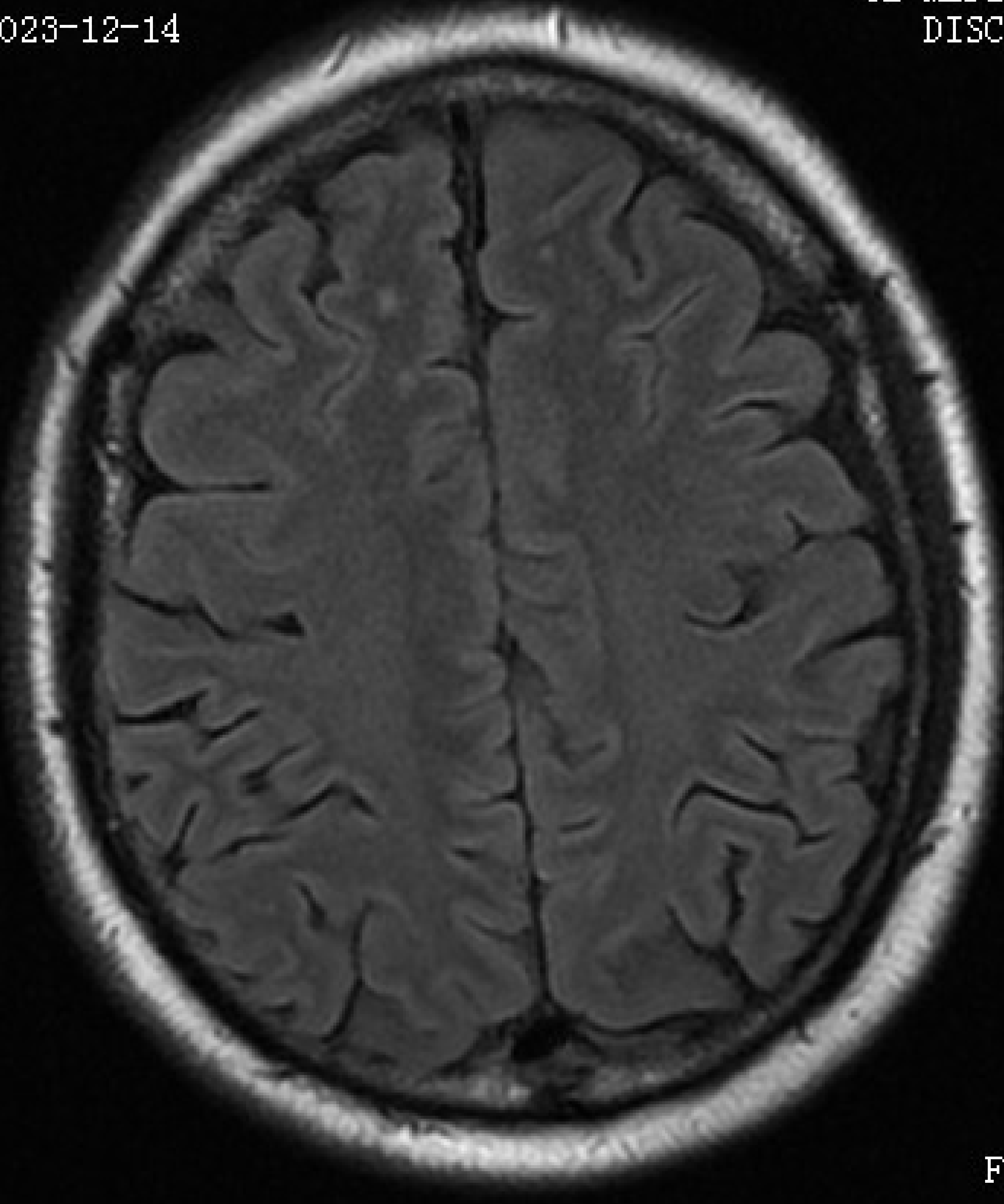

2470.95

24

4
SL: 43.9
TR: 9000.0
TE: 116.4
Flip: 160
Thk 4.0

FOV: 512*512
WW 4072
WL 2036

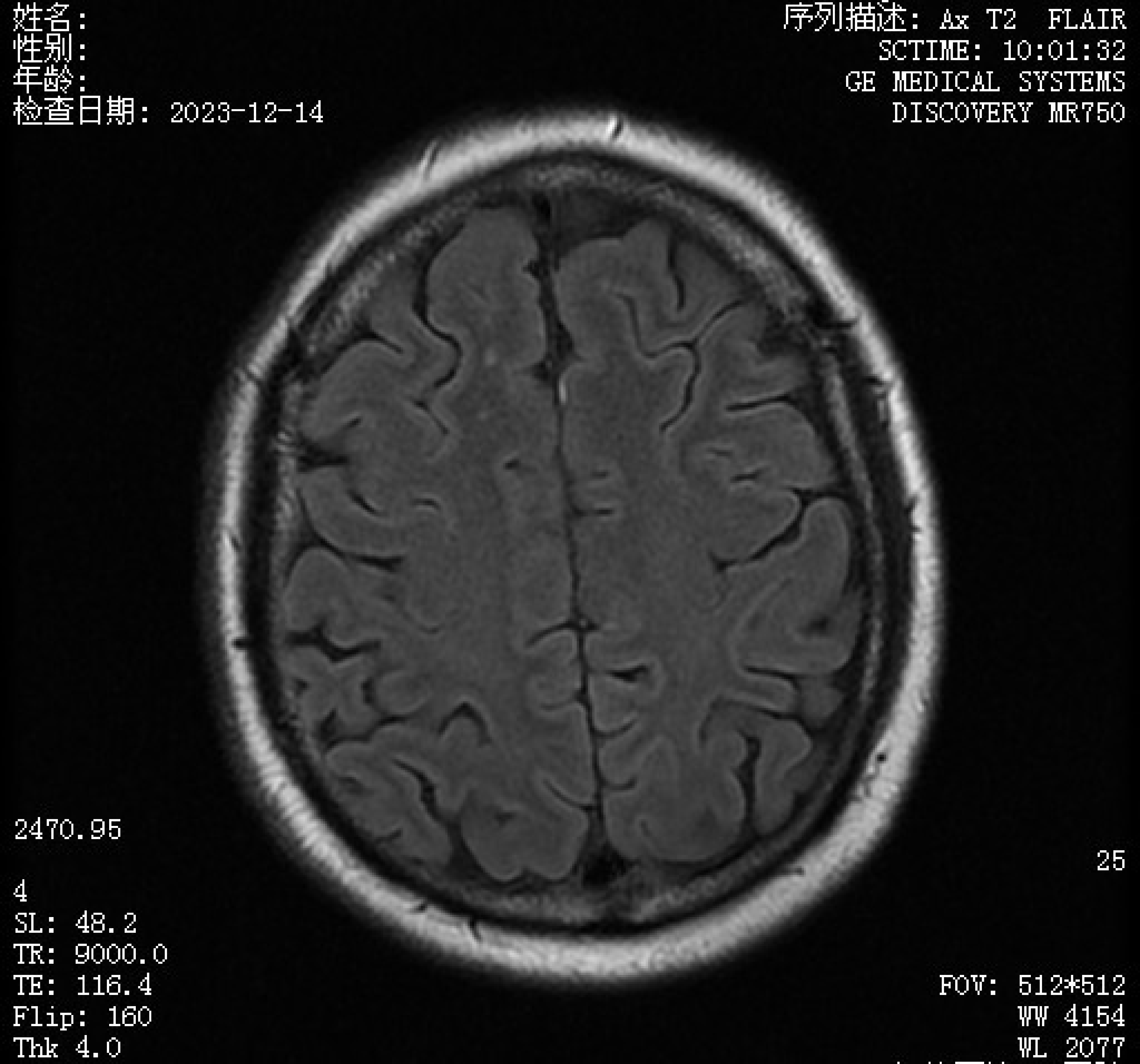
姓名:
性别:
年龄:
检查日期: 2023-12-14
序列描述: Ax T2 FLAIR
SCTIME: 10:01:32
GE MEDICAL SYSTEMS
DISCOVERY MR750
2470.95
25
4
SL: 48.2
TR: 9000.0
TE: 116.4
Flip: 160
Thk 4.0
FOV: 512*512
WW 4154
WL 2077

姓名:
性别:
年龄:
检查日期: 2023-12-14

序列描述: Ax T2 FLAIR
SCTIME: 10:01:32
GE MEDICAL SYSTEMS
DISCOVERY MR750

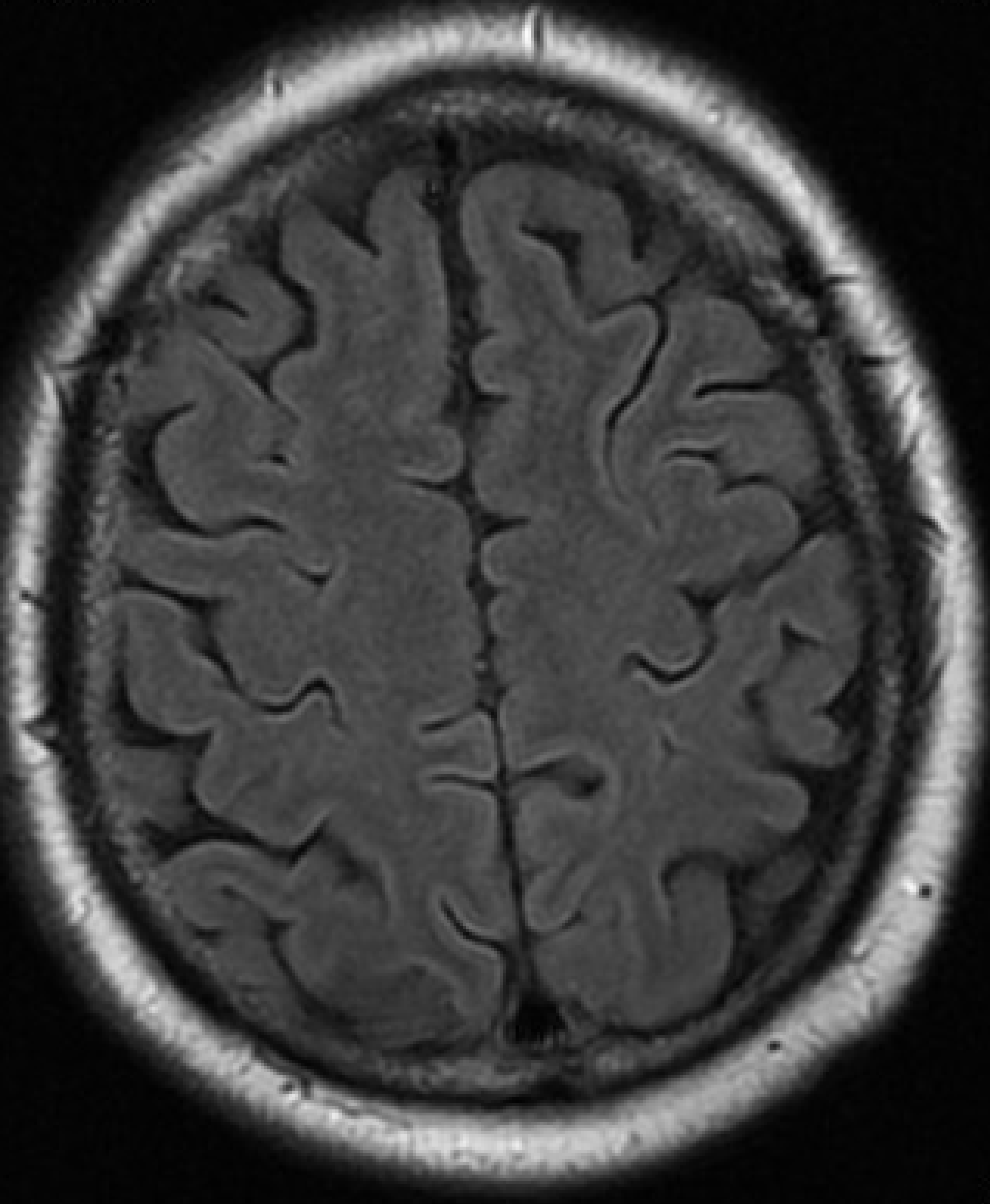

2470.95

26

4
SL: 52.5
TR: 9000.0
TE: 116.4
Flip: 160
Thk 4.0

FOV: 512*512
WW 3895
WL 1947

姓名:
性别:
年龄:
检查日期: 2023-12-14

序列描述: Ax T2 FLAIR
SCTIME: 10:01:32
GE MEDICAL SYSTEMS
DISCOVERY MR750

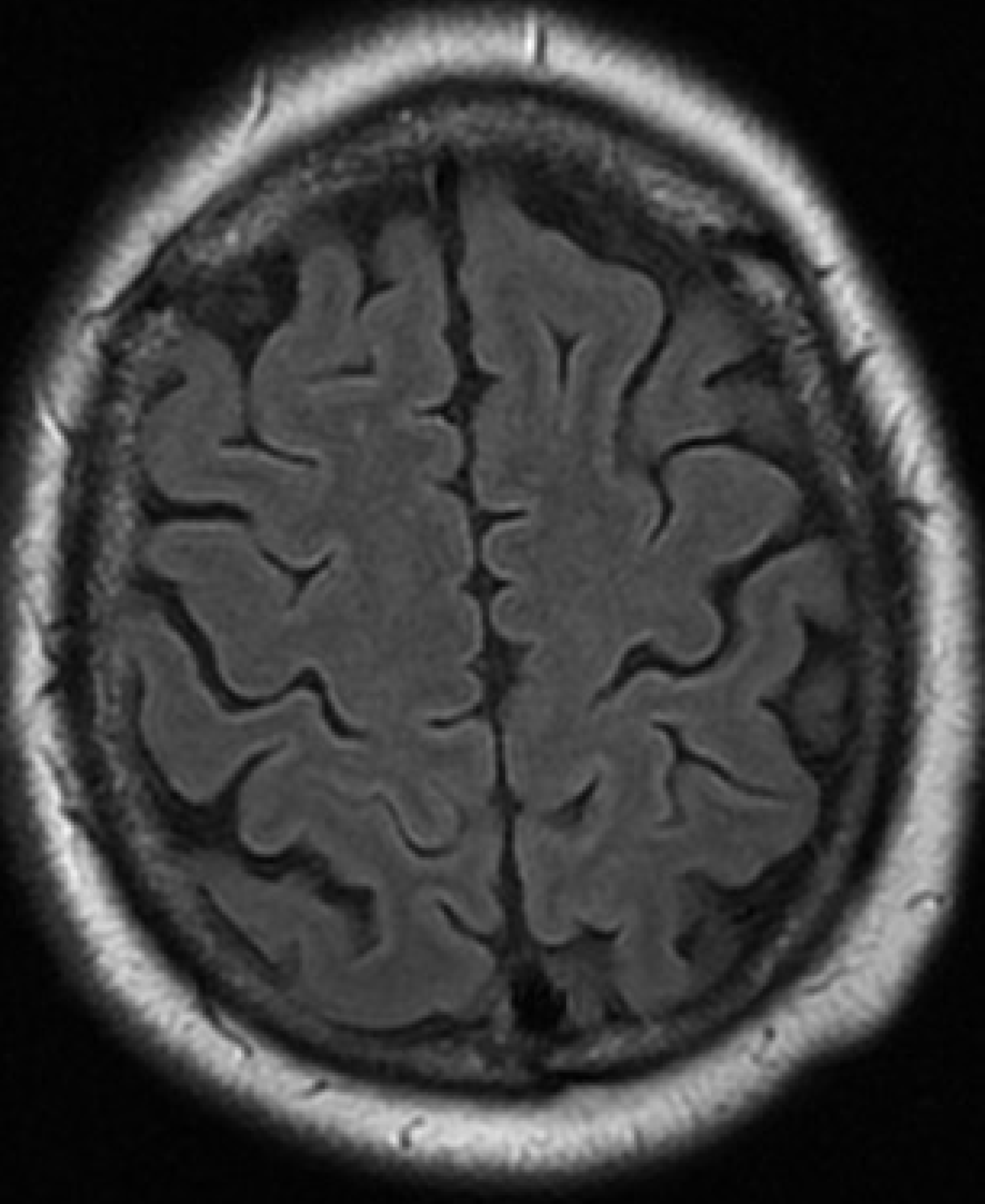

2470.95

27

4
SL: 56.8
TR: 9000.0
TE: 116.4
Flip: 160
Thk 4.0

FOV: 512*512
WW 3955
WL 1977

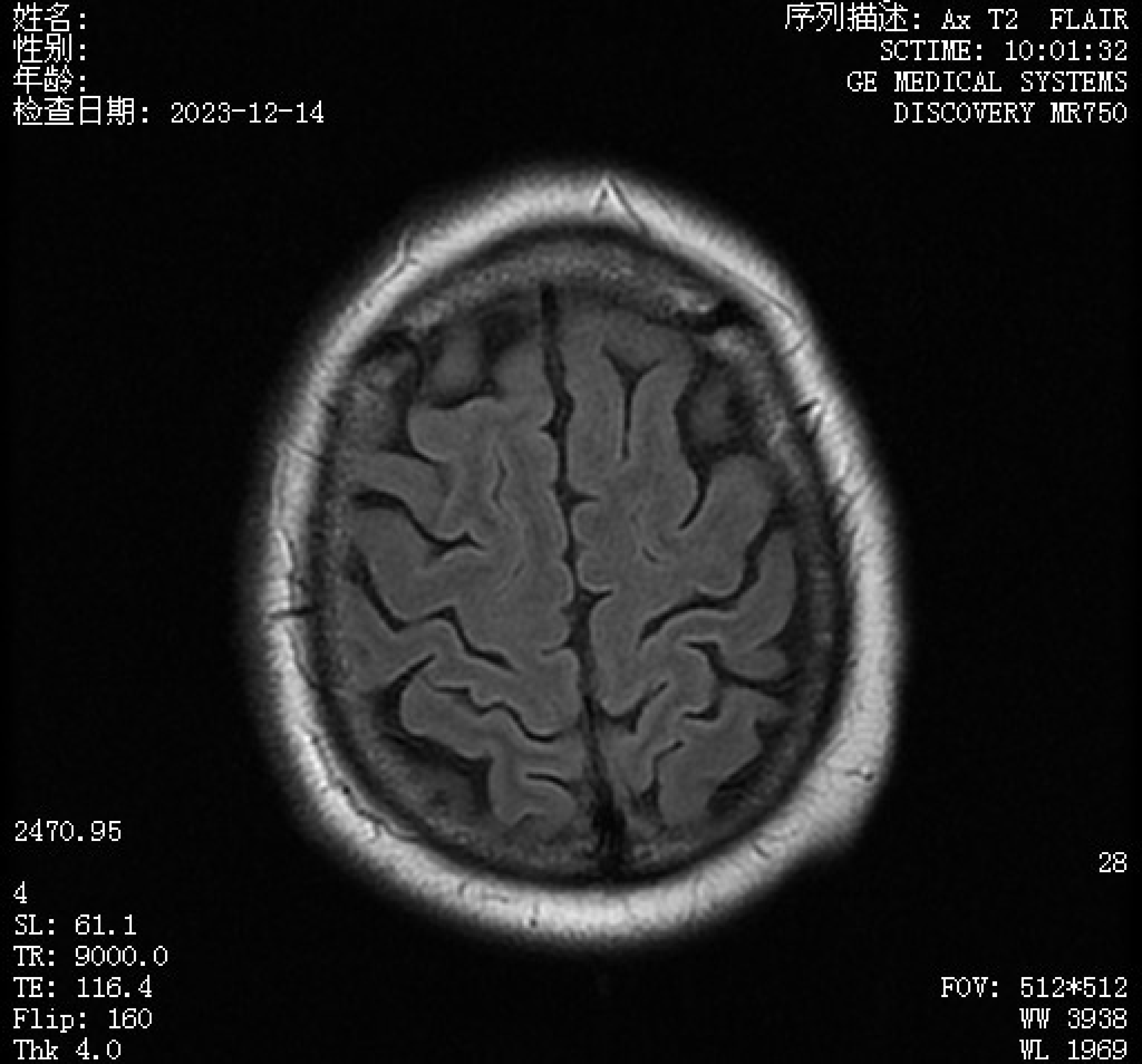
姓名:
性别:
年龄:
检查日期: 2023-12-14
序列描述: Ax T2 FLAIR
SCTIME: 10:01:32
GE MEDICAL SYSTEMS
DISCOVERY MR750
2470.95
28
4
SL: 61.1
TR: 9000.0
TE: 116.4
Flip: 160
Thk 4.0
FOV: 512*512
WW 3938
WL 1969

姓名:
性别:
年龄:
检查日期: 2023-12-14

序列描述: Ax T2 FLAIR
SCTIME: 10:01:32
GE MEDICAL SYSTEMS
DISCOVERY MR750

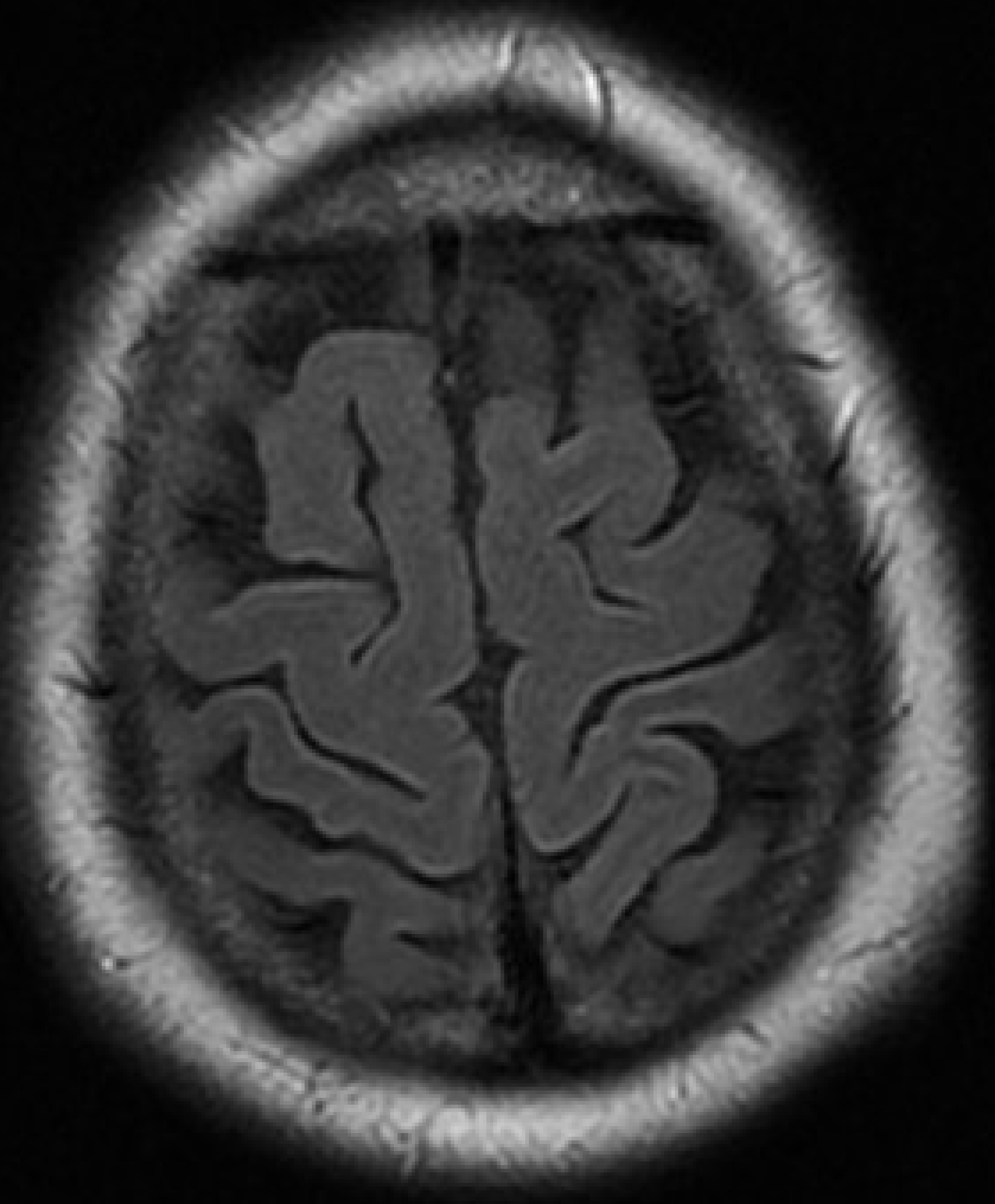

2470.95

29

4
SL: 65.3
TR: 9000.0
TE: 116.4
Flip: 160
Thk 4.0

FOV: 512*512
WW 4497
WL 2248

姓名:
性别:
年龄:
检查日期: 2023-12-14

序列描述: Ax T2 FLAIR
SCTIME: 10:01:32
GE MEDICAL SYSTEMS
DISCOVERY MR750

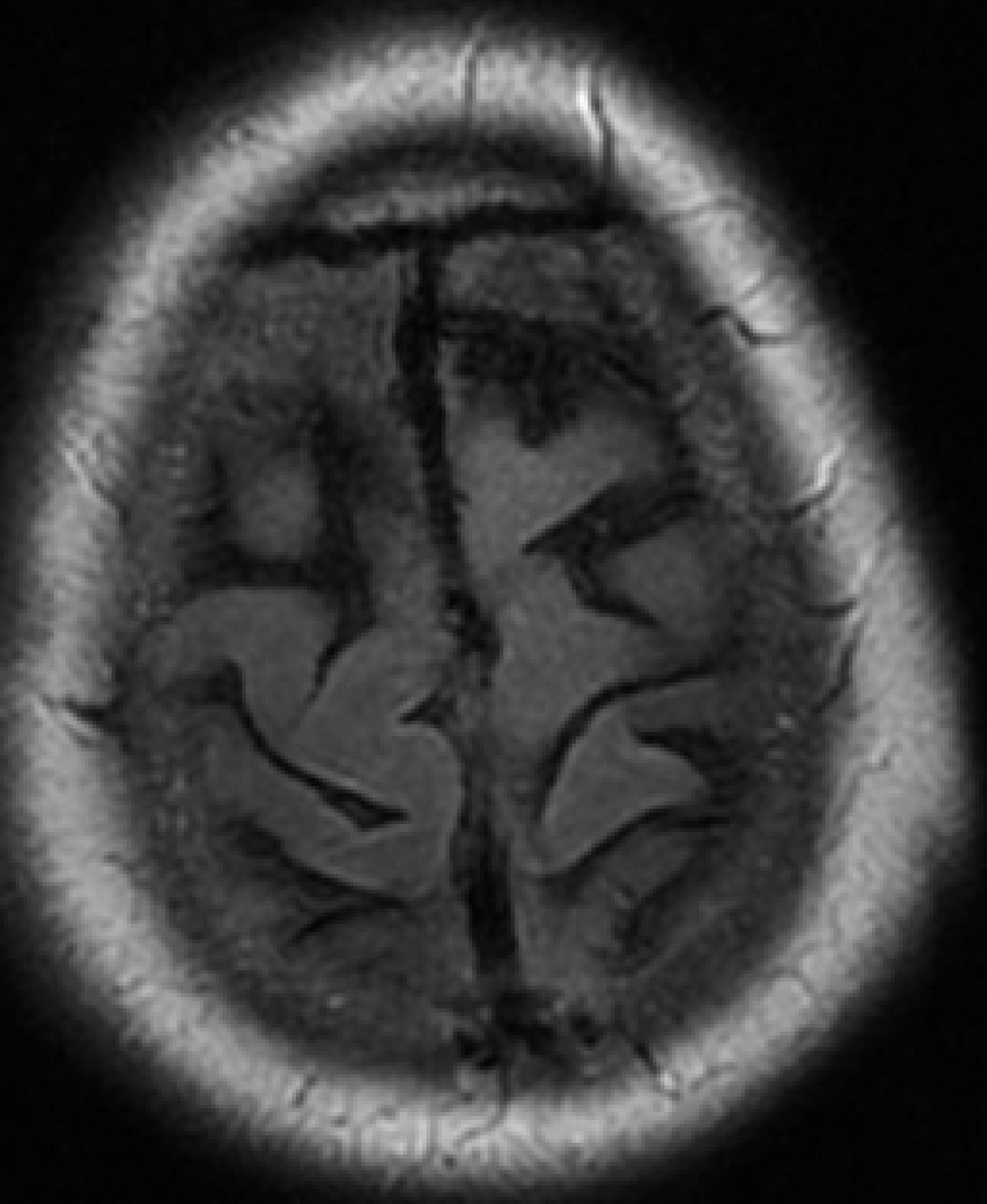

2470.95

30

4
SL: 69.6
TR: 9000.0
TE: 116.4
Flip: 160
Thk 4.0

FOV: 512*512
WW 4513
WL 2256
