# Supplementary material for: Sustained complete response to TMEp-CI-M platform in refractory small-cell lung cancer with brainstem metastasis: a case report with over 20 months of disease-free survival
Source: Front Immunol. 2026 Jun 1;17:1807865. doi: 10.3389/fimmu.2026.1807865 (PMC13265516; doi:10.3389/fimmu.2026.1807865)
Supplement: Supplementary Figure 5 — GTV delineation of brainstem lesion (The GTV is delineated by the purple line). [file DataSheet3.pdf]

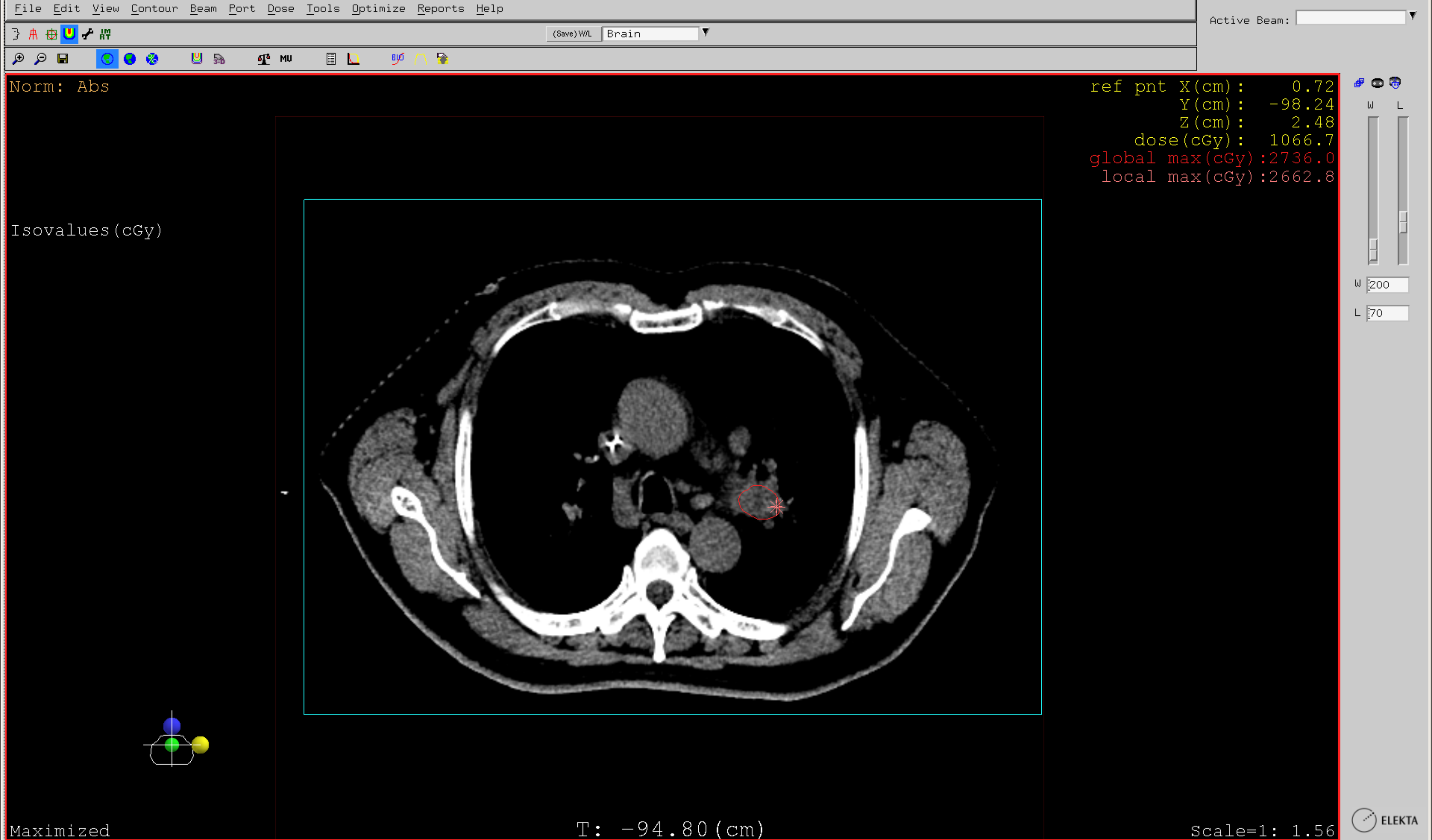

File Edit View Contour Beam Port Dose Tools Optimize Reports Help
Active Beam:
(Save) W/L
Brain
MU
Norm: Abs
ref pnt X(cm): 0.72
Y(cm): -98.24
Z(cm): 2.48
dose(cGy): 1066.7
global max(cGy):2736.0
local max(cGy):2662.8
Isovalues(cGy)
W
L
W 200
L 70
Maximized
T: -94.80(cm)
Scale=1: 1.56
ELEKTA

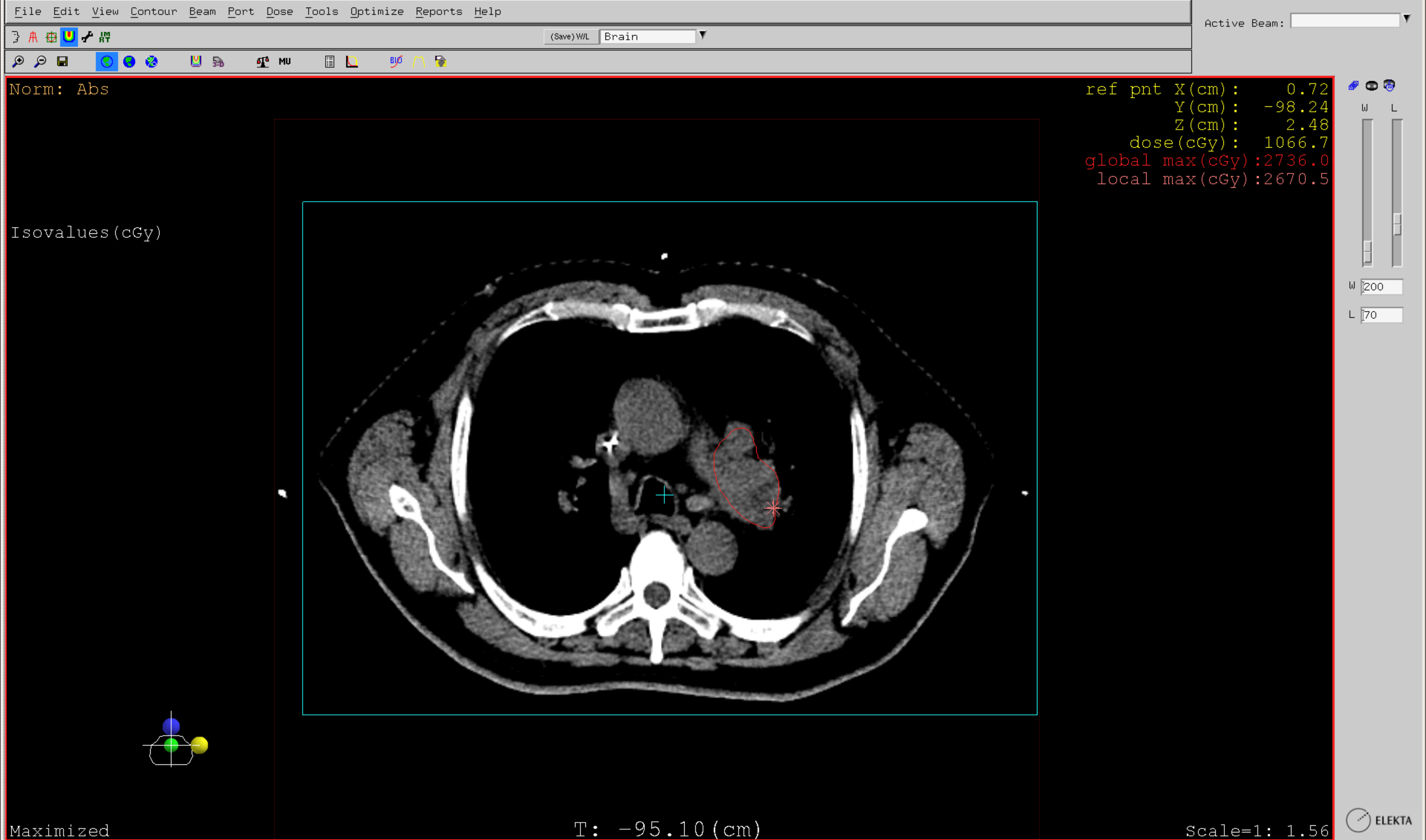
File Edit View Contour Beam Port Dose Tools Optimize Reports Help
Active Beam:
(Save) W/L Brain
MU
Norm: Abs
ref pnt X(cm): 0.72
Y(cm): -98.24
Z(cm): 2.48
dose(cGy): 1066.7
global max(cGy):2736.0
local max(cGy):2670.5
Isovalues(cGy)
W L
W 200
L 70
Maximized
T: -95.10(cm)
Scale=1: 1.56
ELEKTA

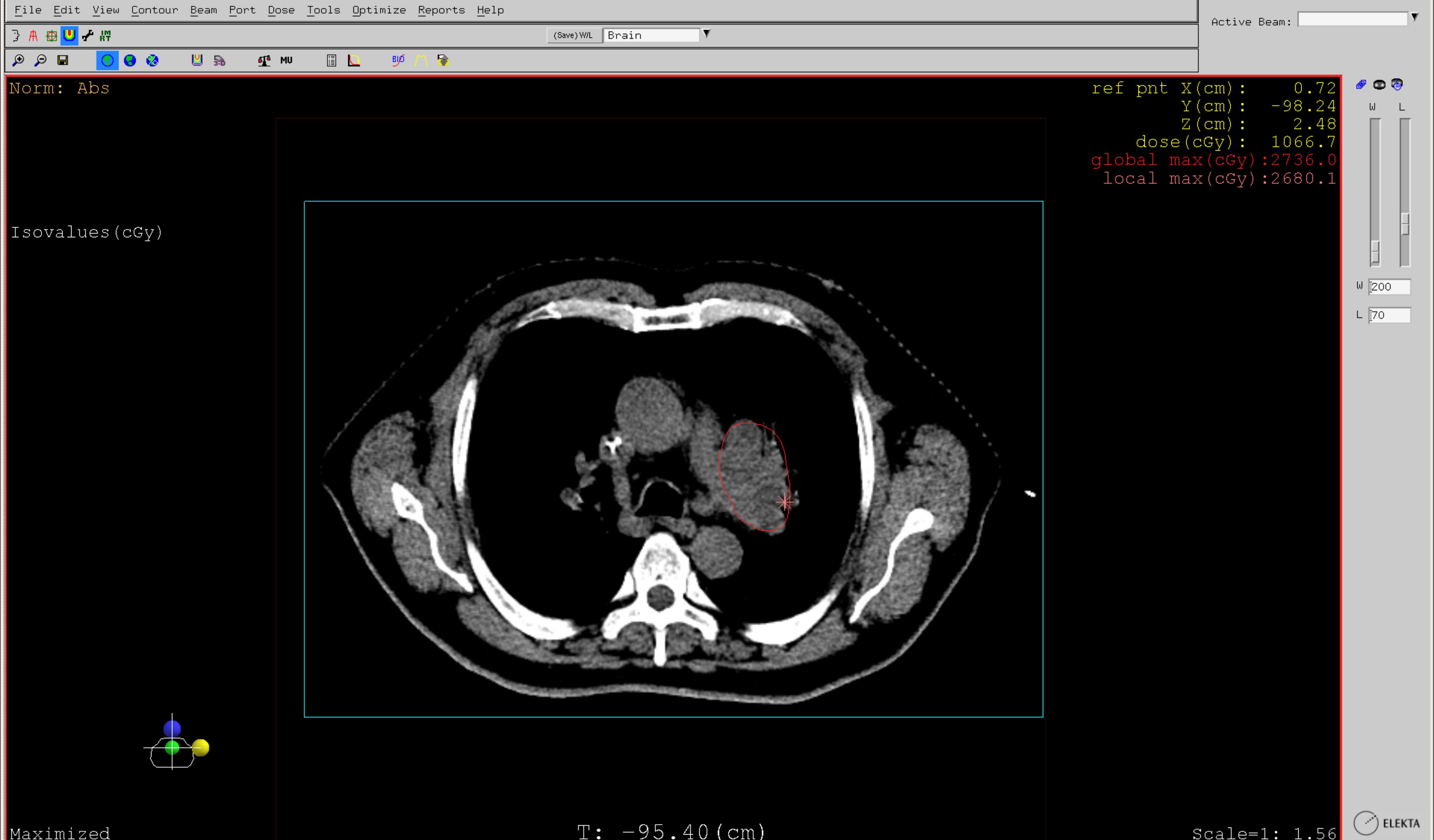
File Edit View Contour Beam Port Dose Tools Optimize Reports Help
Active Beam:
(Save) W/L
Brain
MU
Norm: Abs
ref pnt X(cm): 0.72
Y(cm): -98.24
Z(cm): 2.48
dose(cGy): 1066.7
global max(cGy):2736.0
local max(cGy):2680.1
Isovalues(cGy)
W
L
W 200
L 70
Maximized
T: -95.40(cm)
Scale=1: 1.56
ELEKTA

File Edit View Contour Beam Port Dose Tools Optimize Reports Help

Active Beam:

(Save) W/L Brain

MU

Norm: Abs

ref pnt X(cm): 0.72
Y(cm): -98.24
Z(cm): 2.48
dose(cGy): 1066.7
global max(cGy):2736.0
local max(cGy):2708.4

W L

W 200

L 70

Isovalues(cGy)

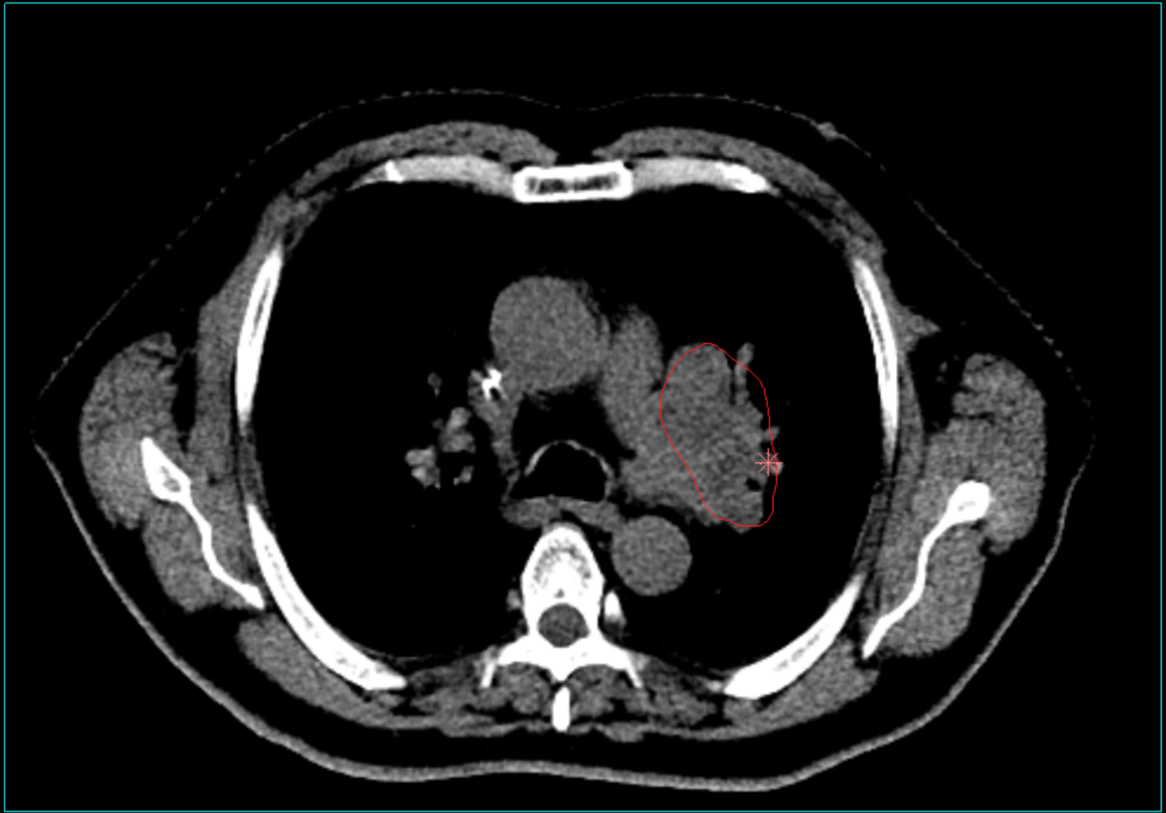

Maximized

T: -95.70(cm)

Scale=1: 1.56

ELEKTA

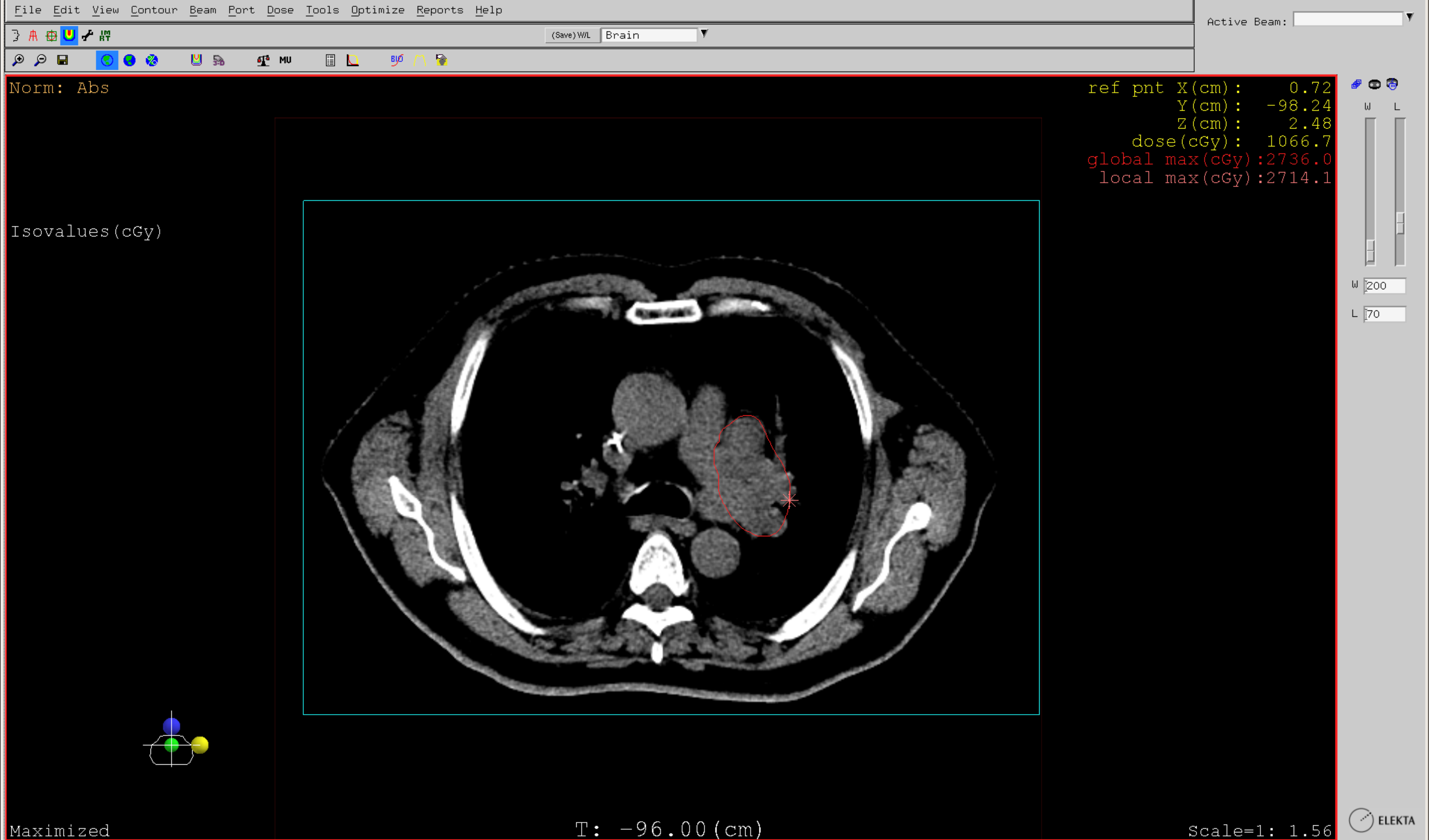
File Edit View Contour Beam Port Dose Tools Optimize Reports Help
Active Beam:
(Save) W/L
Brain
MU
Norm: Abs
ref pnt X(cm): 0.72
Y(cm): -98.24
Z(cm): 2.48
dose(cGy): 1066.7
global max(cGy):2736.0
local max(cGy):2714.1
W
L
W 200
L 70
Isovalues(cGy)
Maximized
T: -96.00(cm)
Scale=1: 1.56
ELEKTA

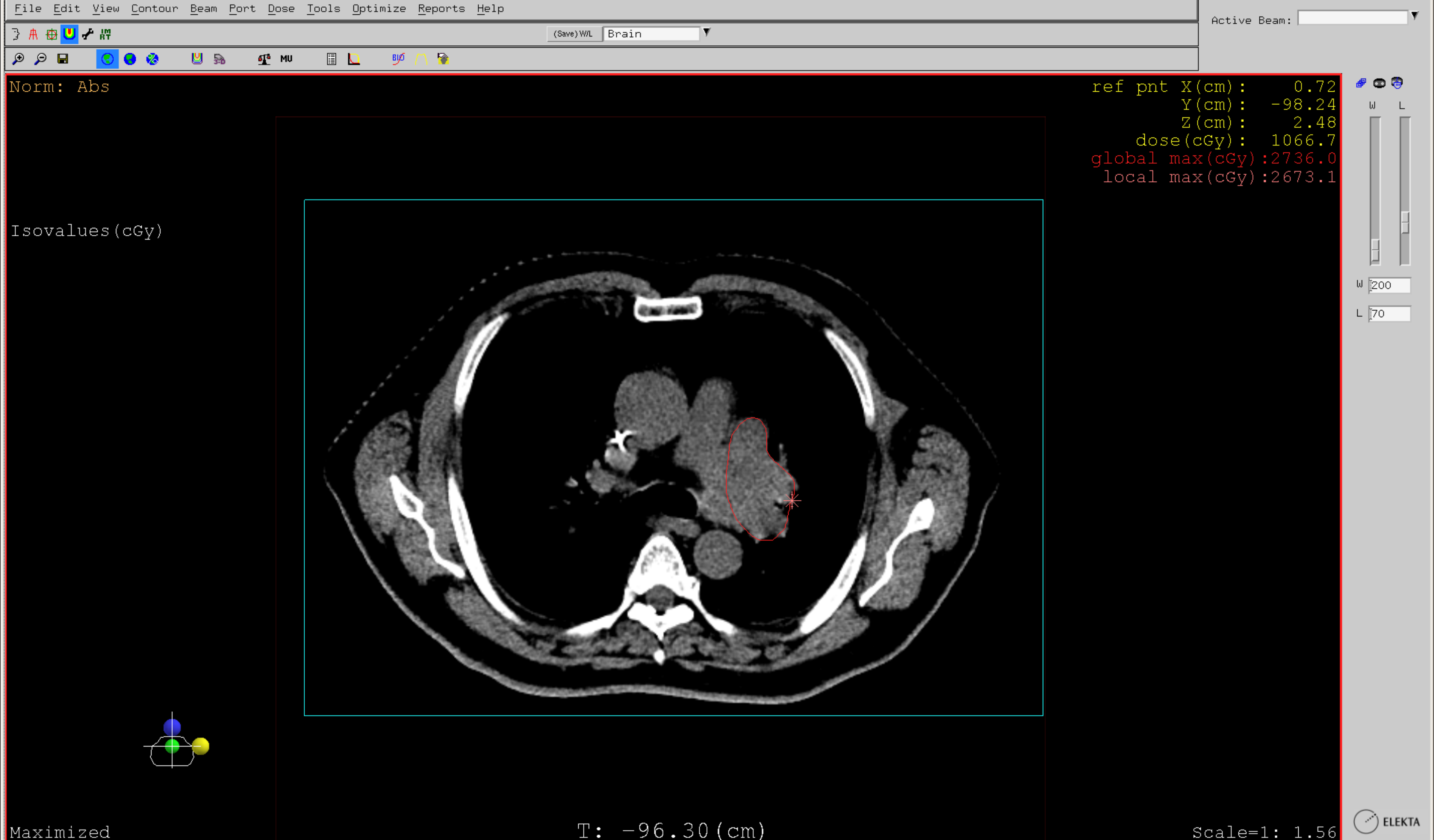
File Edit View Contour Beam Port Dose Tools Optimize Reports Help
Active Beam:
(Save) W/L
Brain
MU
Norm: Abs
ref pnt X(cm): 0.72
Y(cm): -98.24
Z(cm): 2.48
dose(cGy): 1066.7
global max(cGy):2736.0
local max(cGy):2673.1
Isovalues(cGy)
W
L
W 200
L 70
Maximized
T: -96.30(cm)
Scale=1: 1.56
ELEKTA

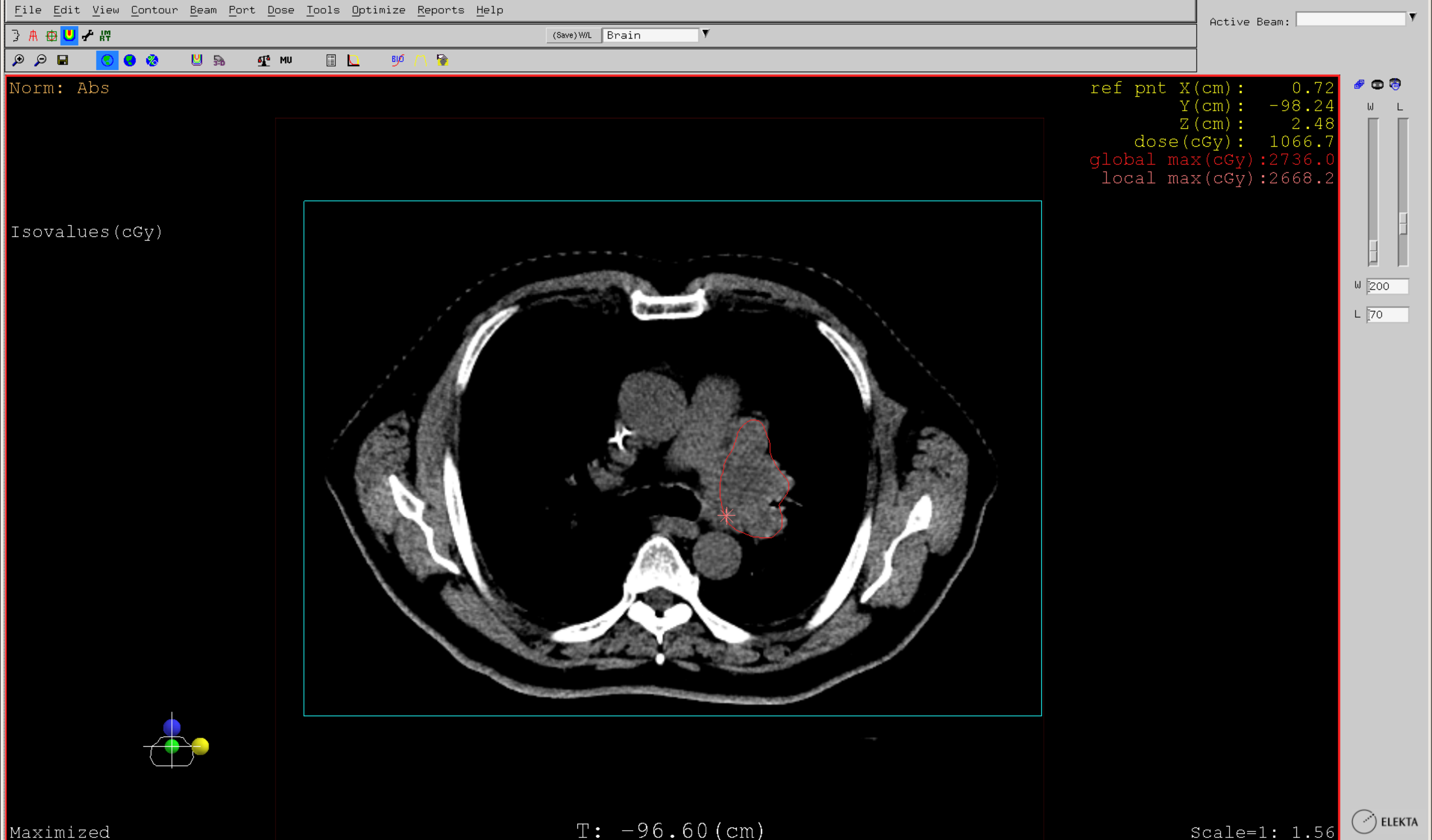
File Edit View Contour Beam Port Dose Tools Optimize Reports Help
Active Beam:
(Save) W/L
Brain
MU
Norm: Abs
ref pnt X(cm): 0.72
Y(cm): -98.24
Z(cm): 2.48
dose(cGy): 1066.7
global max(cGy):2736.0
local max(cGy):2668.2
Isovalues(cGy)
W
L
W 200
L 70
Maximized
T: -96.60(cm)
Scale=1: 1.56
ELEKTA

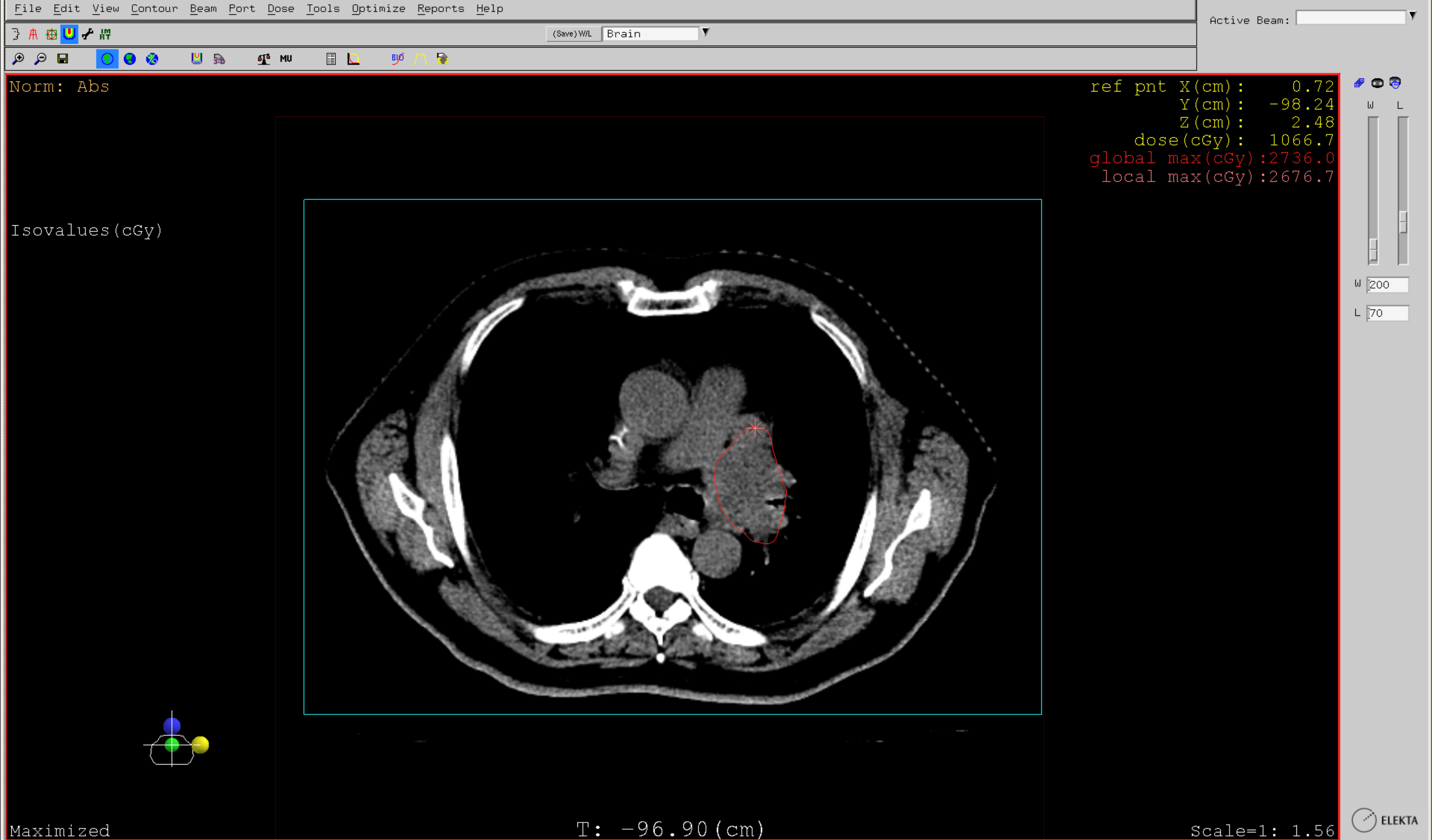
File Edit View Contour Beam Port Dose Tools Optimize Reports Help
Active Beam:
(Save) W/L
Brain
MU
Norm: Abs
ref pnt X(cm): 0.72
Y(cm): -98.24
Z(cm): 2.48
dose(cGy): 1066.7
global max(cGy):2736.0
local max(cGy):2676.7
Isovalues(cGy)
W
L
W 200
L 70
Maximized
T: -96.90(cm)
Scale=1: 1.56
ELEKTA

File Edit View Contour Beam Port Dose Tools Optimize Reports Help

Active Beam:

(Save) W/L Brain

MU

Norm: Abs

ref pnt X(cm): 0.72
Y(cm): -98.24
Z(cm): 2.48
dose(cGy): 1066.7
global max(cGy):2736.0
local max(cGy):2676.2

W L

W 200

L 70

Isovalues(cGy)

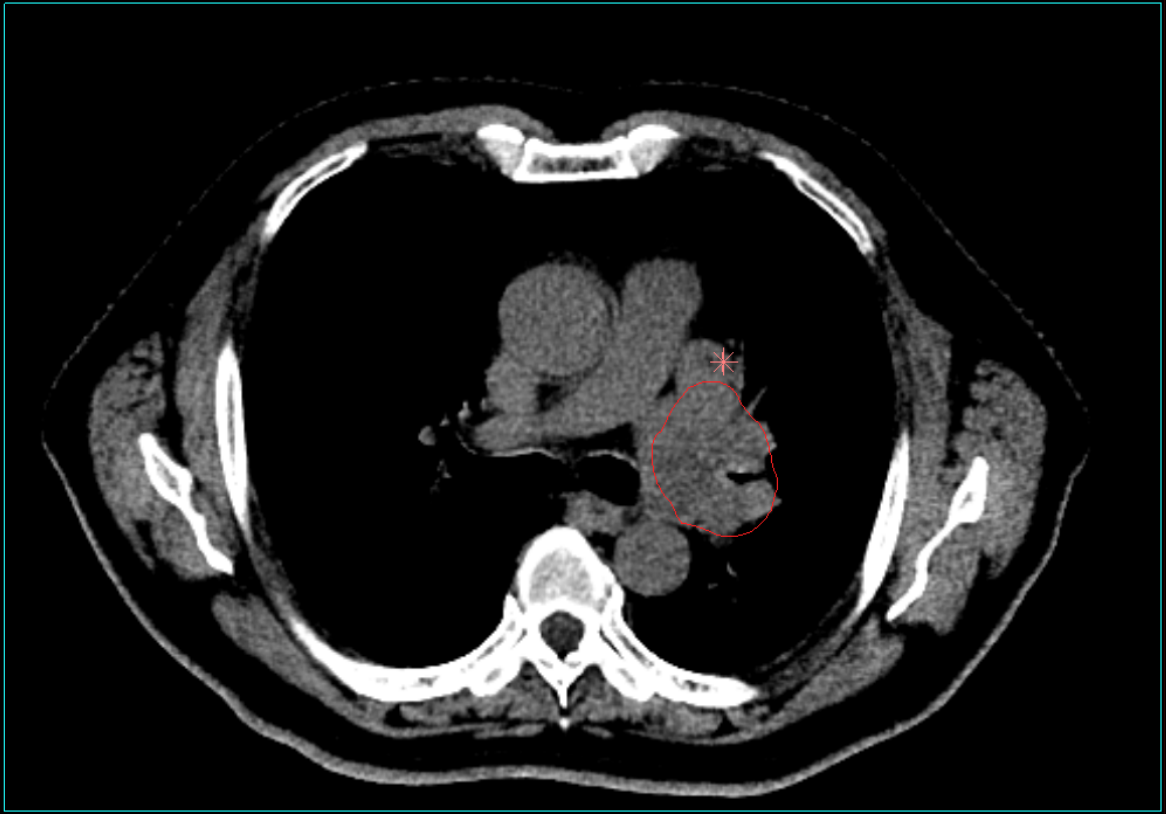

Maximized

T: -97.20(cm)

Scale=1: 1.56

ELEKTA

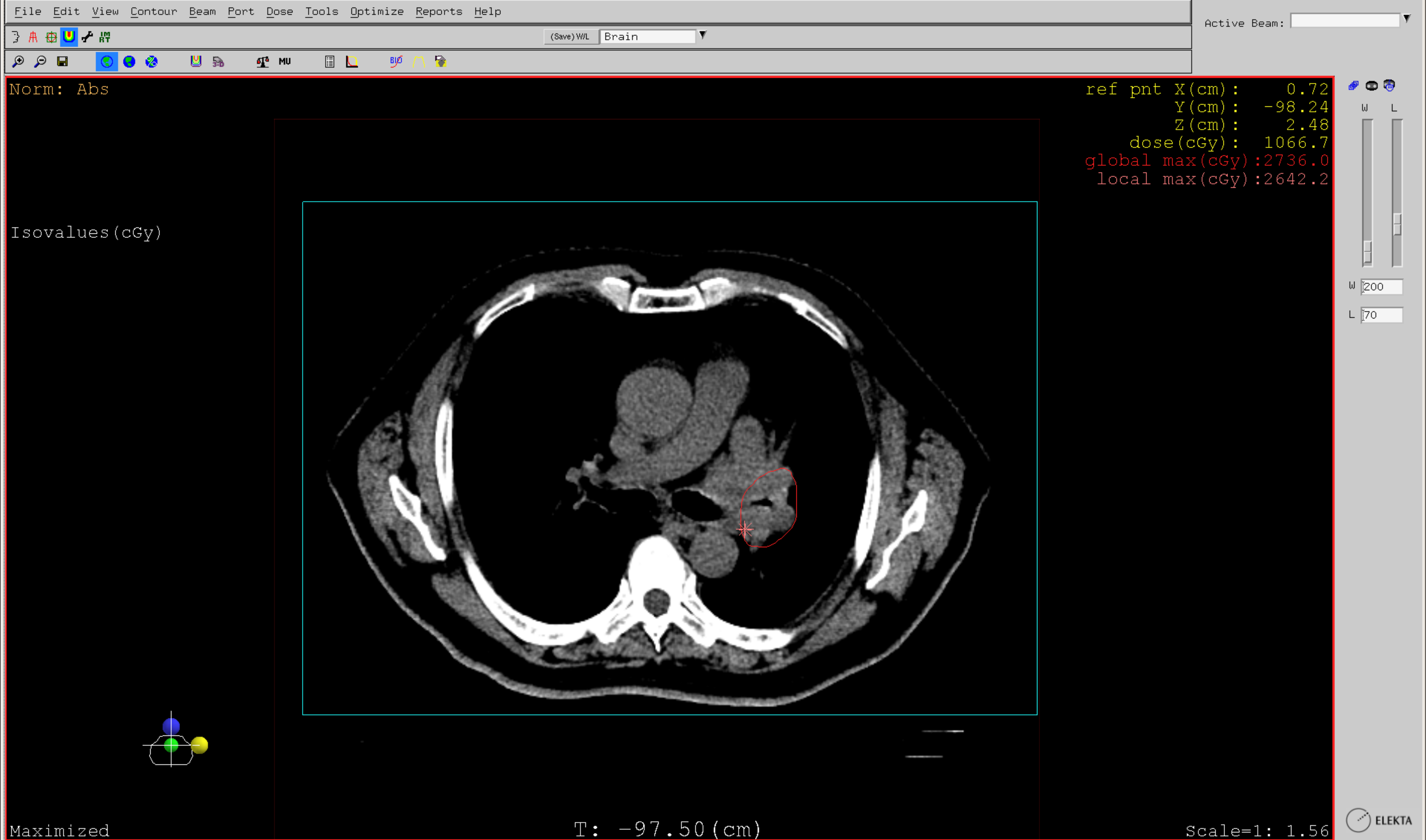
File Edit View Contour Beam Port Dose Tools Optimize Reports Help
Active Beam:
(Save) W/L
Brain
MU
Norm: Abs
ref pnt X(cm): 0.72
Y(cm): -98.24
Z(cm): 2.48
dose(cGy): 1066.7
global max(cGy):2736.0
local max(cGy):2642.2
Isovalues(cGy)
W
L
W 200
L 70
Maximized
T: -97.50(cm)
Scale=1: 1.56
ELEKTA

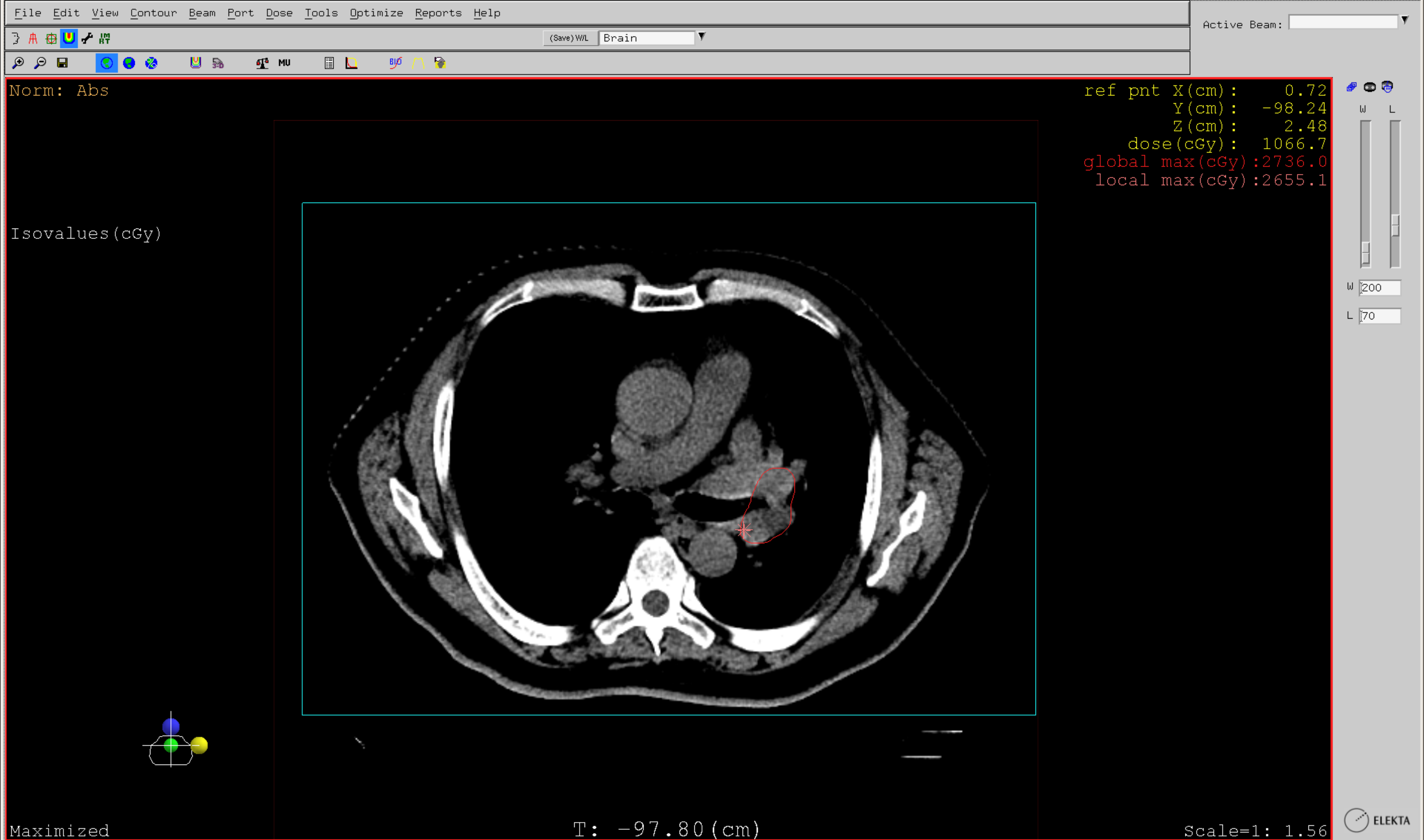
File Edit View Contour Beam Port Dose Tools Optimize Reports Help
Active Beam:
(Save) W/L
Brain
MU
Norm: Abs
ref pnt X(cm): 0.72
Y(cm): -98.24
Z(cm): 2.48
dose(cGy): 1066.7
global max(cGy):2736.0
local max(cGy):2655.1
W
L
W 200
L 70
Isovalues(cGy)
Maximized
T: -97.80(cm)
Scale=1: 1.56
ELEKTA

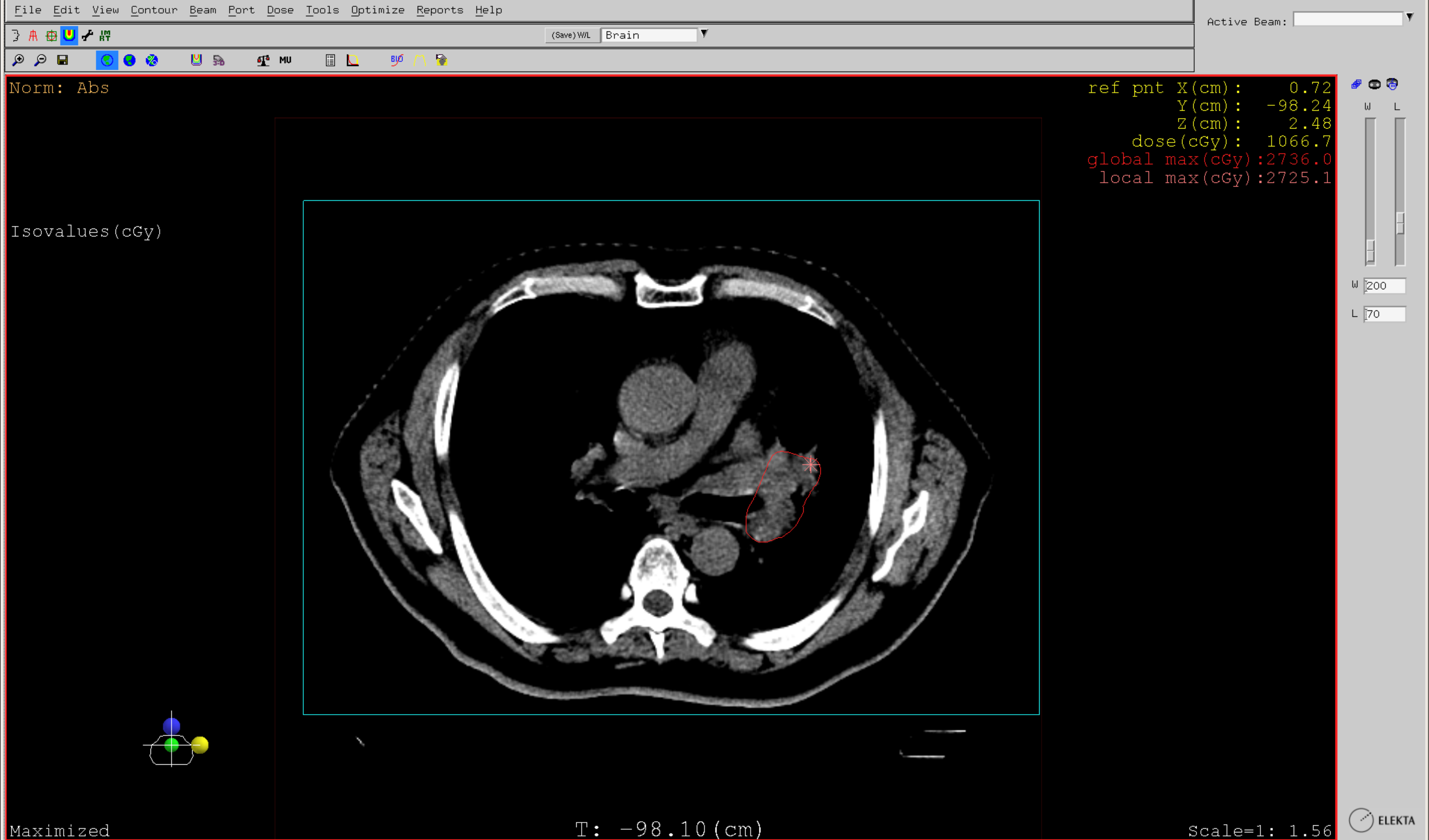

File Edit View Contour Beam Port Dose Tools Optimize Reports Help
(Save) W/L
Brain
Active Beam:
MU
Norm: Abs
ref pnt X(cm): 0.72
Y(cm): -98.24
Z(cm): 2.48
dose(cGy): 1066.7
global max(cGy):2736.0
local max(cGy):2725.1
Isovalues(cGy)
W
L
W 200
L 70
Maximized
T: -98.10(cm)
Scale=1: 1.56
ELEKTA

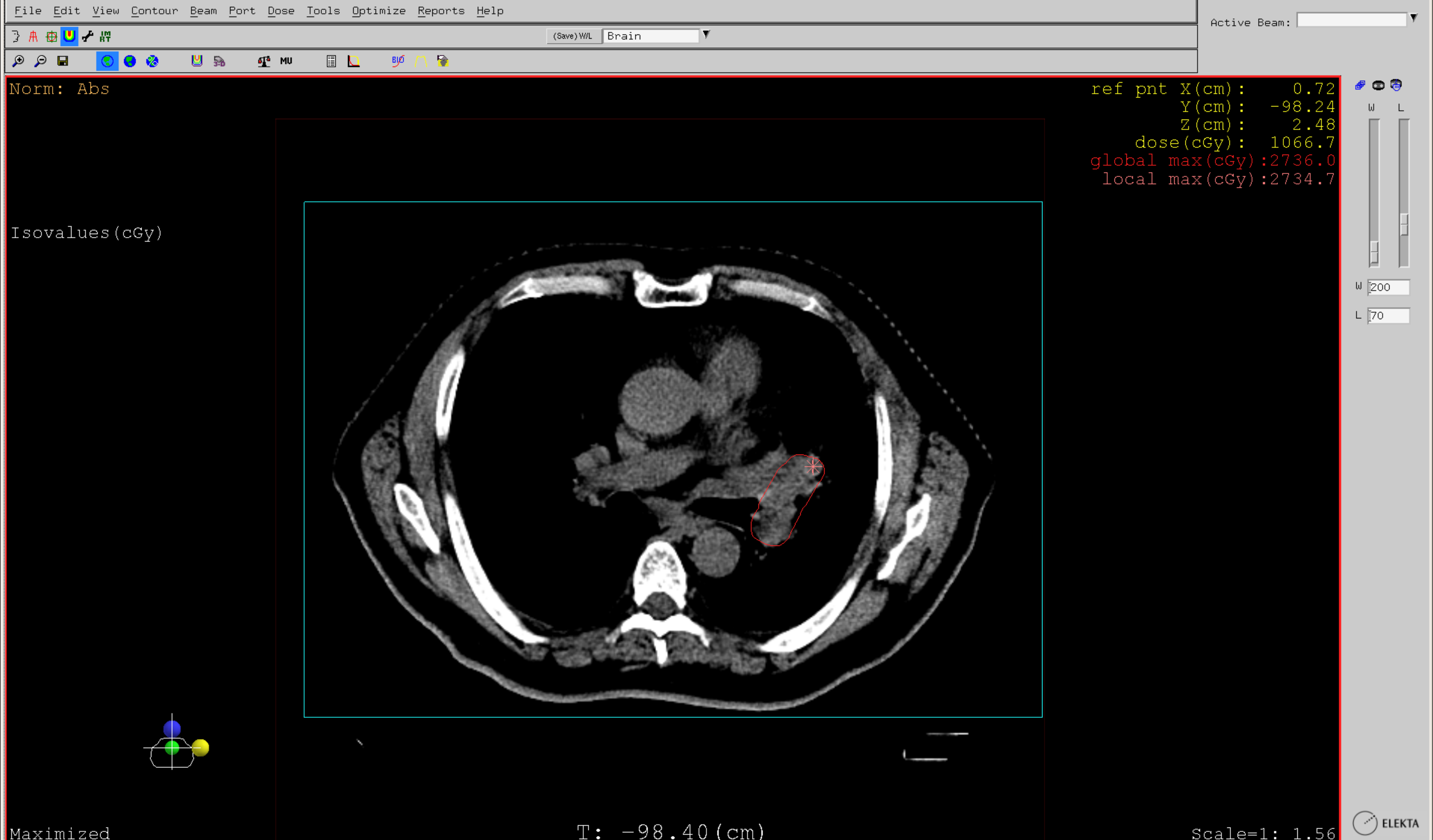
File Edit View Contour Beam Port Dose Tools Optimize Reports Help
Active Beam:
(Save) W/L
Brain
MU
Norm: Abs
ref pnt X(cm): 0.72
Y(cm): -98.24
Z(cm): 2.48
dose(cGy): 1066.7
global max(cGy):2736.0
local max(cGy):2734.7
Isovalues(cGy)
W
L
W 200
L 70
Maximized
T: -98.40(cm)
Scale=1: 1.56
ELEKTA
