# Supplementary material for: Sustained complete response to TMEp-CI-M platform in refractory small-cell lung cancer with brainstem metastasis: a case report with over 20 months of disease-free survival
Source: Front Immunol. 2026 Jun 1;17:1807865. doi: 10.3389/fimmu.2026.1807865 (PMC13265516; doi:10.3389/fimmu.2026.1807865)
Supplement: Supplementary Figure 6 — Serial contrast-enhanced chest CT and brain MRI, assessed according to RECIST version 1.1 and RANO-BM criteria, respectively, demonstrated sustained complete remission. At baseline, the extracranial target lesion consisted of a left hilar mass measuring 6.8×4.8 cm, while the intracranial target lesion was a brainstem metastasis measuring 1.3×0.6 cm. Following two treatment cycles (week 6), the extracranial lesion achieved a partial response (PR), subsequently regressing to a stable scar-like residual lesion accompanied by normalization of serum ProGRP levels; this finding was retrospectively reclassified as a complete response (CR). Sustained extracranial CR was maintained from cycles 4–6 through the end of treatment. Intracranial CR was achieved at the 3-month evaluation and persisted through the final follow-up assessment at 20 months. [file DataSheet4.pdf]

File Edit View Contour Beam Port Dose Tools Optimize Reports Help

Active Beam:

(Save) W/L Brain

MU

Norm: Abs

ref pnt X(cm): -0.12
Y(cm): -80.44
Z(cm): 2.65
dose(cGy): 121.4
global max(cGy):2260.4
local max(cGy):2162.8

W L

W 200

L 70

Isovalues(cGy)

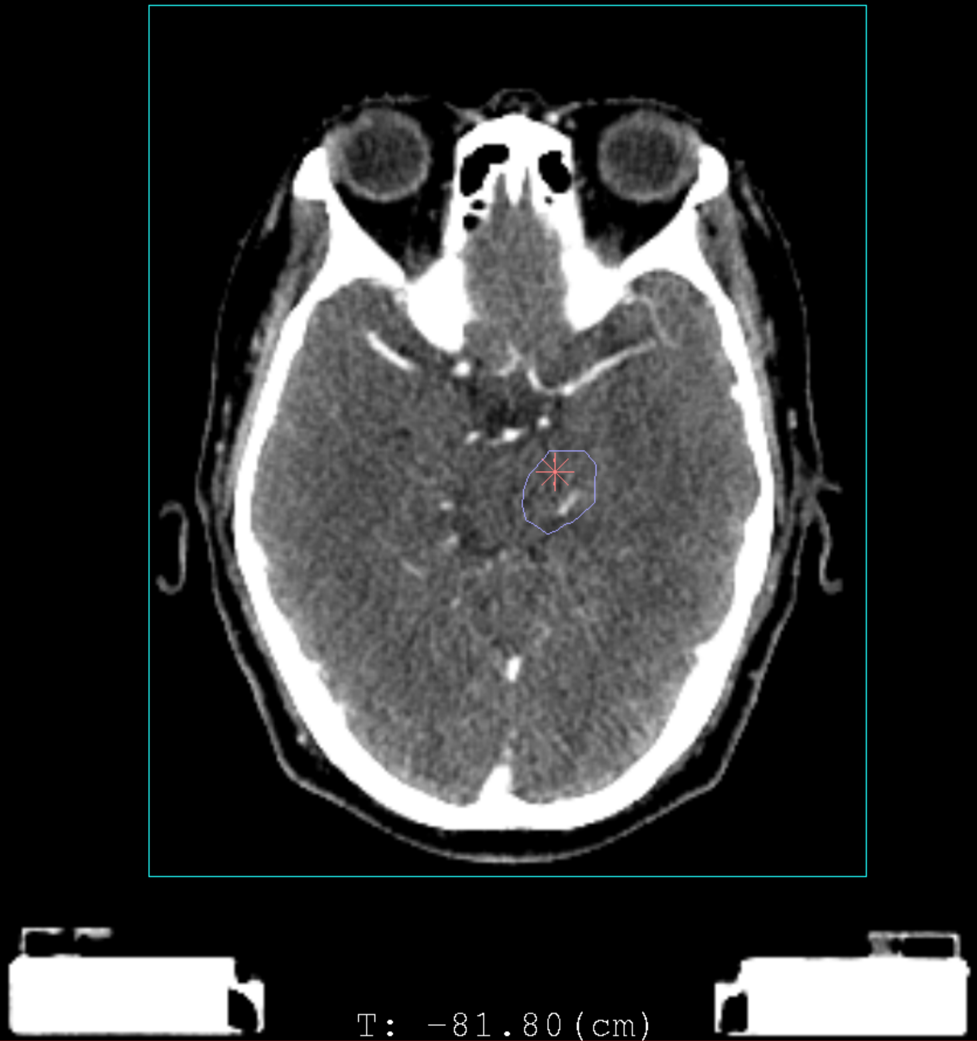

Maximized

T: -81.80(cm)

Scale=1: 1.12

ELEKTA

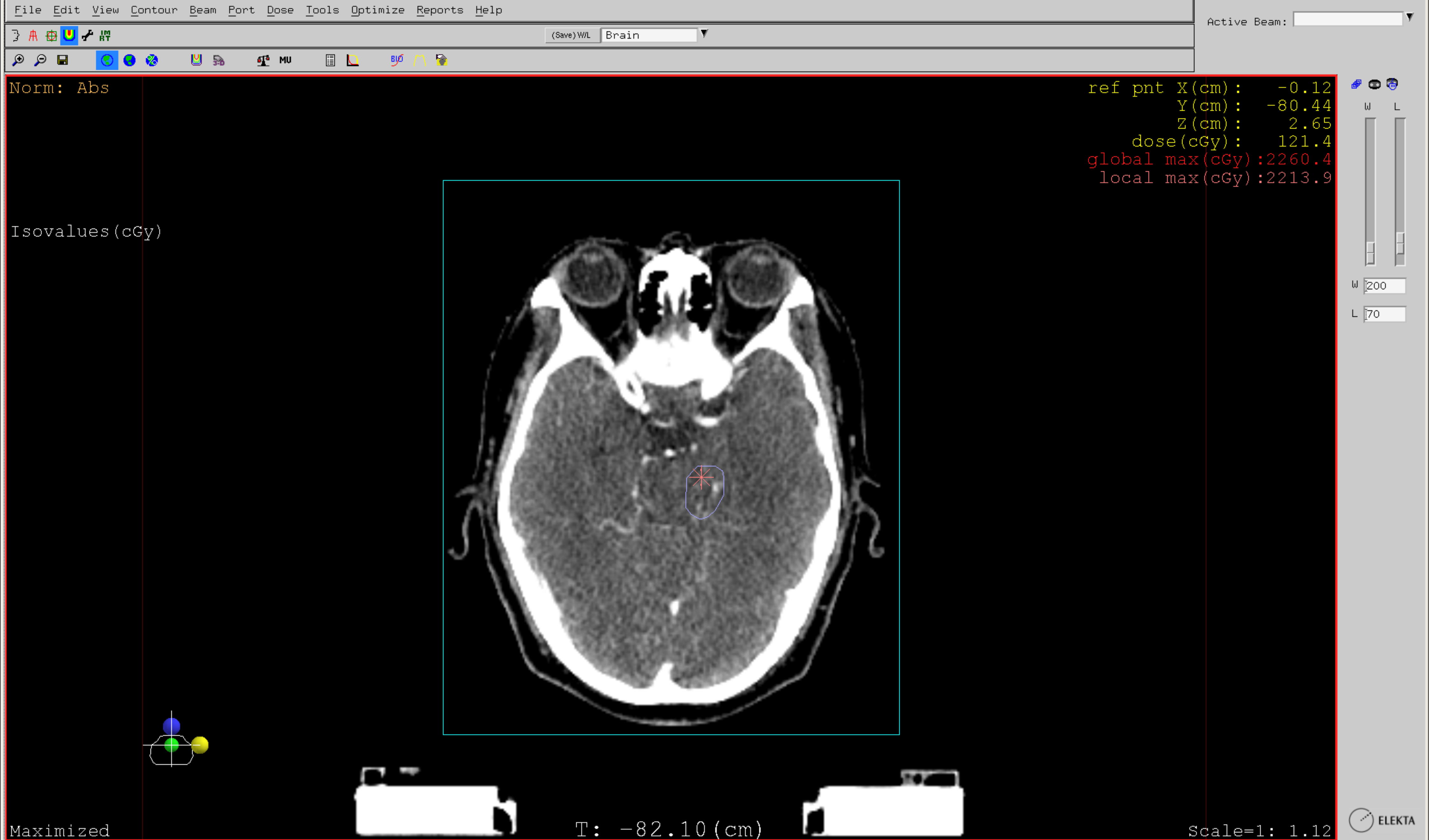
File Edit View Contour Beam Port Dose Tools Optimize Reports Help
Active Beam:
(Save) W/L
Brain
MU
Norm: Abs
ref pnt X(cm): -0.12
Y(cm): -80.44
Z(cm): 2.65
dose(cGy): 121.4
global max(cGy):2260.4
local max(cGy):2213.9
Isovalues(cGy)
W
L
W 200
L 70
Maximized
T: -82.10(cm)
Scale=1: 1.12
ELEKTA

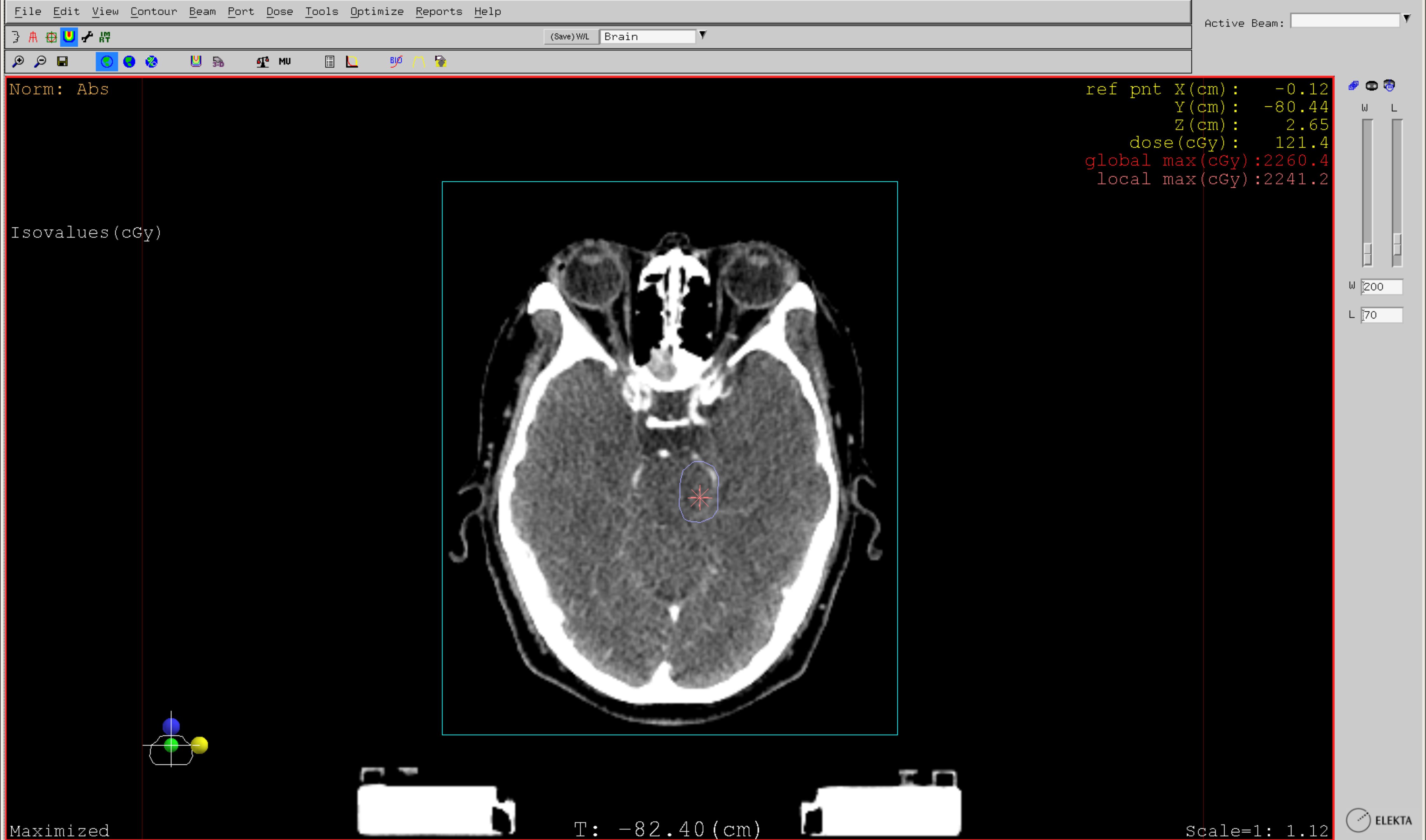

File Edit View Contour Beam Port Dose Tools Optimize Reports Help
Active Beam:
(Save) W/L
Brain
MU
Norm: Abs
ref pnt X(cm): -0.12
Y(cm): -80.44
Z(cm): 2.65
dose(cGy): 121.4
global max(cGy):2260.4
local max(cGy):2241.2
Isovalues(cGy)
W
L
W 200
L 70
Maximized
T: -82.40(cm)
Scale=1: 1.12
ELEKTA

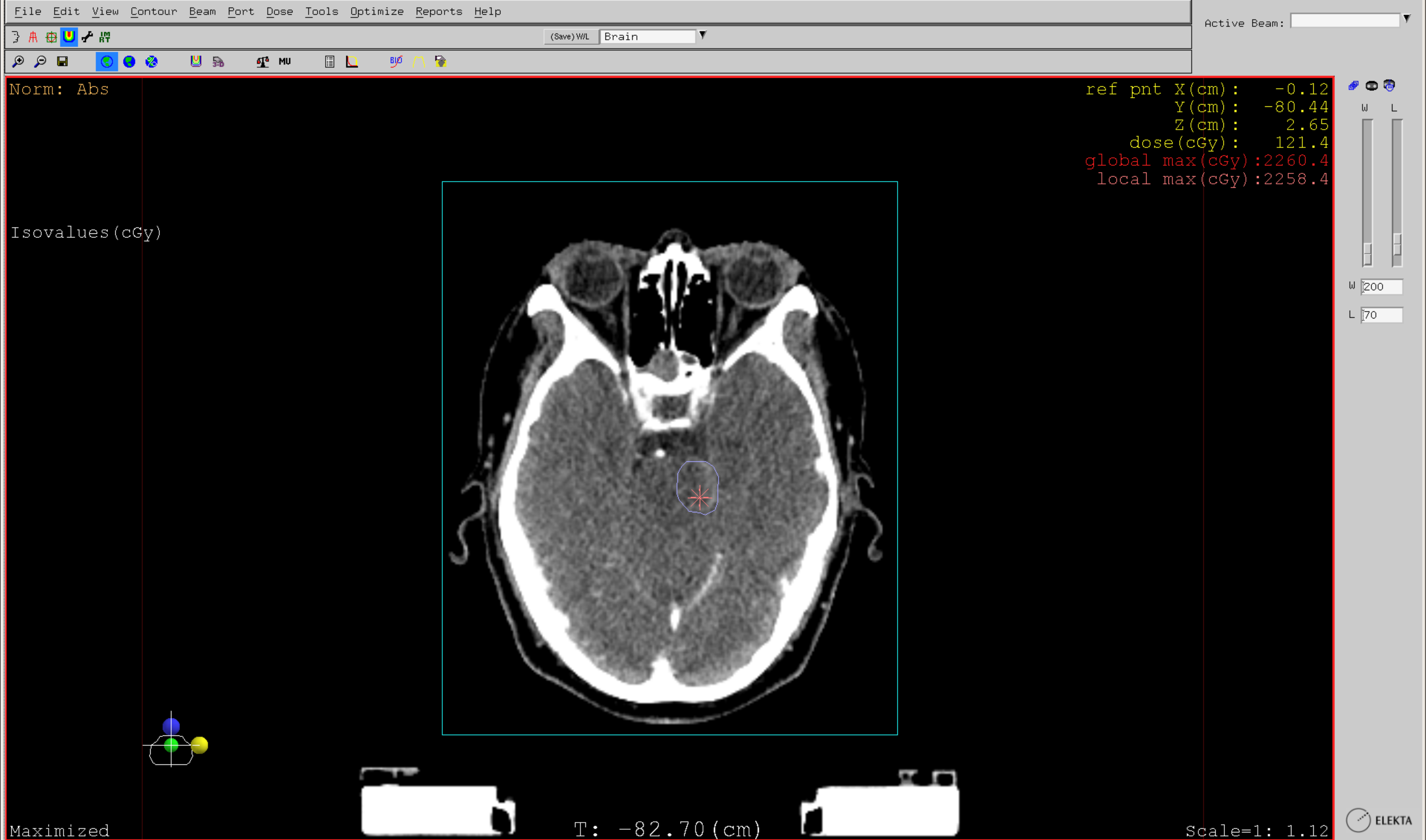
File Edit View Contour Beam Port Dose Tools Optimize Reports Help
Active Beam:
(Save) W/L
Brain
MU
Norm: Abs
ref pnt X(cm): -0.12
Y(cm): -80.44
Z(cm): 2.65
dose(cGy): 121.4
global max(cGy):2260.4
local max(cGy):2258.4
Isovalues(cGy)
W
L
W 200
L 70
Maximized
T: -82.70(cm)
Scale=1: 1.12
ELEKTA

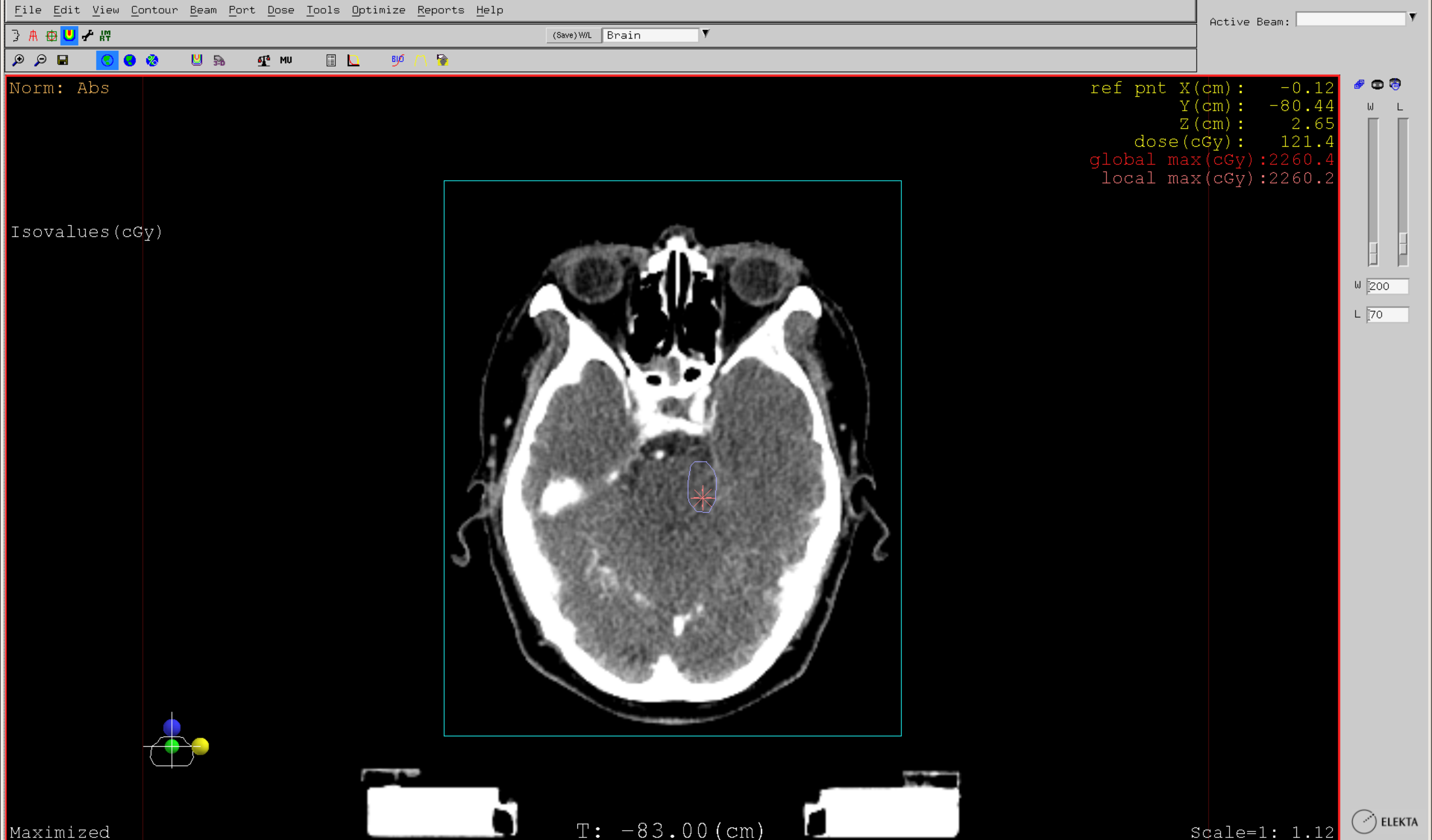
File Edit View Contour Beam Port Dose Tools Optimize Reports Help
Active Beam:
(Save) W/L
Brain
MU
Norm: Abs
ref pnt X(cm): -0.12
Y(cm): -80.44
Z(cm): 2.65
dose(cGy): 121.4
global max(cGy):2260.4
local max(cGy):2260.2
Isovalues(cGy)
W 200
L 70
Maximized
T: -83.00(cm)
Scale=1: 1.12
ELEKTA

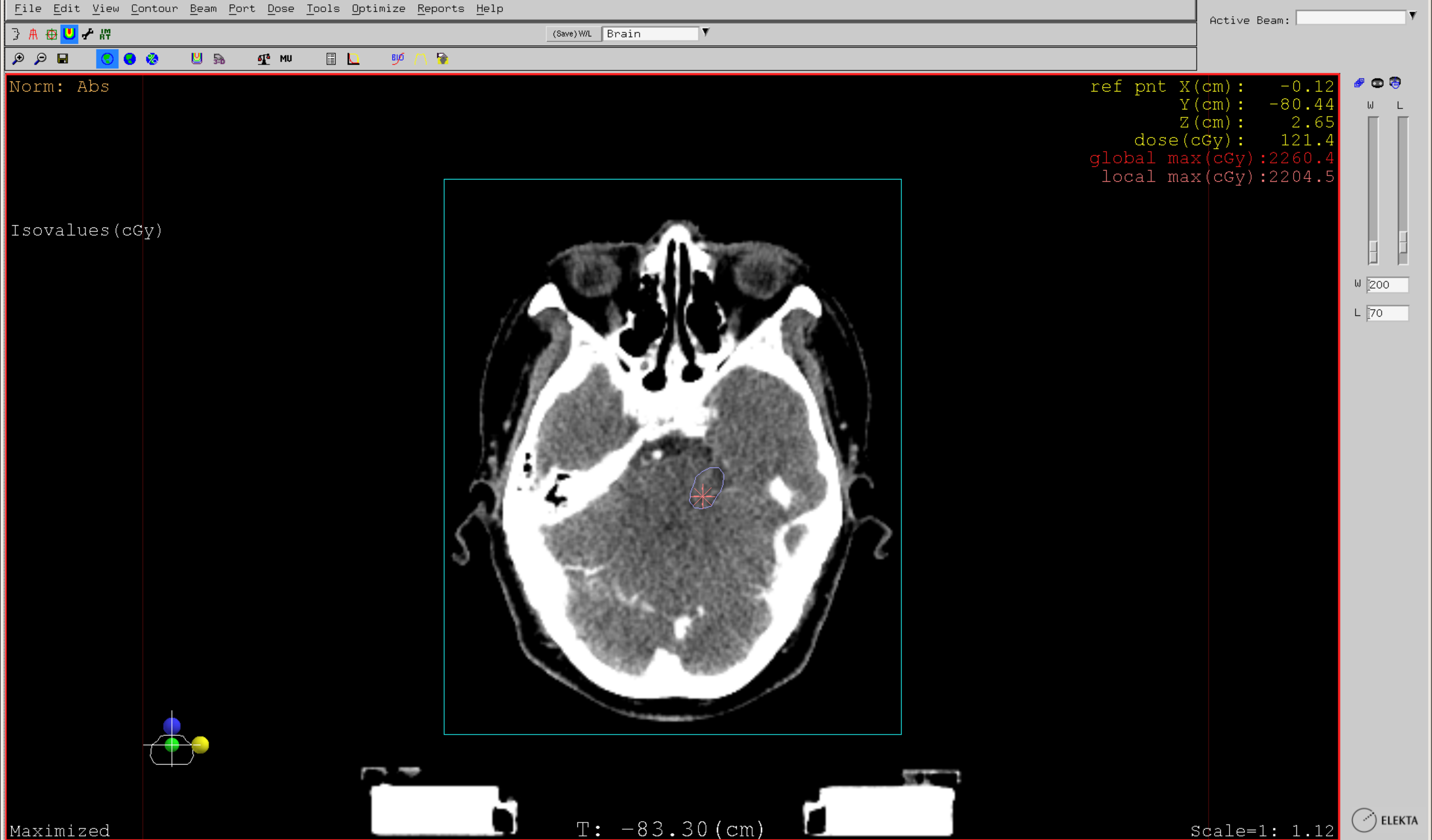
File Edit View Contour Beam Port Dose Tools Optimize Reports Help
Active Beam:
(Save) W/L
Brain
MU
Norm: Abs
ref pnt X(cm): -0.12
Y(cm): -80.44
Z(cm): 2.65
dose(cGy): 121.4
global max(cGy):2260.4
local max(cGy):2204.5
Isovalues(cGy)
W
L
W 200
L 70
Maximized
T: -83.30(cm)
Scale=1: 1.12
ELEKTA
