# Supplementary material for: Sustained complete response to TMEp-CI-M platform in refractory small-cell lung cancer with brainstem metastasis: a case report with over 20 months of disease-free survival
Source: Front Immunol. 2026 Jun 1;17:1807865. doi: 10.3389/fimmu.2026.1807865 (PMC13265516; doi:10.3389/fimmu.2026.1807865)
Supplement: Supplementary Figure 7 — Contrast-enhanced CT of the pulmonary lesion (September 16, 2025). [file DataSheet5.pdf]

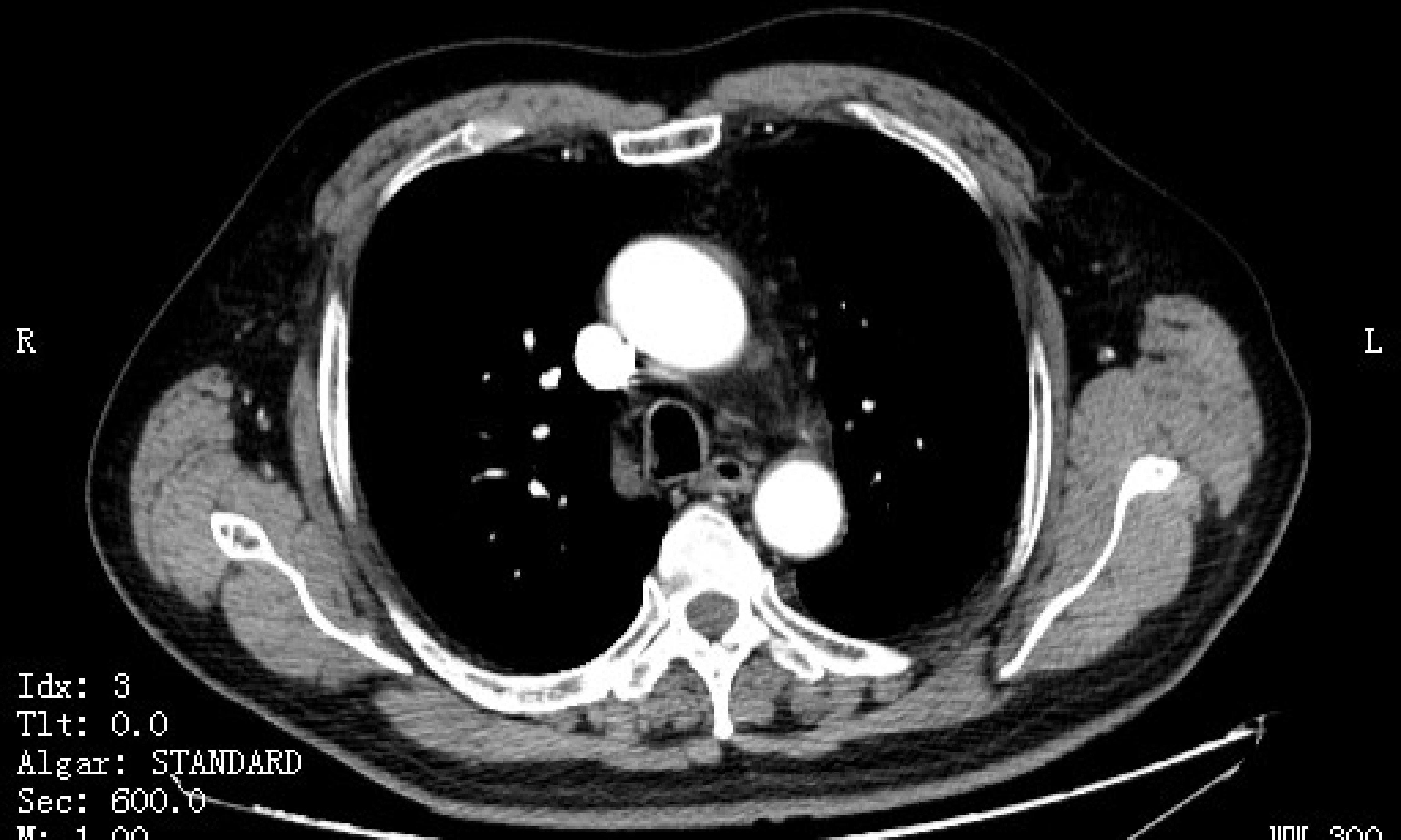
R
L
Idx: 3
Tlt: 0.0
Algar: STANDARD
Sec: 600.0

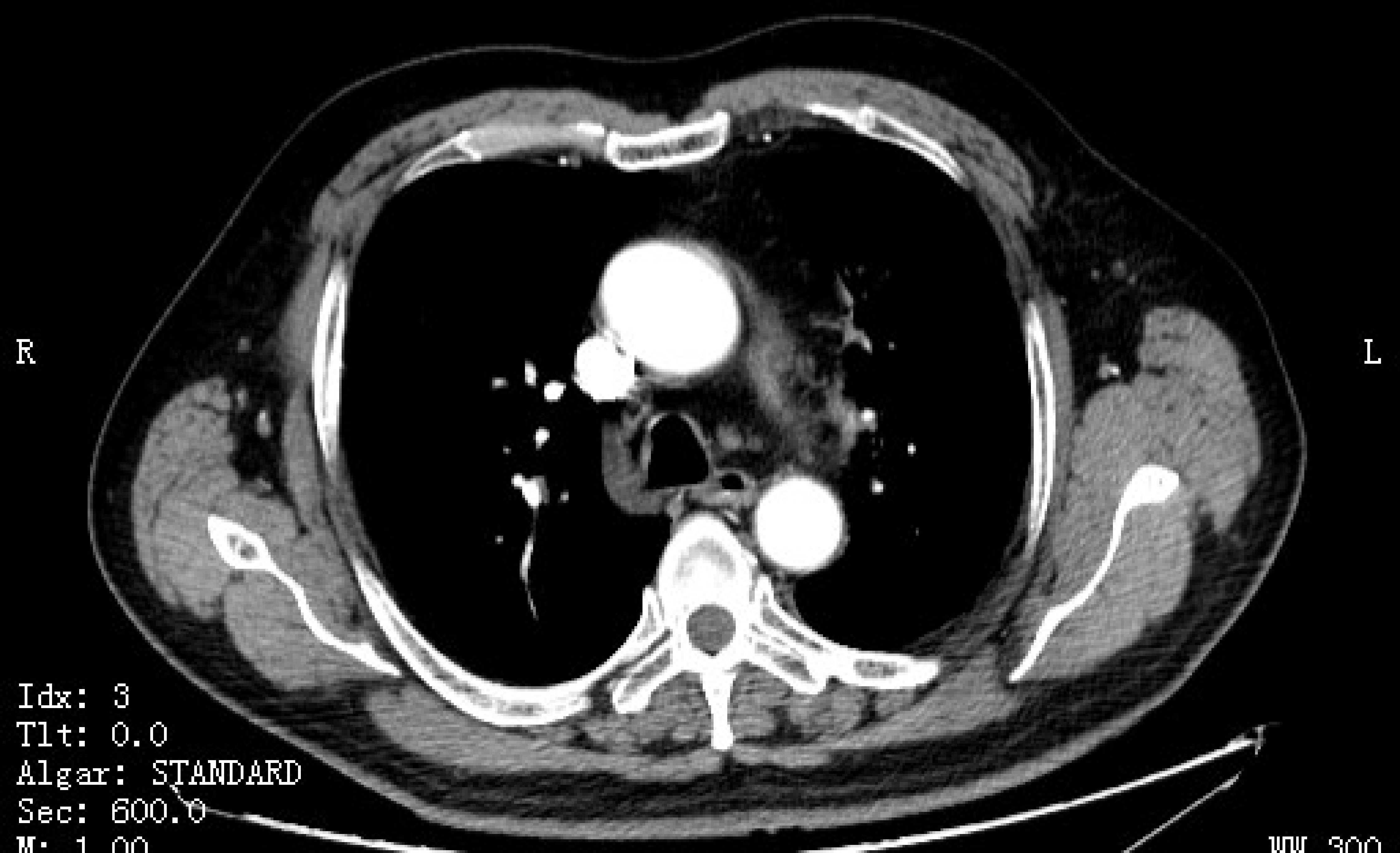
R
L
Idx: 3
Tlt: 0.0
Algar: STANDARD
Sec: 600.0

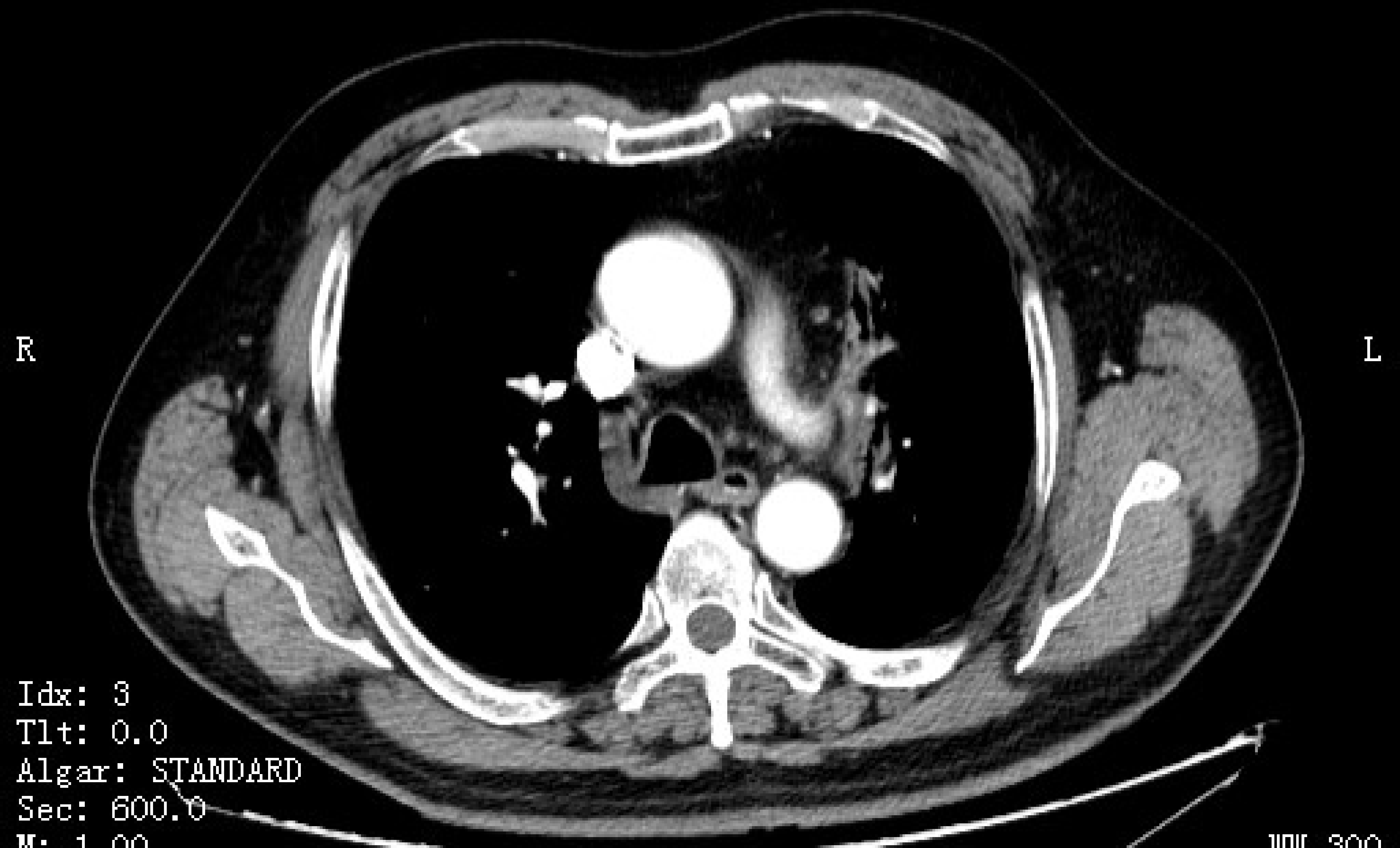
R
L
Idx: 3
Tlt: 0.0
Algar: STANDARD
Sec: 600.0

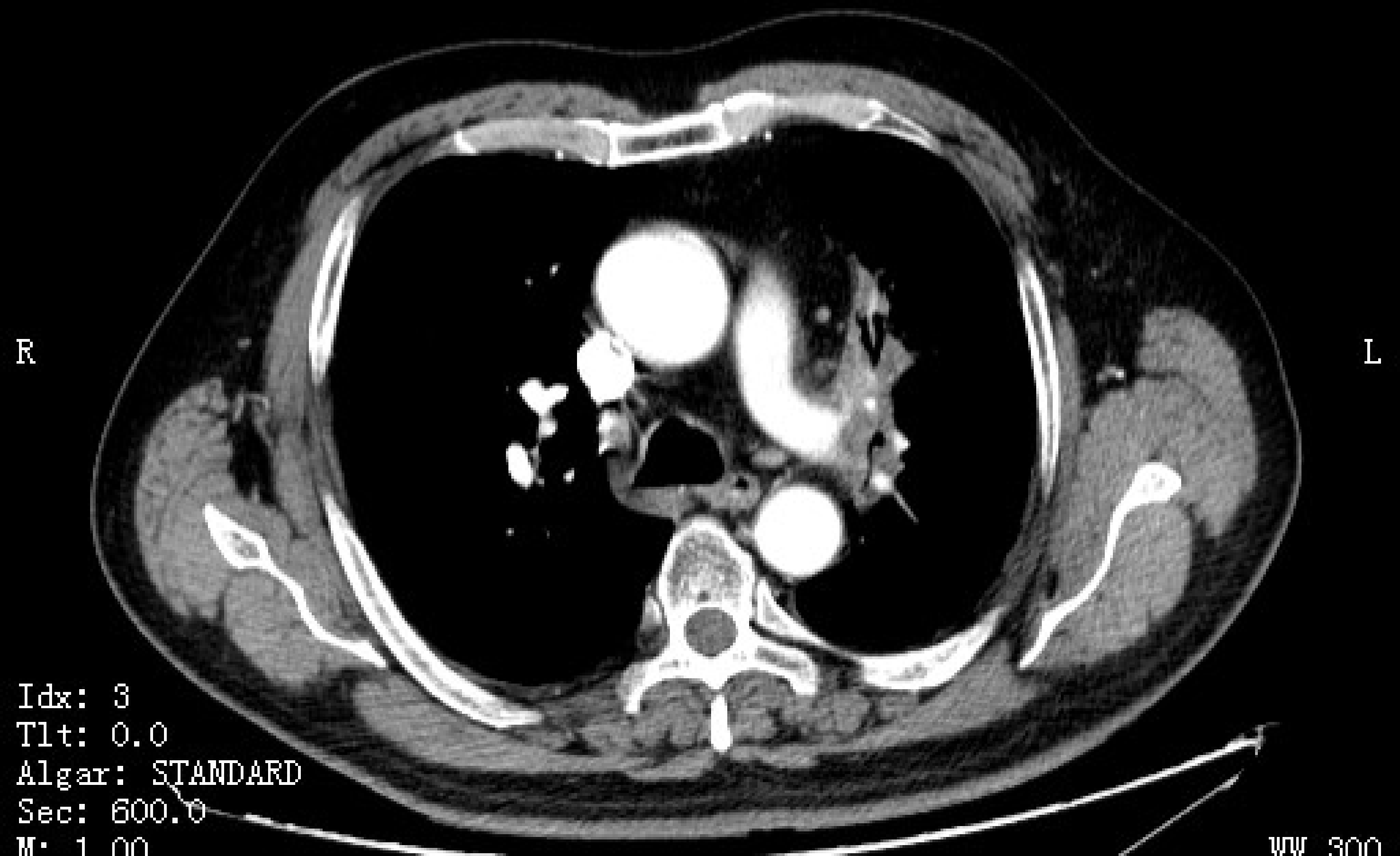
R
L
Idx: 3
Tlt: 0.0
Algar: STANDARD
Sec: 600.0

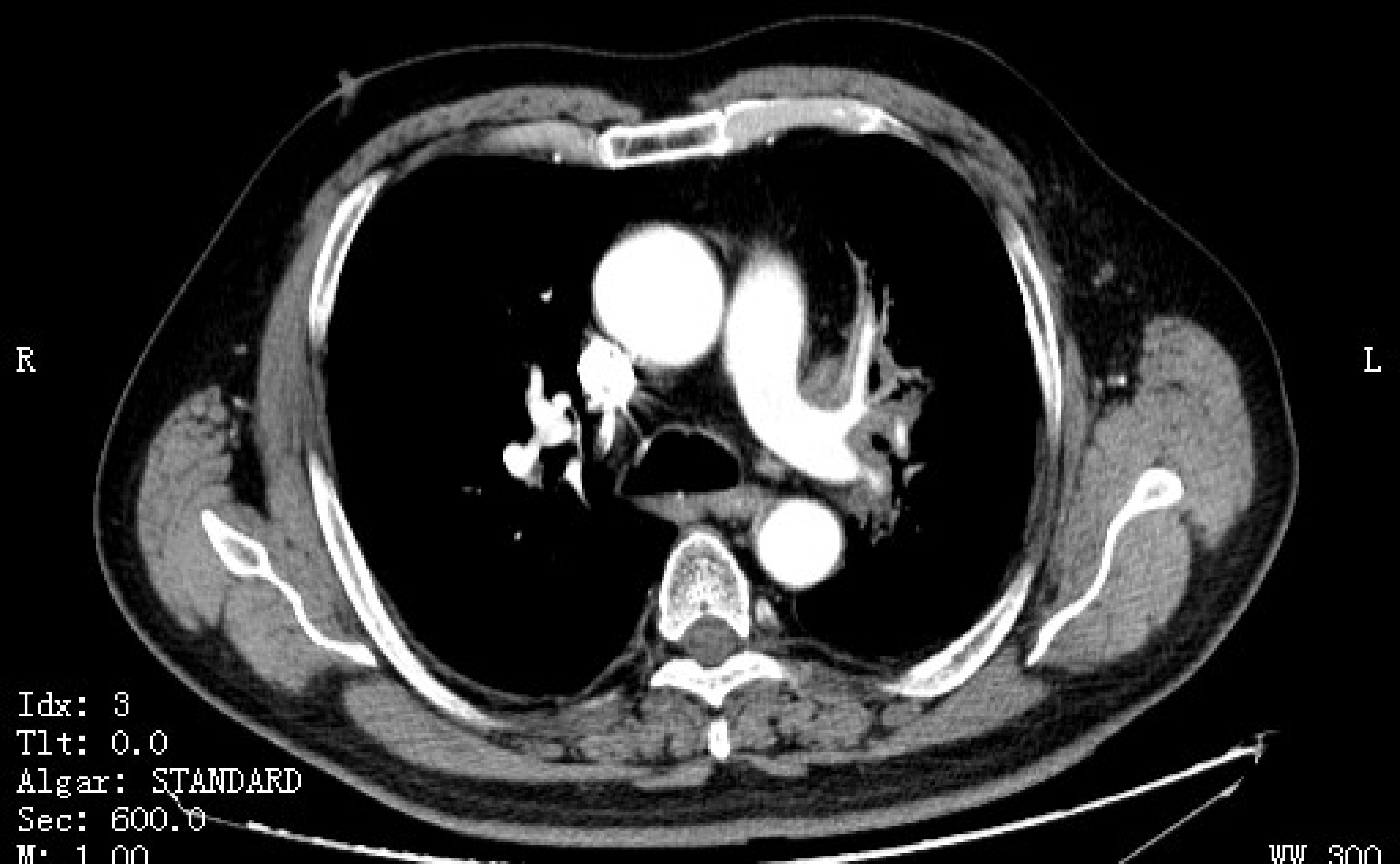
R
L
Idx: 3
Tlt: 0.0
Algar: STANDARD
Sec: 600.0
M: 1.00
WW 300

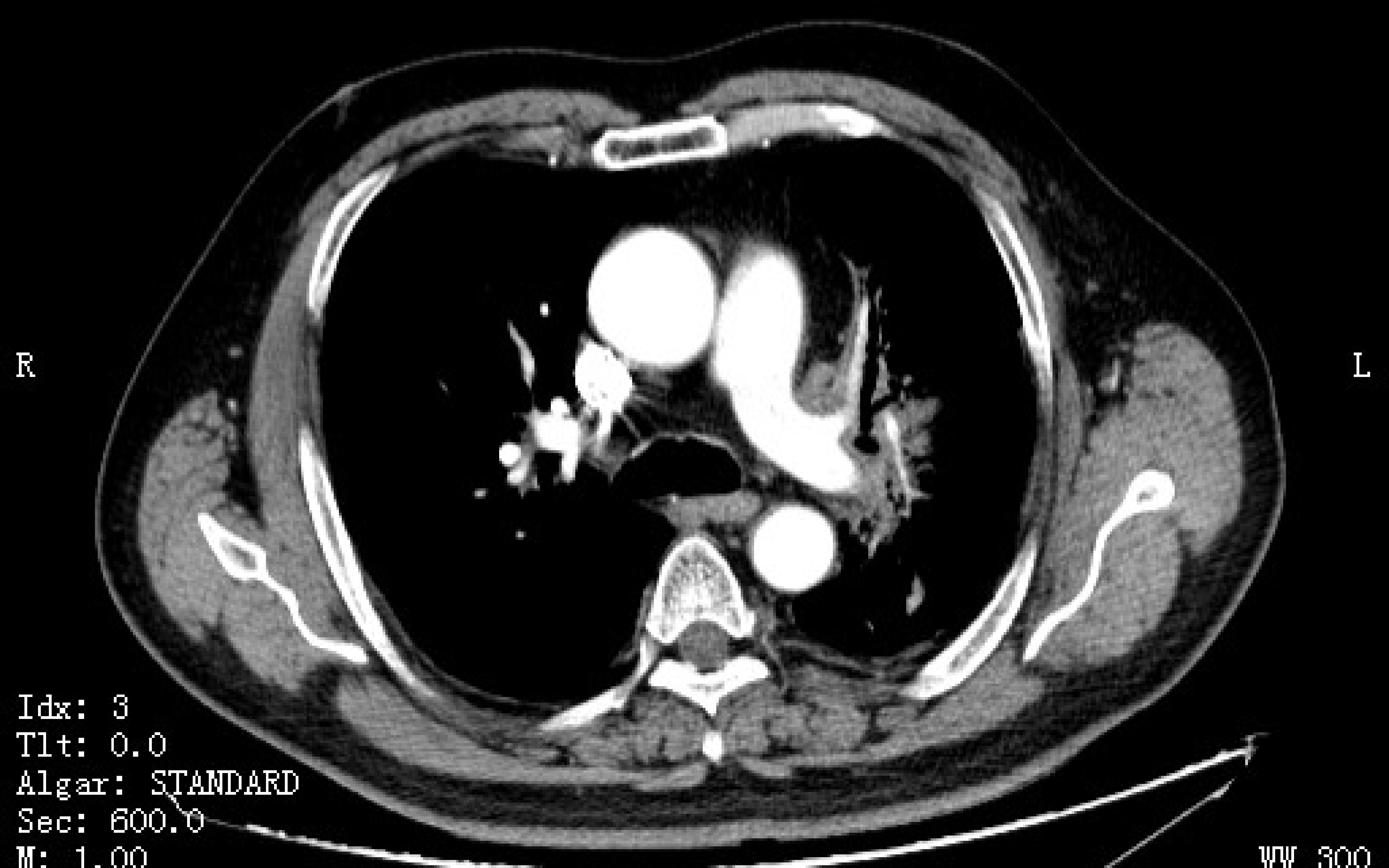
R
L
Idx: 3
Tlt: 0.0
Algar: STANDARD
Sec: 600.0
M: 1.00
WW 300

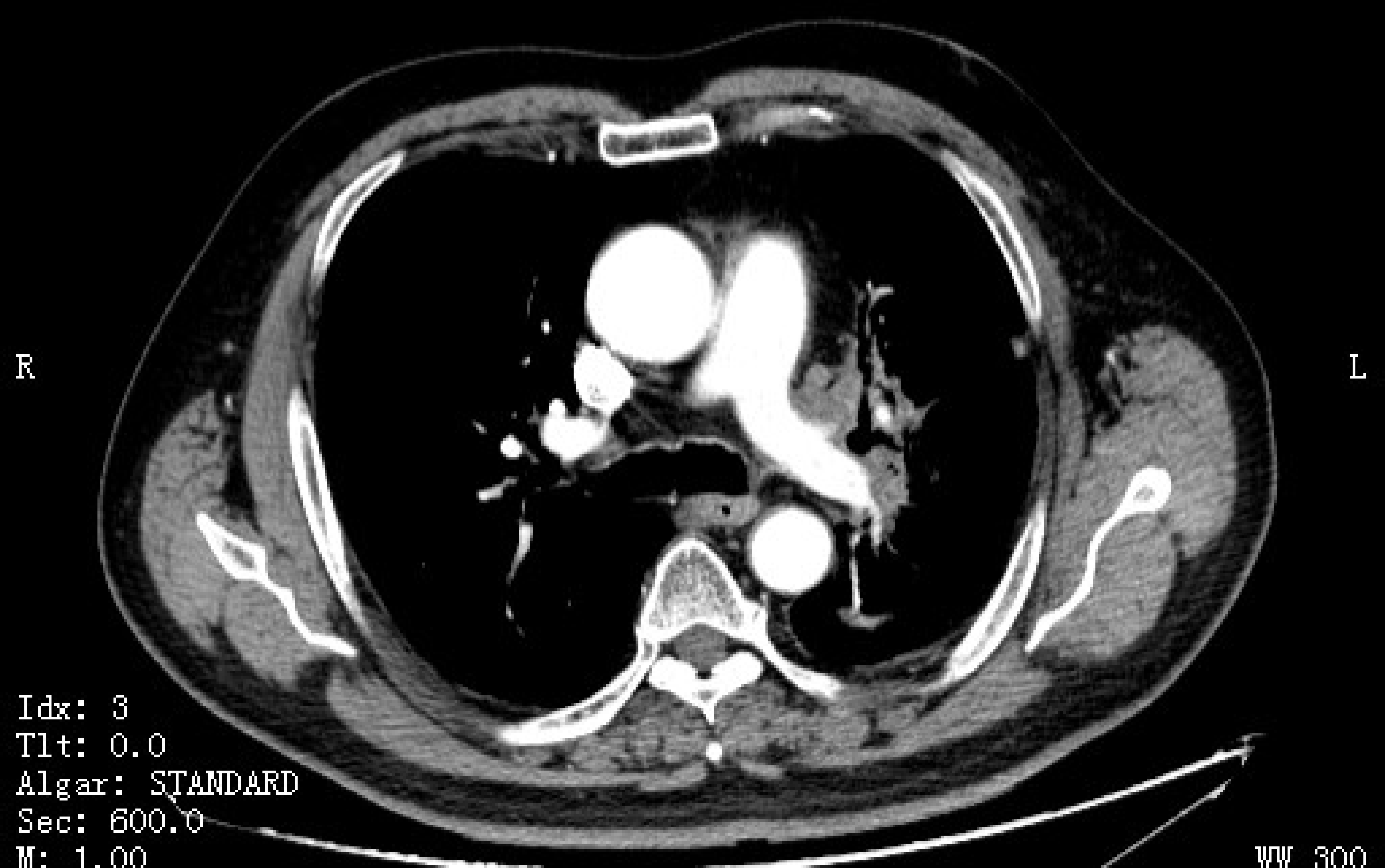
R
L
Idx: 3
Tlt: 0.0
Algar: STANDARD
Sec: 600.0
M: 1.00
WW 300

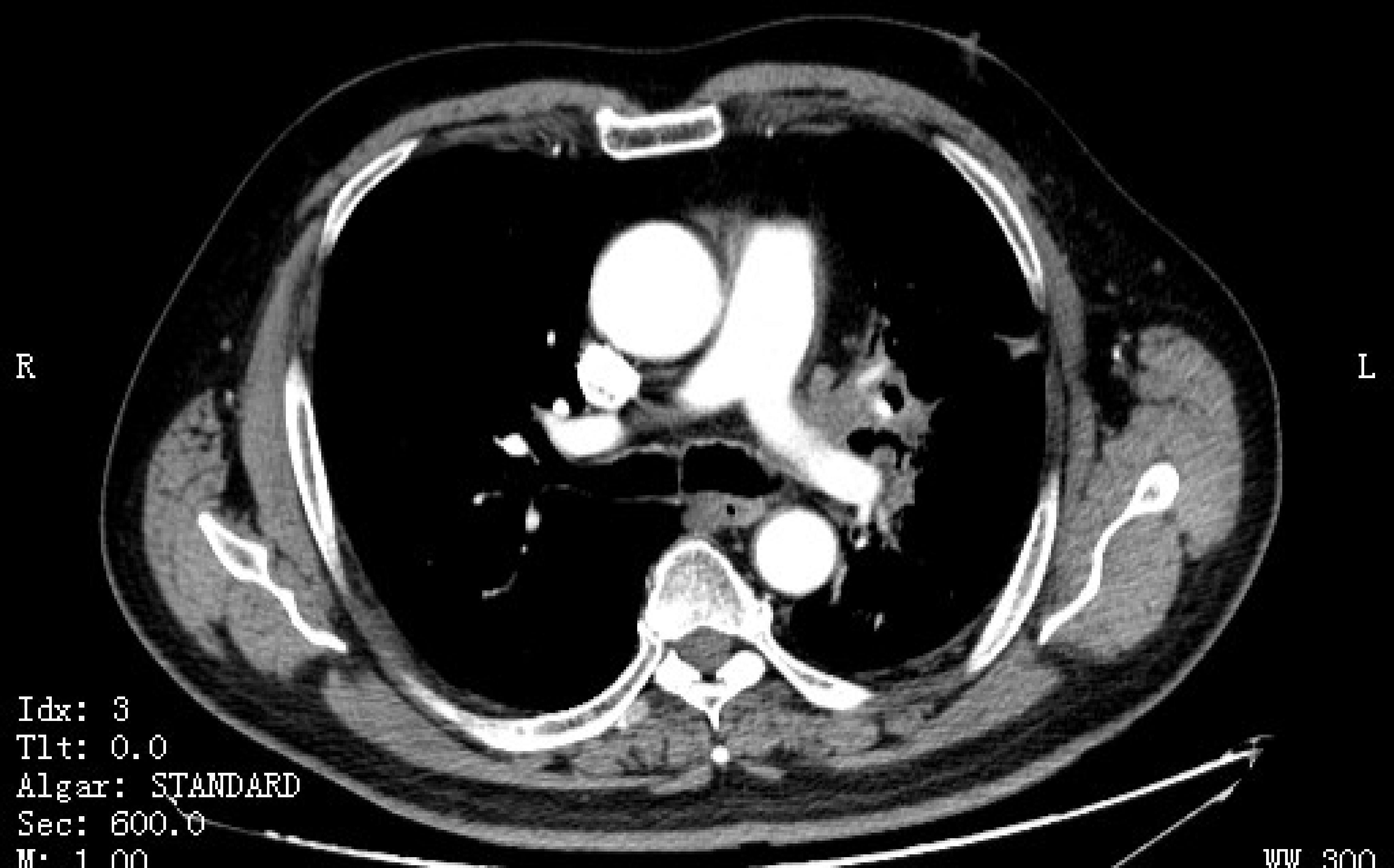
R
L
Idx: 3
Tlt: 0.0
Algar: STANDARD
Sec: 600.0
M: 1.00
WW 300

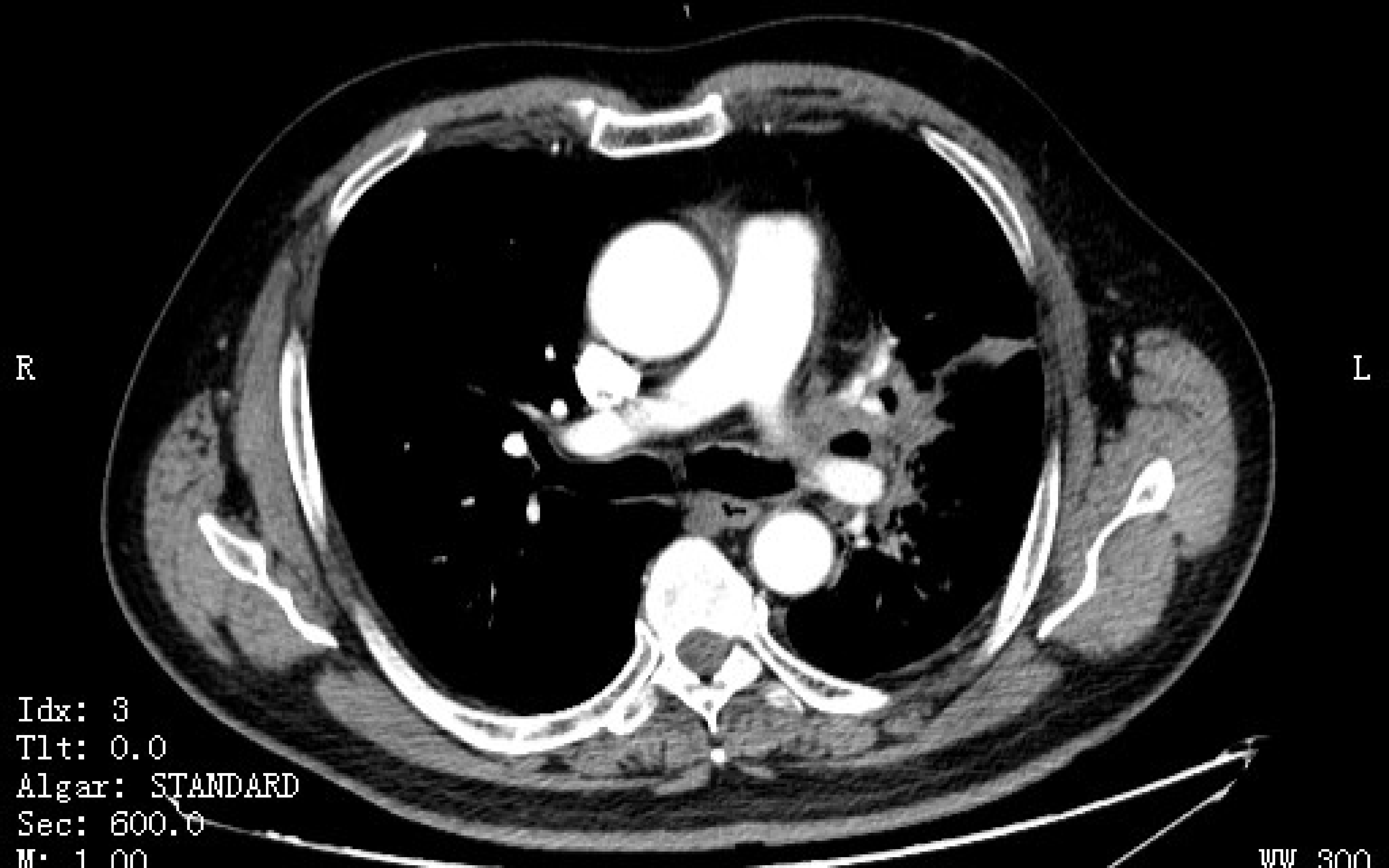
R
L
Idx: 3
Tlt: 0.0
Algar: STANDARD
Sec: 600.0

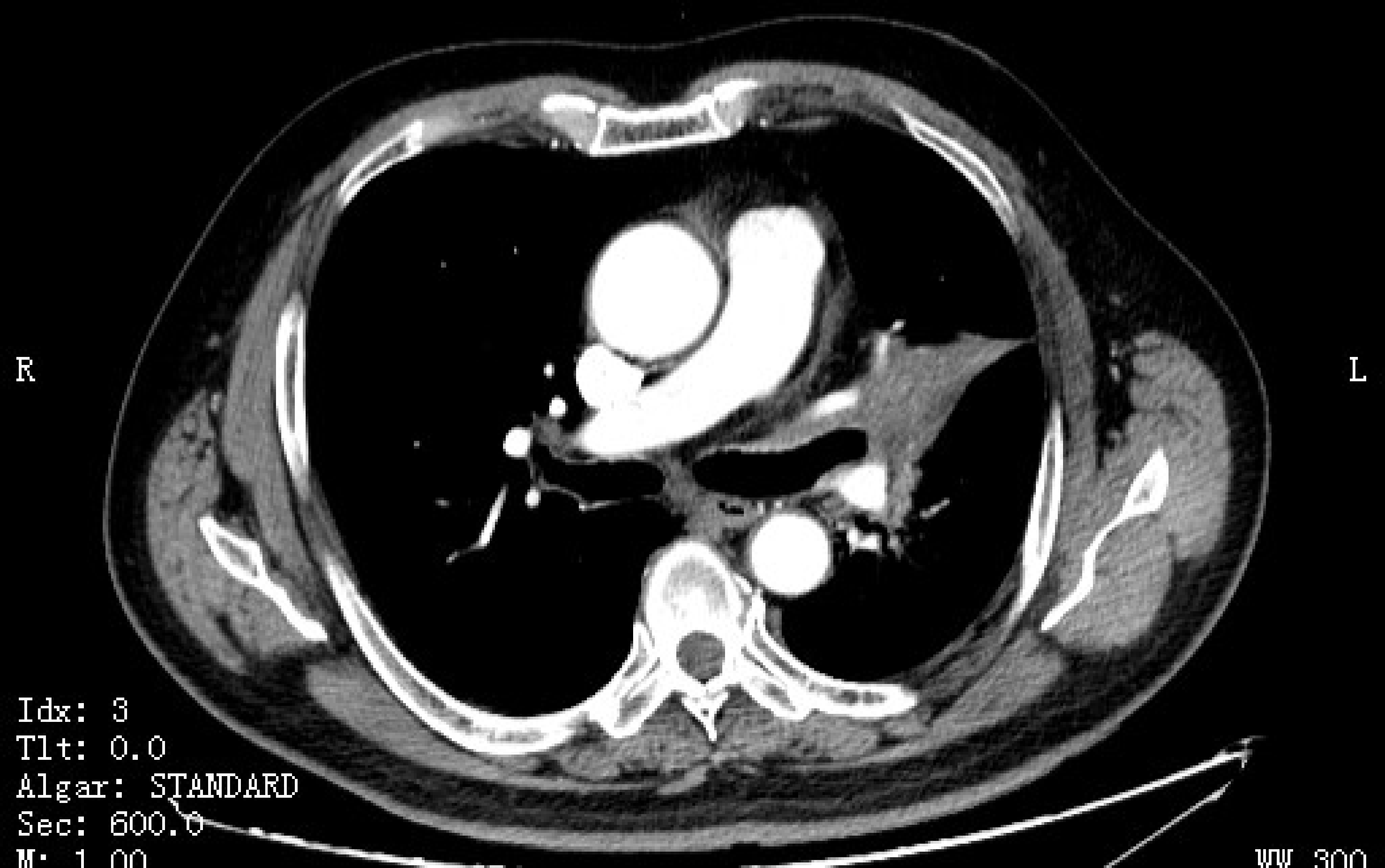
R
L
Idx: 3
Tlt: 0.0
Algar: STANDARD
Sec: 600.0
M: 1.00
WW 300

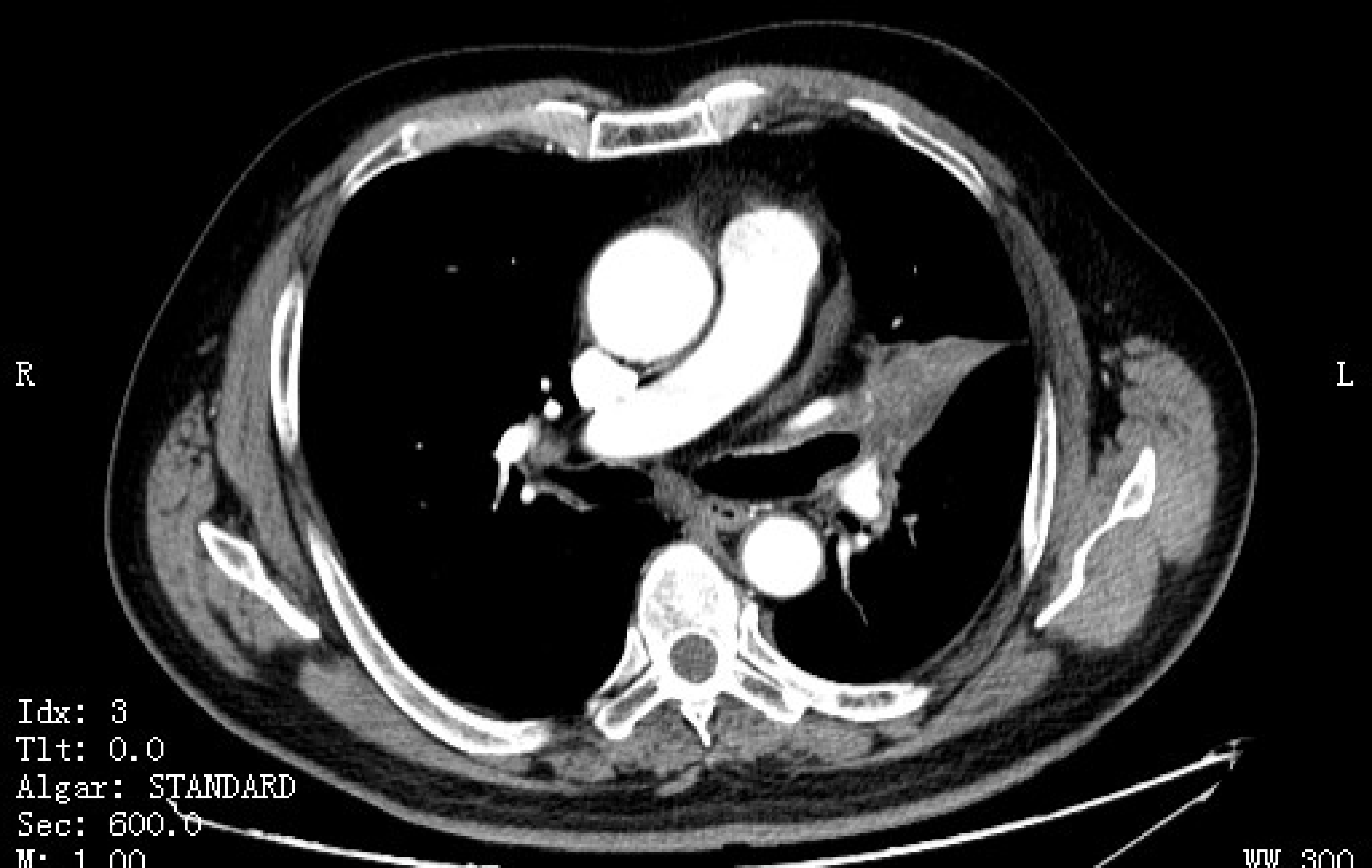
R
L
Idx: 3
Tlt: 0.0
Algar: STANDARD
Sec: 600.0
M: 1.00
WW 300

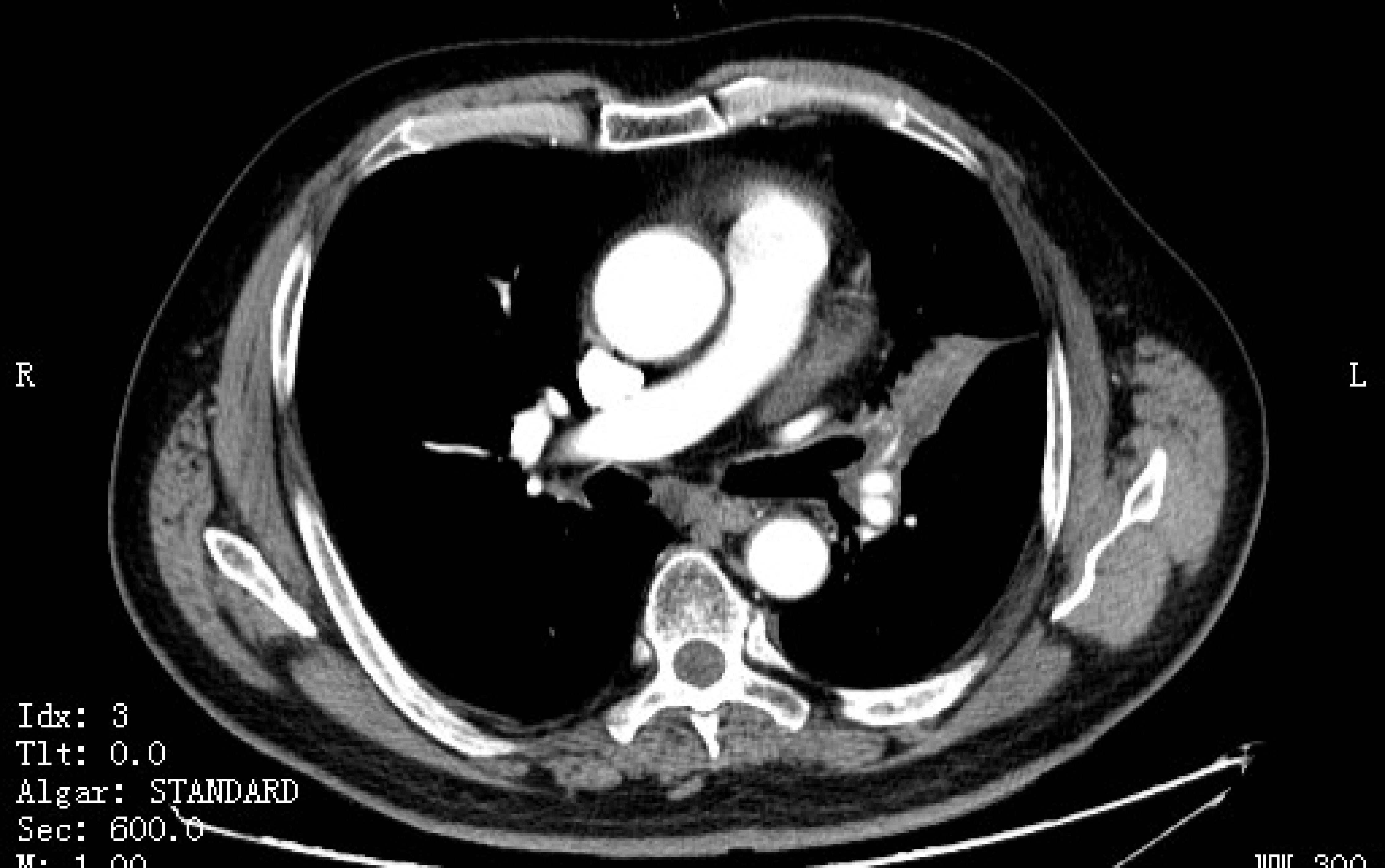
R
L
Idx: 3
Tlt: 0.0
Algar: STANDARD
Sec: 600.0

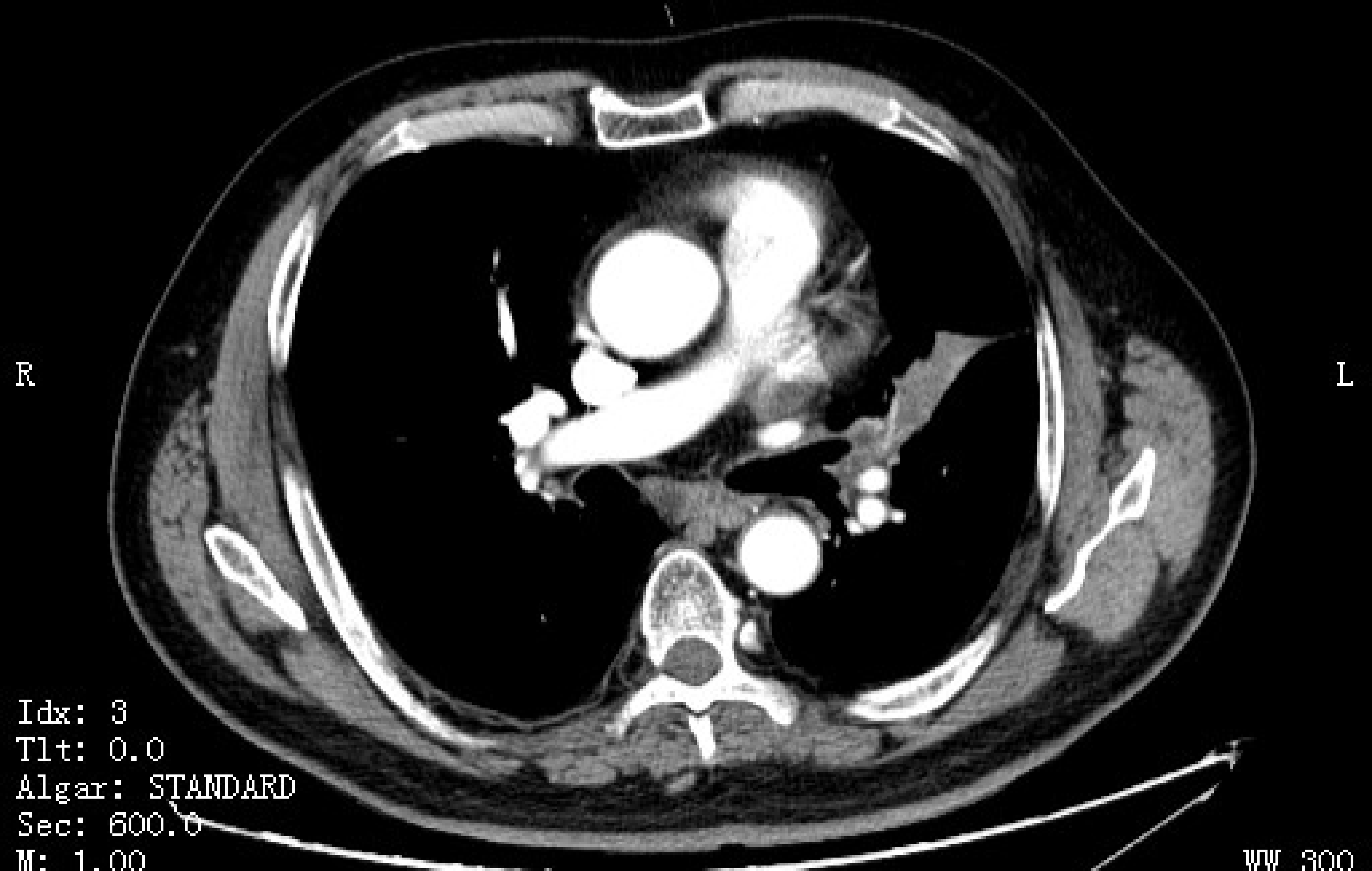
R
L
Idx: 3
Tlt: 0.0
Algar: STANDARD
Sec: 600.0
M: 1.00
WW 300

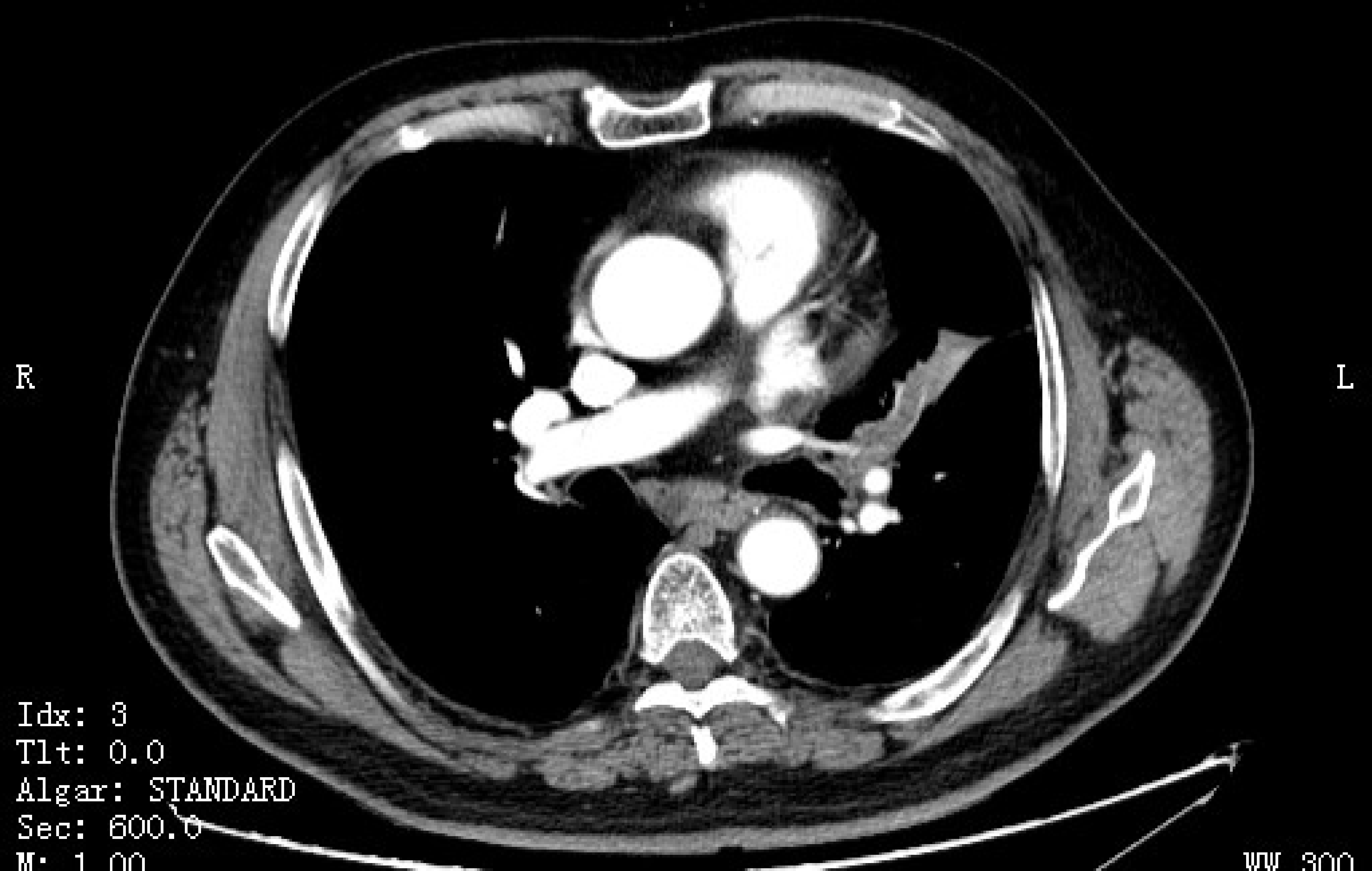
R
L
Idx: 3
Tlt: 0.0
Algar: STANDARD
Sec: 600.0

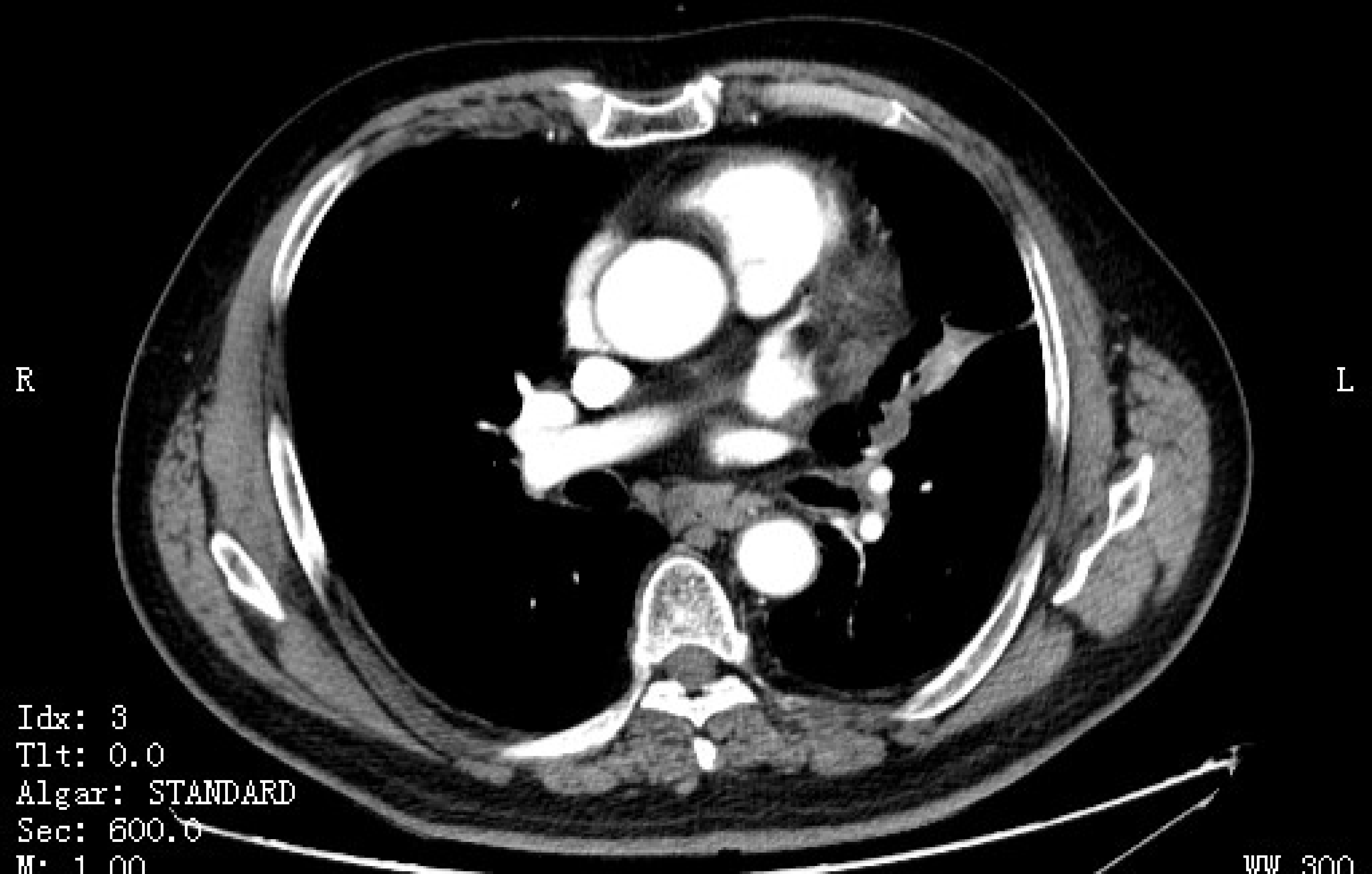
R
L
Idx: 3
Tlt: 0.0
Algar: STANDARD
Sec: 600.0
M: 1.00
WW 300

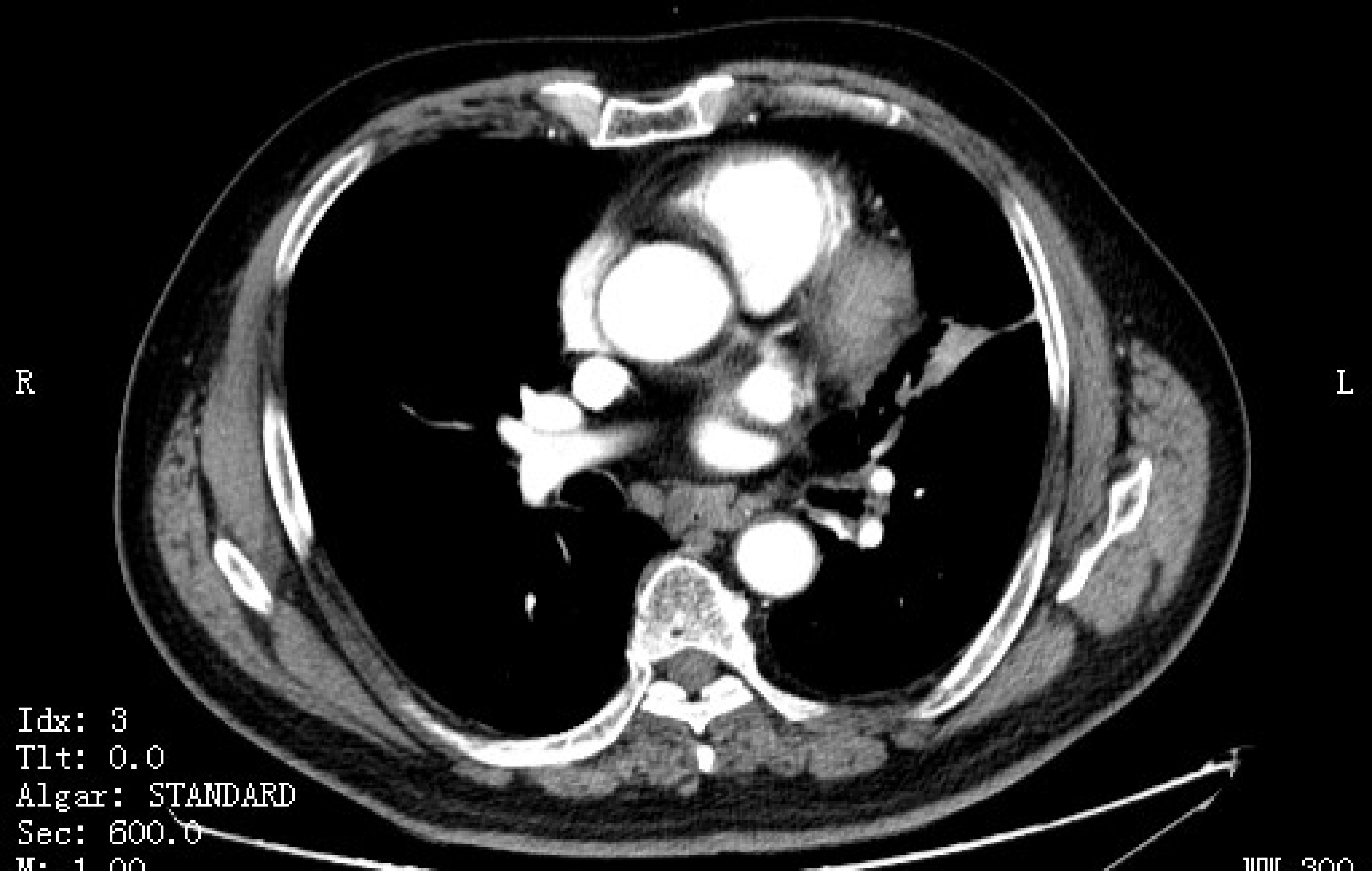
R
L
Idx: 3
Tlt: 0.0
Algar: STANDARD
Sec: 600.0

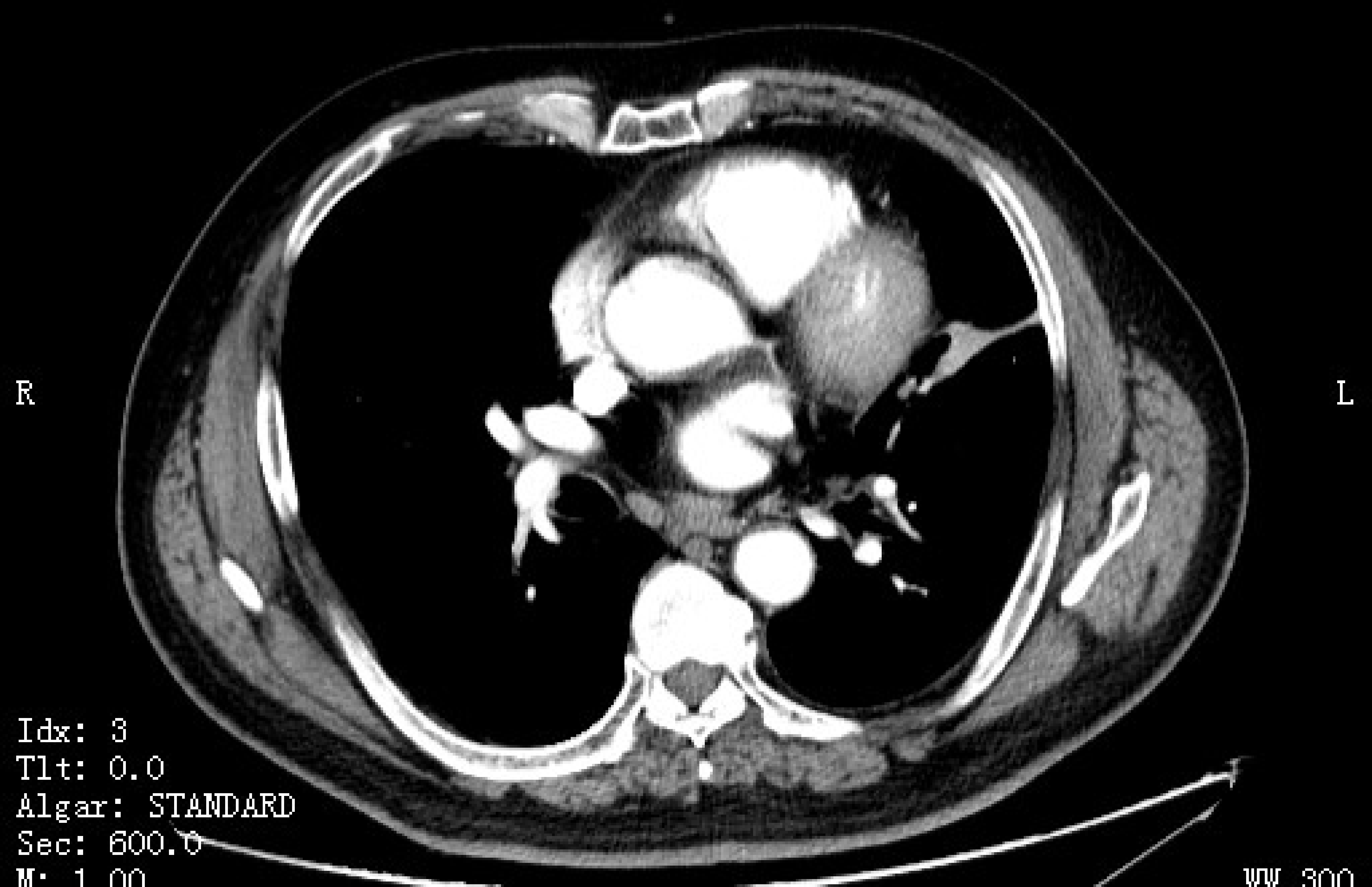
R
L
Idx: 3
Tlt: 0.0
Algar: STANDARD
Sec: 600.0
M: 1.00
WW 300

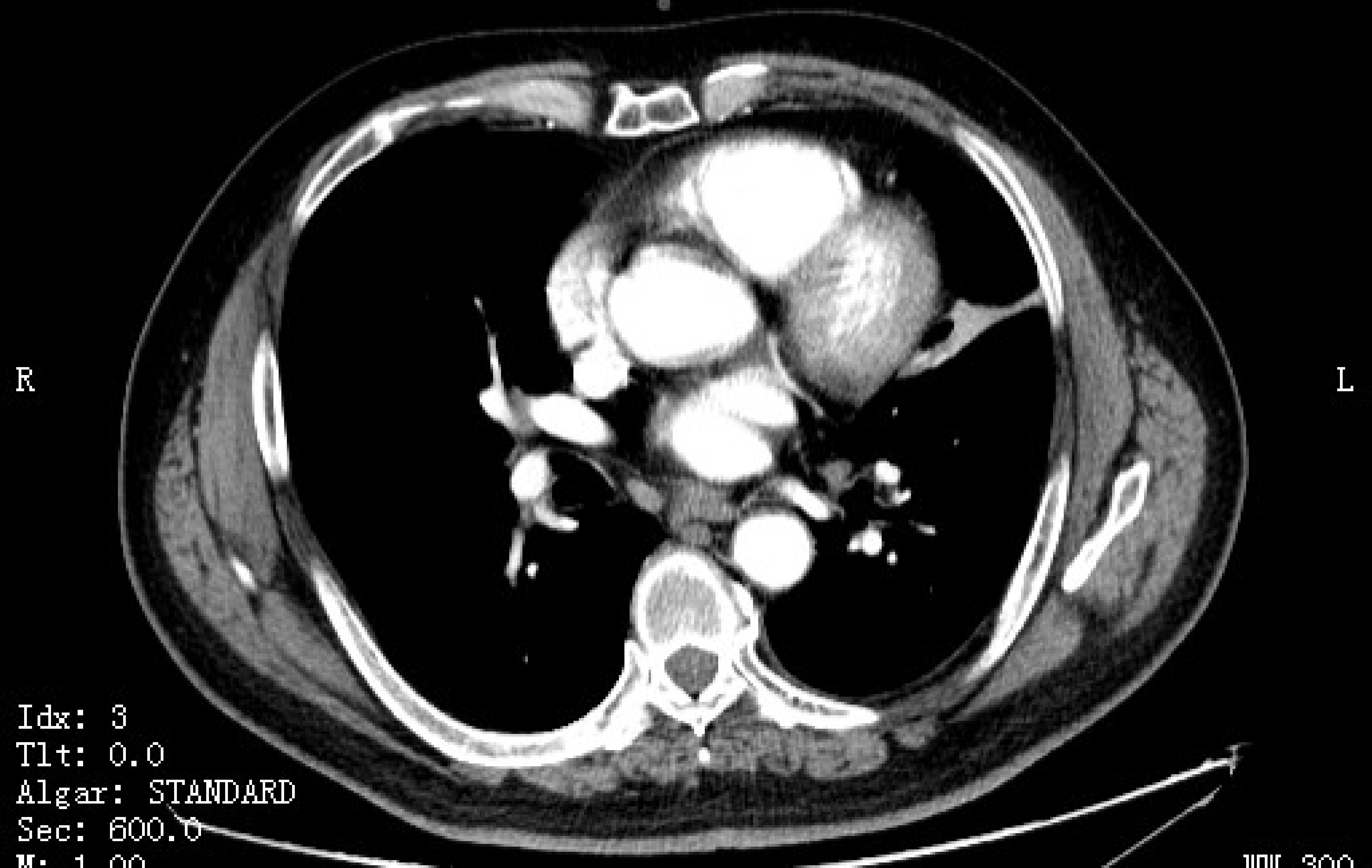
R
L
Idx: 3
Tlt: 0.0
Algar: STANDARD
Sec: 600.0

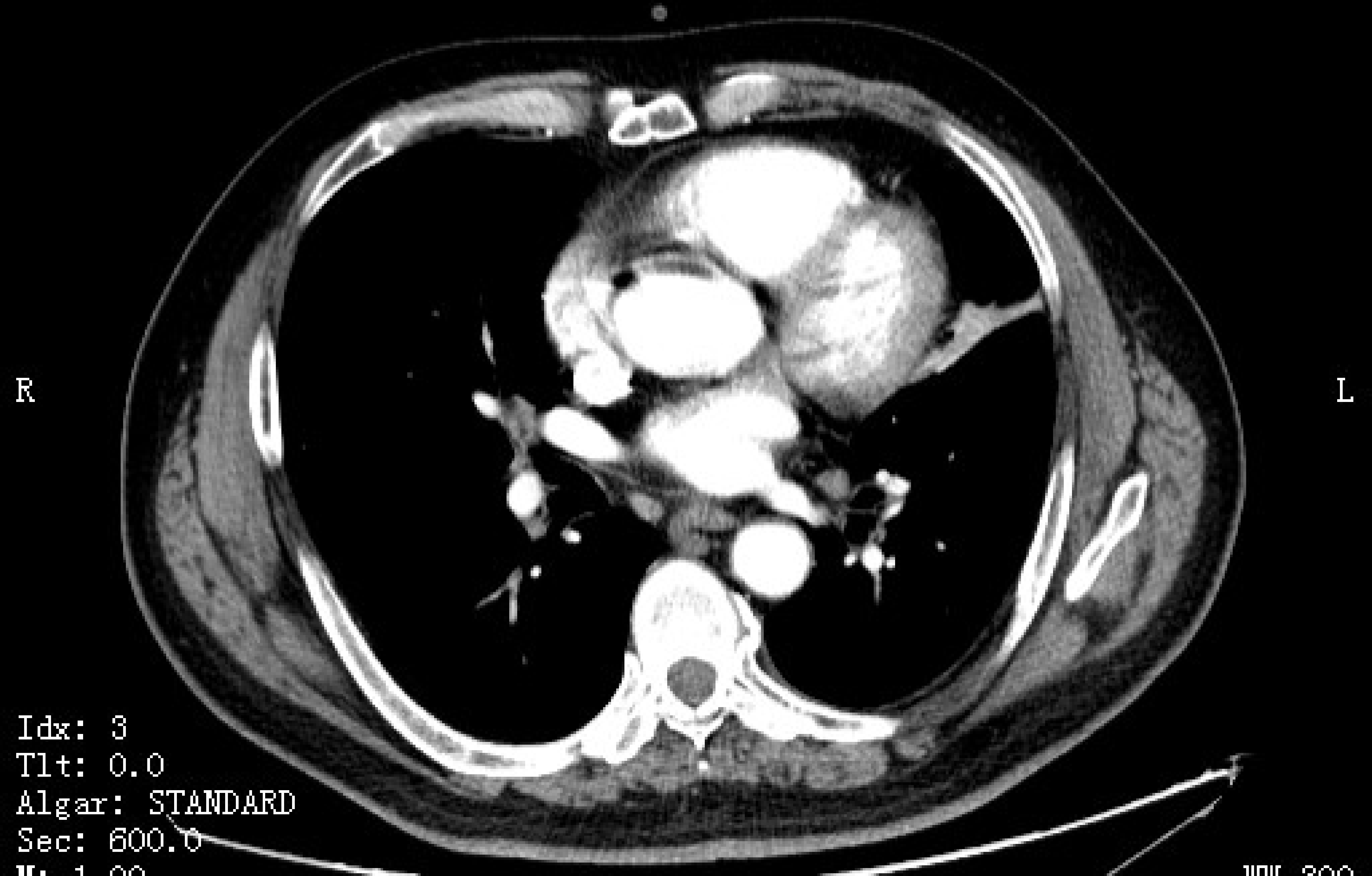
R
L
Idx: 3
Tlt: 0.0
Algar: STANDARD
Sec: 600.0

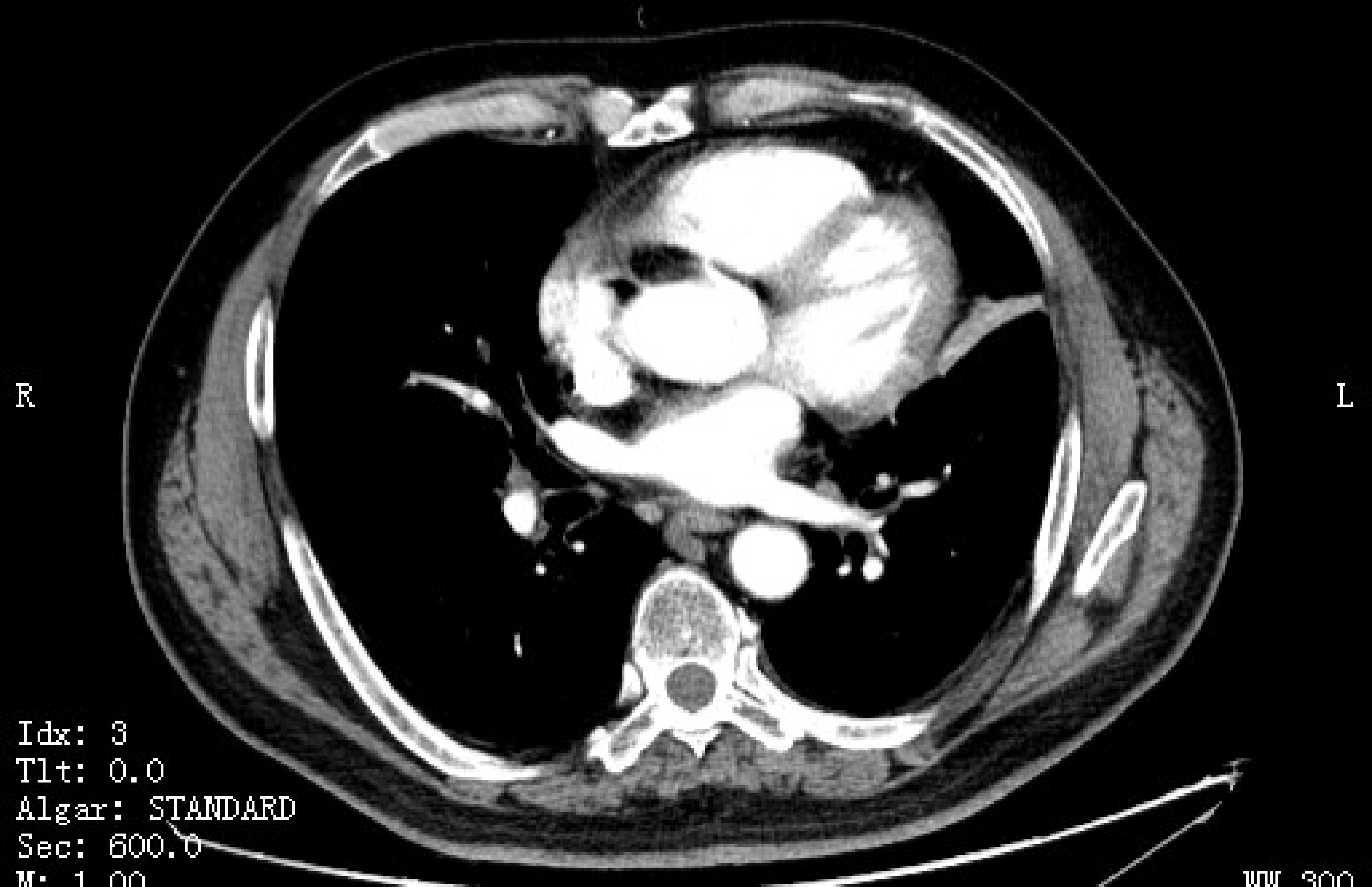
R
L
Idx: 3
Tlt: 0.0
Algar: STANDARD
Sec: 600.0

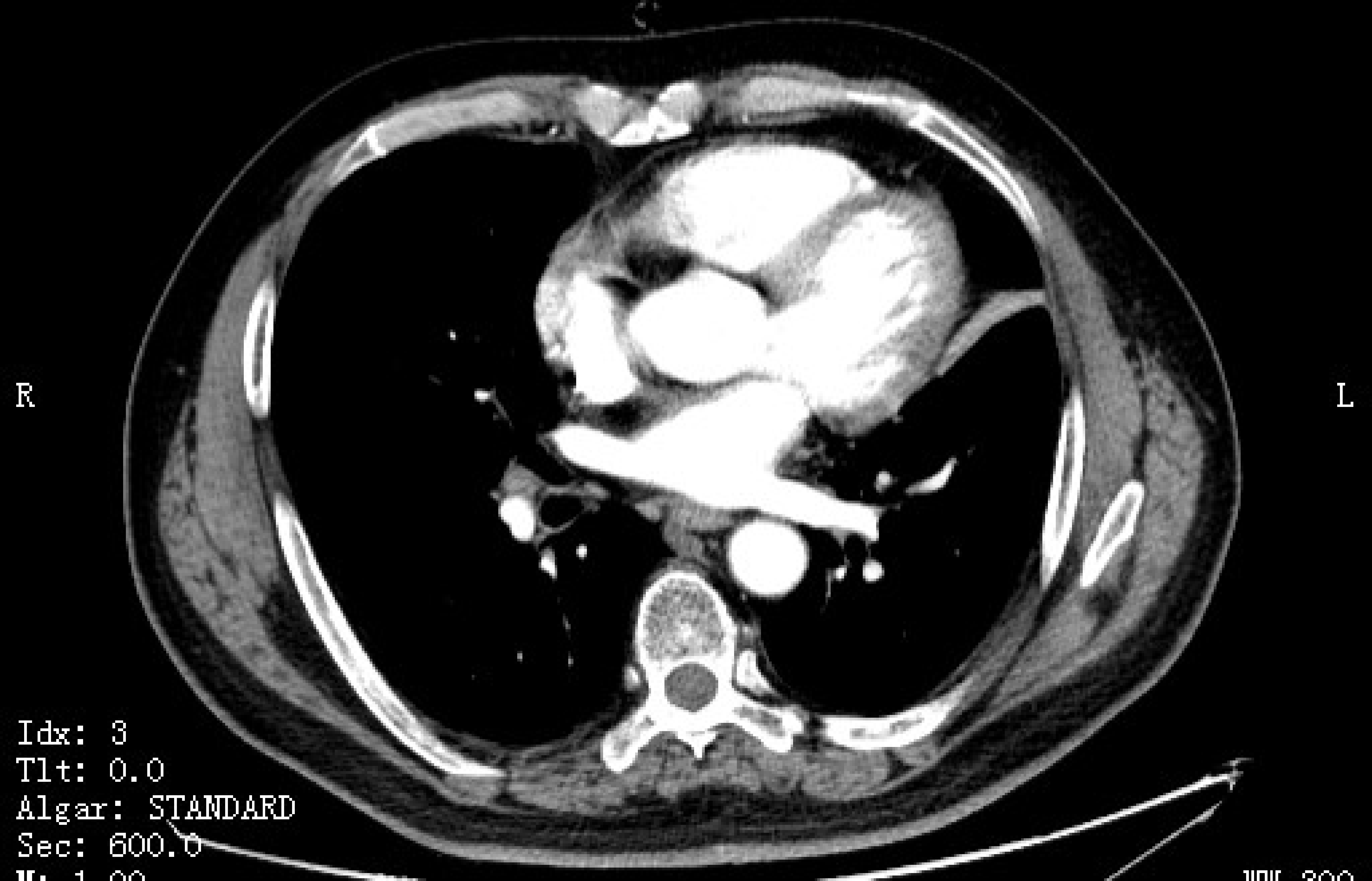
R
L
Idx: 3
Tlt: 0.0
Algar: STANDARD
Sec: 600.0

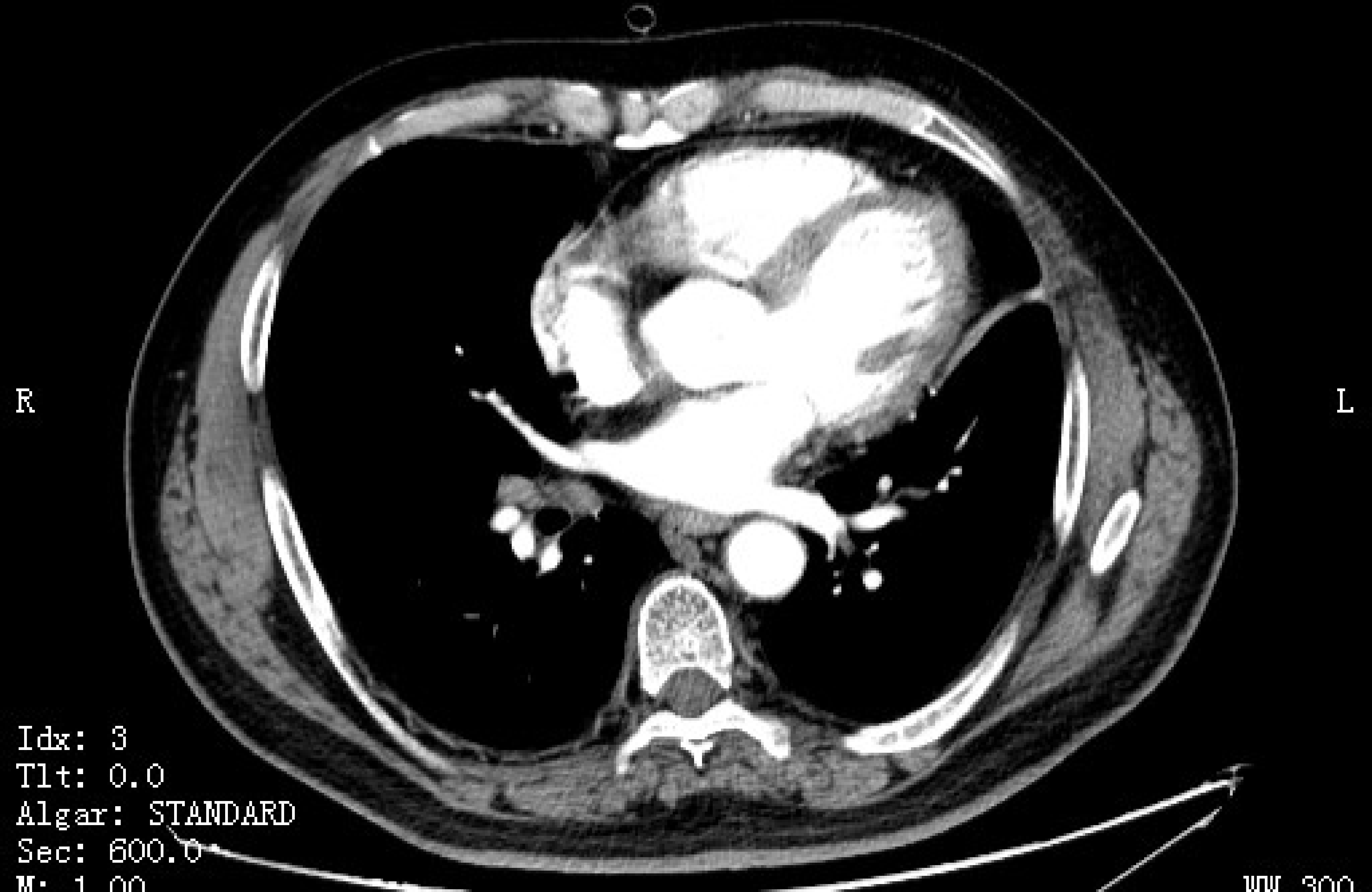
R
L
Idx: 3
Tlt: 0.0
Algar: STANDARD
Sec: 600.0
