# Supplementary material for: Sustained complete response to TMEp-CI-M platform in refractory small-cell lung cancer with brainstem metastasis: a case report with over 20 months of disease-free survival
Source: Front Immunol. 2026 Jun 1;17:1807865. doi: 10.3389/fimmu.2026.1807865 (PMC13265516; doi:10.3389/fimmu.2026.1807865)
Supplement: Supplementary Figure 8 — Contrast-enhanced MRI of the brainstem lesion (September 15, 2025). [file DataSheet6.pdf]

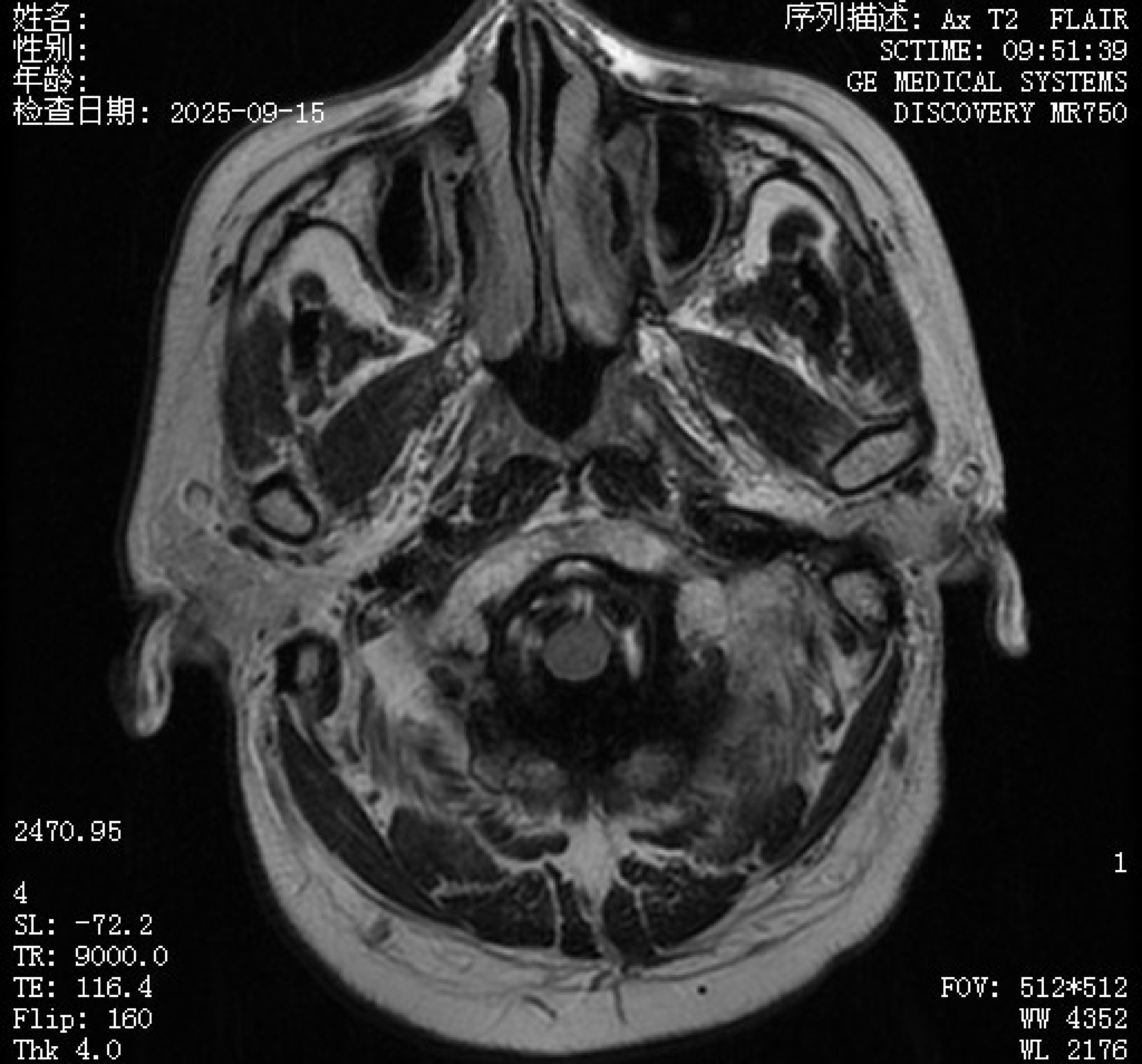
姓名:
性别:
年龄:
检查日期: 2025-09-15
序列描述: Ax T2 FLAIR
SCTIME: 09:51:39
GE MEDICAL SYSTEMS
DISCOVERY MR750
2470.95
1
4
SL: -72.2
TR: 9000.0
TE: 116.4
Flip: 160
Thk 4.0
FOV: 512*512
WW 4352
WL 2176

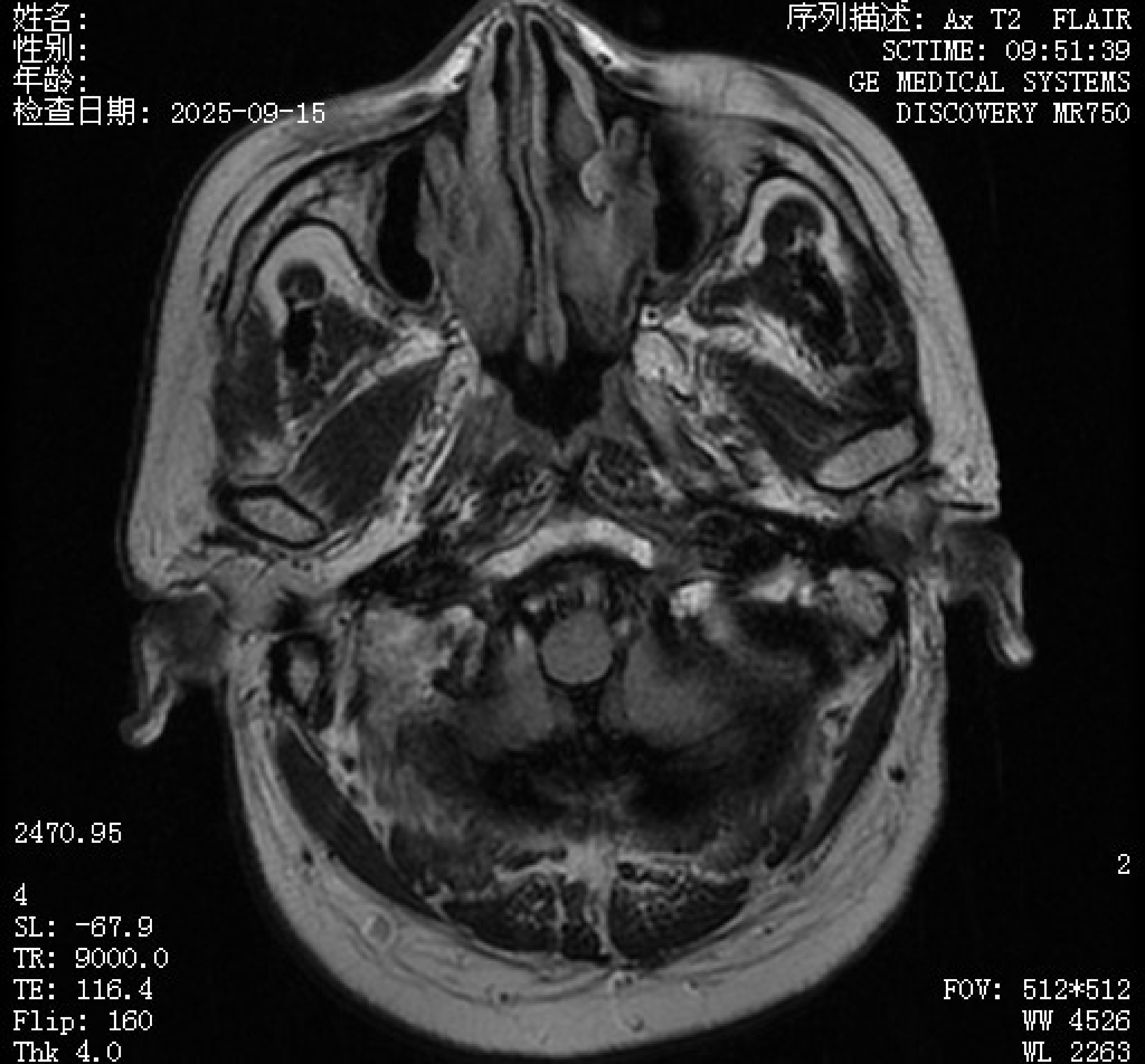
姓名:
性别:
年龄:
检查日期: 2025-09-15
序列描述: Ax T2 FLAIR
SCTIME: 09:51:39
GE MEDICAL SYSTEMS
DISCOVERY MR750
2470.95
2
4
SL: -67.9
TR: 9000.0
TE: 116.4
Flip: 160
Thk 4.0
FOV: 512*512
WW 4526
WL 2263

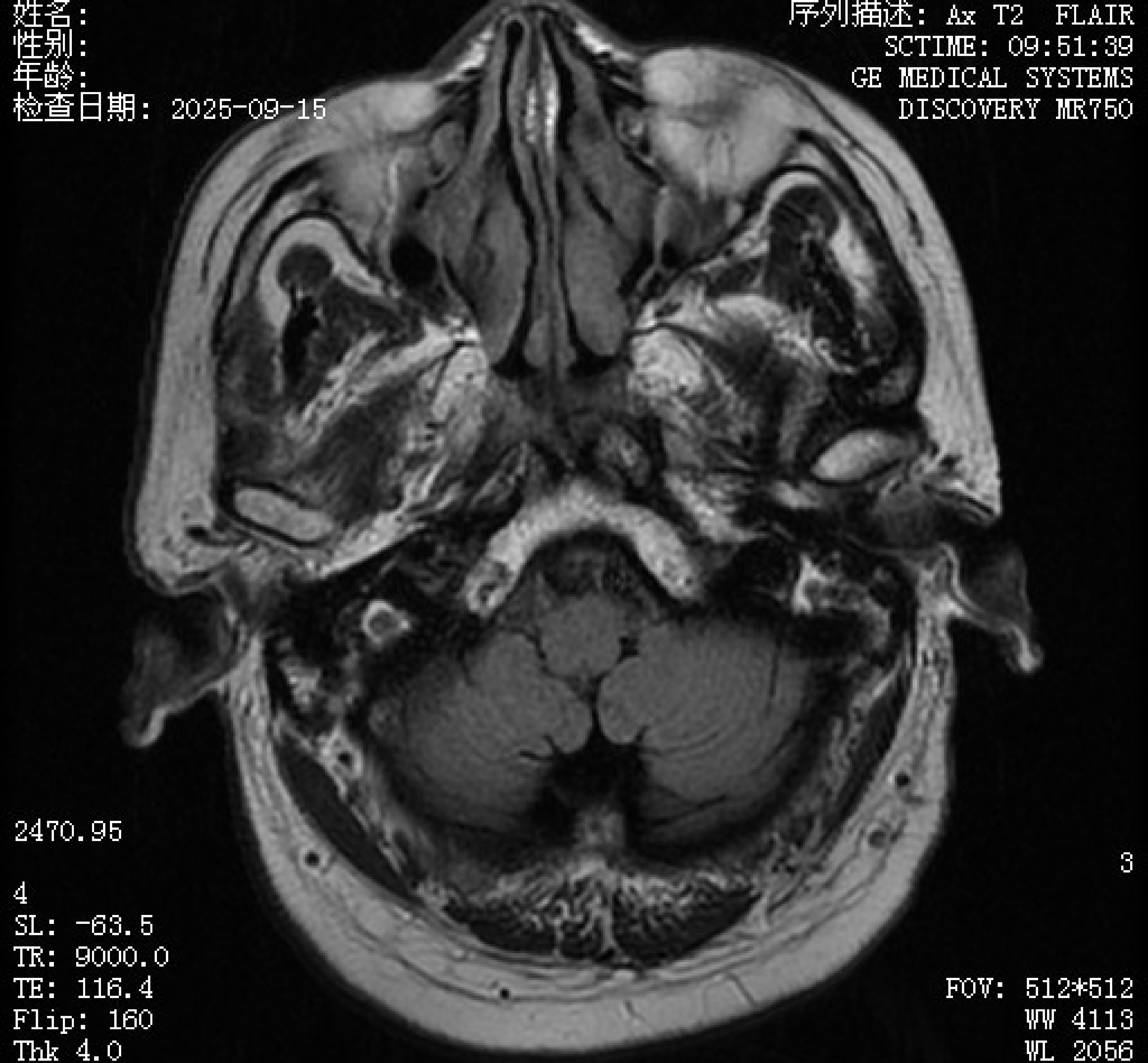

姓名:
性别:
年龄:
检查日期: 2025-09-15
序列描述: Ax T2 FLAIR
SCTIME: 09:51:39
GE MEDICAL SYSTEMS
DISCOVERY MR750
2470.95
3
4
SL: -63.5
TR: 9000.0
TE: 116.4
Flip: 160
Thk 4.0
FOV: 512*512
WW 4113
WL 2056

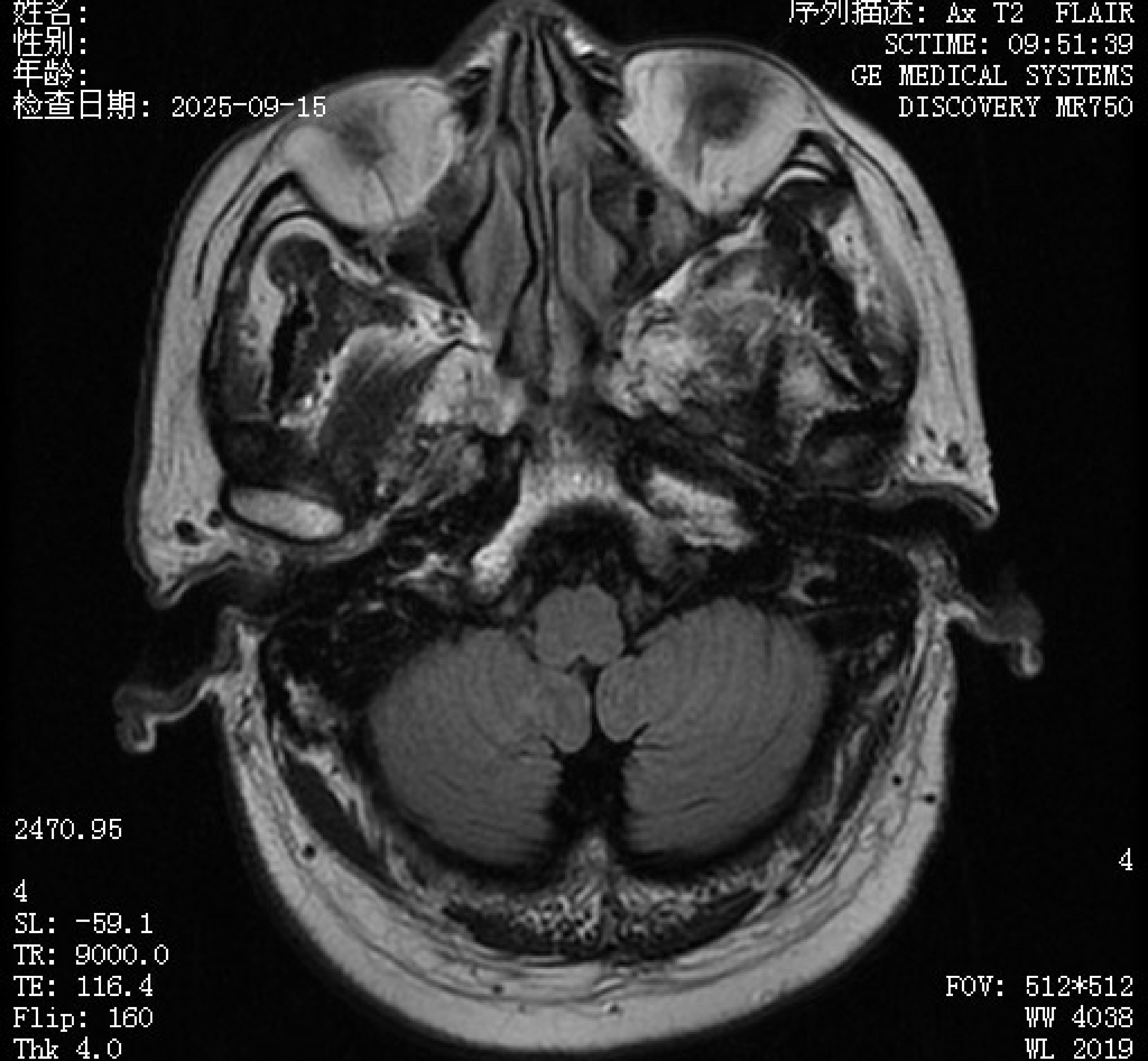

姓名:
性别:
年龄:
检查日期: 2025-09-15
序列描述: Ax T2 FLAIR
SCTIME: 09:51:39
GE MEDICAL SYSTEMS
DISCOVERY MR750
2470.95
4
SL: -59.1
TR: 9000.0
TE: 116.4
Flip: 160
Thk 4.0
4
FOV: 512*512
WW 4038
WL 2019

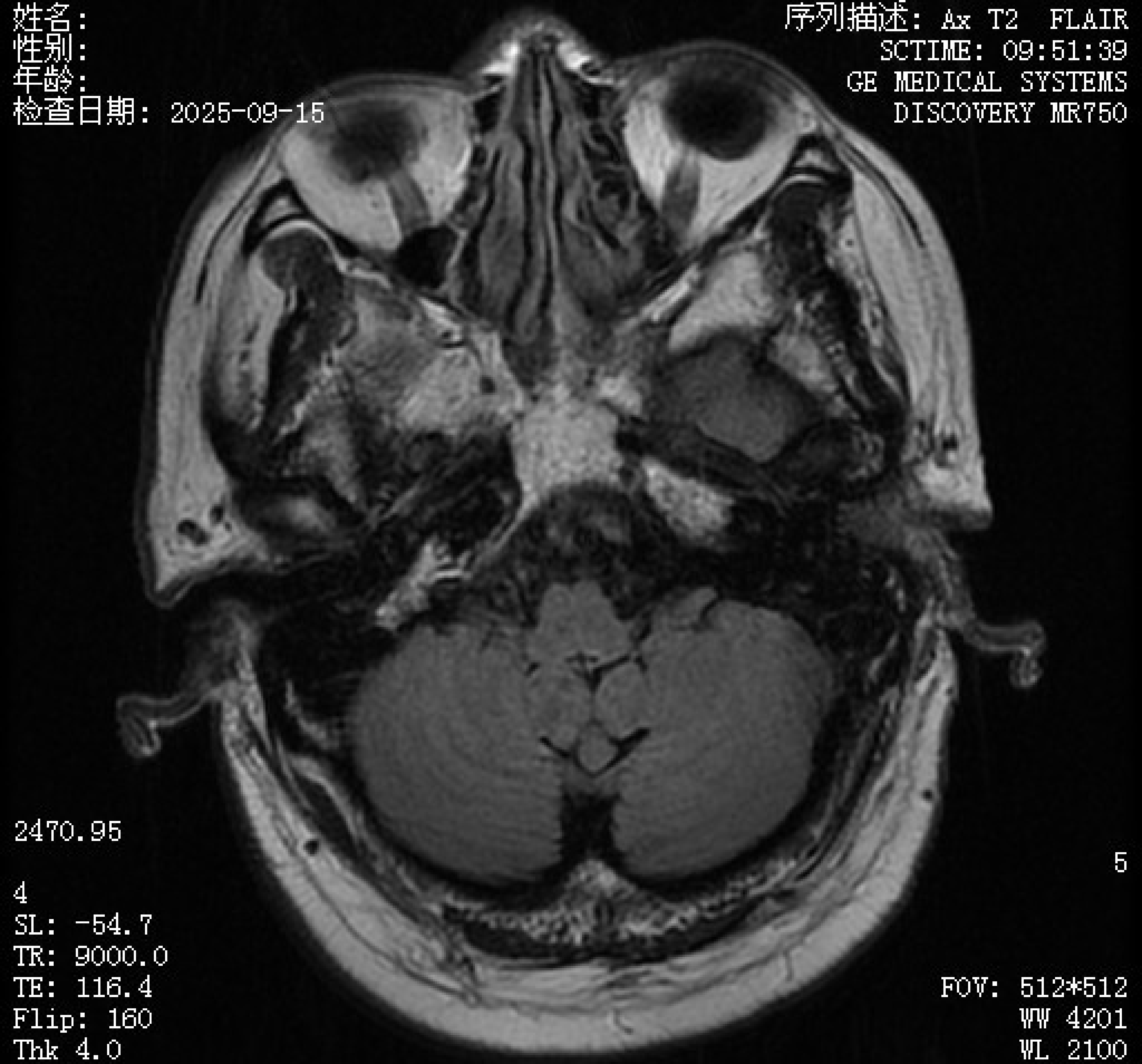
姓名:
性别:
年龄:
检查日期: 2025-09-15
序列描述: Ax T2 FLAIR
SCTIME: 09:51:39
GE MEDICAL SYSTEMS
DISCOVERY MR750
2470.95
5
4
SL: -54.7
TR: 9000.0
TE: 116.4
Flip: 160
Thk 4.0
FOV: 512*512
WW 4201
WL 2100

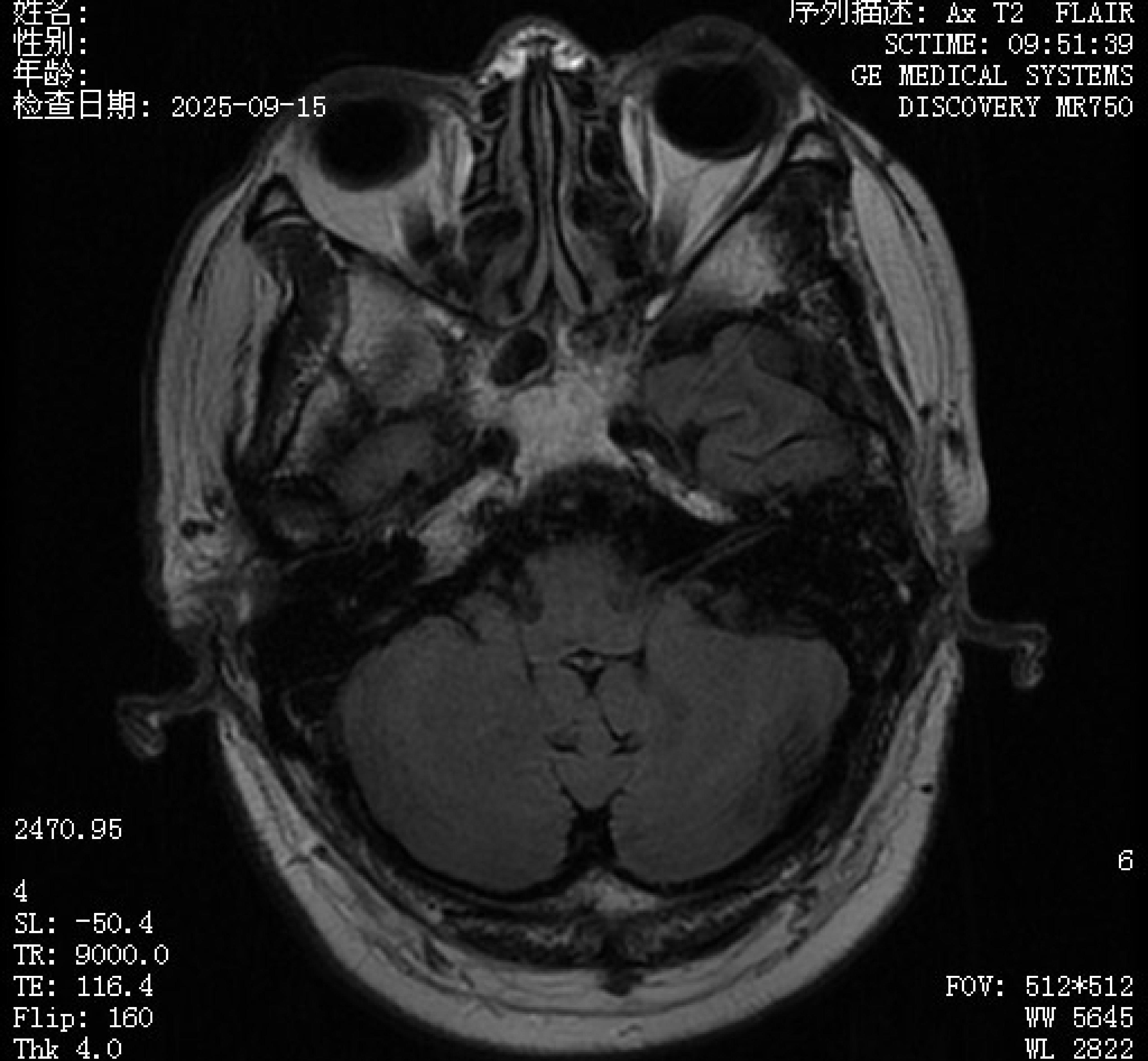
姓名:
性别:
年龄:
检查日期: 2025-09-15
序列描述: Ax T2 FLAIR
SCTIME: 09:51:39
GE MEDICAL SYSTEMS
DISCOVERY MR750
2470.95
6
4
SL: -50.4
TR: 9000.0
TE: 116.4
Flip: 160
Thk 4.0
FOV: 512*512
WW 5645
WL 2822

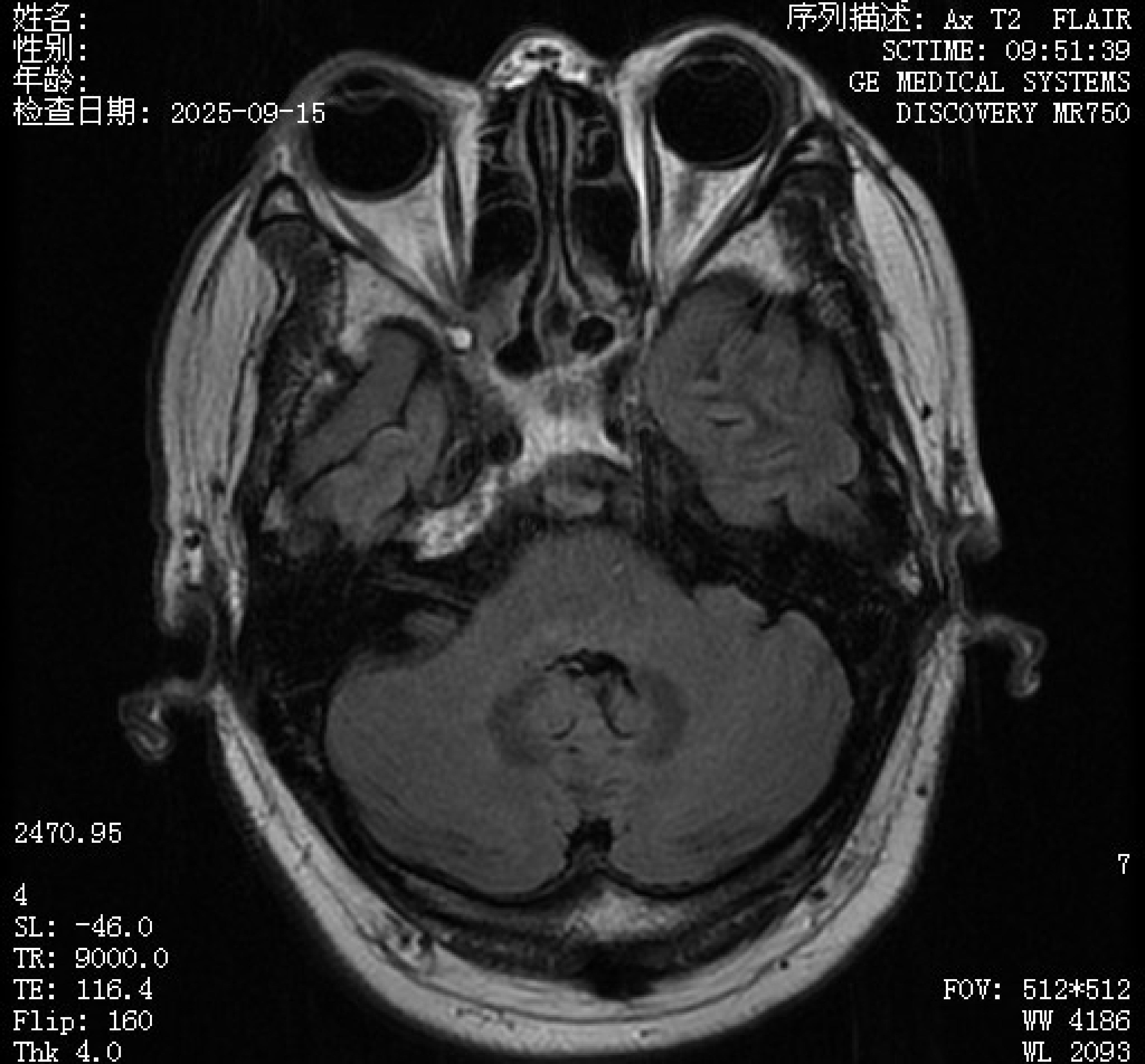
姓名:
性别:
年龄:
检查日期: 2025-09-15
序列描述: Ax T2 FLAIR
SCTIME: 09:51:39
GE MEDICAL SYSTEMS
DISCOVERY MR750
2470.95
7
4
SL: -46.0
TR: 9000.0
TE: 116.4
Flip: 160
Thk 4.0
FOV: 512*512
WW 4186
WL 2093

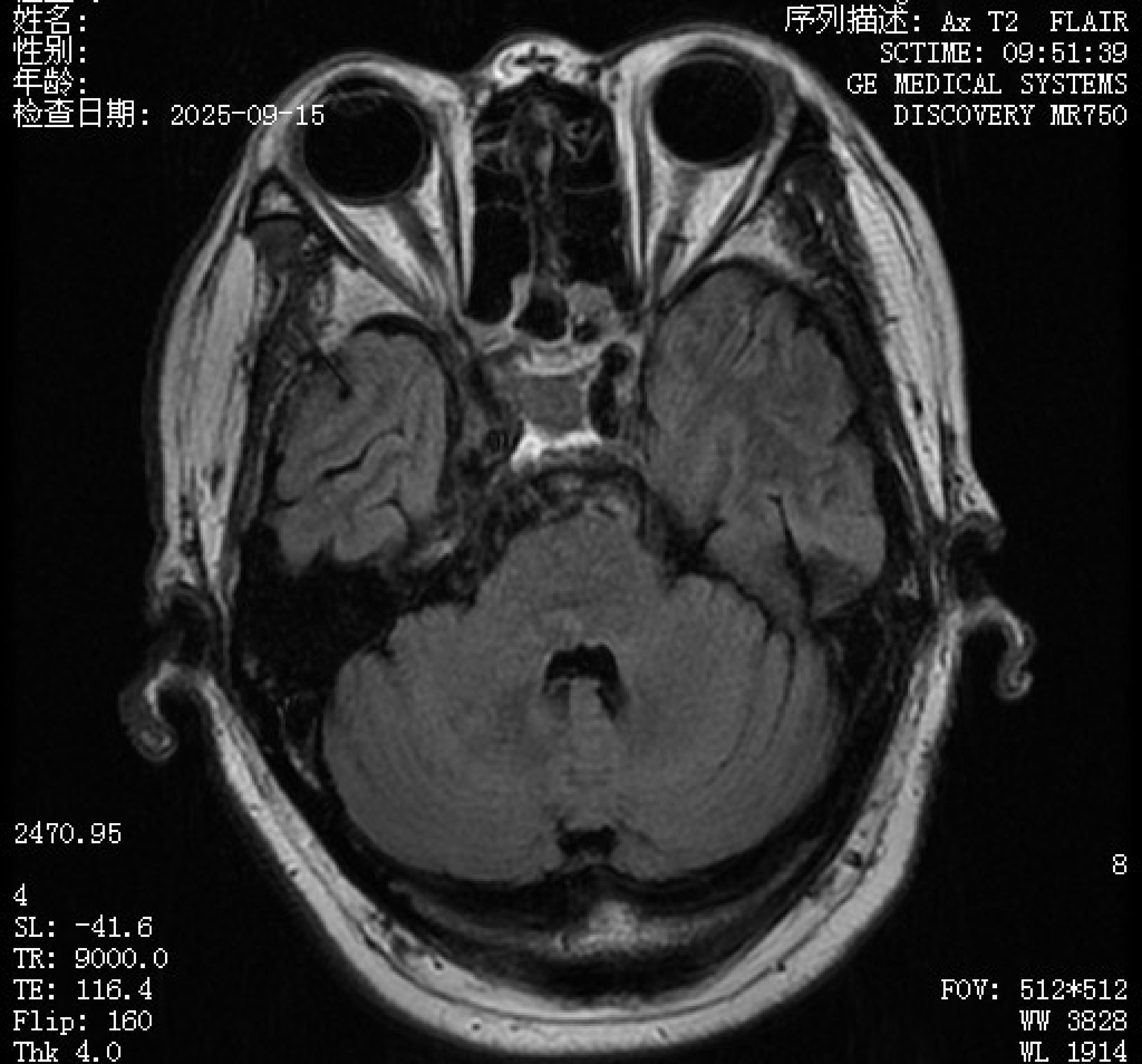

姓名:
性别:
年龄:
检查日期: 2025-09-15
序列描述: Ax T2 FLAIR
SCTIME: 09:51:39
GE MEDICAL SYSTEMS
DISCOVERY MR750
2470.95
8
4
SL: -41.6
TR: 9000.0
TE: 116.4
Flip: 160
Thk 4.0
FOV: 512*512
WW 3828
WL 1914

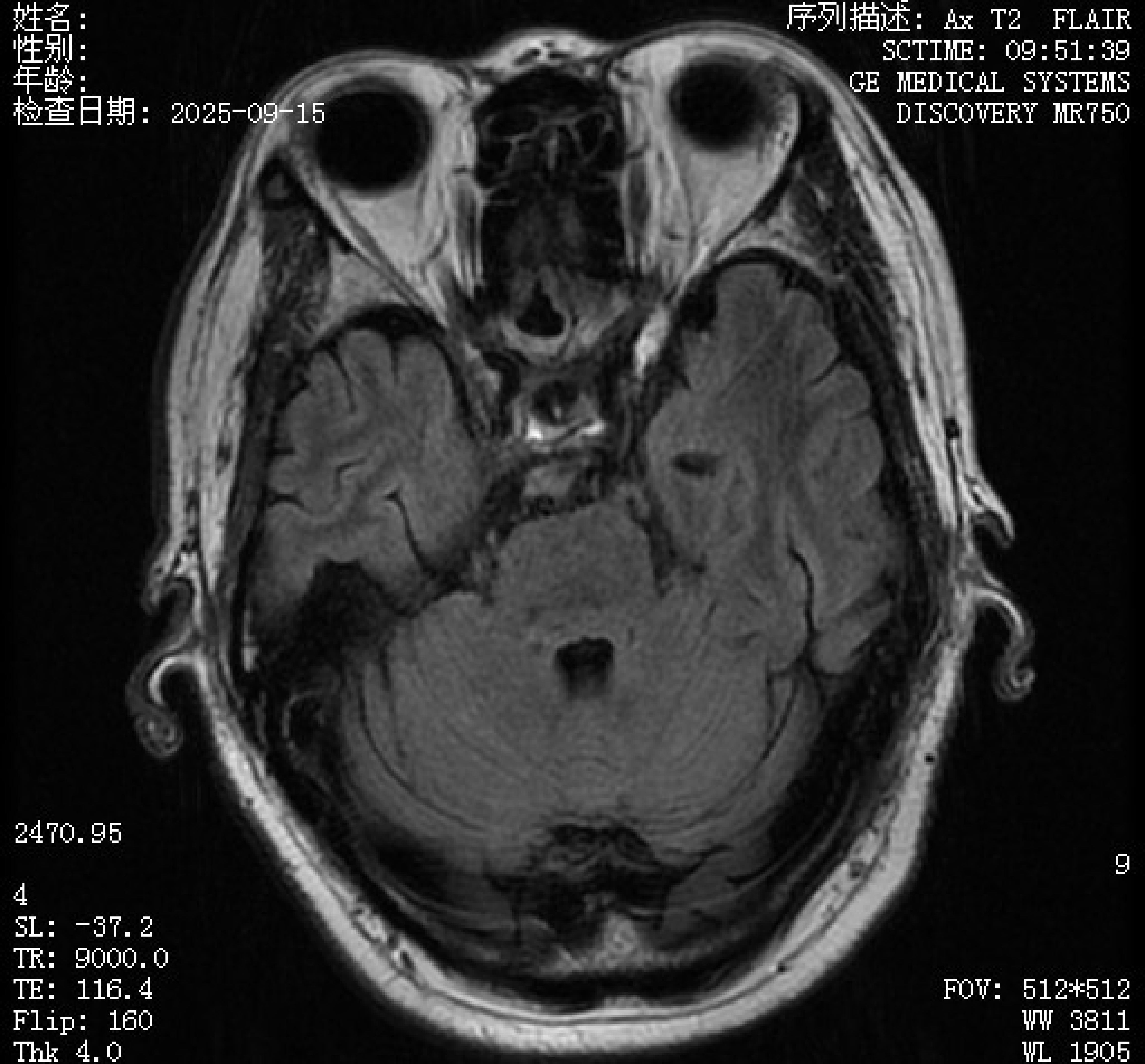
姓名:
性别:
年龄:
检查日期: 2025-09-15
序列描述: Ax T2 FLAIR
SCTIME: 09:51:39
GE MEDICAL SYSTEMS
DISCOVERY MR750
2470.95
9
4
SL: -37.2
TR: 9000.0
TE: 116.4
Flip: 160
Thk 4.0
FOV: 512*512
WW 3811
WL 1905

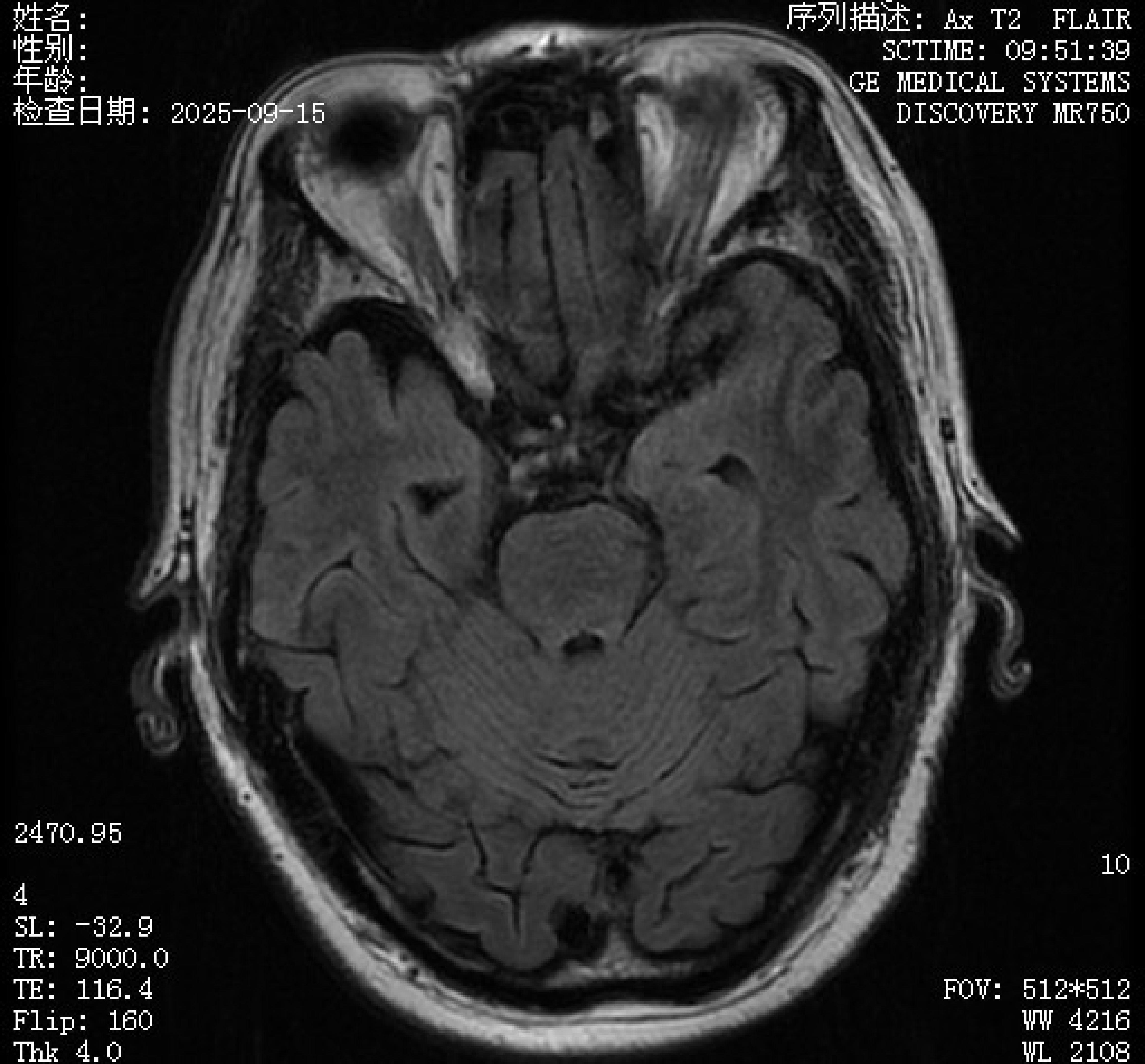
姓名:
性别:
年龄:
检查日期: 2025-09-15
序列描述: Ax T2 FLAIR
SCTIME: 09:51:39
GE MEDICAL SYSTEMS
DISCOVERY MR750
2470.95
4
SL: -32.9
TR: 9000.0
TE: 116.4
Flip: 160
Thk 4.0
10
FOV: 512*512
WW 4216
WL 2108

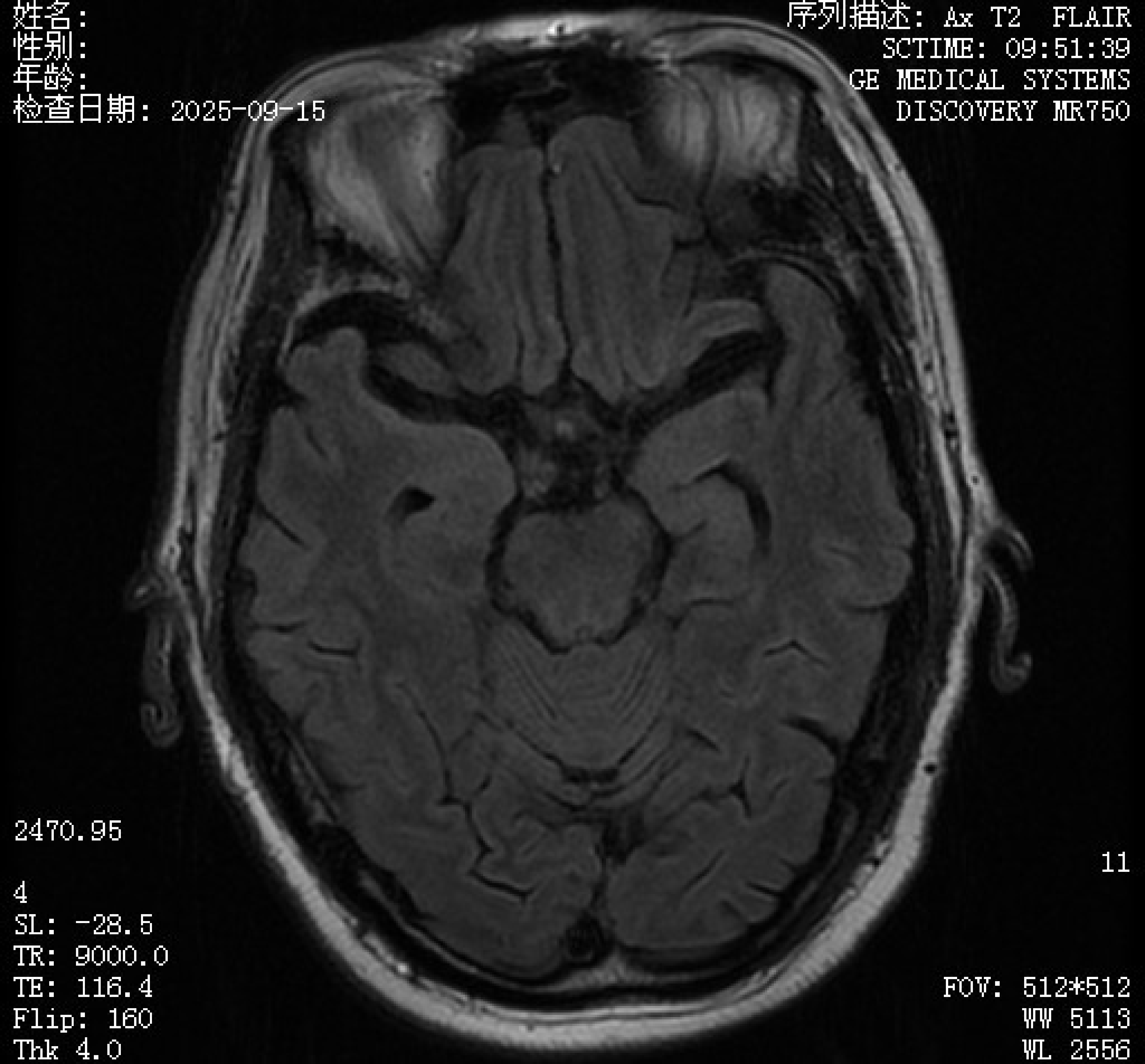
姓名:
性别:
年龄:
检查日期: 2025-09-15
序列描述: Ax T2 FLAIR
SCTIME: 09:51:39
GE MEDICAL SYSTEMS
DISCOVERY MR750
2470.95
11
4
SL: -28.5
TR: 9000.0
TE: 116.4
Flip: 160
Thk 4.0
FOV: 512*512
WW 5113
WL 2556

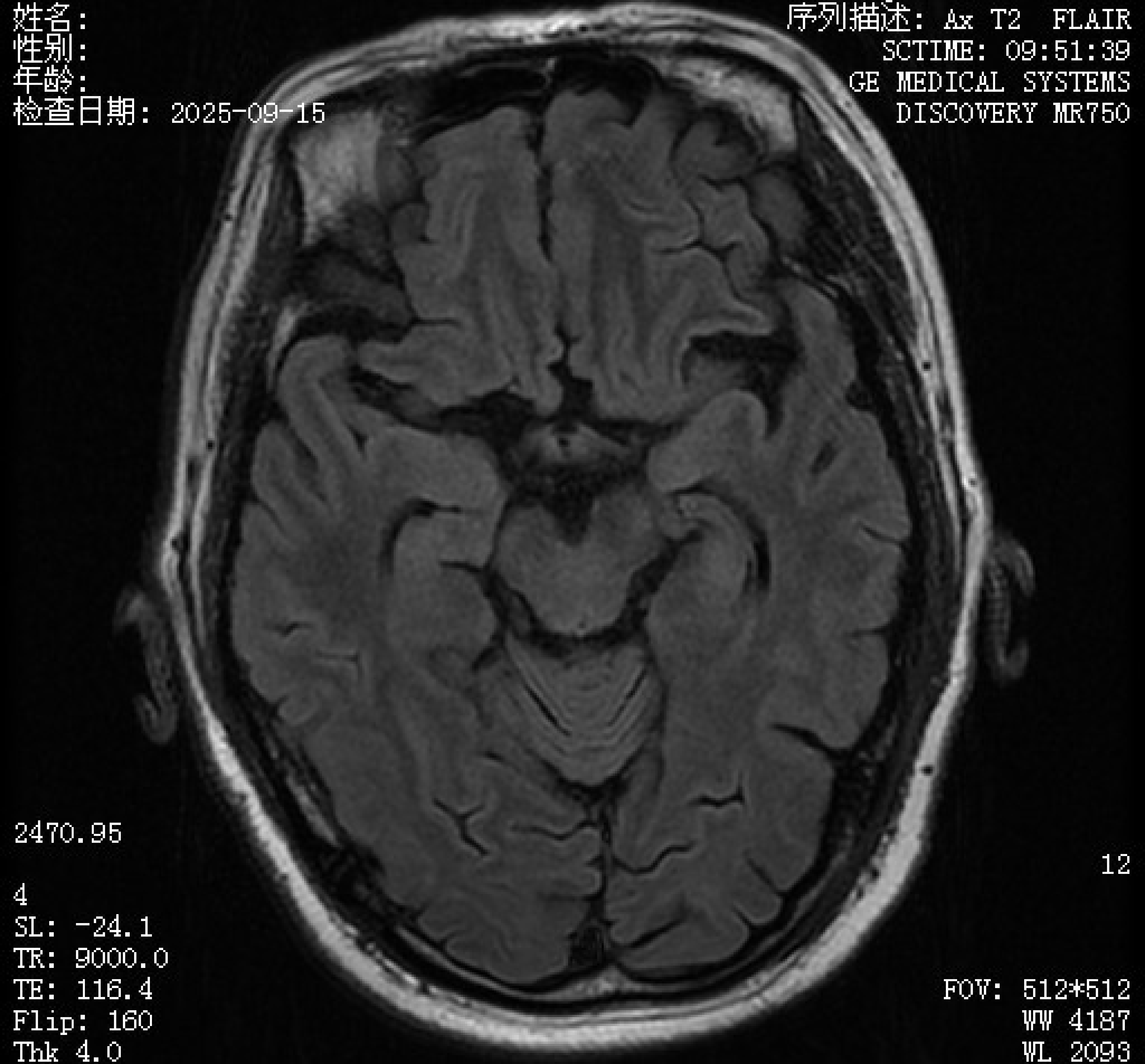
姓名:
性别:
年龄:
检查日期: 2025-09-15
序列描述: Ax T2 FLAIR
SCTIME: 09:51:39
GE MEDICAL SYSTEMS
DISCOVERY MR750
2470.95
4
SL: -24.1
TR: 9000.0
TE: 116.4
Flip: 160
Thk 4.0
12
FOV: 512*512
WW 4187
WL 2093

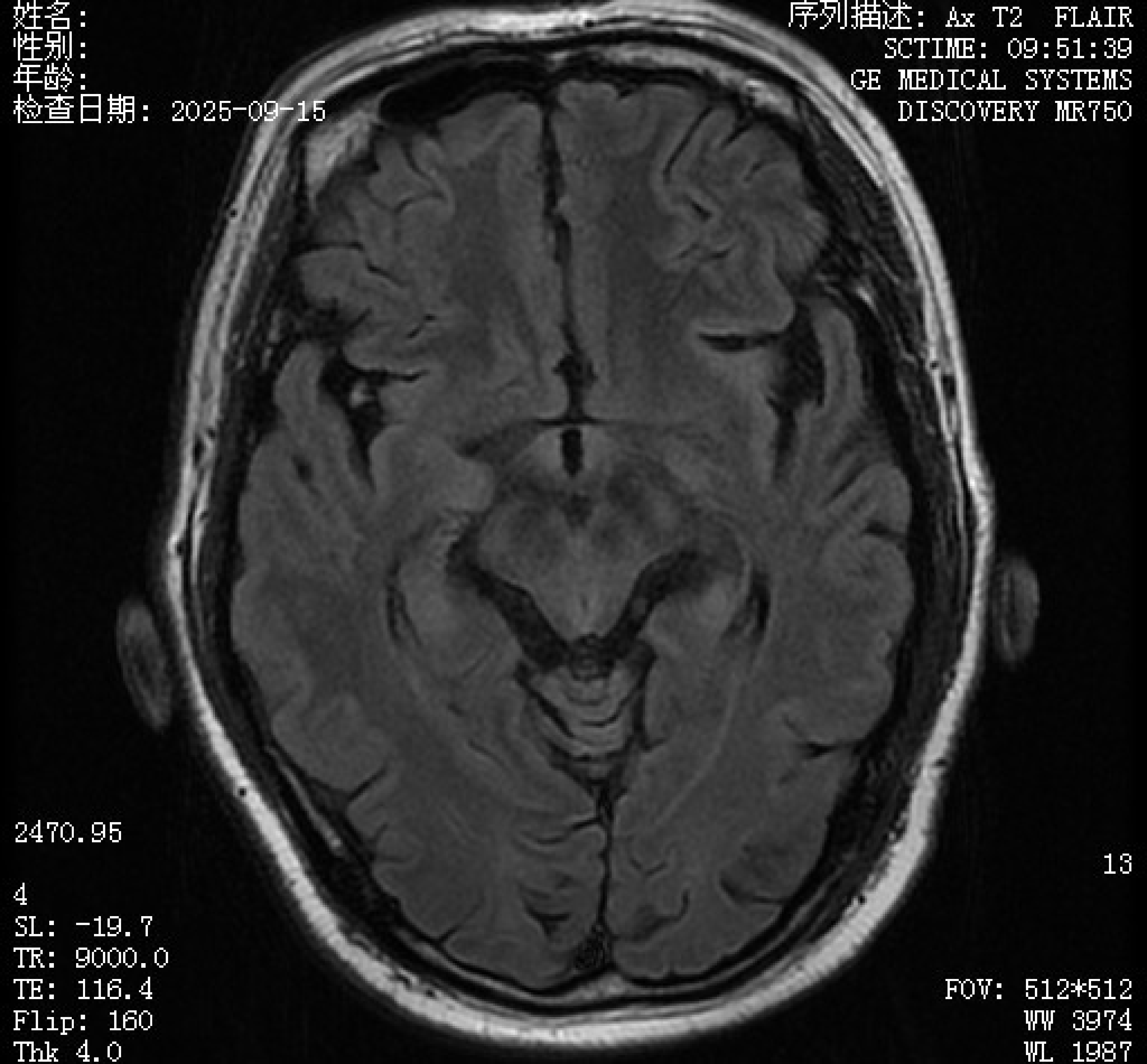

姓名:
性别:
年龄:
检查日期: 2025-09-15
序列描述: Ax T2 FLAIR
SCTIME: 09:51:39
GE MEDICAL SYSTEMS
DISCOVERY MR750
2470.95
13
4
SL: -19.7
TR: 9000.0
TE: 116.4
Flip: 160
Thk 4.0
FOV: 512*512
WW 3974
WL 1987

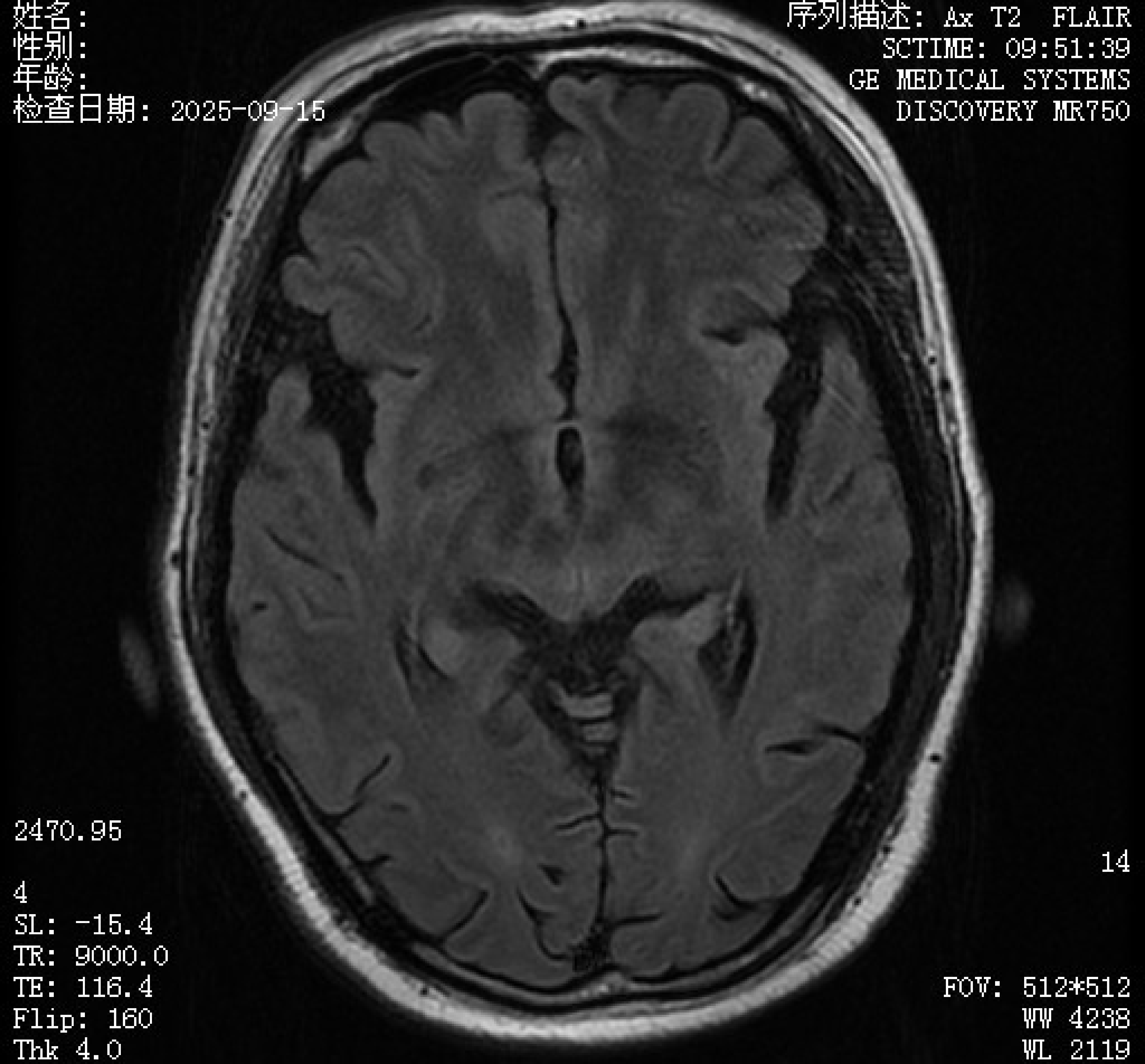

姓名:
性别:
年龄:
检查日期: 2025-09-15
序列描述: Ax T2 FLAIR
SCTIME: 09:51:39
GE MEDICAL SYSTEMS
DISCOVERY MR750
2470.95
4
SL: -15.4
TR: 9000.0
TE: 116.4
Flip: 160
Thk 4.0
14
FOV: 512*512
WW 4238
WL 2119

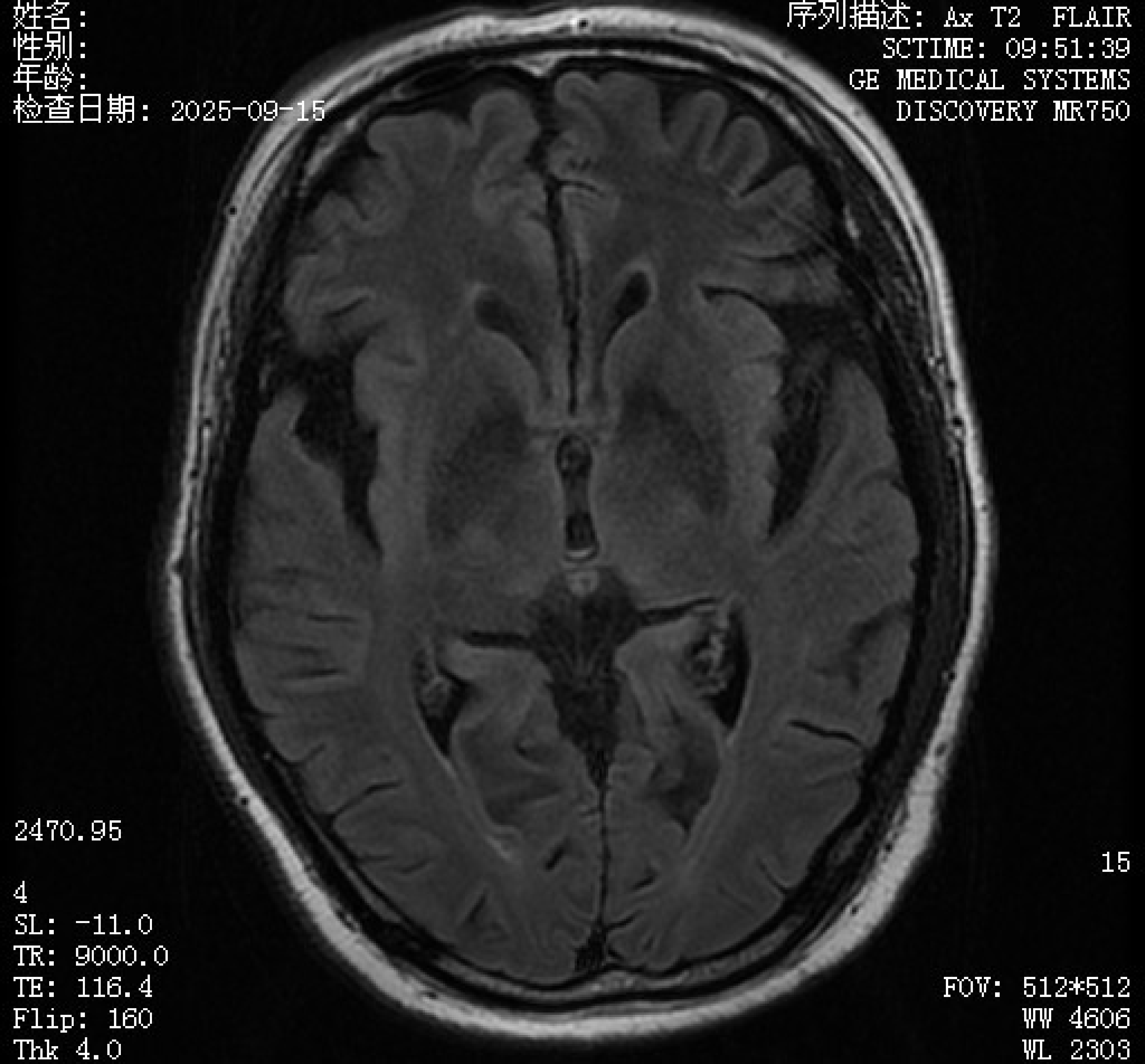
姓名:
性别:
年龄:
检查日期: 2025-09-15
序列描述: Ax T2 FLAIR
SCTIME: 09:51:39
GE MEDICAL SYSTEMS
DISCOVERY MR750
2470.95
4
SL: -11.0
TR: 9000.0
TE: 116.4
Flip: 160
Thk 4.0
15
FOV: 512*512
WW 4606
WL 2303

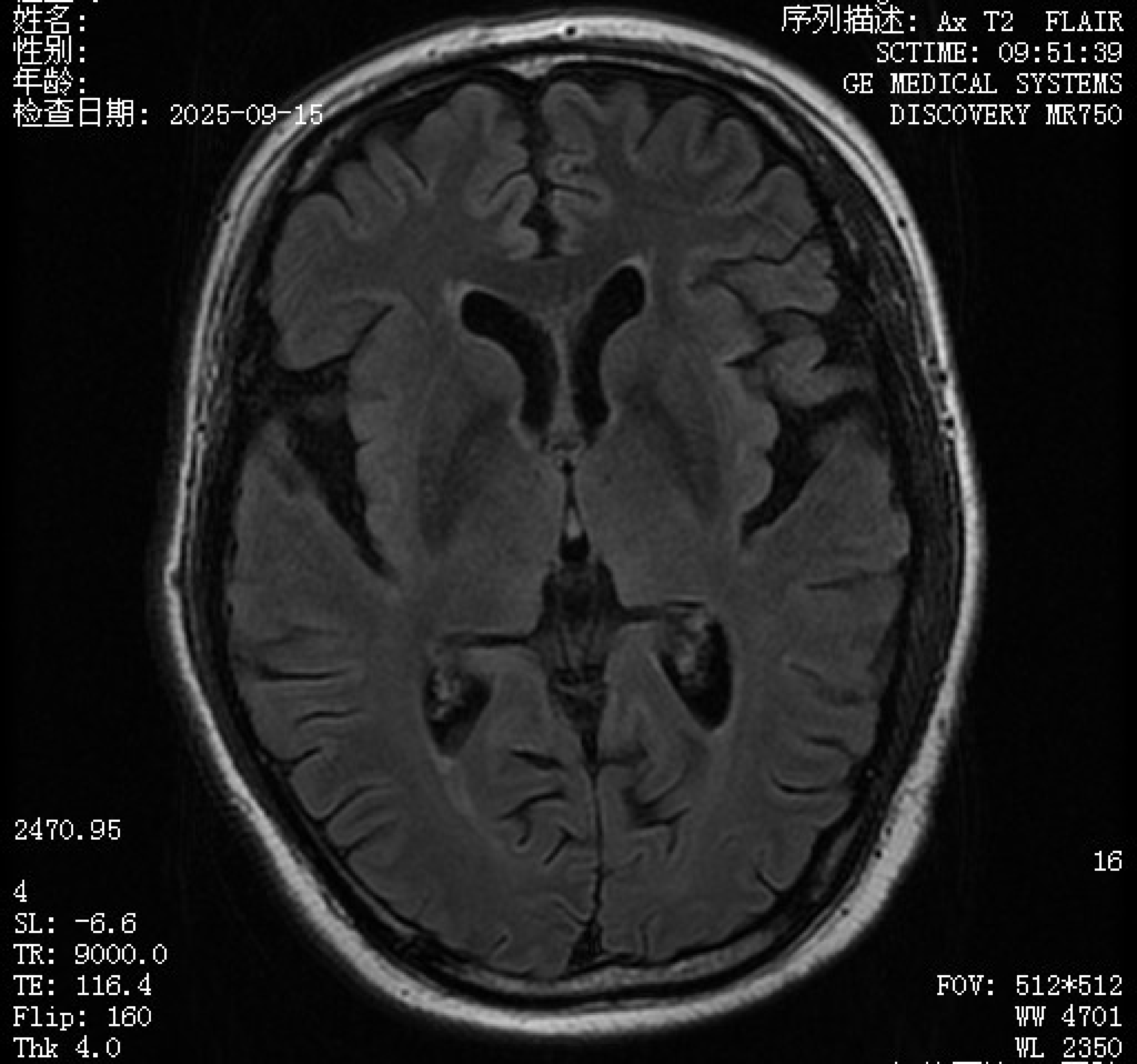

姓名:
性别:
年龄:
检查日期: 2025-09-15
序列描述: Ax T2 FLAIR
SCTIME: 09:51:39
GE MEDICAL SYSTEMS
DISCOVERY MR750
2470.95
16
4
SL: -6.6
TR: 9000.0
TE: 116.4
Flip: 160
Thk 4.0
FOV: 512*512
WW 4701
WL 2350

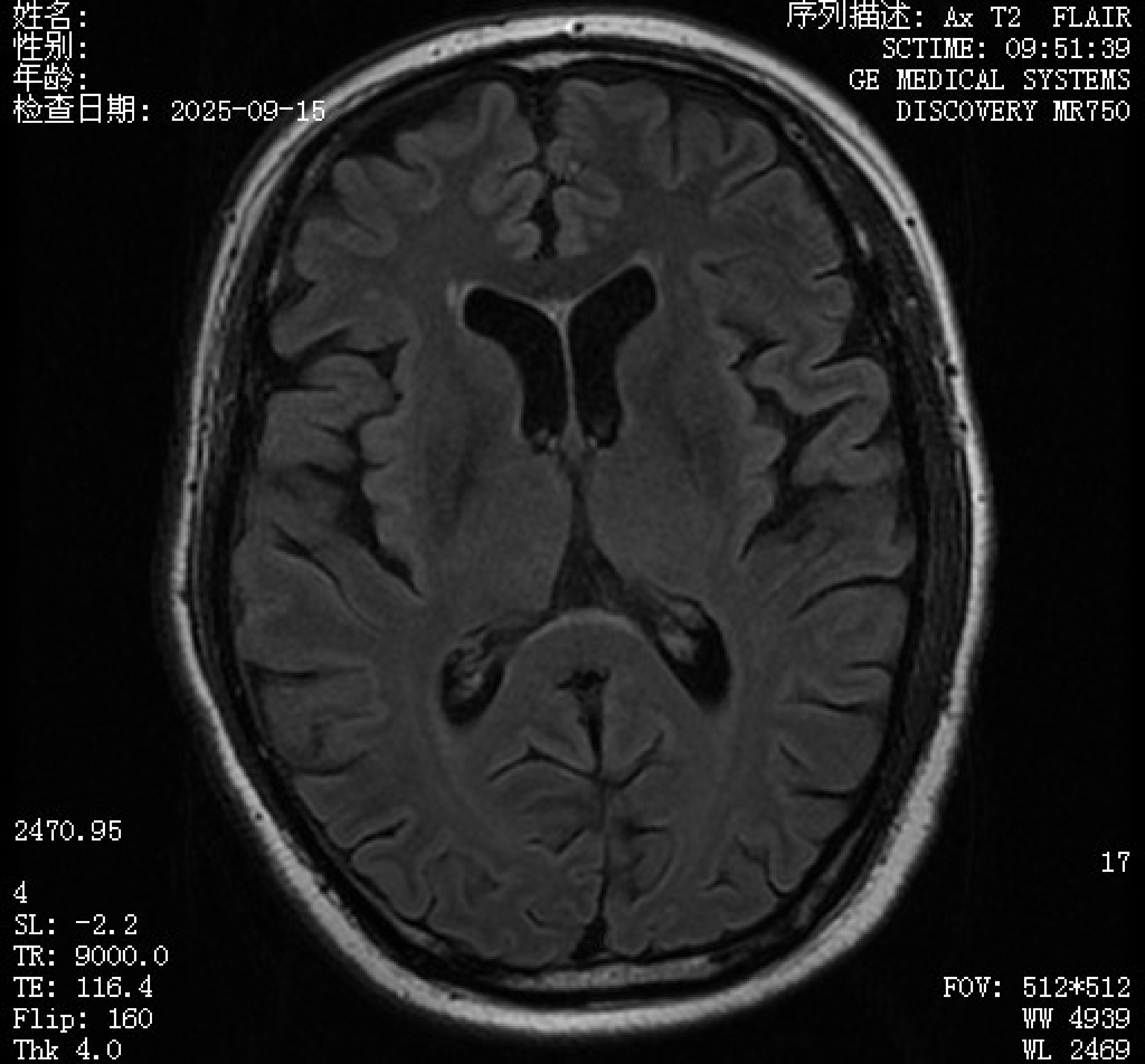
姓名:
性别:
年龄:
检查日期: 2025-09-15
序列描述: Ax T2 FLAIR
SCTIME: 09:51:39
GE MEDICAL SYSTEMS
DISCOVERY MR750
2470.95
4
SL: -2.2
TR: 9000.0
TE: 116.4
Flip: 160
Thk 4.0
17
FOV: 512*512
WW 4939
WL 2469

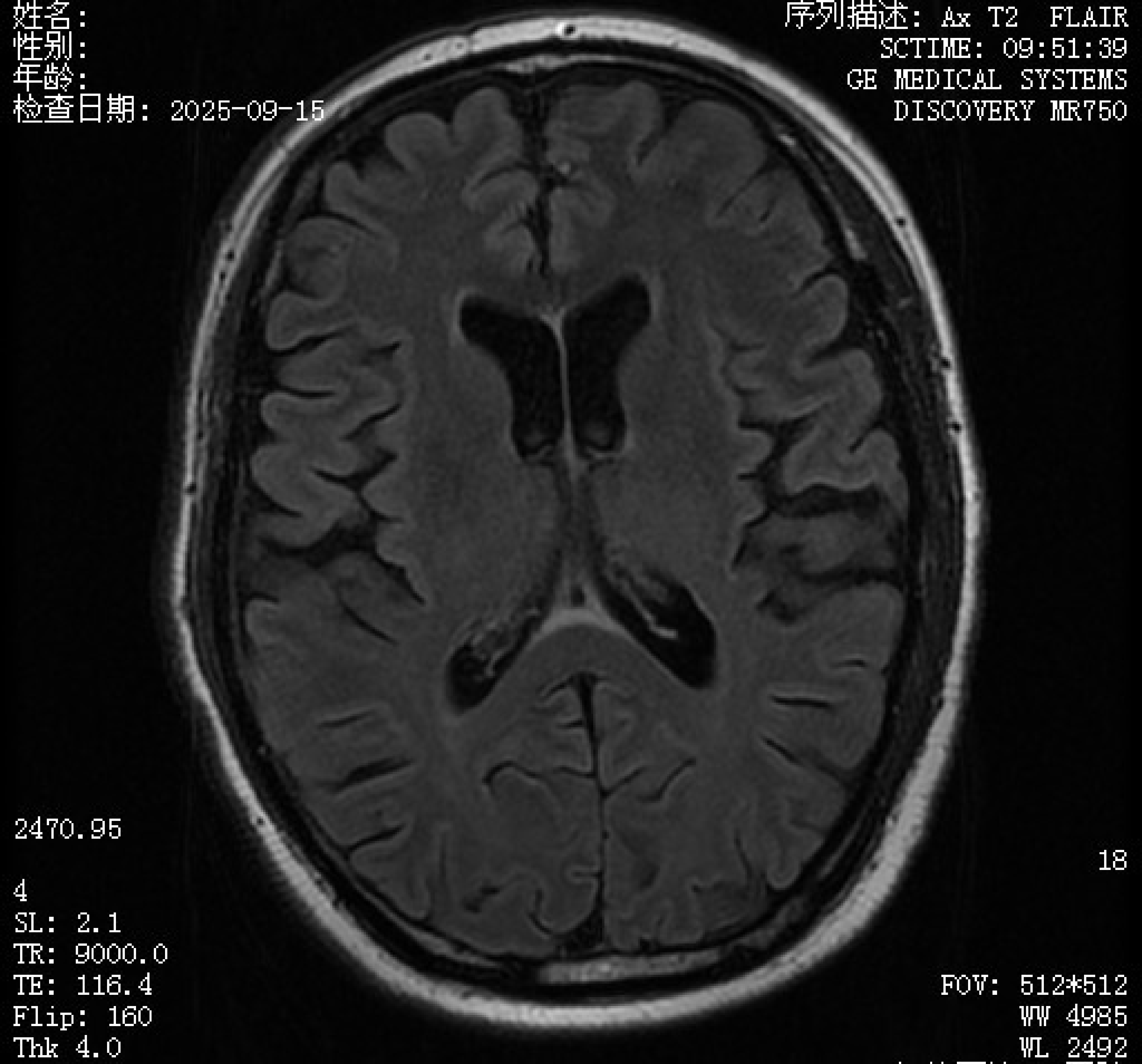

姓名:
性别:
年龄:
检查日期: 2025-09-15
序列描述: Ax T2 FLAIR
SCTIME: 09:51:39
GE MEDICAL SYSTEMS
DISCOVERY MR750
2470.95
18
4
SL: 2.1
TR: 9000.0
TE: 116.4
Flip: 160
Thk 4.0
FOV: 512*512
WW 4985
WL 2492

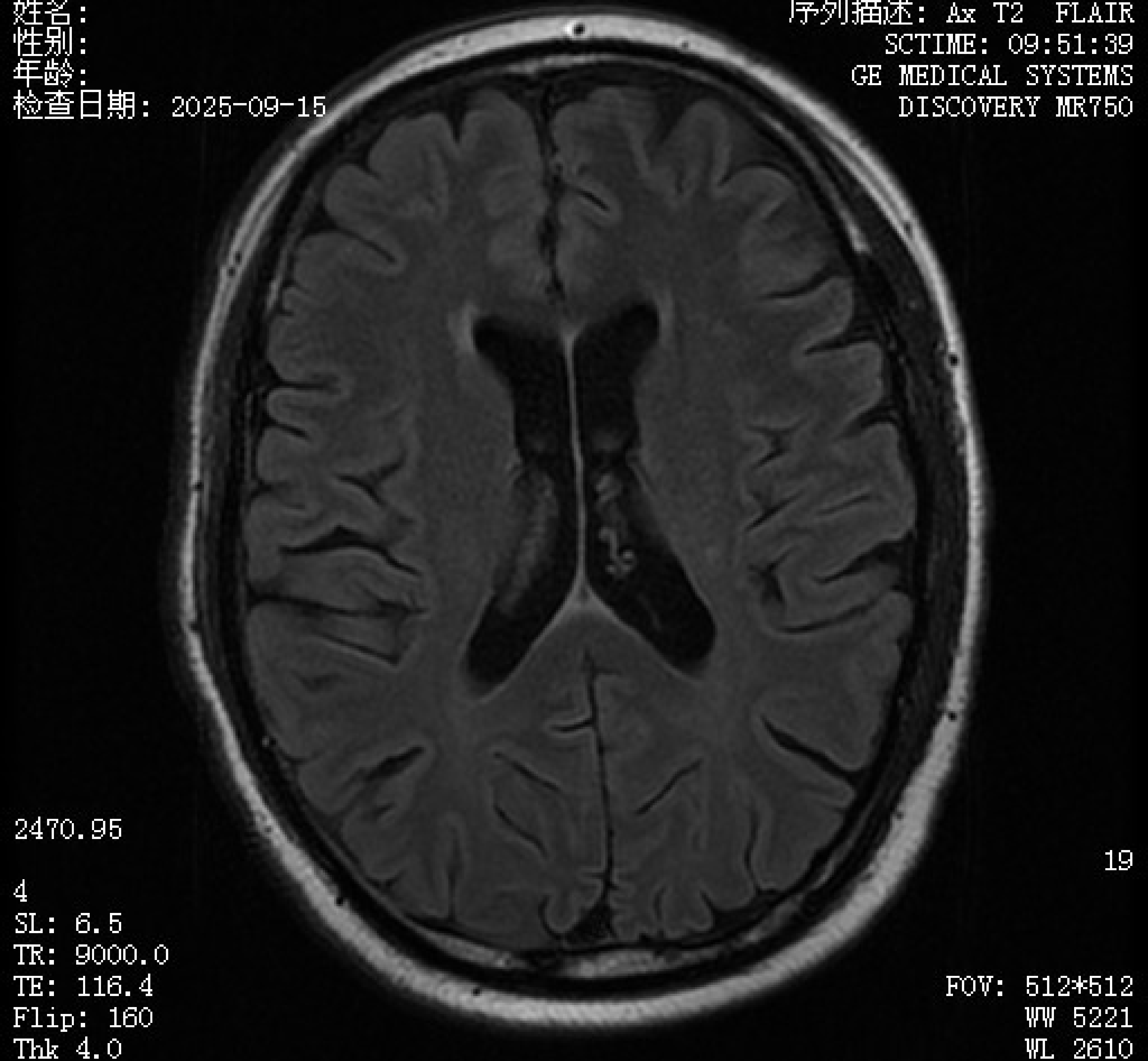
姓名:
性别:
年龄:
检查日期: 2025-09-15
序列描述: Ax T2 FLAIR
SCTIME: 09:51:39
GE MEDICAL SYSTEMS
DISCOVERY MR750
2470.95
19
4
SL: 6.5
TR: 9000.0
TE: 116.4
Flip: 160
Thk 4.0
FOV: 512*512
WW 5221
WL 2610

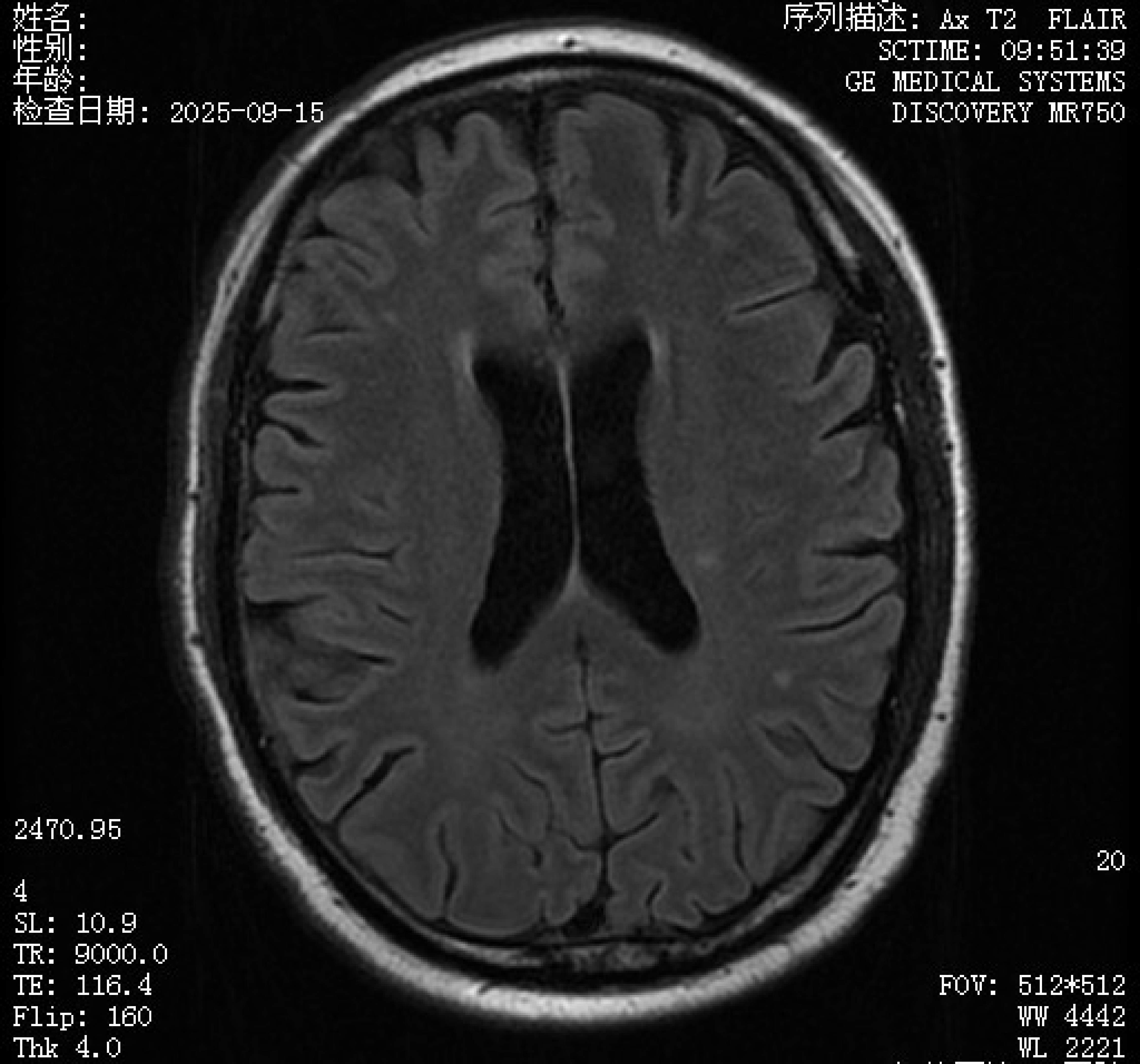
姓名:
性别:
年龄:
检查日期: 2025-09-15
序列描述: Ax T2 FLAIR
SCTIME: 09:51:39
GE MEDICAL SYSTEMS
DISCOVERY MR750
2470.95
4
SL: 10.9
TR: 9000.0
TE: 116.4
Flip: 160
Thk 4.0
20
FOV: 512*512
WW 4442
WL 2221

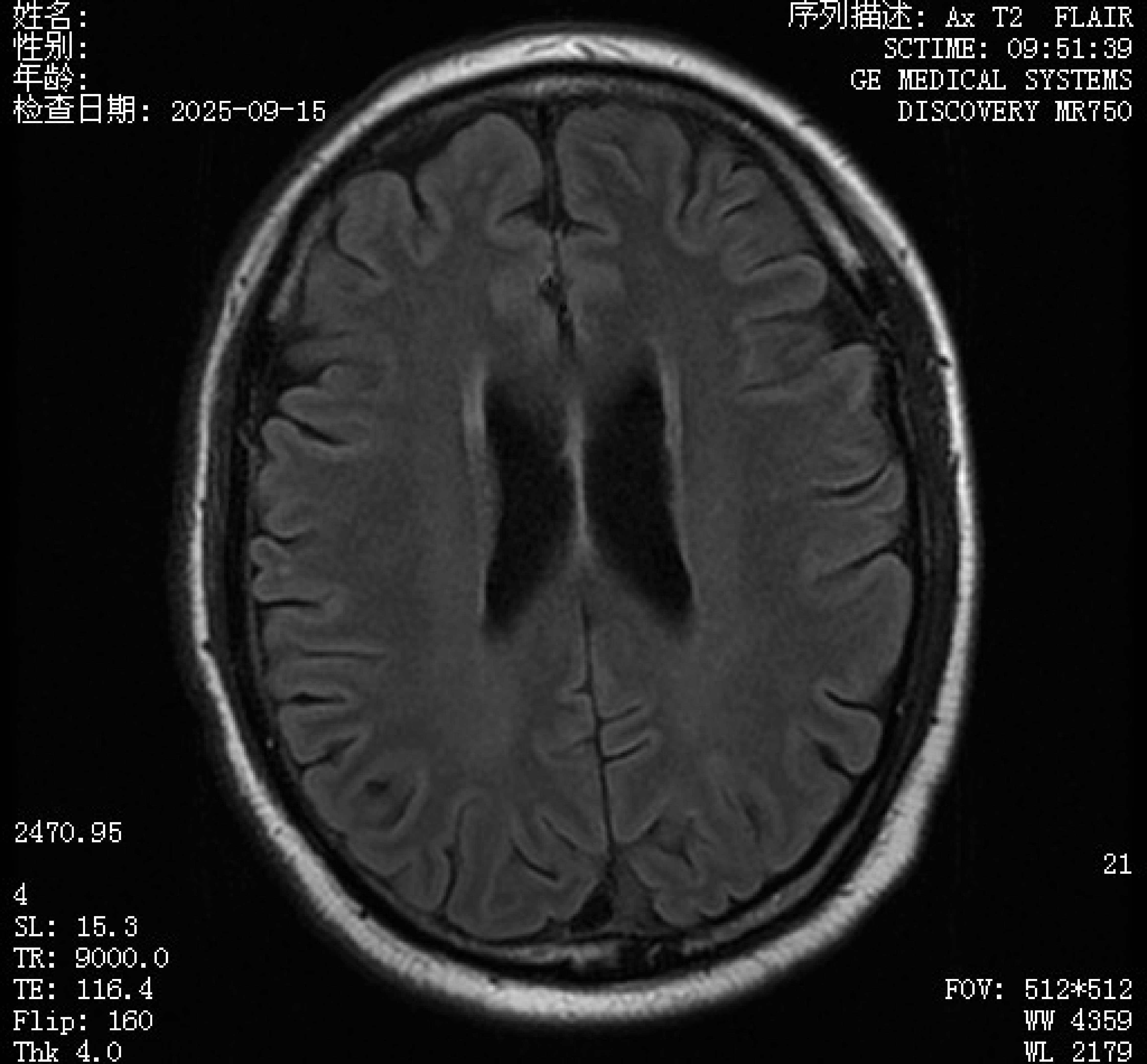
姓名:
性别:
年龄:
检查日期: 2025-09-15
序列描述: Ax T2 FLAIR
SCTIME: 09:51:39
GE MEDICAL SYSTEMS
DISCOVERY MR750
2470.95
21
4
SL: 15.3
TR: 9000.0
TE: 116.4
Flip: 160
Thk 4.0
FOV: 512*512
WW 4359
WL 2179

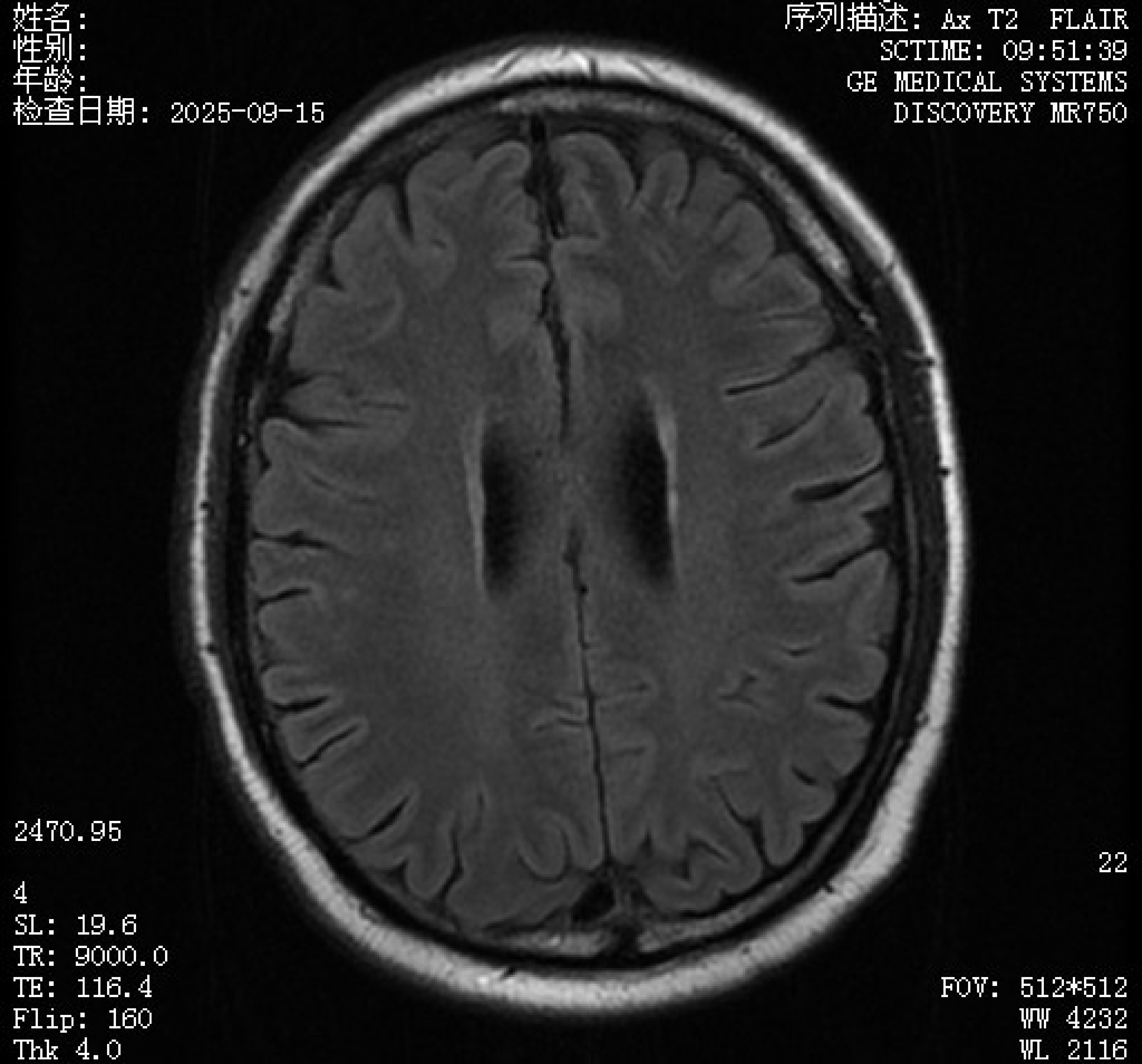
姓名:
性别:
年龄:
检查日期: 2025-09-15
序列描述: Ax T2 FLAIR
SCTIME: 09:51:39
GE MEDICAL SYSTEMS
DISCOVERY MR750
2470.95
22
4
SL: 19.6
TR: 9000.0
TE: 116.4
Flip: 160
Thk 4.0
FOV: 512*512
WW 4232
WL 2116

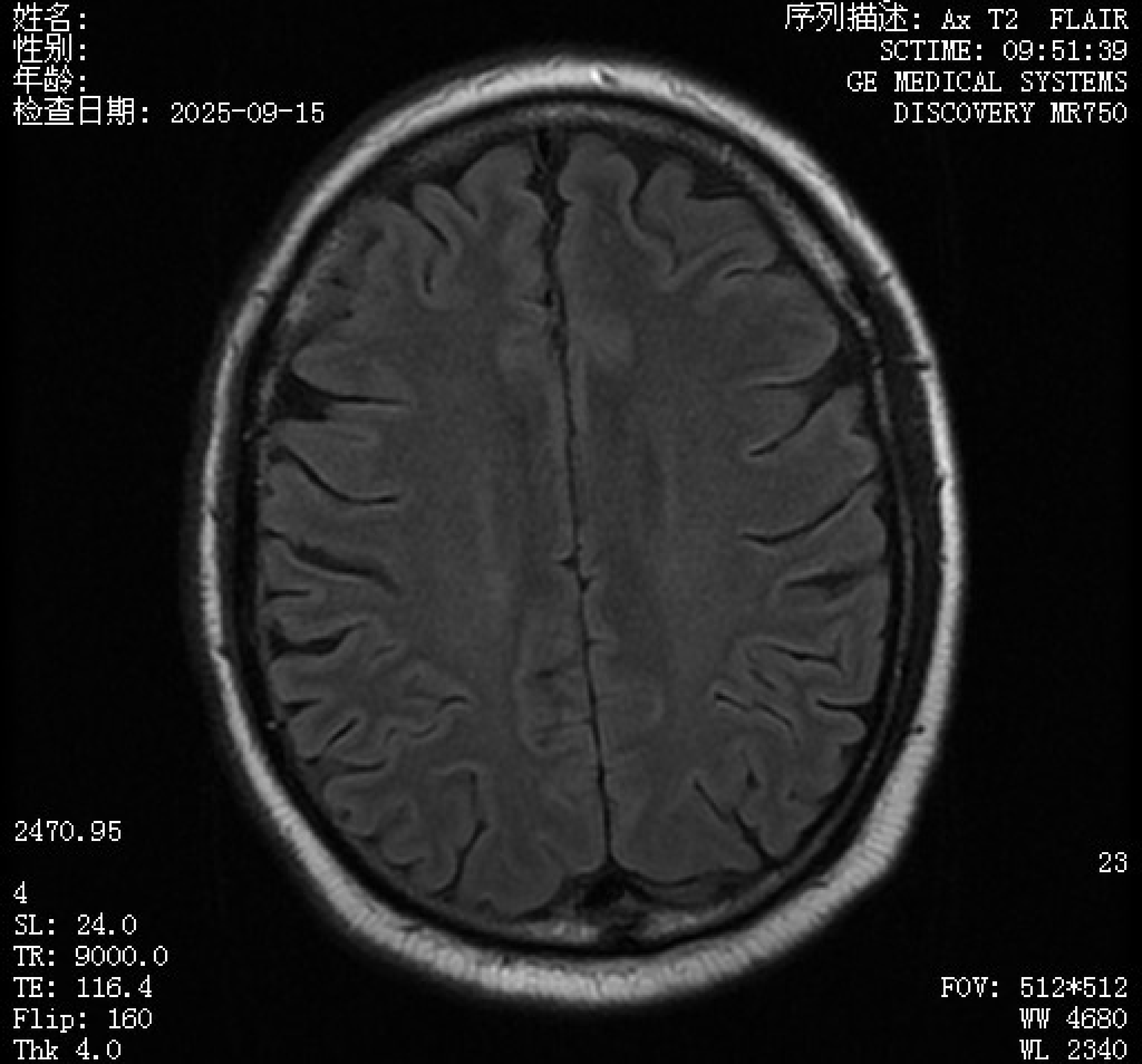
姓名:
性别:
年龄:
检查日期: 2025-09-15
序列描述: Ax T2 FLAIR
SCTIME: 09:51:39
GE MEDICAL SYSTEMS
DISCOVERY MR750
2470.95
4
SL: 24.0
TR: 9000.0
TE: 116.4
Flip: 160
Thk 4.0
23
FOV: 512*512
WW 4680
WL 2340

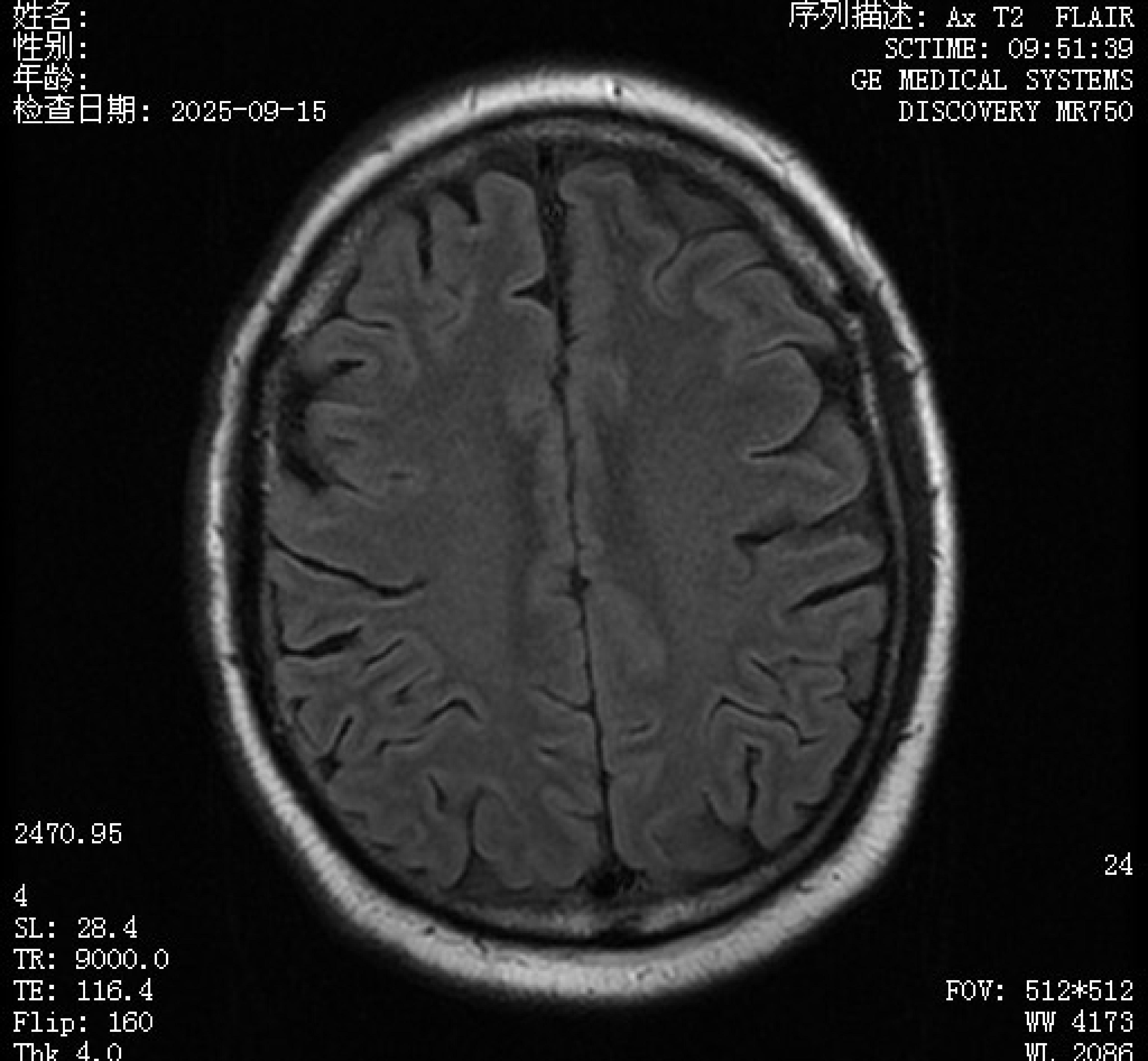
姓名:
性别:
年龄:
检查日期: 2025-09-15
序列描述: Ax T2 FLAIR
SCTIME: 09:51:39
GE MEDICAL SYSTEMS
DISCOVERY MR750
2470.95
24
4
SL: 28.4
TR: 9000.0
TE: 116.4
Flip: 160
Thk 4.0
FOV: 512*512
WW 4173
WL 2086

姓名:
性别:
年龄:
检查日期: 2025-09-15

序列描述: Ax T2 FLAIR
SCTIME: 09:51:39
GE MEDICAL SYSTEMS
DISCOVERY MR750

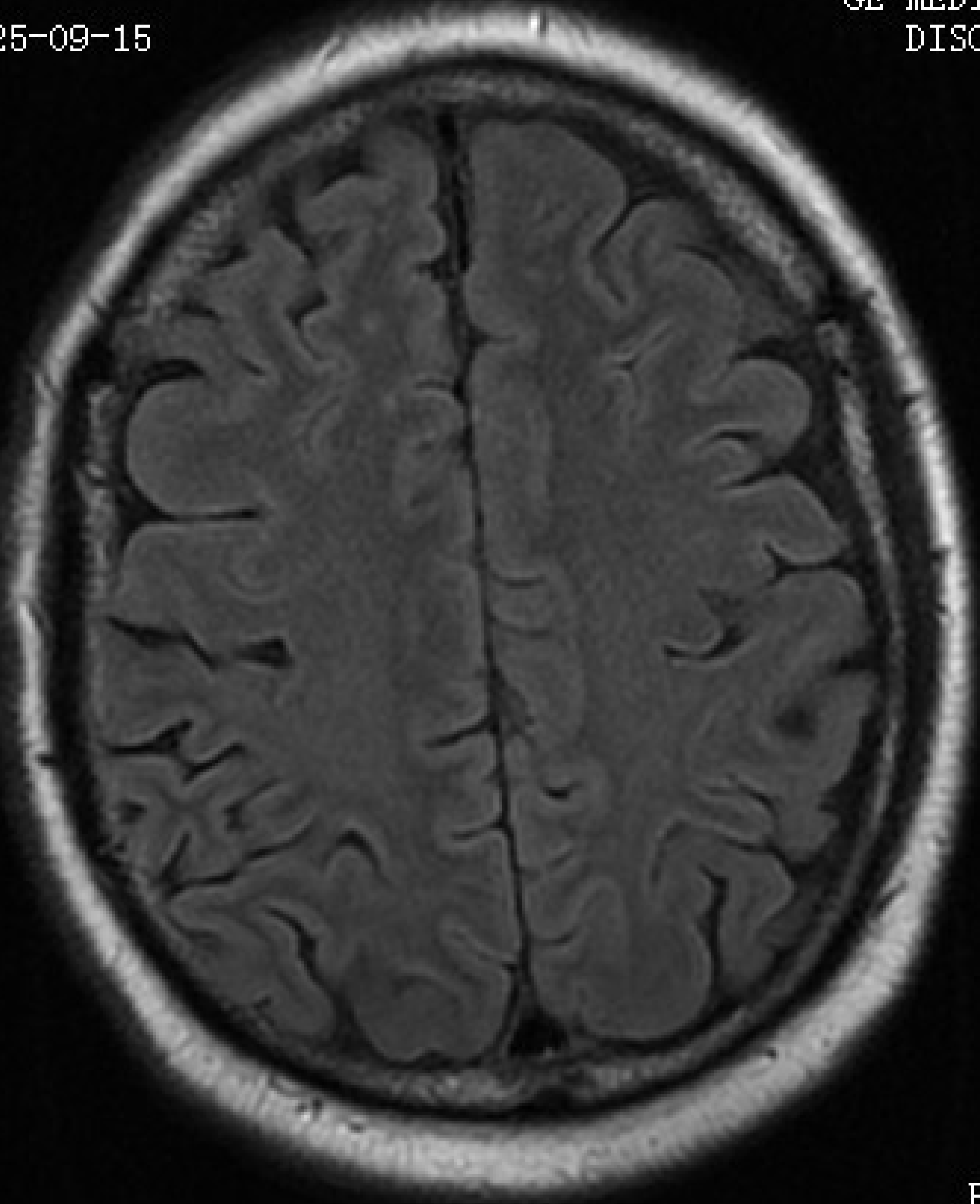

2470.95

25

4
SL: 32.8
TR: 9000.0
TE: 116.4
Flip: 160
Thk 4.0

FOV: 512*512
WW 4188
WL 2094

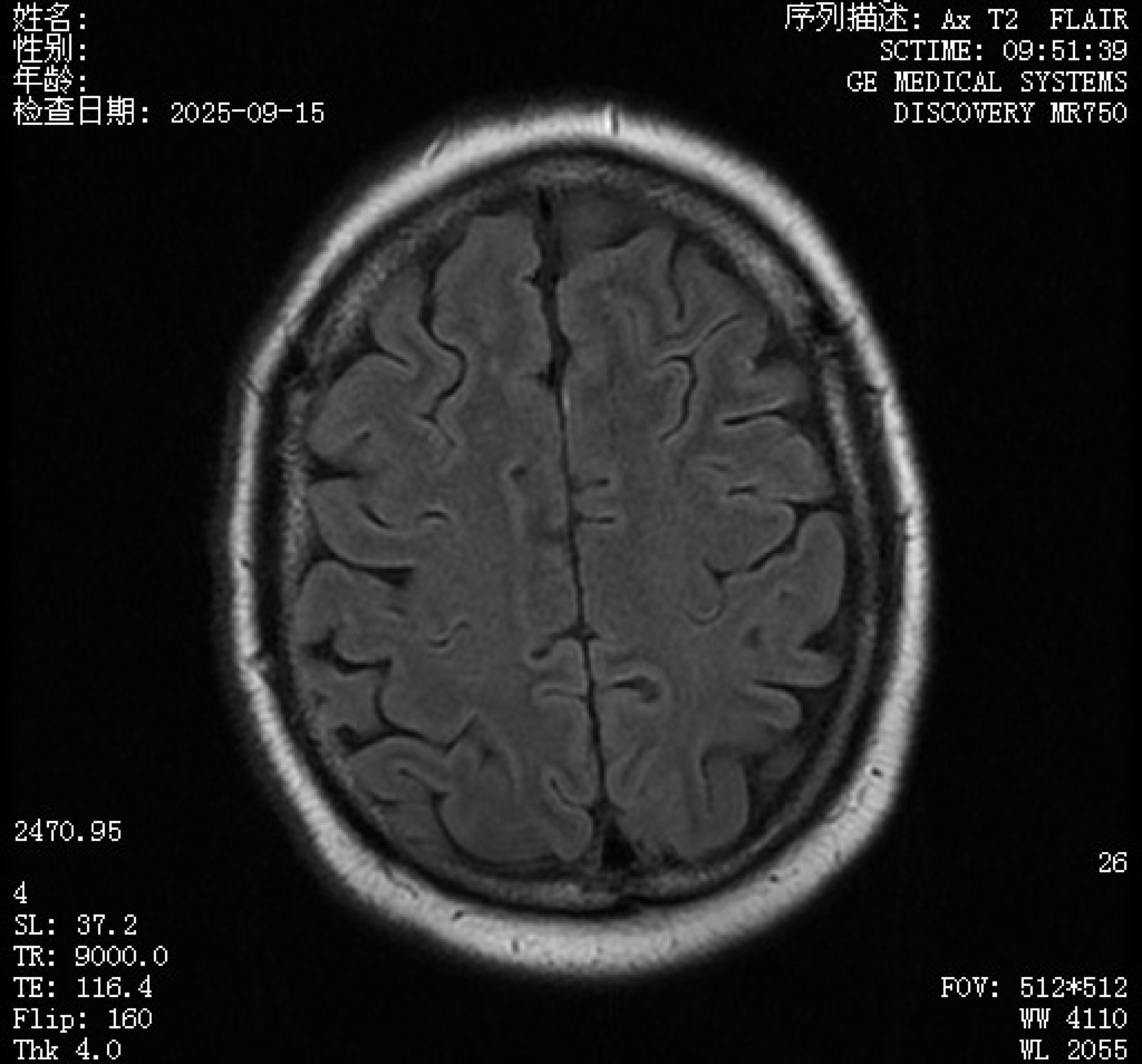
姓名:
性别:
年龄:
检查日期: 2025-09-15
序列描述: Ax T2 FLAIR
SCTIME: 09:51:39
GE MEDICAL SYSTEMS
DISCOVERY MR750
2470.95
26
4
SL: 37.2
TR: 9000.0
TE: 116.4
Flip: 160
Thk 4.0
FOV: 512*512
WW 4110
WL 2055

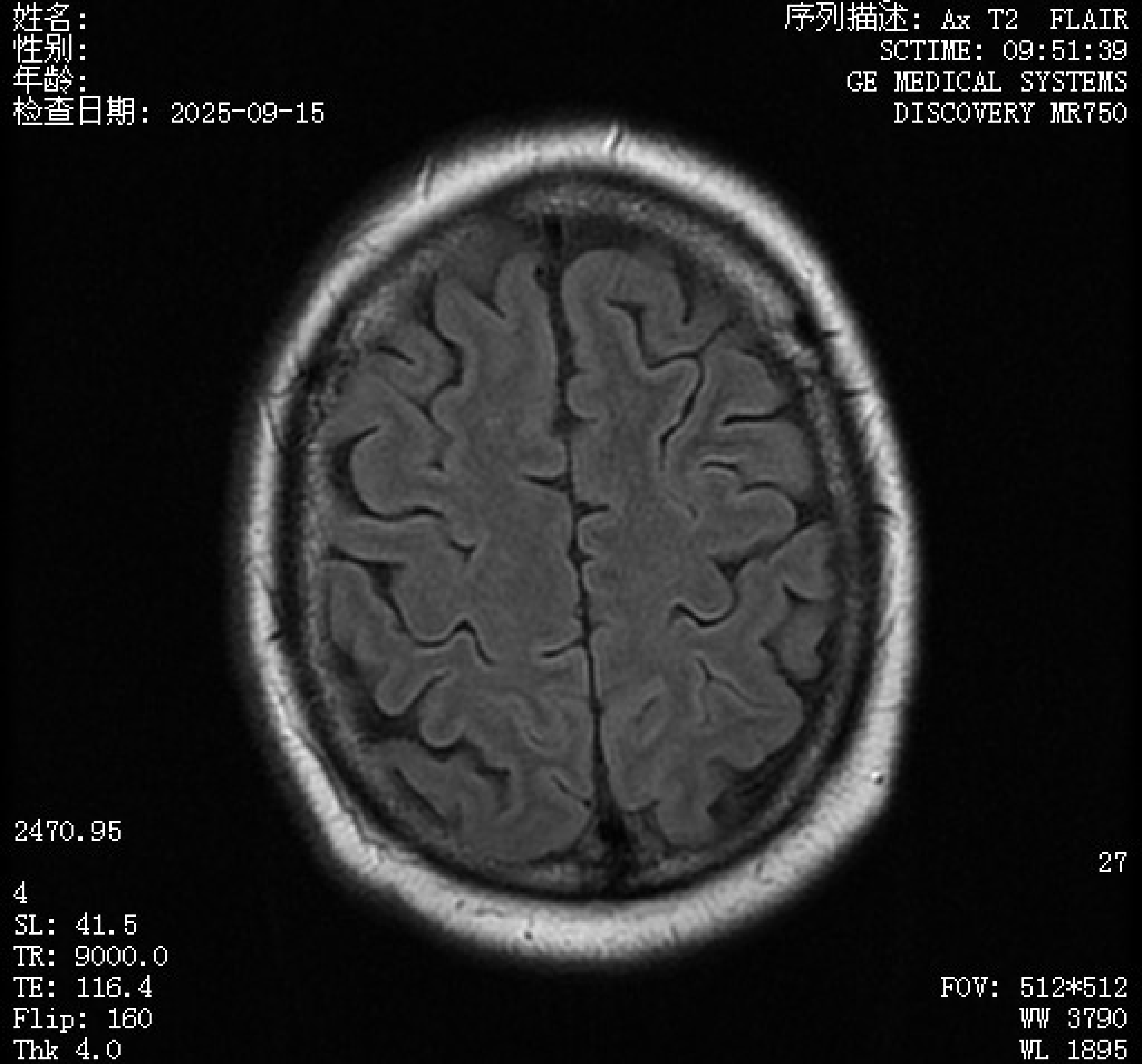
姓名:
性别:
年龄:
检查日期: 2025-09-15
序列描述: Ax T2 FLAIR
SCTIME: 09:51:39
GE MEDICAL SYSTEMS
DISCOVERY MR750
2470.95
27
4
SL: 41.5
TR: 9000.0
TE: 116.4
Flip: 160
Thk 4.0
FOV: 512*512
WW 3790
WL 1895

姓名:
性别:
年龄:
检查日期: 2025-09-15

序列描述: Ax T2 FLAIR
SCTIME: 09:51:39
GE MEDICAL SYSTEMS
DISCOVERY MR750

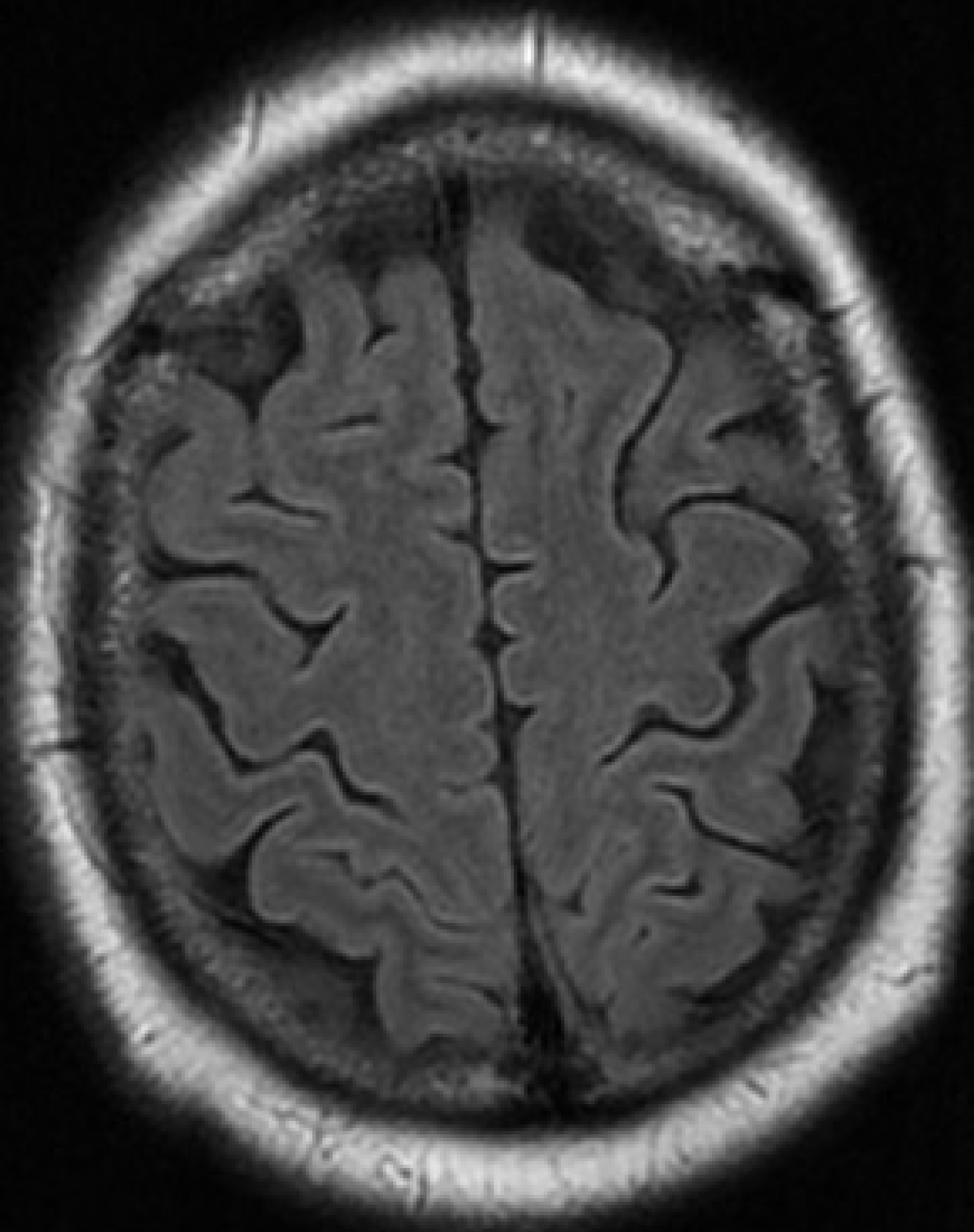

2470.95

28

4
SL: 45.9
TR: 9000.0
TE: 116.4
Flip: 160
Thk 4.0

FOV: 512*512
WW 3703
WL 1851

姓名:
性别:
年龄:
检查日期: 2025-09-15

序列描述: Ax T2 FLAIR
SCTIME: 09:51:39
GE MEDICAL SYSTEMS
DISCOVERY MR750

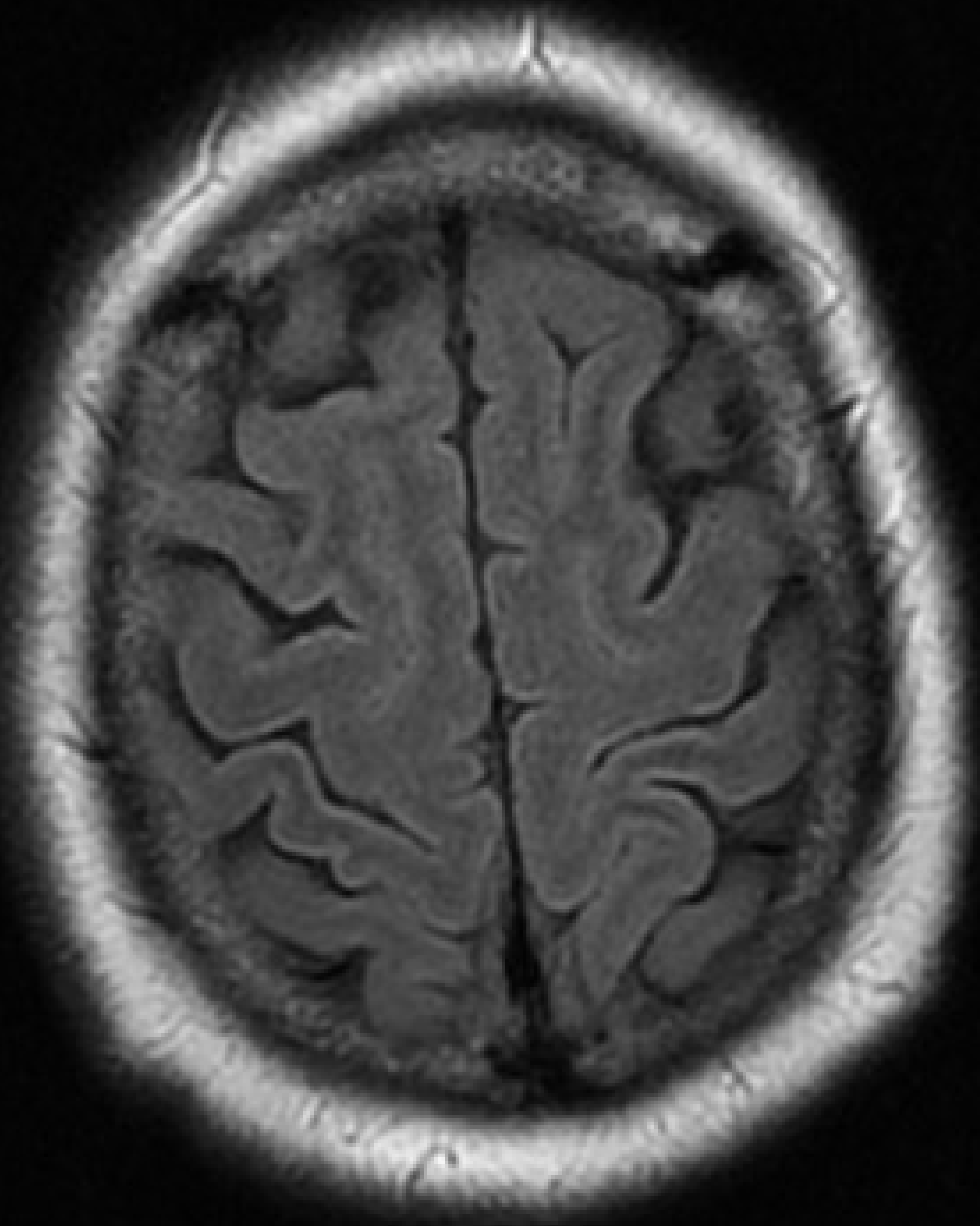

2470.95

29

4
SL: 50.3
TR: 9000.0
TE: 116.4
Flip: 160
Thk 4.0

FOV: 512*512
WW 3703
WL 1851

姓名:
性别:
年龄:
检查日期: 2025-09-15

序列描述: Ax T2 FLAIR
SCTIME: 09:51:39
GE MEDICAL SYSTEMS
DISCOVERY MR750

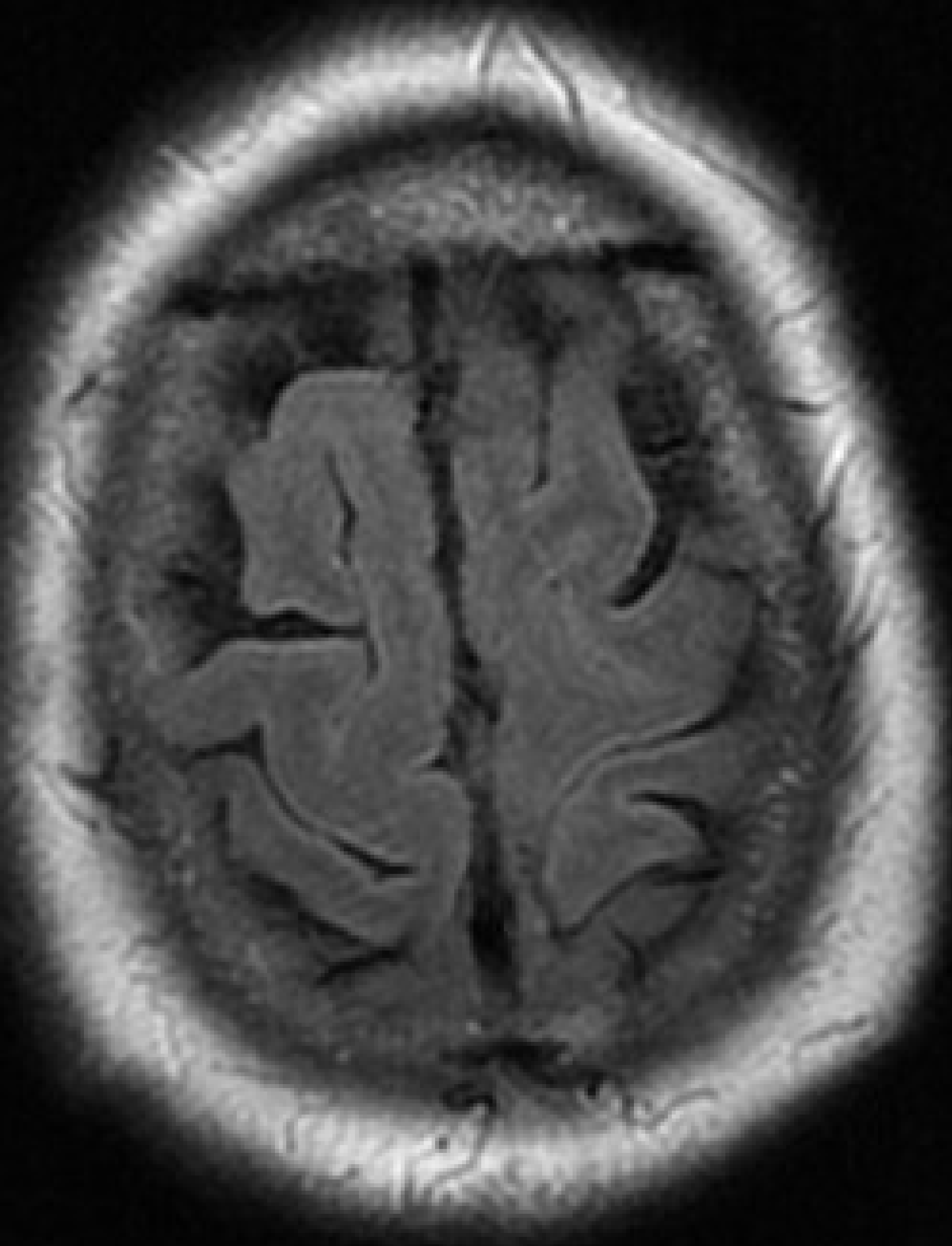

2470.95

30

4
SL: 54.7
TR: 9000.0
TE: 116.4
Flip: 160
Thk 4.0

FOV: 512*512
WW 3696
WL 1848
